# Supplementary material for: Global SARS-CoV-2 seroprevalence from January 2020 to April 2022: A systematic review and meta-analysis of standardized population-based studies
Source: PLoS Med. 2022 Nov 10;19(11):e1004107. doi: 10.1371/journal.pmed.1004107 (PMC9648705; doi:10.1371/journal.pmed.1004107)
Supplement: S1 Materials — File A. PRISMA checklist. File B. Search strategy. File C. PROSPERO protocol registration. File D. Criteria for WHO Unity alignment of seroepidemiological investigations. File E. SeroTracker inclusion and exclusion criteria. File F. Risk of bias tool breakdown. File G. Screening and extraction methods. File H. Supplementary analysis information. Table A. Studies used in ascertainment analysis. Table B. Additional studies used in meta-analysis and meta-regression. Table C. Additional studies used in descriptive analysis. Table D. Risk of bias breakdown for all studies. Table E. Estimated seroprevalence over time by region and globally (uncorrected for test characteristics). Table F. Estimated seroprevalence over time by region and globally (corrected for test characteristics). Table G. Meta-regression results and model comparison. Fig A. WHO Member States with seroprevalence data identified. Fig B. Risk of bias assessment for included studies. Fig C. Asymptomatic prevalence in SEROPREV protocol studies by age and sex. (DOCX) [file pmed.1004107.s001.docx]

**S1 Materials**

[**Supplementary Files**](#_heading=h.1lgyw1mc3yfr) **2**

[File A: PRISMA Checklist](#_heading=h.1y810tw) 2

[File B: Search Strategy](#_heading=h.4i7ojhp) 4

[File C: PROSPERO Protocol Registration](#_heading=h.2xcytpi) 6

[File D: Criteria for WHO Unity-alignment of sero-epidemiological investigations](#_heading=h.1ci93xb) 13

[File E: SeroTracker inclusion and exclusion criteria](#_heading=h.3whwml4) 16

[File F: Risk of Bias Tool Breakdown](#_heading=h.2bn6wsx) 17

[File G: Screening and extraction methods](#_heading=h.qsh70q) 20

[File H: Supplementary analysis information](#_heading=h.3as4poj) 21

[**Supplementary Tables**](#_heading=h.6svbpe7tzrot) **27**

[Table A. Studies used in ascertainment analysis.](#_heading=h.49x2ik5) 27

[Table B. Additional studies used in meta-analysis and meta-regression.](#_heading=h.41mghml) 87

[Table C. Additional studies used in descriptive analysis.](#_heading=h.3tbugp1) 125

[Table D. Risk of bias breakdown for all studies.](#_heading=h.1664s55) 226

[Table E. Estimated seroprevalence over time by region and globally (uncorrected for test characteristics)](#_heading=h.2iq8gzs) 334

[Table F. Estimated seroprevalence over time by region and globally (corrected for test characteristics)](#_heading=h.xvir7l) 357

[Table G. Meta-regression results and model comparison.](#_heading=h.3hv69ve) 373

[**Supplementary figures**](#_heading=h.1x0gk37) **416**

[Fig A. WHO Member States with seroprevalence data identified.](#_heading=h.4h042r0) 417

[Fig B. Risk of Bias Assessment for Included Studies.](#_heading=h.2w5ecyt) 419

[Fig C. Asymptomatic Prevalence in SEROPREV Protocol Studies by Age and Sex.](#_heading=h.1baon6m) 420

#

#

#

# Supplementary Files

## File A: PRISMA Checklist

PRISMA Checklist [1]

| **Section and Topic** | **Item #** | **Checklist item** | **Location where item is reported** |
| --- | --- | --- | --- |
| **TITLE** | | |  |
| Title | 1 | Identify the report as a systematic review. | Title |
| **ABSTRACT** | | |  |
| Abstract | 2 | See the PRISMA 2020 for Abstracts checklist. | Abstract |
| **INTRODUCTION** | | |  |
| Rationale | 3 | Describe the rationale for the review in the context of existing knowledge. | Introduction, paragraphs 1 through 4 |
| Objectives | 4 | Provide an explicit statement of the objective(s) or question(s) the review addresses. | Introduction, last paragraph |
| **METHODS** | | |  |
| Eligibility criteria | 5 | Specify the inclusion and exclusion criteria for the review and how studies were grouped for the syntheses. | Methods, paragraphs 2 and 3; File D and E in S1 Materials |
| Information sources | 6 | Specify all databases, registers, websites, organisations, reference lists and other sources searched or consulted to identify studies. Specify the date when each source was last searched or consulted. | Methods, paragraph 1; File B in S1 Materials |
| Search strategy | 7 | Present the full search strategies for all databases, registers and websites, including any filters and limits used. | File B in S1 Materials |
| Selection process | 8 | Specify the methods used to decide whether a study met the inclusion criteria of the review, including how many reviewers screened each record and each report retrieved, whether they worked independently, and if applicable, details of automation tools used in the process. | Methods, paragraph 2, lines 1-8 |
| Data collection process | 9 | Specify the methods used to collect data from reports, including how many reviewers collected data from each report, whether they worked independently, any processes for obtaining or confirming data from study investigators, and if applicable, details of automation tools used in the process. | Methods, paragraph 2 lines 1-8; paragraph. 4 |
| Data items | 10a | List and define all outcomes for which data were sought. Specify whether all results that were compatible with each outcome domain in each study were sought (e.g. for all measures, time points, analyses), and if not, the methods used to decide which results to collect. | Methods, paragraph 4 |
|  | 10b | List and define all other variables for which data were sought (e.g. participant and intervention characteristics, funding sources). Describe any assumptions made about any missing or unclear information. | Methods, paragraph 4; paragraph 8; File G in S1 Materials |
| Study risk of bias assessment | 11 | Specify the methods used to assess risk of bias in the included studies, including details of the tool(s) used, how many reviewers assessed each study and whether they worked independently, and if applicable, details of automation tools used in the process. | Methods, paragraph 6; File F in S1 Materials |
| Effect measures | 12 | Specify for each outcome the effect measure(s) (e.g. risk ratio, mean difference) used in the synthesis or presentation of results. | Methods, paragraphs 11-14 |
| Synthesis methods | 13a | Describe the processes used to decide which studies were eligible for each synthesis (e.g. tabulating the study intervention characteristics and comparing against the planned groups for each synthesis (item #5)). | Methods, paragraph 9 |
|  | 13b | Describe any methods required to prepare the data for presentation or synthesis, such as handling of missing summary statistics, or data conversions. | Methods, paragraph 7; paragraph 9; File H in S1 Materials |
|  | 13c | Describe any methods used to tabulate or visually display results of individual studies and syntheses. | Methods, paragraph 12 lines 2-7 |
|  | 13d | Describe any methods used to synthesize results and provide a rationale for the choice(s). If meta-analysis was performed, describe the model(s), method(s) to identify the presence and extent of statistical heterogeneity, and software package(s) used. | Methods, paragraphs 10-14 |
|  | 13e | Describe any methods used to explore possible causes of heterogeneity among study results (e.g. subgroup analysis, meta-regression). | Methods, paragraph 10; lines 311-316 |
|  | 13f | Describe any sensitivity analyses conducted to assess robustness of the synthesized results. | Methods, paragraph 15; File H in S1 Materials |
| Reporting bias assessment | 14 | Describe any methods used to assess risk of bias due to missing results in a synthesis (arising from reporting biases). | N/A |
| Certainty assessment | 15 | Describe any methods used to assess certainty (or confidence) in the body of evidence for an outcome. | Methods, paragraph 13 |
| **RESULTS** | | |  |
| Study selection | 16a | Describe the results of the search and selection process, from the number of records identified in the search to the number of studies included in the review, ideally using a flow diagram. | Results, paragraph 1; Fig 1 |
|  | 16b | Cite studies that might appear to meet the inclusion criteria, but which were excluded, and explain why they were excluded. | N/A |
| Study characteristics | 17 | Cite each included study and present its characteristics. | Table A-C in S1 Materials |
| Risk of bias in studies | 18 | Present assessments of risk of bias for each included study. | Table D in S1 Materials |
| Results of individual studies | 19 | For all outcomes, present, for each study: (a) summary statistics for each group (where appropriate) and (b) an effect estimate and its precision (e.g. confidence/credible interval), ideally using structured tables or plots. | Table A-C in S1 Materials |
| Results of syntheses | 20a | For each synthesis, briefly summarise the characteristics and risk of bias among contributing studies. | Results, paragraph 1; Table 1; Fig B in S1 Materials |
|  | 20b | Present results of all statistical syntheses conducted. If meta-analysis was done, present for each the summary estimate and its precision (e.g. confidence/credible interval) and measures of statistical heterogeneity. If comparing groups, describe the direction of the effect. | Results, paragraphs 7-11, Fig 3, Fig 4, Table 2; Fig C, Table E in S1 Materials |
|  | 20c | Present results of all investigations of possible causes of heterogeneity among study results. | Results, paragraph 6-7, Fig 2; Table G in S1 Materials |
|  | 20d | Present results of all sensitivity analyses conducted to assess the robustness of the synthesized results. | Results, last paragraph; Table F in S1 Materials |
| Reporting biases | 21 | Present assessments of risk of bias due to missing results (arising from reporting biases) for each synthesis assessed. | N/A |
| Certainty of evidence | 22 | Present assessments of certainty (or confidence) in the body of evidence for each outcome assessed. | Results Fig 2, Fig 3, Fig 4; Table E-G in S1 Materials |
| **DISCUSSION** | | |  |
| Discussion | 23a | Provide a general interpretation of the results in the context of other evidence. | Discussion, paragraph 2-3 |
|  | 23b | Discuss any limitations of the evidence included in the review. | Discussion, paragraph 6 |
|  | 23c | Discuss any limitations of the review processes used. | Discussion, paragraph 7 |
|  | 23d | Discuss implications of the results for practice, policy, and future research. | Discussion, paragrah 8-10 |
| **OTHER INFORMATION** | | |  |
| Registration and protocol | 24a | Provide registration information for the review, including register name and registration number, or state that the review was not registered. | Methods, paragraph 1, last sentence; File C in S1 Materials |
|  | 24b | Indicate where the review protocol can be accessed, or state that a protocol was not prepared. | Methods, paragraph 1, last sentence; File C in S1 Materials |
|  | 24c | Describe and explain any amendments to information provided at registration or in the protocol. | N/A |
| Support | 25 | Describe sources of financial or non-financial support for the review, and the role of the funders or sponsors in the review. | Funding section; Declaration of interests section |
| Competing interests | 26 | Declare any competing interests of review authors. | Declaration of interests, section |
| Availability of data, code and other materials | 27 | Report which of the following are publicly available and where they can be found: template data collection forms; data extracted from included studies; data used for all analyses; analytic code; any other materials used in the review. | Data availability section |

**References**

[1. Page MJ, McKenzie JE, Bossuyt PM, et al. The PRISMA 2020 statement: an updated guideline for reporting systematic reviews. *BMJ*. Published online March 29, 2021:n71. doi:10.1136/bmj.n71](https://www.zotero.org/google-docs/?vDe9BQ)

## File B: Search Strategy

Database: Ovid MEDLINE and Epub Ahead of Print, In-Process & Other Non-Indexed Citations and Daily

Dates: January 1, 2020 to May 20, 2022

Notes: Covid-19 search terms were adapted from Ovid Expert Searches

| **#** | **Search terms** |
| --- | --- |
| 1 | exp Coronavirus/ |
| 2 | exp Coronavirus Infections/ |
| 3 | (coronavirus* or corona virus* or OC43 or NL63 or 229E or HKU1 or HCoV* or ncov* or covid* or sars-cov* or sarscov* or Sars-coronavirus* or Severe Acute Respiratory Syndrome Coronavirus*).tw,kf.[EB2] |
| 4 | or/1-3 |
| 5 | 4 not ((MERS or MERS-CoV or Middle East respiratory syndrome or camel* or dromedar* or equine or coronary or coronal or covidence* or covidien or influenza virus or HIV or bovine or calves or TGEV or feline or porcine or BCoV or PED or PEDV or PDCoV or FIPV or FCoV or SADS-CoV or canine or CCov or zoonotic or avian influenza or H1N1 or H5N1 or H5N6 or IBV).mp. or (animals/ not humans/)) |
| 6 | ((pneumonia or covid* or coronavirus* or corona virus* or ncov* or 2019-ncov or sars* or virus).tw,kf. or exp pneumonia/) and Wuhan.tw,kf. |
| 7 | (2019-ncov* or 2019nCov* or ncov19 or ncov-19 or 2019-novel CoV or sars-cov2* or sars-cov-2* or sarscov2* or sarscov-2* or Sars-coronavirus2 or Sars-coronavirus-2 or SARS-like coronavirus* or coronavirus 2 or coronavirus2* or corona or coronavirus-19 or covid19 or covid-19 or covid 2019 or ((novel or new or nouveau) adj2 (CoV or nCoV or covid or coronavirus* or corona virus or Pandemi*2)) or ((covid or covid19* or covid-19) and pandemic*2) or (coronavirus* and pneumonia)).tw,kf. |
| 8 | COVID-19.rx,px,ox. or severe acute respiratory syndrome coronavirus 2.os. |
| 9 | or/6-8 |
| 10 | 5 or 9 |
| 11 | immunoglobulins/ or antibodies/ or antibodies, blocking/ or exp antibodies, neutralizing/ or antibodies, viral/ or antigen-antibody complex/ or immune sera/ or exp immunoglobulin isotypes/ or immunoglobulin a/ or immunoglobulin d/ or immunoglobulin e/ or immunoglobulin g/ or immunoglobulin m/ |
| 12 | serologic tests/ or complement fixation tests/ or hemagglutination inhibition tests/ or neutralization tests/ |
| 13 | immunoassay/ or fluoroimmunoassay/ or exp immunoblotting/ or immunoenzyme techniques/ or exp enzyme-linked immunosorbent assay/ or exp enzyme-linked immunospot assay/ or immunosorbent techniques/ or serologic tests/ or complement fixation tests/ or hemagglutination inhibition tests/ or neutralization tests/ or Serology/di |
| 14 | (enzyme linked immunosorbent or enzyme-linked immunosorbent or ELISA or immunofluorescence or complement fixation or hemagglutination inhibition or immunoblot or western blot or neutrali*).tw,kf. |
| 15 | (antibod* or immunoglobulin* or immune globulin* or titer* or isotype* or IgG or IgM or IgA or neutrali* or sera or serum or serolog* or saliva).tw,kf. |
| 16 | or/11-14 |
| 17 | seroepidemiologic studies/ |
| 18 | incidence/ or prevalence/ |
| 19 | (seroconver* or seroprevalence or sero-prevalence or seroincidence or sero-incidence or seroepidemiolog* or sero-epidemiolog*).mp. |
| 20 | (inciden* or prevalen* or count* or rate*).mp. |
| 21 | (serosurvey or sero-survey or screen* or diagnostic).mp. |
| 22 | (seroconver* or seroprevalence or sero-prevalence or seroincidence or sero-incidence or seroepidemiolog* or sero-epidemiolog* or inciden* or prevalen* or silent or asymptomatic or serosurvey or sero-survey).tw,kf. |
| 23 | or/17-21 |
| 24 | 10 and (16 and 23) |
| 25 | 10 and 15 |
| 26 | 10 and 22 |
| 27 | or/24-26 |
| 28 | limit 27 to yr="2020-Current" |
| 29 | remove duplicates from 28 |

## File C: PROSPERO Protocol Registration

The following protocol is pulled from our most recent PROSPERO protocol registration (October 2021 version) CRD42020183634.

**Review question**

Our objective is to provide ongoing up-to-date synthesis of SARS-CoV-2 seroprevalence surveys from studies aligned with the WHO Unity sero-epidemiological protocol around the world.

**Searches**

We will search four electronic peer-review literature databases (MEDLINE, EMBASE, Web of Science, and Cochrane Database of Systematic Reviews) from January 1, 2020 until the end of the SARS-CoV-2 pandemic (according to WHO) using a comprehensive set of search terms developed in consultation with a health sciences librarian.

We will search grey literature in two ways. First, we will search for pre-print articles through Europe PMC electronic databases. When we encounter reports of potentially eligible government studies we will conduct a targeted search to locate and include the government study.

We will conduct two types of secondary searches. First, we will consult with international experts via e-mail to identify additional literature after all other sources have been searched. Second, we will invite submission of seroprevalence study results on our live dashboard SeroTracker.com. Third, we will coordinate with WHO regional offices to identify seroepidemiological investigators and studies that are receiving support from WHO including financial support, equipment (e.g., serological assays), or expertise.

**Types of study to be included**

We will include cross-sectional studies (single time point or repeated/panel design) and cohort studies. All other study designs will be excluded.

Studies will also be excluded if they have unclear sampling frames, sampling methods other than convenience or random sampling, and studies deliberately excluding cases of COVID-19. These criteria mirror the standards of the WHO sero-epidemiological Unity protocol.

**Condition or domain being studied**

Severe acute respiratory syndrome coronavirus 2.

**Participants/population**

Humans, any age. We will include studies whose participants fall into the category of “general population” as defined by the WHO Unity sero-epidemiological studies: Household and community samples, residual sera, outpatients, samples drawn from general population surveys or registries, outpatients, pregnant and parturient women.

**Intervention(s), exposure(s)**

We will include studies conducting serological testing – defined as the collection and testing of serum (or proxy such as oral fluid) specimens from a sample of a defined population over a specified period of time – to estimate the prevalence of antibodies against SARS-CoV-2 in the general population.

Studies will be excluded if they do not meet the serological assay performance threshold of 90% sensitivity and 97% specificity, as reported by the manufacturer (or author-evaluated in-house test, if manufacturer data is not present). An exception will be made for studies in a humanitarian country, as data from these settings may be sparse and their inclusion is essential for a global understanding of SARS-CoV-2 seroprevalence. These criteria mirror the standards for the WHO sero-epidemiological Unity protocol.

**Comparator(s)/control**

None

**Context**

All settings are eligible. Exceptions to the inclusion criteria will be made for studies in humanitarian settings to maximize inclusion of data from those resource limited settings.

**Main outcome(s)**

The outcome of interest was seroprevalence (the proportion of the population with detectable antibodies).

***Measures of effect***

Proportions

**Additional outcome(s)**

There are two additional outcomes of interest: the ratio of confirmed cases (according to nucleic acid testing) to seroprevalence (according to serology testing); and the prevalence ratios between subgroups, e.g. age, sex, and vaccination status.

***Measures of effect***

Ratio

Prevalence ratio

**Data extraction (selection and coding)**

References will be uploaded to Covidence, de-duplicated, and screened (first by title and abstract, then by reading full-text) by two independent researchers. Discrepancies in screening and extraction will be resolved by arbitration with a third author. We will contact study investigators in order to obtain complete data. The research team has members proficient in reading sources written in English, French, Spanish, Portuguese, Croatian, Serbian, Romanian. Other non-English language data sources will be read via Google Translate.

Included articles will be uploaded into a purpose-built Airtable database for data extraction. Data will be extracted on the characteristics of the study, sample (age, sex, vaccination subgroups), antibody test name, and the prevalence estimates. We will extract sub-group data based on the stratification of one variable (e.g., age) but not two variables (e.g., age and ethnicity). To contextualize prevalence estimates we will extract the number of total confirmed cases from the Johns Hopkins COVID-19 tracker (country level data) with population denominators from the UN Population Division estimates. We will extract the number of confirmed cases at the median date and end date for each prevalence study. We will also extract the number of total people vaccinated from the Our World in Data tracker (country level data) at the median date and end date for each prevalence study.

Data from unpublished studies will be included from investigators participating in the WHO Unity Studies collaboration. These data will be submitted using standardized templates, extracted directly into R, and analyzed as part of this study. Results from these unpublished studies will be housed temporarily on an open-access database to ensure reproducibility until authors have published their results, then the published version will replace any unpublished data in our database.

**Risk of bias (quality) assessment**

We will assess risk of bias for each study using a modified version of the Joanna Briggs Critical Appraisal Checklist for Prevalence Studies.

Two reviewers will provide independent assessments for two of the JBI items (item 1, item 5), while the other seven items will be automatically completed based on the other data elements extracted for the review . These nine items will be synthesized to generate a summary risk of bias assessment (low, moderate, high, unclear), determined using a decision rule (SeroTracker-ROB).

**Strategy for data synthesis**

The purpose of the analysis is to gather, synthesize, critically appraise, and present the best available estimates of seroprevalence in the general population to inform decision making in different regions and populations. We will summarize findings from all included studies. We will synthesize prevalence estimates with 95% confidence intervals for all WHO regions, time periods, and particular groups for which data is available (e.g., age, sex, vacacinated vs non-vaccinated individuals, symptomatic vs asymptomatic individuals). Where there are multiple estimates within each country and time period, we will aggregate these studies with random-effects meta-analysis where deemed appropriate.

We will compare seroprevalence estimates to the cumulative incidence of confirmed cases to understand the gap between these two figures. We will use meta-regression to identify the effects of study- and geographic-level factors on seroprevalence estimates, such as overall risk of bias, geographic scope, WHO region, income class, and severity of public health and social measures (PHSM).

**Analysis of subgroups or subsets**

We will conduct analyses overall and grouped by WHO geographical region. We will stratify the EURO and PAHO WHO regions by World Bank income level (high-income countries (HIC) vs. low-, lower-middle, and upper-middle-income countries (LMIC)).

We will analyze the seroprevalence ratio between different subpopulations within the same study. Variables of interest for this analysis include sex groups and age groups.

Sensitivity analyses will be conducted by stratifying studies based on risk of bias. To compare antibody kinetics and infection- and vaccination-induced seroprevalence in countries using S-based vaccines we will also stratify seroprevalence estimates for studies measuring Spike protein antibodies (anti-S) and studies measuring Nucleocapsid protein antibodies (anti-N)

**Contact details for further information**

Dr Isabel Bergeri

bergerii@who.int

**Organisational affiliation of the review**

World Health Organization, https://www.who.int/

Epiconcept, https://www.epiconcept.fr/en/

University of Calgary, https://www.ucalgary.ca/

University of Toronto, https://www.utoronto.ca/

University of Oxford, https://www.ox.ac.uk/

McGill University, https://www.mcgill.ca/

**Review team members and their organisational affiliations**

Dr Niklas Bobrovitz. University of Toronto

Mr Rahul Arora. University of Oxford

Ms Emily Boucher. University of Calgary

Ms Tingting Yan. University of Toronto

Ms Hannah Rahim. University of Calgary

Mr Jordan Van Wyk. University of Waterloo

Mr Nathan Duarte. University of Waterloo

Mr Austin Atmaja. University of Waterloo

Ms Simona Rocco. University of Waterloo

Mr Abel Joseph. University of Waterloo

Mr Ewan May. University of Calgary

Mr Michael Liu. Harvard University

Ms Claire Donnici. University of Calgary

Mr Christian Cao. University of Calgary

Ms Natasha Illincic. University of Toronto

Mr Lucas Penny. University of Toronto

Mr Mitchell Segal. University of Toronto

Assistant/Associate Professor Tyler Williamson. University of Calgary

Professor Jim Kellner. University of Calgary

Assistant/Associate Professor Cedric Yansouni. McGill University

Assistant/Associate Professor Jesse Papenburg. McGill University

Professor Timothy Evans. McGill University

Assistant/Associate Professor Jonathan Chevrier. McGill University

Assistant/Associate Professor Matthew Cheng. McGill University

Ms Mairead Whelan. University of Calgary

Miss Harriet Ware. University of Calgary

Miss Xiaomeng Ma. University of Toronto

Mr Zihan Li. University of Waterloo

Dr Isabel Bergeri. World Health Organization

Miss Brianna Cheng. World Health Organization

Mrs Hannah Lewis. World Health Organization

Dr Anthony Nardone. Epiconcept

Dr Marta Valenciano. Epiconcept

Dr Lorenzo Subissi. World Health Organization

**Collaborators**

Miss Lubna Al Ariqi. World Health Organization, Regional Office for the Eastern Mediterranean

Dr Andrea Vicari. Pan American Health Organization

Dr Joseph Okeibunor. World Health Organization, Regional Office for Africa

Dr Tasnim Azim. World Health Organization, Regional Office for South-East Asia

Dr Pushpa Wijesinghe. World Health Organization, Regional Office for South-East Asia

Eman Abdelkreem. World Health Organization, Regional Office for the Eastern Mediterranean

Dr Aisling Vaughan. World Health Organization, Regional Office for Europe

Dr Richard Pebody. World Health Organization, Regional Office for Europe

Dr Linh-Vi Ve. World Health Organization, Regional Office for the Western Pacific

**Type and method of review**

Epidemiologic, Meta-analysis, Systematic review

**Anticipated or actual start date**

16 April 2020

**Anticipated completion date**

30 June 2022

**Funding sources/sponsors**

Public Health Agency of Canada 2021-HQ-000056 - operational funding for review and for SeroTracker.com, plus other non-review activities.

WHO Unity Studies, with funding to World Health Organization by the COVID-19 Solidarity Response Fund and the German Federal Ministry of Health (BMG) COVID-19 Research and development (Ref:2021/1114309-0).

***Grant number(s)***

***State the funder, grant or award number and the date of award***

Public Health Agency of Canada 2021-HQ-000056

World Health Organization Ref:2021/1114309-0

**Conflicts of interest**

**Language**

English

**Country**

Canada, Congo, Denmark, Egypt, England, India, Namibia, Philippines, Switzerland, United States of America

**Stage of review**

Review Completed published being updated

**Details of final report/publication(s) or preprints if available**

Niklas Bobrovitz, Rahul Krishan Arora, Christian Cao, Emily Boucher, Michael Liu, Claire Donnici, Mercedes Yanes-Lane, Mairead Whelan, Sara Perlman-Arrow, Judy Chen, Hannah Rahim, Natasha Ilincic, Mitchell Segal, Nathan Duarte, Jordan Van Wyk, Tingting Yan, Austin Atmaja, Simona Rocco, Abel Joseph, Lucas Penny, David A. Clifton, Tyler Williamson, Cedric P. Yansouni, Timothy Grant Evans, Jonathan Chevrier, Jesse Papenburg, Matthew P. Cheng. Global seroprevalence of SARS-CoV-2 antibodies: A systematic review and meta-analysis. PLOS One. Published: June 23, 2021. https://doi.org/10.1371/journal.pone.0252617

<https://journals.plos.org/plosone/article?id=10.1371/journal.pone.0252617>

**Subject index terms status**

Subject indexing assigned by CRD

**Subject index terms**

COVID-19; Humans; SARS Virus; Seroepidemiologic Studies; severe acute respiratory syndrome coronavirus 2

**Date of registration in PROSPERO**

07 May 2020

**Date of first submission**

02 May 2020

**Details of any existing review of the same topic by the same authors**

**Stage of review at time of this submission**

| **Stage** | **Started** | **Completed** |
| --- | --- | --- |
| Preliminary searches | Yes | Yes |
| Piloting of the study selection process | Yes | Yes |
| Formal screening of search results against eligibility criteria | Yes | No |
| Data extraction | No | No |
| Risk of bias (quality) assessment | No | No |
| Data analysis | No | No |

**Revision note**

Our previous review focused on published studies and used primarily descriptive analysis. This updated protocol includes the following changes to expand the robustness of our analysis and data sources: - Focuses on studies whose design aligns with the WHO Unity protocols, thereby updating inclusion criteria - Uses an expanded search strategy to incorporate ongoing, unpublished data accessible through WHO networks and stakeholders which will be made available in an open-access platform to assure reproducibility - New analysis of seroprevalence data including global meta-analysis and seroprevalence over time - New approach to automated critical appraisal and risk of bias assessment using a validated tool - Addition of team members and collaborators with expertise in global health, infectious diseases epidemiology, and biostatistics

*The record owner confirms that the information they have supplied for this submission is accurate and complete and they understand that deliberate provision of inaccurate information or omission of data may be construed as scientific misconduct.*

*The record owner confirms that they will update the status of the review when it is completed and will add publication details in due course.*

## File D: Criteria for WHO Unity-alignment of sero-epidemiological investigations

The primary aim of the Unity Studies is to contribute to global standardization of sero-epidemiological studies, which in turn will facilitate international comparison of data. The aim is to collect estimates at population level rather than in specific populations. To achieve this, the study protocol “Population-based age-stratified seroepidemiological investigation protocol for coronavirus 2019 (COVID-19) infection” [1] contains criteria outlining methodological approaches related to study design, data collection and analysis. The following key criteria should be adhered to:

- **Study population:** General population, any age group. Age stratification is not required. For studies not providing age-stratification, authors will be requested to provide that data if available.
- **Study design**: A one-time cross-sectional; or repeat cross sectional study; or a longitudinal cohort study. There is no sample size requirement.
- **Geographic scope**: national, sub-national, or local (ideally representative of overall burden of infection)
- **Sampling**: Both random sampling or convenience sampling is acceptable
- ***Random Sampling***: Individuals can be approached to participate in investigations using a variety of probability sampling techniques.
- ***Convenience Sampling***: Where a robust sampling frame can be described that approximates to a wider population, convenience samples are included as UNITY studies. Thus, for example, individuals attending medical services (e.g. blood donors, pregnant mothers, primary care attendees, etc.) or residual sera taken from patients for other a variety of other investigations can be included. Sampling of specific (e.g. healthcare workers) or closed (e.g. residents of institutions such as boarding schools or prisons) populations or recruited without a sampling frame (e.g. through advertising or volunteers at a venue) are not included.
- There may be circumstances where a study makes use of a patient population for a prevalence disease (e.g. some HIV clinics) as a proxy for the general population, subject to evaluation by the Unity team for inclusion on a case-by-case basis
- **Recruitment**: Study participants can be recruited through existing studies (e.g. general health surveys, research population, or patient cohorts (non-COVID-19 patient cohorts, etc.) or by establishing new studies.
- **Inclusion criteria**: All individuals identified for recruitment into the investigation, irrespective of acute or prior COVID-19 infection. All attempts should be made to include participants over a range of ages.
- **Exclusion criteria**: Refusal to give informed consent, or contraindication to venipuncture.

Suspected or confirmed acute or prior COVID-19 infection should not be excluded, with an exception for excluding active COVID-19 cases (for research staff safety reasons or local isolation and public health protocols).

- **Serological testing method**: Serological testing may be carried out using any testing method (and may use multiple assays), whether enzyme linked immunosorbent assay (ELISA), immunofluorescence assay (IFA), or rapid diagnostic test (RDT), **as long as they meet the specified sensitivity and specificity thresholds as per below.**
  - Test should be validated for the specimen type being used
  - Test must meet 90% sensitivity, 97% specificity or higher as reported by the manufacturer (or author-evaluated in-house test, if manufacturer data is not present)
- **An exception regarding the use of assays below a certain sensitivity and specificity can be made for studies conducted in** **humanitarian settings** (63 countries).
  - We will include all assay types from HRP countries regardless of their reported sensitivity and specificity, as long as the authors report an assay which has been independently validated from either an in-house evaluation or a WHO-approved head-to-head evaluation
- **Ethical clearance**: Availability of ethical approval/exception

Sero-epidemiological investigations that are conducted in line with the key criteria outlined above can be considered as **‘Unity-aligned’**.

While the criteria listed above are broad and inclusive, accounting for various circumstances and offering many possibilities for investigators to collect data, there are several methodological aspects that are **not acceptable** for ‘Unity-alignment’:

- There may be circumstances where probability sampling techniques cannot be used and therefore non-probability sampling can be justified. In this case it is pivotal to make sure that the sampling frame clearly corresponds to the study population. For example, while it is acceptable to approach a clearly defined number of individuals attending specific medical services within a pre-defined time frame, it is not acceptable to call for participants/volunteers where it is not possible to establish representativeness of the study population at large.
- Studies for which robust sampling frames that do not approximate to wider populations (e.g. through advertising or volunteers at a venue) or that sample specific (e.g. healthcare workers) or closed (e.g. residents of institutions such as boarding schools or prisons) populations.
- Use of serological assays below a certain sensitivity (90%) and specificity (97%) threshold, unless countries meet the HRP exception criteria explained above.
- Use of multiple assays with a non-overlapping algorithm (study uses two or more different tests for different subsets of samples)

Unity-aligned vs not unity-aligned sero-epidemiological studies

|  | **Unity-aligned** | **Not Unity-aligned** |
| --- | --- | --- |
| **Study population** | Age-stratified sample from general population | Populations with substantial comorbidities or elevated exposure to COVID-19 |
| **Potential output and analysis** | - Seroprevalence of antibodies to COVID-19   Cumulative incidence of infection/Infection attack rates   - Fraction of asymptomatic infection - (Case fatality ratio) | Any other outcomes |
| **Study design** | Population-based, age-stratified prospective study   - One-time cross-sectional investigation - Repeated cross-sectional investigation in the same geographic area (but not sampling the same individuals) - Longitudinal cohort investigation with serial sampling of the same individuals each time |  |
| **Geographic scope** | - National - Sub-national - Local |  |
| **Sampling** | - Random sampling - Convenience sampling | Absence of sampling framework that approximates to a wider population |
| **Recruitment** | - Through new studies - Through existing studies |  |
| **Inclusion criteria** | - General population - All individuals identified for recruitment into the investigation, irrespective of age and irrespective of acute or prior COVID-19 infection. - All attempts should be made to include participants over a range of ages. | High-risk group i.e. those with substantial comorbidities or elevated exposure (e.g. healthcare workers with immediate exposure to COVID-19 patients) |
| **Exclusion criteria** | - Suspected or confirmed acute or prior COVID-19 infection should **not** be excluded | - Exclusion of study participants with suspected or confirmed acute or prior COVID-19 infection |
| **Testing method** | - ELISA - IFA - CLIA/CMIA - Multiplex immunoassay - Other comprehensively validated test | - Use of serological assays below sensitivity/specificity threshold.   (**except in humanitarian settings** reporting assays that have been independently validated from either an in-house evaluation or a WHO-approved head-to-head evaluation) |
| **Ethical considerations** | - Ethical clearance (i.e. ethical approval or exemption) prior to study implementation - Informed consent | - No ethical clearance (including the absence of ethical exemption) - Absence of informed consent |

**References**

[1. Bergeri I, Lewis HC, Subissi L, et al. Early epidemiological investigations: World Health Organization UNITY protocols provide a standardized and timely international investigation framework during the COVID-19 pandemic. *Influenza Other Respir Viruses*. 2021;n/a(n/a). doi:10.1111/irv.12915](https://www.zotero.org/google-docs/?vDe9BQ)

## File E: SeroTracker inclusion and exclusion criteria

Criteria for including evidence (must meet all the criteria to be included)

| **Characteristics** | **Criteria for inclusion** |
| --- | --- |
| Population | - Humans of any age   - If it’s a general population sample, it may include COVID-19 antigen positive persons and those with suspected disease. |
| Study design | - Sero-surveys – defined as the collection and testing of serum (or proxy such as oral fluid) specimens from a sample of a defined population over a specified period of time to estimate the prevalence of antibodies against SARS-CoV-2 as an indicator of immunity - Cross-sectional, repeated cross sectional, and cohort study designs, with serology measurements at single time points or repeated at multiple time points |
| *Special design | - Include systematic reviews and meta-analysis of seroprevalence studies for the purpose of tracking evidence synthesis efforts |
| Sampling | - Any sampling method |
| Types of evidence | - Published or unpublished academic literature, grey literature (government or institutional reports), or media reports. Slide deck presentations were included if we could identify the person giving the presentation and the date of the presentation |
| Outcome measures | - Reports a seroprevalence estimate (proportion of the population with detectable antibodies) - Reports the number of participants enrolled in the study (denominator) - Reports study sampling end date/week - Reports the locations at which the study took places such that they could be categorized as neighbourhood, city, state/province/territory, or country |
| Languages | - Any |

Criteria for excluding evidence (if any met then exclude)

| **Characteristics** | **Criteria for exclusion** |
| --- | --- |
| Population | - Non-human (e.g., *in silico*, animal, *in vitro*) - The study only included individuals with suspected, active, or previously diagnosed with COVID-19 using PCR, antigen testing, clinical assessment, or self-assessment - The study only included individuals vaccinated against SARS-CoV2 |
| Study design | - Study designs other than cross-sectional or cohort design: case reports, case-control studies, evaluations of serological tests, study protocols, |
| Sampling | - N/A |
| Types of evidence | - Multimedia sources of data (audio clips, video clips) were excluded due to the feasibility of extracting. Slide deck presentations were excluded if we could not identify the person giving the presentation and the date of the presentation - Dashboards not associated with a defined serology study |
| Outcome measures | - Only reports incidence or prevalence of SARS-CoV-2 antigen (as opposed to antibody) - Does not report study sampling end date/week - Does not report the number of participants included in the study (sample denominator) - Does not report the location at which the study took place |
| Language | - N/A |

We defined a seroprevalence study as the collection and testing of serum, plasma, whole blood, dried blood specimens, or saliva from a sample of a defined population over a specified period of time to estimate the proportion of the population with antibodies against SARS-CoV-2 [[4–6]](https://www.zotero.org/google-docs/?uwAmRr).

If a study sampled people with confirmed or suspected SARS-CoV-2 for the reason of obtaining estimates that were representative of the general population then the study was included. We included studies examining seroprevalence among close contacts of patients with confirmed infection. Each cluster was considered one unit, and therefore, studies of one unit (i.e., one cluster around a patient) were considered case reports and excluded.

Multimedia sources of data (audio clips, video clips) were excluded due to the feasibility of extracting. However, slide deck presentations were included if we could identify the person giving the presentation, the date of the presentation, and the location where the presentation was given.

Systematic reviews and meta-analysis of seroprevalence studies were included for the purposes of tracking evidence synthesis efforts however, these data will not be quantitatively analysed.

Assay validation studies that test the effectiveness of the assay on a sample of the population irrespective of PCR diagnostics status were included.

Articles not in English or French were included if they could be extracted in full using machine translation.

## File F: Risk of Bias Tool Breakdown

To assess risk of bias, a decision rule assigned a rating of low, moderate, or high risk of bias to each study based on the specific combination of JBI checklist ratings for that study [[1]](https://www.zotero.org/google-docs/?e6PLHh). This decision rule was developed based on guidance on estimating disease prevalence [[17,18]](https://www.zotero.org/google-docs/?Oc2SJT) and was validated against assessments derived manually by two independent reviewers for 2,070 seroprevalence studies in the SeroTracker database, showing good agreement with manual review (intraclass correlation 0.77, 95% CI 0.74-0.80) in a recent preprint [[1]](https://www.zotero.org/google-docs/?HgfqVa).

​

| **Item 1: Was the sample frame appropriate to address the target population?** | |
| --- | --- |
| Yes | Sample frame described and approximated the target population |
| No | Sample frame did not approximate the target population (e.g., blood donors do not represent general population, doctors do not represent all health care providers) |
| Exclude | Sample frame not described |
| *Notes | The term “target population” should not be taken to infer every individual from everywhere or with similar disease or exposure characteristics. Instead, give consideration to specific population characteristics in the study, including age range, gender, morbidities, medications, and other potentially influential factors. For example, a sample frame may not be appropriate to address the target population if a certain group has been used (such as those working for one organisation, or one profession) and the results then inferred to the target population (i.e. working adults). A sample frame may be appropriate when it includes almost all the members of the target population (i.e. a census, or a complete list of participants or complete registry data). |

| **Item 2: Were study participants recruited in an appropriate way?** | |
| --- | --- |
| Yes | Probability sampling method (simple or stratified random) or entire sample (e.g., an entire town) was used |
| No | Non-probability sampling |
| Exclude | Sampling method not reported |

| **Item 3: Was the sample size adequate?** | |
| --- | --- |
| Yes | >599 |
| No | <599 |
| Exclude | Sample size not reported |
| *Notes | To calculate the required sample size we used an assumed prevalence of 2.5%, which was the global average estimated by the WHO in April, 2020.^1^ Based on guidance by the Joanna Briggs Institute and published medical statistical recommendations we selected a precision value that was half the assumed prevalence (1.25%) [2,3]. We calculated a minimum sample size of 599 using these inputs:  Sample size calculation:  Where n = sample size;  Z = Z statistic for level of confidence (95%);  P = expected prevalence (2.5% WHO global estimate);  d = precision (1.25%)    In cases where the sample size calculation was provided and the required sample for 80% power was below our threshold (n<599) but was met by the study, this item was marked as yes. |

| **Item 4: Were the study subjects and setting described in detail?** | |
| --- | --- |
| Yes | Average age and distribution of gender/sex provided |
| No | Neither age or gender/sex is provided, or only one of age and gender/sex is provided |

| **Item 5: Was data analysis conducted with sufficient coverage of the identified sample?** | |
| --- | --- |
| Yes | The demographic characteristics (gender/sex, age, and ethnicity) of the sample are at least somewhat representative of the population in both the main and sub-group analyses |
| No | The demographic characteristics (gender/sex, age, and ethnicity) of the sample are not representative of the population in both the main and sub-group analyses |
| Unclear | Information is not provided about demographic characteristics of the sample (gender/sex, age, and ethnicity) |

| **Item 6: Were valid methods used for the identification of the condition?** | |
| --- | --- |
| Yes | - The serological test used met the WHO Unity Study Criteria for serological tests: sensitivity minimum 90%, specificity minimum 97% - Two test algorithms with combined sensitivity >=90% and combined specificity >=97%. Calculation varies based on whether parallel or series testing was utilized - Two test algorithms using a commercial or in-house binding assay with confirmatory testing using virus neutralization assay irrespective of combined sensitivity/specificity - Multiple testing algorithms employing three or more tests using robust strategies and well performing tests including:   - Parallel testing with two tests followed by a third confirmatory test for discordant results. ⅔ positive is defined as true positive   - Series testing with 3 tests (⅔ positive for true seropositive)   - Parallel testing with 3 tests (At least one positive tests and no negative tests) |
| No | - The serological test used did not meet the WHO Unity Study Criteria for serological tests: sensitivity minimum 90%, specificity minimum 97% - The combined sensitivity and specificity for the two test algorithm is below the threshold - Complex multiple test algorithms without a rational or robust strategy |
| Exclude | Test sensitivity and specificity not reported |

| **Item 7: Was the condition measured in a standard, reliable way for all participants?** | |
| --- | --- |
| Yes | The same serology test was used for all participants |
| No | Different serology tests were used for participants |
| Unclear | No details were provided about which participants received which serology tests |

| **Item 8: Was there appropriate statistical analysis?** | |
| --- | --- |
| Yes | Does all of the following: corrects for test characteristics, corrects for population characteristics or the sample is somewhat representative of the population (probability sampling), and provides the information necessary to determine the numerator, denominator, prevalence estimate, and confidence interval. |
| No | Does not correct for population characteristics and the sample is not likely representative of the population (non-probability sampling), does not correct for test or provide the information necessary to correct for test characteristics, or does not provide the information necessary to determine the numerator, denominator, prevalence estimate, and confidence interval. |

| **Item 9: Was the response rate adequate, and if not, was the low response rate managed appropriately?** | |
| --- | --- |
| Yes | Response rate > 60% or the demographics of the sample were a reasonable match to those of the target population |
| No | Response rate < 60% and the demographics of the sample were not a reasonable match to those of the target population |
| Unclear | Response rate not provided and it was unclear if the demographics of the sample differed from the target population |
| N/A | Response rate not applicable (e.g., blood donors, residual sera) |

| **Item 10: Overall risk of bias** | |
| --- | --- |
| Low | The estimates are very likely correct for the target population. To obtain a low risk of bias classification, all criteria must be met or departures from the criteria must be minimal and unlikely to impact on the validity and reliability of the prevalence estimate. These include sample sizes that are just below the threshold when all other criteria are met, reporting only some of characteristics of the sample, test characteristics below the threshold but corrections for the test performance, and response rates that are just below the threshold in the context of probability based sampling of an appropriate sampling frame with population weighted seroprevalence estimates. |
| Moderate | The estimates are likely correct for the target population. To obtain a moderate risk of bias classification, most criteria must be met and departures from the criteria are likely to have only a small impact on the validity and reliability of the prevalence estimates. |
| High | The estimates are not likely correct for the target population. To obtain a high risk of bias, many criteria must not be met or departures from criteria are likely to have a major impact on the validity and reliability of the prevalence estimates. |
| Unclear | There was insufficient information to assess the risk of bias. |

**References**

[1. Bobrovitz N, Noel KC, Li Z, et al. *SeroTracker-ROB: Reproducible Decision Rules for Risk of Bias Assessment of Seroprevalence Studies*. Epidemiology; 2021. doi:10.1101/2021.11.17.21266471](https://www.zotero.org/google-docs/?vDe9BQ)

## File G: Screening and extraction methods

Preliminary screening was conducted using the SeroTracker screening criteria [[1]](https://www.zotero.org/google-docs/?tDhCCq) for seroprevalence studies and then further refined by the ‘Unity’ aligned criteria (below, and S2.3), using Covidence software. Studies were then uploaded to AirTable for extraction of summary-level estimates and study factors.

Notably, published studies meeting the inclusion criteria, or otherwise deemed “Unity Aligned” beyond the May 20th cutoff date for this analysis, are extracted and updated weekly on an interactive and open-access dashboard and data platform, SeroTracker.com/Unity [[2]](https://www.zotero.org/google-docs/?wIId0q).

Study- and Estimate-level Classification Decisions

| **Geographic scope** | **Definitions** |
| --- | --- |
| National | Research was conducted across multiple regions within a country |
| Sub-national | Research was conducted across multiple sub-regions or municipalities within the same state or province. |
| Local | Research was conducted in a single county, municipality, or neighborhood. |
| **Sample Frames** | **Definitions** |
| Household and community samples/samples from existing surveillance programs | Population based studies with community or house-hold based surveys derived from nonspecific members of the general population (including members selected from previously established population surveys) |
| Blood donors | Retention samples that have already been taken from banked blood derived from blood donation practices; or samples that are taken from individuals who have donated blood. |
| Residual sera | Samples that have already been taken in health care settings for a variety of reasons other than COVID serological testing; or general groups of non-COVID patients attending healthcare that do not solely belong to a specific patient population |
| Pregnant or parturient women | Samples that have already been taken from pregnant women during routine pregnancy trimester screening; or samples taken from or parturient women. |
| Persons living in slums | Samples taken from people residing in slum dwellings. |
| Representative patient population | Patient population groups where the health condition is common/endemic enough for it to constitute a meaningful sample of the general population (Malaria, HIV) |
| Multiple general populations | Samples that are composed of multiple distinct above-described populations with a single pooled seroprevalence estimate. |
| **Sampling method** | **Definitions** |
| Convenience | Non-probability sampling based on convenient sources of specimens. The sampling method should still have a clear and defined sample frame (i.e., advertisements in public spaces are not included) |
| Sequential | Non-probability sampling based on entire sampling performed within a self-determined time interval. The sampling method should still have a clear and defined sample frame, and is often drawn from convenience samples. |
| Stratified non-probability (Quota) | Non-probability sampling based on groupings of similar units. Groupings are then sampled in a non-randomized fashion, wherein nonresponders may be also ignored and replaced |
| Probability | Probability sampling based on randomized sampling methods wherein all individuals have an equal probability of being selected. |
| **Serological assays** | **Definitions ^[3]^** |
| Multiplex | Assay that simultaneously detects presence of antibodies against multiple viral antigens (i.e., spike, nucleocapsid, envelope). |
| Rapid diagnostic test | Point-of-care diagnostic platform that can be used and evaluated directly at the testing site. Typical features include short turnaround time (<15 minutes), low cost, easy interpretation of results (typically binary), and ease of use with minimal training. |
| ELISA | Enzyme linked immunosorbent assay: Wells coated with the viral antigen of interest. After the formation of immune complexes, an enzyme-linked secondary antibody followed by the enzyme’s substrate are added, which results in chemical modification of the substrate in the presence of immune complexes, which is measured colorimetrically through spectroscopy. The relative light absorption at a particular wavelength is directly proportional to the amount of labeled complexes present. |
| CLIA | Chemiluminescent immunoassay (includes chemiluminescent microparticle immunoassays [CMIA]): Stationary solid particles or wells coated with the viral antigen of interest. After the formation of immune complexes, substrate is added which results in generation of light, the intensity of which is directly proportional to the amount of labeled complexes present. |
| IFA | Immunofluorescence assay. Antibodies bind to the SARS-CoV-2 protein of interest. Fluorescent dyes are coupled to these immune complexes in order to visualize the protein of interest using microscopy or flow cytometry |
| LFIA | Lateral flow immunoassay: A liquid sample containing the analyte of interest moves without the assistance of external forces (capillary action) through various zones of polymeric strips. Antibodies of interest can bind to immobilized antigen of interest in various zones. A secondary reporter antibody (typically functionalized with gold nanoparticles) then binds to these immune complexes resulting in visual band formation for interpretation. |
| Neutralization assay | Heat treated, inactivated serum is added to cell culture (typically Vero E6 cells). Presence of neutralizing antibodies against SARS-CoV-2 is measured by the extent of binding inhibition in cell culture inoculated with the virus . Quantification is determined by the serum dilutions needed to prevent cytopathic effects in the cell monolayer. |

**References**

1[. Bobrovitz N, Arora RK, Cao C, et al. Global seroprevalence of SARS-CoV-2 antibodies: A systematic review and meta-analysis. Khudyakov YE, ed. *PLoS ONE*. 2021;16(6):e0252617. doi:10.1371/journal.pone.0252617](https://www.zotero.org/google-docs/?vDe9BQ)

2[. Arora RK, Joseph A, Van Wyk J, et al. SeroTracker: a global SARS-CoV-2 seroprevalence dashboard. *Lancet Infect Dis*. 2021;21(4):e75-e76. doi:10.1016/S1473-3099(20)30631-9](https://www.zotero.org/google-docs/?vDe9BQ)

3[. Mohit E, Rostami Z, Vahidi H. A comparative review of immunoassays for COVID-19 detection. *Expert Rev Clin Immunol*. 2021;17(6):573-599. doi:10.1080/1744666X.2021.1908886](https://www.zotero.org/google-docs/?vDe9BQ)

## File H: Supplementary analysis information

**Estimate Prioritization**

Where multiple summary estimates were available or multiple estimates available for one subgroup, we prioritized estimates based on estimate adjustment (population adjustment, test adjustment), antibody isotypes measured (Total Ab, IgG, IgM, other), test type used (Neutralization, CLIA, ELISA, LFIA, other), and antibody targets measured (Multiple Ab targets, Spike, Nucleocapsid).

When multiple estimate adjustments were available, we prioritized as follows:

1. Test unadjusted and Population adjusted
2. Both unadjusted
3. Both adjusted
4. Test adjusted and Population unadjusted

When multiple isotypes were available, we prioritized as follows:

1. Total antibodies
2. IgG OR other antibodies
3. IgG only
4. IgM OR other antibodies
5. IgG AND other antibodies
6. IgM only
7. IgM AND other antibodies
8. Other

When multiple test types were available (i.e. from use of different tests), we prioritized as follows:

1. Neutralization
2. CLIA
3. ELISA
4. LFIA
5. Other

When multiple antibody targets were available (i.e. from use of different tests), we prioritized as follows:

1. Multiple antibody targets
2. Anti - Spike
3. Anti - Nucleocapsid

If the above defined prioritization criteria led to estimates left on equal terms, we further introduced test sensitivity and specificity (highest sensitivity and specificity) prioritization criteria. If multiple estimates were present (e.g. an extraction only contained equivalent subgroup data), we further pooled estimate information to generate a single summary estimate.

**The Python code used for our automated estimate prioritization is available on GitHub:** https://github.com/serotracker/iit-backend/blob/8059e9b905395de997f28a1a2dff5def795276ad/app/utils/estimate_prioritization/estimate_prioritization.py

**Additional analysis description**

We drew confirmed case data from the WHO COVID-19 dashboard matched to nine days before the study mid-date to account for time from infection to seroconversion [[24,25]](https://www.zotero.org/google-docs/?oYGbBL). We used data from Our World in Data dashboard to pull vaccination data, timed fourteen days before the study’s mid date [3]. We also calculated the average global public health and social measures (PHSM) stringency index value on a scale from 1 to 10 in the country of the study between the date of the 100th reported case and 14 days before the study mid date. The PHSM data was taken from a global dataset created in collaboration between the London School of Hygiene and Tropical Medicine (LSHTM) and WHO, which aggregates six indicators including masks, international travel, gatherings, schools, workplaces and businesses, and movement into one overall stringency index [[4]](https://www.zotero.org/google-docs/?3qSxOQ). Finally, we defined the transmission phase in the country of the study using the seven-day rolling average of daily confirmed cases from the WHO dashboard. We smoothed the case series using smoothing splines and identified the peaks of the series using local maxima. We then classified the study mid date as before the peak of 1st SARS-CoV-2 wave, after the peak of 1st SARS-CoV-2 wave, or after the peak of 2nd SARS-CoV-2 wave in the country of the study.

We produced estimates of seroprevalence over time by first meta-analyzing studies by country in 12-week rolling windows. For each month starting January 1, 2020, we first identified the sub-national and national studies in each region within a 12-week moving window centred on that week. If there was a single study in the window, we specified seroprevalence to be equal to that study’s estimate. If there were multiple studies in the window, we pooled the studies using a random intercept logistic regression model (rma.glmm from R package metafor) to calculate a pooled seroprevalence for that week. We then advanced the 12-week window to the next week and repeated the process.

We then produced monthly regional estimates by taking weighted averages of country estimates by population, ensuring that country contributions to these estimates are proportional to country population. The regional estimates were calculated as

$p_{r}=\Sigma w_{c}\times p_{c}$,

Where weights $w_{c}$ are weights for each country, normalized to sum to 1, and $p_{c}$ are the seroprevalence proportions in each region computed via random-effects meta-analysis (‘metaprop’). The standard error of the global estimates was calculated using the laws of variance as

$${se}_{r}=\sqrt{\Sigma w_{c}^{2}\times{se}_{c}^{2}},$$

where ${se}_{c}$ are the standard errors of the seroprevalence proportions in each country computed via metaprop on the logit scale. The confidence intervals of the regional estimates were calculated using the normal distribution as $p_{r}\pm2\times{se}_{r}.$ The prediction intervals were calculated similarly using the standard errors for prediction intervals in each country computed via metaprop on the logit scale.

We computed global estimates as the population-weighted average of the regional estimates, carrying forward the latest regional estimates to September 2021 to represent all regions.

To capture the trend in seroprevalence in each WHO region and globally, we took the weighted point estimates and fitted a flexible, smooth function of time using non-parametric regression. To anchor the trend line in the early pandemic period before the first serosurveys were conducted, we specified seroprevalence to be less than 0.1 percent before the 500th confirmed case of COVID-19 in each region.

We repeated the previous step using only national studies and used the results to compute the ratio between regional and global seroprevalence and the corresponding cumulative incidence of confirmed SARS-CoV-2 cases. This analysis measured to what extent confirmed SARS-CoV-2 cases underestimated seroprevalence, as reported by the WHO’s case definition [[5]](https://www.zotero.org/google-docs/?P1FBxl).

To produce estimates of ascertainment, we adjusted 2021 seroprevalence data for antibody target and vaccination to generate estimates of seroprevalence attributable to infection. In countries administering only vaccines using Spike (S) protein antigens (e.g., mRNA, viral vector), we calculated the ascertainment ratio from January to April 2021 using only studies that detected anti-nucleocapsid (N) seroprevalence, as a proxy for infection-induced seroprevalence. In countries administering inactivated vaccines that may generate both anti-S and anti-N responses (e.g., Sinopharm, Bharat Biotech), we adjusted the reported seroprevalence for studies between January and April 2021 using the formula

${SP}_{inf}=({SP}_{obs}-v)/(1-v)$,

Where ${SP}_{obs}$ is observed seroprevalence, $v$ is the vaccination rate in adults, and ${SP}_{inf}$is a proxy for infection-induced seroprevalence [[6]](https://www.zotero.org/google-docs/?uJ2NwI).

To examine study and geographic factors affecting seroprevalence estimates, we constructed a Poisson generalized linear mixed-effects model with log link function using the glmer function from the lme4 package in R [7]. Categorical covariates in the meta-regression, coded as indicator variables, included country WHO region (reference: Americas), country World Bank income class (reference: High income), study geographic scope (reference: national), study sample frame (reference: household and community samples), and transmission phase in the country of the study at the time of the study (reference: before peak of 1st wave). Continuous covariates included cumulative incidence of reported cases per hundred in the country of the study and LSHTM global index tracking PHSM in the country of the study. The PHSM index scale ranged from 0 (least stringent) to 10 (most stringent). We performed model comparison using all possible models that could be constructed by dropping a single *a priori* specified predictor, i.e., using $p-1$ predictors where there were $p$ predictors in total.

In a sensitivity analysis, we adjusted seroprevalence estimates for test characteristics through Bayesian measurement error models, with binomial sensitivity and specificity distributions. The sensitivity and specificity values for correction were prioritized from the WHO SARS-CoV-2 Test Kit Comparative Study conducted at the NRL Australia [1], followed by a multicentre evaluation of 47 commercial SARS-CoV-2 immunoassays by 41 Dutch laboratories [2], and from independent evaluations by study authors where author-designed assays were used.

**The Python code used for our test adjustment is available on GitHub:**

<https://github.com/serotracker/iit-backend/blob/011fdb721d82abea449206ac5c84c86684b0867a/app/namespaces/test_adjustment/test_adjustment_service.py>

**References**

1[. NRL. WHO COVID Evaluations Summary of Results; 2020 [cited 22 November 2021]. Database: nrlquality [Internet]. Available from: https://www.nrlquality.org.au/who-covid-evaluations-summary-of-results](https://www.zotero.org/google-docs/?vDe9BQ)

2. [Beld MJC van den, Murk JL, Kluytmans J, Koopmans MPG, Reimerink J, Loo IHM van, et al. Increasing the Efficiency of a National Laboratory Response to COVID-19: a Nationwide Multicenter Evaluation of 47 Commercial SARS-CoV-2 Immunoassays by 41 Laboratories. *J Clin Microbiol*. 2021;59(9):e00767-21.](https://www.zotero.org/google-docs/?vlnQXq)

3[. Ritchie H, Mathieu E, Rodés-Guirao L, et al. Coronavirus Pandemic (COVID-19); 2020 [cited 4 November 2021]. Database: Our World in Data [Internet]. Available from: https://ourworldindata.org/coronavirus](https://www.zotero.org/google-docs/?vDe9BQ)

4[*. World Health Organization; 2021 [cited 11 June 2021]. Database: WHO Coronavirus Disease (COVID-19) Dashboard [Internet]. Available from: https://covid19.who.int/*](about:blank)

5[. WHO Headquarters (HQ). WHO COVID-19 Case definition. 2020. Available from: https://www.who.int/publications-detail-redirect/WHO-2019-nCoV-Surveillance_Case_Definition-2020.2](https://www.zotero.org/google-docs/?vDe9BQ)

6[. Institute for Health metrics and Evaluation (IHME). COVID-19: Estimating the Historical Time Series of Infections. 2021. Available from: http://www.healthdata.org/special-analysis/covid-19-estimating-historical-infections-time-series](https://www.zotero.org/google-docs/?vDe9BQ)

7[. Bates D, Mächler M, Bolker B, Walker S. Fitting Linear Mixed-Effects Models Using **lme4**. *J Stat Softw*. 2015;67(1). doi:10.18637/jss.v067.i01](https://www.zotero.org/google-docs/?vDe9BQ)

# Supplementary Tables

## Table A. Studies used in ascertainment analysis.

#### Africa region

| Country (Location) | Author (Organization) | Sampling Dates (YMD) | Geographic scope | Overall risk of bias | Sample size | Age | Sex | Sampling method | Sample frame | Seroprevalence (95% CI) | Test Manufacturers - Isotypes - Names | Test Sens, Spec | Sens, Spec Source |
| --- | --- | --- | --- | --- | --- | --- | --- | --- | --- | --- | --- | --- | --- |
| Cameroon - HRP | Tiffany Harris (ICAP at Columbia University)[1] | 2020-10-20 - 2020-12-16 | National | Moderate | 9,332 | Multiple groups | All | Convenience | Household and community samples | 10.5% (95% CI 9.1-12%) | Abbott Laboratories - IgG - Abbott Architect SARS-CoV-2 IgG,Beijing Wantai Biological - IgG IgM IgA - Wantai SARS-CoV-2 Total Ab ELISA | 0.957, 1 | Complex test algorithm: Manual calculation |
| Ethiopia - HRP | Enyew Birru Tadesse (Federal Ministry of Health)[2] | 2020-06-24 - 2020-07-08 | National | Low | 16,932 | Multiple groups | All | Probability | Household and community samples | 1.9% | Abbott Laboratories - IgG - Abbott Architect SARS-CoV-2 IgG | 0.99, 1 | Validated by manufacturer |
| Gabon | Dr Paulin ESSONE NDONG, Pr Edgar Brice NGOUNGOU, Pr Ayola Akim ADEGNIKA (Laboratoire National de Santé Publique, CIRMF, CERMEL, Laboratoire CHUMEFJE, Laboratoire Bactério-virologie USS)[3] | 2021-11-28 - 2021-12-20 | National | Moderate | 3,659 | Multiple groups | All | Probability | Household and community samples | 86.8% (95% CI 85.6-87.9%) | Beijing Wantai Biological - IgG IgM IgA - Wantai SARS-CoV-2 Total Ab ELISA | 0.967, 0.975 | Validated by manufacturer |
| Ghana | Irene Owusu Donkor (Noguchi Memorial Institute for Medical Rsearch)[4] | 2021-12-12 - 2021-12-19 | National | Moderate | 1,027 | Multiple groups | All | Probability | Household and community samples | 86.7% (95% CI 84.4-88.7%) | Beijing Wantai Biological - IgG IgM IgA - Wantai SARS-CoV-2 Total Ab ELISA | 0.967, 0.975 | Validated by manufacturer |
| Ghana | Irene Owusu Donkor (Noguchi Memorial Institute for Medical Rsearch)[4] | 2021-07-01 - 2021-07-29 | National | Moderate | 2,275 | Multiple groups | All | Probability | Household and community samples | 66.9% (95% CI 64.9-68.8%) | Beijing Wantai Biological - IgG IgM IgA - Wantai SARS-CoV-2 Total Ab ELISA | 0.967, 0.975 | Validated by manufacturer |
| Ghana | Irene Owusu Donkor (Noguchi Memorial Institute for Medical Rsearch)[4] | 2021-05-03 - 2021-06-30 | National | Moderate | 2,234 | Multiple groups | All | Probability | Household and community samples | 65.9% (95% CI 63.9-67.9%) | Beijing Wantai Biological - IgG IgM IgA - Wantai SARS-CoV-2 Total Ab ELISA | 0.967, 0.975 | Validated by manufacturer |
| Ghana | Irene Owusu Donkor (Noguchi Memorial Institute for Medical Rsearch)[4] | 2021-02-13 - 2021-02-23 | National | Moderate | 401 | Multiple groups | All | Probability | Household and community samples | 29.4% (95% CI 25-34.2%) | Beijing Wantai Biological - IgG IgM IgA - Wantai SARS-CoV-2 Total Ab ELISA | 0.967, 0.975 | Validated by manufacturer |
| Kenya - HRP | Sophie Uyoga (KEMRI-Wellcome Trust Research Programme)[5] | 2021-01-03 - 2021-03-15 | National | Moderate | 3,018 | Multiple groups | All | Convenience | Blood donors | 45.3% | NA - IgG - Author designed (ELISA) -Spike | 0.927, 0.99 | Validated by independent authors/third party/non-developers |
| Kenya - HRP | Ifedayo M. O. Adetifa (KEMRI-Wellcome Trust Research Programme)[6] | 2020-08-20 - 2020-09-30 | National | Moderate | 3,723 | Adults (18-64 years) | All | Sequential | Blood donors | 9.1% | NA - IgG - Author designed (ELISA) -Spike | 0.927, 0.99 | Validated by independent authors/third party/non-developers |
| Malawi | Kondwani Jambo (Malawi-Liverpool-Wellcome Trust Clinical Research programme, Liverpool School of Tropical Medicine)[7] | 2021-07-01 - 2021-07-31 | National | Low | 288 | Multiple groups | All | Probability | Blood donors | 70.1% | Beijing Wantai Biological - IgG IgM IgA - Wantai SARS-CoV-2 Total Ab ELISA,Omega diagnostics - IgG - COVID-19 IgG ELISA | 0.928, 1 | Complex test algorithm: Manual calculation |
| Malawi | Kondwani Jambo (Malawi-Liverpool-Wellcome Trust Clinical Research programme, Liverpool School of Tropical Medicine)[7] | 2021-06-01 - 2021-06-30 | National | Low | 319 | Multiple groups | All | Probability | Blood donors | 61.8% | Beijing Wantai Biological - IgG IgM IgA - Wantai SARS-CoV-2 Total Ab ELISA,Omega diagnostics - IgG - COVID-19 IgG ELISA | 0.928, 1 | Complex test algorithm: Manual calculation |
| Malawi | Kondwani Jambo (Malawi-Liverpool-Wellcome Trust Clinical Research programme, Liverpool School of Tropical Medicine)[7] | 2021-05-01 - 2021-05-31 | National | Low | 298 | Multiple groups | All | Probability | Blood donors | 63.6% | Beijing Wantai Biological - IgG IgM IgA - Wantai SARS-CoV-2 Total Ab ELISA,Omega diagnostics - IgG - COVID-19 IgG ELISA | 0.928, 1 | Complex test algorithm: Manual calculation |
| Malawi | Kondwani Jambo (Malawi-Liverpool-Wellcome Trust Clinical Research programme, Liverpool School of Tropical Medicine)[7] | 2021-04-01 - 2021-04-30 | National | Low | 296 | Multiple groups | All | Probability | Blood donors | 64.7% | Beijing Wantai Biological - IgG IgM IgA - Wantai SARS-CoV-2 Total Ab ELISA,Omega diagnostics - IgG - COVID-19 IgG ELISA | 0.928, 1 | Complex test algorithm: Manual calculation |
| Malawi | Kondwani Jambo (Malawi-Liverpool-Wellcome Trust Clinical Research programme, Liverpool School of Tropical Medicine)[7] | 2021-03-01 - 2021-03-31 | National | Low | 298 | Multiple groups | All | Probability | Blood donors | 53.1% | Beijing Wantai Biological - IgG IgM IgA - Wantai SARS-CoV-2 Total Ab ELISA,Omega diagnostics - IgG - COVID-19 IgG ELISA | 0.928, 1 | Complex test algorithm: Manual calculation |
| Malawi | Kondwani Jambo (Malawi-Liverpool-Wellcome Trust Clinical Research programme, Liverpool School of Tropical Medicine)[7] | 2021-02-01 - 2021-02-28 | National | Low | 365 | Multiple groups | All | Probability | Blood donors | 53.1% | Beijing Wantai Biological - IgG IgM IgA - Wantai SARS-CoV-2 Total Ab ELISA,Omega diagnostics - IgG - COVID-19 IgG ELISA | 0.928, 1 | Complex test algorithm: Manual calculation |
| Malawi | Kondwani Jambo (Malawi-Liverpool-Wellcome Trust Clinical Research programme, Liverpool School of Tropical Medicine)[7] | 2021-01-01 - 2021-01-31 | National | Low | 303 | Multiple groups | All | Probability | Blood donors | 26.7% | Beijing Wantai Biological - IgG IgM IgA - Wantai SARS-CoV-2 Total Ab ELISA,Omega diagnostics - IgG - COVID-19 IgG ELISA | 0.928, 1 | Complex test algorithm: Manual calculation |
| Malawi | Kondwani Jambo (Malawi-Liverpool-Wellcome Trust Clinical Research programme, Liverpool School of Tropical Medicine)[7] | 2020-12-01 - 2020-12-31 | National | Low | 263 | Multiple groups | All | Probability | Blood donors | 11% | Beijing Wantai Biological - IgG IgM IgA - Wantai SARS-CoV-2 Total Ab ELISA,Omega diagnostics - IgG - COVID-19 IgG ELISA | 0.928, 1 | Complex test algorithm: Manual calculation |
| Malawi | Kondwani Jambo (Malawi-Liverpool-Wellcome Trust Clinical Research programme, Liverpool School of Tropical Medicine)[7] | 2020-11-01 - 2020-11-30 | National | Low | 365 | Multiple groups | All | Probability | Blood donors | 10.5% | Beijing Wantai Biological - IgG IgM IgA - Wantai SARS-CoV-2 Total Ab ELISA,Omega diagnostics - IgG - COVID-19 IgG ELISA | 0.928, 1 | Complex test algorithm: Manual calculation |
| Malawi | Matthews Kagoli/Joe Theu (Public Health Institute of Malawi(PHIM) MOH)[8] | 2020-10-14 - 2020-12-08 | National | Low | 4,261 | Multiple groups | All | Probability | Household and community samples | 7.8% | Beijing Wantai Biological - IgG IgM IgA - Wantai SARS-CoV-2 Total Ab ELISA | 0.967, 0.975 | Validated by manufacturer |
| Malawi | Kondwani Jambo (Malawi-Liverpool-Wellcome Trust Clinical Research programme, Liverpool School of Tropical Medicine)[7] | 2020-10-01 - 2020-10-31 | National | Low | 324 | Multiple groups | All | Probability | Blood donors | 21.1% | Beijing Wantai Biological - IgG IgM IgA - Wantai SARS-CoV-2 Total Ab ELISA,Omega diagnostics - IgG - COVID-19 IgG ELISA | 0.928, 1 | Complex test algorithm: Manual calculation |
| Malawi | Kondwani Jambo (Malawi-Liverpool-Wellcome Trust Clinical Research programme, Liverpool School of Tropical Medicine)[7] | 2020-09-01 - 2020-09-30 | National | Low | 309 | Multiple groups | All | Probability | Blood donors | 13% | Beijing Wantai Biological - IgG IgM IgA - Wantai SARS-CoV-2 Total Ab ELISA,Omega diagnostics - IgG - COVID-19 IgG ELISA | 0.928, 1 | Complex test algorithm: Manual calculation |
| Malawi | Kondwani Jambo (Malawi-Liverpool-Wellcome Trust Clinical Research programme, Liverpool School of Tropical Medicine)[7] | 2020-08-01 - 2020-08-31 | National | Low | 192 | Multiple groups | All | Probability | Blood donors | 9.9% | Beijing Wantai Biological - IgG IgM IgA - Wantai SARS-CoV-2 Total Ab ELISA,Omega diagnostics - IgG - COVID-19 IgG ELISA | 0.928, 1 | Complex test algorithm: Manual calculation |
| Malawi | Kondwani Jambo (Malawi-Liverpool-Wellcome Trust Clinical Research programme, Liverpool School of Tropical Medicine)[7] | 2020-07-01 - 2020-07-31 | National | Low | 204 | Multiple groups | All | Probability | Blood donors | 8.2% | Beijing Wantai Biological - IgG IgM IgA - Wantai SARS-CoV-2 Total Ab ELISA,Omega diagnostics - IgG - COVID-19 IgG ELISA | 0.928, 1 | Complex test algorithm: Manual calculation |
| Malawi | Kondwani Jambo (Malawi-Liverpool-Wellcome Trust Clinical Research programme, Liverpool School of Tropical Medicine)[7] | 2020-06-01 - 2020-06-30 | National | Low | 242 | Multiple groups | All | Probability | Blood donors | 1.4% | Beijing Wantai Biological - IgG IgM IgA - Wantai SARS-CoV-2 Total Ab ELISA,Omega diagnostics - IgG - COVID-19 IgG ELISA | 0.928, 1 | Complex test algorithm: Manual calculation |
| Malawi | Kondwani Jambo (Malawi-Liverpool-Wellcome Trust Clinical Research programme, Liverpool School of Tropical Medicine)[7] | 2020-05-01 - 2020-05-31 | National | Low | 194 | Multiple groups | All | Probability | Blood donors | 1.1% | Beijing Wantai Biological - IgG IgM IgA - Wantai SARS-CoV-2 Total Ab ELISA,Omega diagnostics - IgG - COVID-19 IgG ELISA | 0.928, 1 | Complex test algorithm: Manual calculation |
| Malawi | Kondwani Jambo (Malawi-Liverpool-Wellcome Trust Clinical Research programme, Liverpool School of Tropical Medicine)[7] | 2020-04-01 - 2020-04-30 | National | Low | 205 | Multiple groups | All | Probability | Blood donors | 0.5% | Beijing Wantai Biological - IgG IgM IgA - Wantai SARS-CoV-2 Total Ab ELISA,Omega diagnostics - IgG - COVID-19 IgG ELISA | 0.928, 1 | Complex test algorithm: Manual calculation |
| Malawi | Kondwani Jambo (Malawi-Liverpool-Wellcome Trust Clinical Research programme, Liverpool School of Tropical Medicine)[7] | 2020-01-01 - 2020-01-31 | National | Low | 227 | Multiple groups | All | Probability | Blood donors | 0% | Beijing Wantai Biological - IgG IgM IgA - Wantai SARS-CoV-2 Total Ab ELISA,Omega diagnostics - IgG - COVID-19 IgG ELISA | 0.928, 1 | Complex test algorithm: Manual calculation |
| Malawi | Kondwani Jambo (Malawi-Liverpool-Wellcome Trust Clinical Research programme, Liverpool School of Tropical Medicine)[7] | 2020-02-01 - 2020-02-29 | National | Low | 194 | Multiple groups | All | Probability | Blood donors | 0.3% | Beijing Wantai Biological - IgG IgM IgA - Wantai SARS-CoV-2 Total Ab ELISA,Omega diagnostics - IgG - COVID-19 IgG ELISA | 0.928, 1 | Complex test algorithm: Manual calculation |
| Malawi | Kondwani Jambo (Malawi-Liverpool-Wellcome Trust Clinical Research programme, Liverpool School of Tropical Medicine)[7] | 2020-03-01 - 2020-03-31 | National | Low | 199 | Multiple groups | All | Probability | Blood donors | 0.5% | Beijing Wantai Biological - IgG IgM IgA - Wantai SARS-CoV-2 Total Ab ELISA,Omega diagnostics - IgG - COVID-19 IgG ELISA | 0.928, 1 | Complex test algorithm: Manual calculation |
| Nigeria - HRP | Olatunji Kolawole (Ministerial Expert Advisory Committee on COVID-19- Health Sector Response)[9] | 2021-06-29 - 2021-08-31 | National | Moderate | 4,904 | Multiple groups | All | Probability | Residual sera | 78.9% (95% CI 77.7-80%) | Beijing Wantai Biological - IgG IgM IgA - Wantai SARS-CoV-2 Total Ab ELISA | 0.967, 0.975 | Validated by manufacturer |
| Senegal | Cheikh Talla (Institut Pasteur de Dakar)[10] | 2020-10-25 - 2020-11-26 | National | Low | 1,422 | Multiple groups | All | Probability | Household and community samples | 27.9% | Omega diagnostics - IgG - COVID-19 IgG ELISA,ID.Vet - IgG - ID Screen,Beijing Wantai Biological - IgG, IgM, IgA - Wantai SARS-CoV-2 Total Ab ELISA | N/A | Complex test algorithm: Manual review |
| Sierra Leone - HRP | Mohamed Bailor Barrie (Harvard Medical School)[11] | 2021-03-01 - 2021-03-31 | National | Low | 1,893 | Multiple groups | All | Probability | Household and community samples | 2.8% | Hangzhou Biotest Biotech Co. Ltd - IgG, IgM - RightSign COVID-19 IgG/IgM Rapid Test Cassette | 0.914, 1 | Validated by manufacturer |
| South Africa | Nicole Wolter (National Health Laboratory Service)[12] | 2021-04-01 - 2021-04-30 | National | Moderate | 1,849 | Multiple groups | All | Probability | Multiple general populations | 47.2% | Beijing Wantai Biological - IgG, IgM, IgA - Wantai SARS-CoV-2 Total Ab ELISA,Roche Diagnostics - IgG, IgM, IgA - Elecsys  Anti‐SARS‐CoV‐2 (N) | 1, 0.97 | Complex test algorithm: Manual calculation |
| South Africa | Marion Vermeulen (South African National Blood Service)[13] | 2021-01-15 - 2021-05-15 | National | Moderate | 16,762 | Multiple groups | All | Sequential | Blood donors | 47.4% | Roche Diagnostics - IgG, IgM, IgA - Elecsys  Anti‐SARS‐CoV‐2 (N) | 1, 0.995 | Validated by manufacturer |
| Togo - HRP | Didier K Ekouevi (Université de Lomé)[14] | 2021-05-05 - 2021-06-30 | National | Low | 7,593 | Multiple groups | All | Probability | Household and community samples | 61.8% | Beijing Wantai Biological - IgG IgM IgA - Wantai SARS-CoV-2 Total Ab ELISA | 0.967, 0.975 | Validated by manufacturer |
| Uganda - HRP | Dr. Henry Kyobe Bosa (Ministry of Health/ UPDF)[15] | 2021-03-14 - 2021-03-28 | National | Moderate | 7,029 | Multiple groups | All | Probability | Household and community samples | 15.6% (95% CI 14.7-16.4%) | Hangzhou Biotest Biotech Co. Ltd - IgG IgM - RightSign COVID-19 IgG/IgM Rapid Test Cassette | 0.914, 1 | Validated by manufacturer |
| Zambia - HRP | Jonas Z Hines (US Centers for Disease Control and Prevention)[16] | 2020-07-02 - 2020-07-31 | National | Moderate | 1,657 | Multiple groups | All | Convenience | Residual sera | 5.3% | EUROIMMUN - IgG - Anti-SARS-CoV-2 ELISA IgG | 0.9, 1 | Validated by manufacturer |
| Zambia - HRP | Lloyd B Mulenga (Zambia Ministry of Health)[17] | 2020-07-04 - 2020-07-27 | National | Low | 2,704 | Multiple groups | All | Probability | Household and community samples | 2.1% | EUROIMMUN - IgG - Anti-SARS-CoV-2 ELISA IgG | 0.9, 1 | Validated by manufacturer |

####

#### Americas region

| Country (Location) | Author (Organization) | Sampling Dates (YMD) | Geographic scope | Overall risk of bias | Sample size | Age | Sex | Sampling method | Sample frame | Seroprevalence (95% CI) | Test Manufacturers - Isotypes - Names | Test Sens, Spec | Sens, Spec Source |
| --- | --- | --- | --- | --- | --- | --- | --- | --- | --- | --- | --- | --- | --- |
| Brazil - HRP | Fernando C Barros (Universidade Federal de Pelotas)[18] | 2020-05-14 - 2020-06-23 | National | Moderate | 89,362 | Multiple groups | All | Probability | Household and community samples | 2.3% | Guangzhou Wondfo Biotech Co. Ltd - IgG, IgM, IgA - Wondfo SARS-CoV-2 Antibody Test | 0.864, 0.996 | Validated by manufacturer |
| Colombia - HRP | Marcela Mercado-Reye (Instituto Nacional de Salud)[19] | 2020-09-21 - 2020-12-11 | National | Moderate | 17,863 | Multiple groups | All | Probability | Household and community samples | 32.5% | Siemens - IgG, IgM, IgA - ADVIA Centaur Immunoassay System | 0.86, 0.99 | Validated by independent authors/third party/non-developers |
| Dominican Republic - HRP | Robert Paulino Ramirez (Universidad Iberoamericana)[20] | 2020-04-15 - 2020-06-15 | National | Moderate | 12,897 | Multiple groups | All | Probability | Household and community samples | 5.5% | GenBody Inc. - IgG, IgM - GenBody COVID-19 IgM/IgG | 0.893, 0.959 | Validated by manufacturer |
| Honduras | Mario Rene Mejia Nunez (Colegio Médico)[21] | 2020-06-16 - 2020-06-23 | National | Moderate | 792 | Multiple groups | All | Probability | Household and community samples | 6.2% | NA - - Not reported/ Unable to specify | 0.934, 0.977 | Validated by manufacturers |
| Mexico - HRP | Miguel Fernández-Rojas (Salud Digna A.C.)[22] | 2020-07-05 - 2020-12-31 | National | Moderate | 522,690 | Multiple groups | All | Sequential | Residual sera | 32.8% | Roche Diagnostics - IgG, IgM, IgA - Elecsys  Anti‐SARS‐CoV‐2 (Type Unknown) | 0.995, 0.998 | Validated by manufacturers |
| Mexico - HRP | Francisco Canto-Osorio (Instituto Nacional de Salud Públic)[23] | 2020-08-15 - 2020-11-15 | National | Low | 944 | Children and Youth (0-17 years) | All | Probability | Household and community samples | 18.7% | Roche Diagnostics - IgG, IgM, IgA - Elecsys  Anti‐SARS‐CoV‐2 (N) | 1, 0.995 | Validated by manufacturer |
| Mexico - HRP | Francisco Canto-Osorio (Instituto Nacional de Salud Públic)[23] | 2020-08-15 - 2020-11-15 | National | Low | 858 | Children and Youth (0-17 years) | All | Probability | Household and community samples | 26.7% | Roche Diagnostics - IgG, IgM, IgA - Elecsys  Anti‐SARS‐CoV‐2 (N) | 1, 0.995 | Validated by manufacturer |
| Mexico - HRP | Ana Basto-Abreu (National Institute of Public Health)[24] | 2020-08-15 - 2020-11-15 | National | Moderate | 9,463 | Multiple groups | All | Probability | Household and community samples | 24.9% | Roche Diagnostics - IgG, IgM, IgA - Elecsys  Anti‐SARS‐CoV‐2 (N) | 1, 0.995 | Validated by manufacturer |
| Canada | Canadian Blood Service (Canadian Blood Services )[25] | 2022-04-08 - 2022-04-15 | National | Moderate | 7,908 | Multiple groups | All | Sequential | Blood donors | 35.3% | Roche Diagnostics - IgG, IgM, IgA - Elecsys  Anti‐SARS‐CoV‐2 (N) | 1, 0.995 | Validated by manufacturer |
| Canada | Canadian Blood Services (Canadian Blood Services )[26] | 2022-01-01 - 2022-01-31 | National | Moderate | 32,505 | Multiple groups | All | Sequential | Blood donors | 97.7% | Roche Diagnostics - IgG, IgM, IgA - Elecsys  Anti‐SARS‐CoV‐2 (S) | 0.988, 1 | Validated by manufacturer |
| Canada | Canadian Blood Services (Canadian Blood Services )[27] | 2021-12-14 - 2021-12-30 | National | Moderate | 16,816 | Multiple groups | All | Sequential | Blood donors | 97.3% | Roche Diagnostics - IgG, IgM, IgA - Elecsys  Anti‐SARS‐CoV‐2 (S) | 0.988, 1 | Validated by manufacturer |
| Canada | Canadian Blood Services (Canadian Blood Services )[28] | 2021-11-13 - 2021-11-24 | National | Low | 9,018 | Multiple groups | All | Probability | Blood donors | 97.3% | Roche Diagnostics - IgG, IgM, IgA - Elecsys  Anti‐SARS‐CoV‐2 (S) | 0.988, 1 | Validated by manufacturer |
| Canada | Canadian Blood Services (Canadian Blood Services )[29] | 2021-10-14 - 2021-10-23 | National | Low | 9,627 | Multiple groups | All | Probability | Blood donors | 96.6% | Roche Diagnostics - IgG, IgM, IgA - Elecsys  Anti‐SARS‐CoV‐2 (S) | 0.988, 1 | Validated by manufacturer |
| Canada | Canadian Blood Services (Canadian Blood Services )[30] | 2021-09-14 - 2021-09-24 | National | Low | 9,363 | Multiple groups | All | Probability | Blood donors | 95.9% | Roche Diagnostics - IgG, IgM, IgA - Elecsys  Anti‐SARS‐CoV‐2 (S) | 0.988, 1 | Validated by manufacturer |
| Canada | Canadian Blood Services (Canadian Blood Services )[31] | 2021-08-15 - 2021-08-26 | National | Low | 9,109 | Multiple groups | All | Probability | Blood donors | 95% | Roche Diagnostics - IgG, IgM, IgA - Elecsys  Anti‐SARS‐CoV‐2 (S) | 0.988, 1 | Validated by manufacturer |
| Canada | Canadian Blood Services (Canadian Blood Services )[32] | 2021-07-14 - 2021-07-23 | National | Low | 8,457 | Multiple groups | All | Probability | Blood donors | 93.6% | Roche Diagnostics - IgG, IgM, IgA - Elecsys  Anti‐SARS‐CoV‐2 (S) | 0.988, 1 | Validated by manufacturer |
| Canada | Canadian Blood Services (Canadian Blood Services)[33] | 2021-06-14 - 2021-06-29 | National | Moderate | 16,884 | Multiple groups | All | Sequential | Blood donors | 89.7% | Roche Diagnostics - IgG, IgM, IgA - Elecsys  Anti‐SARS‐CoV‐2 (S) | 0.988, 1 | Validated by manufacturer |
| Canada | Canadian Blood Services (Canadian Blood Services)[34] | 2021-05-08 - 2021-06-04 | National | Moderate | 17,001 | Multiple groups | All | Sequential | Blood donors | 63.3% | Roche Diagnostics - IgG, IgM, IgA - Elecsys  Anti‐SARS‐CoV‐2 (S) | 0.988, 1 | Validated by manufacturer |
| Canada | Canadian Blood Services (Canadian Blood Services)[35] | 2021-04-13 - 2021-04-30 | National | Moderate | 16,931 | Multiple groups | All | Sequential | Blood donors | 26.9% | Roche Diagnostics - IgG, IgM, IgA - Elecsys  Anti‐SARS‐CoV‐2 (S) | 0.988, 1 | Validated by manufacturer |
| Canada | Canadian Blood Services (Canadian Blood Services)[36] | 2021-02-27 - 2021-03-13 | National | Moderate | 16,873 | Multiple groups | All | Sequential | Blood donors | 10.1% | Roche Diagnostics - IgG, IgM, IgA - Elecsys  Anti‐SARS‐CoV‐2 (S) | 0.988, 1 | Validated by manufacturer |
| Canada | Canadian Blood Services (Canadian Blood Services)[37] | 2021-01-01 - 2021-01-27 | National | Moderate | 33,400 | Multiple groups | All | Sequential | Blood donors | 3.2% | Roche Diagnostics - IgG, IgM, IgA - Elecsys  Anti‐SARS‐CoV‐2 (S) | 0.988, 1 | Validated by manufacturer |
| Canada | Canadian Blood Services (Canadian Blood Services)[38] | 2020-12-10 - 2020-12-23 | National | Moderate | 16,961 | Multiple groups | All | Sequential | Blood donors | 1.4% | Abbott Laboratories - IgG - Abbott Architect SARS-CoV-2 IgG | 0.99, 1 | Validated by manufacturer |
| Canada | Canadian Blood Services (Canadian Blood Services)[38] | 2020-11-07 - 2020-11-25 | National | Moderate | 17,049 | Multiple groups | All | Sequential | Blood donors | 1.5% | Abbott Laboratories - IgG - Abbott Architect SARS-CoV-2 IgG | 0.99, 1 | Validated by manufacturer |
| Canada | Canadian Blood Services (Canadian Blood Services)[38] | 2020-10-12 - 2020-10-31 | National | Moderate | 16,811 | Multiple groups | All | Sequential | Blood donors | 0.9% | Abbott Laboratories - IgG - Abbott Architect SARS-CoV-2 IgG | 0.99, 1 | Validated by manufacturer |
| Canada | Sahar Saeed (Canadian Blood Services)[39] | 2020-05-09 - 2020-07-21 | National | Moderate | 74,642 | Multiple groups | All | Probability | Blood donors | 0.7% | Abbott Laboratories - IgG - Abbott Architect SARS-CoV-2 IgG | 0.99, 1 | Validated by manufacturer |
| Chile - HRP | Pablo Vial (Universidad del Desarrollo)[40] | 2020-09-25 - 2020-11-25 | National | Low | 2,493 | Multiple groups | All | Probability | Household and community samples | 10.4% | Roche Diagnostics - IgG, IgM, IgA - Elecsys  Anti‐SARS‐CoV‐2 (S),Zhuhai Livzon Diagnostics Inc - IgG, IgM - 2019-nCoV IgG/IgM Antibody Detection Kit | 0.999, 0.992 | Complex test algorithm: Manual calculation |
| United States of America | Roger Dodd (American Red Cross)[41] | 2021-06-18 - 2021-06-25 | National | Moderate | 45,966 | Multiple groups | All | Convenience | Blood donors | 90.1% | Ortho Clinical Diagnostics Inc. - IgG, IgM, IgA - VITROS Immunodiagnostic Products Anti-SARS-CoV-2 Total | 1, 1 | Validated by manufacturer |
| United States of America | Jefferson Jones (Vitalant Research Institute)[42] | 2021-05-01 - 2021-05-31 | National | Moderate | 134,949 | Multiple groups | All | Probability | Blood donors | 83.3% | Ortho Clinical Diagnostics Inc. - IgG, IgM, IgA - VITROS Immunodiagnostic Products Anti-SARS-CoV-2 Total,Roche Diagnostics - IgG, IgM, IgA - Elecsys  Anti‐SARS‐CoV‐2 (N) | 1, 0.995 | Complex test algorithm: Manual calculation |
| United States of America | Jefferson Jones (Vitalant Research Institute)[42] | 2021-04-01 - 2021-04-30 | National | Moderate | 134,510 | Multiple groups | All | Probability | Blood donors | 71.4% | Ortho Clinical Diagnostics Inc. - IgG, IgM, IgA - VITROS Immunodiagnostic Products Anti-SARS-CoV-2 Total,Roche Diagnostics - IgG, IgM, IgA - Elecsys  Anti‐SARS‐CoV‐2 (N) | 1, 0.995 | Complex test algorithm: Manual calculation |
| United States of America | Jefferson Jones (Vitalant Research Institute)[42] | 2021-03-01 - 2021-03-31 | National | Moderate | 134,557 | Multiple groups | All | Probability | Blood donors | 49.2% | Ortho Clinical Diagnostics Inc. - IgG, IgM, IgA - VITROS Immunodiagnostic Products Anti-SARS-CoV-2 Total,Roche Diagnostics - IgG, IgM, IgA - Elecsys  Anti‐SARS‐CoV‐2 (N) | 1, 0.995 | Complex test algorithm: Manual calculation |
| United States of America | Jefferson Jones (Vitalant Research Institute)[42] | 2021-02-01 - 2021-02-28 | National | Moderate | 133,288 | Multiple groups | All | Probability | Blood donors | 33.5% | Ortho Clinical Diagnostics Inc. - IgG, IgM, IgA - VITROS Immunodiagnostic Products Anti-SARS-CoV-2 Total,Roche Diagnostics - IgG, IgM, IgA - Elecsys  Anti‐SARS‐CoV‐2 (N) | 1, 0.995 | Complex test algorithm: Manual calculation |
| United States of America | Jefferson Jones (Vitalant Research Institute)[42] | 2021-01-01 - 2021-01-31 | National | Moderate | 134,632 | Multiple groups | All | Probability | Blood donors | 20.5% | Ortho Clinical Diagnostics Inc. - IgG, IgM, IgA - VITROS Immunodiagnostic Products Anti-SARS-CoV-2 Total,Roche Diagnostics - IgG, IgM, IgA - Elecsys  Anti‐SARS‐CoV‐2 (N) | 1, 0.995 | Complex test algorithm: Manual calculation |
| United States of America | Patrick Sullivan (Emory University)[43] | 2020-11-01 - 2020-12-08 | National | Low | 2,241 | Multiple groups | All | Probability | Household and community samples | 7.2% | Bio-rad - IgG, IgM, IgA - Platelia SARS-CoV-2 Total Ab assay | 0.993, 0.98 | Validated by manufacturer |
| United States of America | Shuchi Anand (Stanford University)[44] | 2020-07-01 - 2020-07-31 | National | Low | 28,503 | Multiple groups | All | Probability | Residual sera | 9.3% | Siemens - IgG, IgM, IgA - ADVIA Centaur Immunoassay System | 1, 0.998 | Validated by manufacturers |

####

#### Eastern Mediterranean region

| Country (Location) | Author (Organization) | Sampling Dates (YMD) | Geographic scope | Overall risk of bias | Sample size | Age | Sex | Sampling method | Sample frame | Seroprevalence (95% CI) | Test Manufacturers - Isotypes - Names | Test Sens, Spec | Sens, Spec Source |
| --- | --- | --- | --- | --- | --- | --- | --- | --- | --- | --- | --- | --- | --- |
| Iran (Islamic Republic of) - HRP | Kazem Khalagi (Tehran University of Medical Sciences)[45] | 2020-08-03 - 2020-10-31 | National | Low | 11,256 | Multiple groups | All | Probability | Household and community samples | 11.8% | Pishtaz Diagnostics Iran - IgG, IgM - ELISA kit Pishtaz Teb Diagnostics | 0.941, 0.983 | Validated by manufacturer |
| Iran (Islamic Republic of) - HRP | Hossein Poustchi (Tehran University of Medical Sciences)[46] | 2020-04-17 - 2020-06-02 | National | Low | 3,530 | Multiple groups | All | Probability | Household and community samples | 12.9% | Pishtaz Diagnostics Iran - IgG, IgM - ELISA kit Pishtaz Teb Diagnostics | 0.941, 0.983 | Validated by manufacturer |
| Jordan - HRP | Saverio Bellizzi (World Health Organization)[47] | 2020-12-27 - 2021-01-06 | National | Moderate | 5,044 | Multiple groups | All | Probability | Household and community samples | 34.2% | Beijing Wantai Biological - IgM - Wantai SARS-CoV-2 IgM ELISA | 0.868, 1 | Validated by manufacturer |
| Jordan - HRP | Saverio Bellizzi (World Health Organization)[47] | 2020-10-01 - 2020-10-31 | National | Moderate | 5,470 | Multiple groups | All | Probability | Household and community samples | 7% | Beijing Wantai Biological - IgM - Wantai SARS-CoV-2 IgM ELISA | 0.868, 1 | Validated by manufacturer |
| Jordan - HRP | Saverio Bellizzi (World Health Organization)[47] | 2020-08-01 - 2020-08-31 | National | Moderate | 4,704 | Multiple groups | All | Probability | Household and community samples | 0.3% | Beijing Wantai Biological - IgM - Wantai SARS-CoV-2 IgM ELISA | 0.868, 1 | Validated by manufacturer |
| Lebanon - HRP | Dr Alissar Rady (WHO)[@] | 2021-02-15 - 2021-03-22 | National | Low | 1,252 | Multiple groups | All | Probability | Household and community samples | 39.2% | Beijing Wantai Biological - IgG IgM IgA - Wantai SARS-CoV-2 Total Ab ELISA | 0.967, 0.975 | Validated by manufacturer |
| Lebanon - HRP | Dr Alissar Rady (WHO)[@] | 2021-02-15 - 2021-03-22 | National | Moderate | 755 | Multiple groups | All | Probability | Household and community samples | 46.7% | Beijing Wantai Biological - IgG IgM IgA - Wantai SARS-CoV-2 Total Ab ELISA | 0.967, 0.975 | Validated by manufacturer |
| Lebanon - HRP | Abbas Hoballah (Lebanese University)[48] | 2020-12-07 - 2021-01-15 | National | Low | 2,058 | Multiple groups | All | Probability | Household and community samples | 15.9% | Technogenetics - IgG, IgM - nCOVID-19 IgG & IgM POCT | 0.946, 0.993 | Validated by manufacturer |
| occupied Palestinian territory, including east Jerusalem - HRP | Sharif E. Qaddomi (PNIPH)[@] | 2020-12-01 - 2020-12-31 | National | Moderate | 6,154 | Multiple groups | All | Probability | Household and community samples | 39.7% (95% CI 38.5-40.9%) | Roche Diagnostics - IgG IgM IgA - Elecsys  Anti‐SARS‐CoV‐2 (S) | 0.988, 1 | Validated by manufacturer |
| Oman | Seif Al-Abri (Ministry of Health (Oman))[49] | 2020-11-08 - 2020-11-19 | National | Moderate | 4,064 | Multiple groups | All | Probability | Household and community samples | 22% | DiaSorin - IgG - Liaison SARS-CoV-2 S1/S2 IgG | 0.974, 0.985 | Validated by manufacturer |
| Oman | Seif Al-Abri (Ministry of Health (Oman))[49] | 2020-09-13 - 2020-09-24 | National | Moderate | 4,780 | Multiple groups | All | Probability | Household and community samples | 16.4% | DiaSorin - IgG - Liaison SARS-CoV-2 S1/S2 IgG | 0.974, 0.985 | Validated by manufacturer |
| Oman | Seif Al-Abri (Ministry of Health (Oman))[49] | 2020-08-16 - 2020-08-27 | National | Moderate | 4,403 | Multiple groups | All | Probability | Household and community samples | 9.7% | DiaSorin - IgG - Liaison SARS-CoV-2 S1/S2 IgG | 0.974, 0.985 | Validated by manufacturer |
| Oman | Seif Al-Abri (Ministry of Health (Oman))[49] | 2020-07-12 - 2020-07-23 | National | Moderate | 4,210 | Multiple groups | All | Probability | Household and community samples | 5.5% | DiaSorin - IgG - Liaison SARS-CoV-2 S1/S2 IgG | 0.974, 0.985 | Validated by manufacturer |
| Pakistan - HRP | Ahsan Ahmad (Ministry of National Health Services Regulations and Coordination)[50] | 2020-10-21 - 2020-11-08 | National | Moderate | 4,998 | Multiple groups | All | Probability | Household and community samples | 7% | Bioperfectus - IgG, IgM - Novel Corona Virus (SARS-CoV-2) IgM/IgG Rapid Test Kit | 0.9, 0.965 | Validated by manufacturer |
| Pakistan - HRP | Mohsina Haq (Riphah International University )[51] | 2020-07-15 - 2020-07-31 | National | Moderate | 15,390 | Multiple groups | All | Probability | Household and community samples | 42.4% | Roche Diagnostics - IgG, IgM, IgA - Elecsys  Anti‐SARS‐CoV‐2 (N) | 1, 0.995 | Validated by manufacturer |
| Qatar | Peter Coyle (Cornell University)[52] | 2020-10-10 - 2020-10-21 | National | Moderate | 709 | Multiple groups | All | Convenience | Residual sera | 71.9% | Roche Diagnostics - IgG, IgM, IgA - Elecsys  Anti‐SARS‐CoV‐2 (N) | 1, 0.995 | Validated by manufacturer |
| Qatar | Peter Coyle (Cornell University)[53] | 2020-05-12 - 2020-09-09 | National | Moderate | 112,941 | Multiple groups | All | Sequential | Residual sera | 13.3% | Roche Diagnostics - IgG, IgM, IgA - Elecsys  Anti‐SARS‐CoV‐2 (N) | 1, 0.995 | Validated by manufacturer |

#### Europe region

| Country (Location) | Author (Organization) | Sampling Dates (YMD) | Geographic scope | Overall risk of bias | Sample size | Age | Sex | Sampling method | Sample frame | Seroprevalence (95% CI) | Test Manufacturers - Isotypes - Names | Test Sens, Spec | Sens, Spec Source |
| --- | --- | --- | --- | --- | --- | --- | --- | --- | --- | --- | --- | --- | --- |
| Kazakhstan | Manar Smangul (Ministry of Health of the Republic of Kazakhstan)[54] | 2020-10-24 - 2021-01-11 | National | Moderate | 6,891 | Multiple groups | All | Probability | Household and community samples | 57% (95% CI 55.8-58.2%) | Beijing Wantai Biological - IgG IgM IgA - Wantai SARS-CoV-2 Total Ab ELISA | 0.967, 0.975 | Validated by manufacturer |
| Kyrgyzstan | Anna Popova (Saint Petersburg Pasteur Institute)[55] | 2021-06-28 - 2021-07-03 | National | Moderate | 9,471 | Multiple groups | All | Probability | Household and community samples | 48.7% | NA - - Author designed (ELISA) - Nucleocapsid | NA, NA | Validated by independent authors/third party/non-developers |
| Kyrgyzstan | Nurmatov Zuridin, Kuchuk Tatyana (Scientific Production Association “Preventive medicine”)[56] | 2020-07-04 - 2020-08-12 | National | Moderate | 4,540 | Multiple groups | All | Convenience | Household and community samples | 32.5% (95% CI 31.1-33.9%) | Beijing Wantai Biological - IgG IgM IgA - Wantai SARS-CoV-2 Total Ab ELISA | 0.967, 0.975 | Validated by manufacturer |
| Republic of Moldova | Alexei Ceban Stela Gheorghita, WHO CO (National Agency for Public Health)[57] | 2020-11-17 - 2021-01-15 | National | Moderate | 5,656 | Multiple groups | All | Probability | Residual sera | 48.4% (95% CI 47.1-49.7%) | Beijing Wantai Biological - IgG IgM IgA - Wantai SARS-CoV-2 Total Ab ELISA | 0.967, 0.975 | Validated by manufacturer |
| Uzbekistan | R A Rakhimov (Research Institute of Virology)[58] | 2020-08-27 - 2020-09-11 | National | Moderate | 86,879 | Multiple groups | All | Convenience | Residual sera | 23.1% | Beijing Wantai Biological - IgG, IgM, IgA - Wantai Rapid Test for Total Antibody to SARS-CoV-2 | 0.947, 0.989 | Validated by manufacturer |
| Andorra | Cristina Royo Cebrecos (ISGlobal)[59] | 2020-05-04 - 2020-05-28 | National | Moderate | 72,964 | Multiple groups | All | Probability | Household and community samples | 11% | Zhuhai Livzon Diagnostics Inc - IgG, IgM - 2019-nCoV IgG/IgM Antibody Detection Kit | 0.906, 0.992 | Validated by manufacturer |
| Belgium | Sereina Herzog (University of Antwerp)[60] | 2020-10-12 - 2020-10-17 | National | Low | 2,966 | Multiple groups | All | Probability | Residual sera | 4.2% | EUROIMMUN - IgG - Anti-SARS-CoV-2 ELISA IgG | 0.9, 1 | Validated by manufacturer |
| Belgium | Sereina Herzog (University of Antwerp)[60] | 2020-09-07 - 2020-09-12 | National | Low | 3,047 | Multiple groups | All | Probability | Residual sera | 3.3% | EUROIMMUN - IgG - Anti-SARS-CoV-2 ELISA IgG | 0.9, 1 | Validated by manufacturer |
| Belgium | Sereina Herzog (University of Antwerp)[60] | 2020-06-29 - 2020-07-04 | National | Low | 3,023 | Multiple groups | All | Probability | Residual sera | 3.7% | EUROIMMUN - IgG - Anti-SARS-CoV-2 ELISA IgG | 0.9, 1 | Validated by manufacturer |
| Belgium | Sereina Herzog (University of Antwerp)[60] | 2020-06-08 - 2020-06-13 | National | Low | 2,960 | Multiple groups | All | Probability | Residual sera | 4.7% | EUROIMMUN - IgG - Anti-SARS-CoV-2 ELISA IgG | 0.9, 1 | Validated by manufacturer |
| Belgium | Sereina Herzog (University of Antwerp)[60] | 2020-05-18 - 2020-05-25 | National | Low | 3,242 | Multiple groups | All | Probability | Residual sera | 6.2% | EUROIMMUN - IgG - Anti-SARS-CoV-2 ELISA IgG | 0.9, 1 | Validated by manufacturer |
| Belgium | Sereina Herzog (University of Antwerp)[60] | 2020-04-20 - 2020-04-26 | National | Low | 3,397 | Multiple groups | All | Probability | Residual sera | 5.2% | EUROIMMUN - IgG - Anti-SARS-CoV-2 ELISA IgG | 0.9, 1 | Validated by manufacturer |
| Belgium | Sereina Herzog (University of Antwerp)[60] | 2020-03-30 - 2020-04-05 | National | Low | 3,910 | Multiple groups | All | Probability | Residual sera | 1.8% | EUROIMMUN - IgG - Anti-SARS-CoV-2 ELISA IgG | 0.9, 1 | Validated by manufacturer |
| Denmark | Kamille Fogh (Copenhagen University Hospital)[61] | 2021-01-08 - 2021-01-31 | National | Moderate | 22,677 | Multiple groups | All | Probability | Blood donors | 5.8% | Beijing Wantai Biological - IgG, IgM, IgA - Wantai SARS-CoV-2 Total Ab ELISA | 0.967, 0.975 | Validated by manufacturer |
| Denmark | Laura Espenhain (Statens Serum Institut)[62] | 2020-12-01 - 2020-12-31 | National | Low | 4,044 | Multiple groups | All | Probability | Household and community samples | 4.1% | Beijing Wantai Biological - IgG, IgM, IgA - Wantai SARS-CoV-2 Total Ab ELISA | 0.967, 0.975 | Validated by manufacturer |
| Denmark | Laura Espenhain (Statens Serum Institut)[62] | 2020-10-01 - 2020-11-30 | National | Low | 9,654 | Multiple groups | All | Probability | Household and community samples | 2.2% | Beijing Wantai Biological - IgG, IgM, IgA - Wantai SARS-CoV-2 Total Ab ELISA | 0.967, 0.975 | Validated by manufacturer |
| Denmark | Kamille Fogh (Copenhagen University Hospital)[63] | 2020-10-02 - 2020-10-11 | National | Moderate | 318,552 | Multiple groups | All | Probability | Household and community samples | 0.8% | Zhuhai Livzon Diagnostics Inc - IgG, IgM - 2019-nCoV IgG/IgM Antibody Detection Kit | 0.906, 0.992 | Validated by manufacturer |
| Denmark | Laura Espenhain (Statens Serum Institut)[62] | 2020-08-15 - 2020-09-30 | National | Low | 11,478 | Multiple groups | All | Probability | Household and community samples | 2.1% | Beijing Wantai Biological - IgG, IgM, IgA - Wantai SARS-CoV-2 Total Ab ELISA | 0.967, 0.975 | Validated by manufacturer |
| Denmark | Ole Birger Pedersen (Copenhagen University Hospital Rigshospitalet)[64] | 2020-06-02 - 2020-06-19 | National | Low | 1,200 | Seniors (65+ years) | All | Probability | Blood donors | 1.8% | Beijing Wantai Biological - IgG, IgM, IgA - Wantai SARS-CoV-2 Total Ab ELISA | 0.967, 0.975 | Validated by manufacturer |
| Denmark | Laura Espenhain (Statens Serum Institut)[62] | 2020-05-05 - 2020-05-15 | National | Low | 2,512 | Multiple groups | All | Probability | Household and community samples | 1.2% | Beijing Wantai Biological - IgG, IgM, IgA - Wantai SARS-CoV-2 Total Ab ELISA | 0.967, 0.975 | Validated by manufacturer |
| Denmark | Christian Erikstrup (Copenhagen University Hospital)[65] | 2020-04-06 - 2020-05-03 | National | Moderate | 20,640 | Adults (18-64 years) | All | Probability | Blood donors | 2% | Zhuhai Livzon Diagnostics Inc - IgG, IgM - 2019-nCoV IgG/IgM Antibody Detection Kit | 0.906, 0.992 | Validated by manufacturer |
| Finland | Finnish institute for health and welfare (Finnish Institute for Health and Welfare)[66] | 2021-12-19 - 2021-12-25 | National | Moderate | 2 | Adults (18-64 years) | All | Probability | Household and community samples | 50% | NA - IgG, IgM - Author designed (IFA) - MULTIPLEXED | 1, 1 | Validated by developers |
| Finland | Finnish institute for health and welfare (Finnish Institute for Health and Welfare)[66] | 2021-12-12 - 2021-12-18 | National | Moderate | 1 | Adults (18-64 years) | All | Probability | Household and community samples | 100% | NA - IgG, IgM - Author designed (IFA) - MULTIPLEXED | 1, 1 | Validated by developers |
| Finland | Finnish institute for health and welfare (Finnish Institute for Health and Welfare)[66] | 2021-12-05 - 2021-12-11 | National | Moderate | 4 | Adults (18-64 years) | All | Probability | Household and community samples | 100% | NA - IgG, IgM - Author designed (IFA) - MULTIPLEXED | 1, 1 | Validated by developers |
| Finland | Finnish institute for health and welfare (Finnish Institute for Health and Welfare)[66] | 2021-11-28 - 2021-12-04 | National | Moderate | 4 | Adults (18-64 years) | All | Probability | Household and community samples | 75% | NA - IgG, IgM - Author designed (IFA) - MULTIPLEXED | 1, 1 | Validated by developers |
| Finland | Finnish institute for health and welfare (Finnish Institute for Health and Welfare)[66] | 2021-11-21 - 2021-11-27 | National | Moderate | 8 | Adults (18-64 years) | All | Probability | Household and community samples | 87.5% | NA - IgG, IgM - Author designed (IFA) - MULTIPLEXED | 1, 1 | Validated by developers |
| Finland | Finnish institute for health and welfare (Finnish Institute for Health and Welfare)[66] | 2021-11-14 - 2021-11-20 | National | Moderate | 13 | Adults (18-64 years) | All | Probability | Household and community samples | 92.3% | NA - IgG, IgM - Author designed (IFA) - MULTIPLEXED | 1, 1 | Validated by developers |
| Finland | Finnish institute for health and welfare (Finnish Institute for Health and Welfare)[66] | 2021-11-07 - 2021-11-13 | National | Moderate | 30 | Adults (18-64 years) | All | Probability | Household and community samples | 93.3% | NA - IgG, IgM - Author designed (IFA) - MULTIPLEXED | 1, 1 | Validated by developers |
| Finland | Finnish institute for health and welfare (Finnish Institute for Health and Welfare)[66] | 2021-10-31 - 2021-11-06 | National | Moderate | 79 | Adults (18-64 years) | All | Probability | Household and community samples | 91.1% | NA - IgG, IgM - Author designed (IFA) - MULTIPLEXED | 1, 1 | Validated by developers |
| Finland | Finnish institute for health and welfare (Finnish Institute for Health and Welfare)[66] | 2021-10-24 - 2021-10-30 | National | Moderate | 11 | Adults (18-64 years) | All | Probability | Household and community samples | 90.9% | NA - IgG, IgM - Author designed (IFA) - MULTIPLEXED | 1, 1 | Validated by developers |
| Finland | Finnish institute for health and welfare (Finnish Institute for Health and Welfare)[66] | 2021-10-17 - 2021-10-23 | National | Moderate | 1 | Adults (18-64 years) | All | Probability | Household and community samples | 0% | NA - IgG, IgM - Author designed (IFA) - MULTIPLEXED | 1, 1 | Validated by developers |
| Finland | Finnish institute for health and welfare (Finnish Institute for Health and Welfare)[66] | 2021-09-26 - 2021-10-02 | National | Moderate | 2 | Adults (18-64 years) | All | Probability | Household and community samples | 50% | NA - IgG, IgM - Author designed (IFA) - MULTIPLEXED | 1, 1 | Validated by developers |
| Finland | Finnish institute for health and welfare (Finnish Institute for Health and Welfare)[66] | 2021-09-12 - 2021-09-18 | National | Moderate | 1 | Adults (18-64 years) | All | Probability | Household and community samples | 100% | NA - IgG, IgM - Author designed (IFA) - MULTIPLEXED | 1, 1 | Validated by developers |
| Finland | Finnish institute for health and welfare (Finnish Institute for Health and Welfare)[66] | 2021-09-05 - 2021-09-11 | National | Moderate | 1 | Adults (18-64 years) | All | Probability | Household and community samples | 100% | NA - IgG, IgM - Author designed (IFA) - MULTIPLEXED | 1, 1 | Validated by developers |
| Finland | Finnish institute for health and welfare (Finnish Institute for Health and Welfare)[66] | 2021-08-29 - 2021-09-04 | National | Moderate | 1 | Adults (18-64 years) | All | Probability | Household and community samples | 0% | NA - IgG, IgM - Author designed (IFA) - MULTIPLEXED | 1, 1 | Validated by developers |
| Finland | Finnish institute for health and welfare (Finnish Institute for Health and Welfare)[66] | 2021-08-22 - 2021-08-28 | National | Moderate | 8 | Adults (18-64 years) | All | Probability | Household and community samples | 75% | NA - IgG, IgM - Author designed (IFA) - MULTIPLEXED | 1, 1 | Validated by developers |
| Finland | Finnish institute for health and welfare (Finnish Institute for Health and Welfare)[66] | 2021-08-15 - 2021-08-21 | National | Moderate | 7 | Adults (18-64 years) | All | Probability | Household and community samples | 85.7% | NA - IgG, IgM - Author designed (IFA) - MULTIPLEXED | 1, 1 | Validated by developers |
| Finland | Finnish institute for health and welfare (Finnish Institute for Health and Welfare)[66] | 2021-08-08 - 2021-08-14 | National | Moderate | 25 | Adults (18-64 years) | All | Probability | Household and community samples | 84% | NA - IgG, IgM - Author designed (IFA) - MULTIPLEXED | 1, 1 | Validated by developers |
| Finland | Finnish institute for health and welfare (Finnish Institute for Health and Welfare)[66] | 2021-08-01 - 2021-08-07 | National | Moderate | 42 | Adults (18-64 years) | All | Probability | Household and community samples | 92.9% | NA - IgG, IgM - Author designed (IFA) - MULTIPLEXED | 1, 1 | Validated by developers |
| Finland | Finnish institute for health and welfare (Finnish Institute for Health and Welfare)[66] | 2021-07-25 - 2021-07-31 | National | Moderate | 11 | Adults (18-64 years) | All | Probability | Household and community samples | 81.8% | NA - IgG, IgM - Author designed (IFA) - MULTIPLEXED | 1, 1 | Validated by developers |
| Finland | Finnish institute for health and welfare (Finnish Institute for Health and Welfare)[66] | 2021-07-18 - 2021-07-24 | National | Moderate | 2 | Adults (18-64 years) | All | Probability | Household and community samples | 100% | NA - IgG, IgM - Author designed (IFA) - MULTIPLEXED | 1, 1 | Validated by developers |
| Finland | Finnish institute for health and welfare (Finnish Institute for Health and Welfare)[66] | 2021-07-11 - 2021-07-17 | National | Moderate | 2 | Adults (18-64 years) | All | Probability | Household and community samples | 100% | NA - IgG, IgM - Author designed (IFA) - MULTIPLEXED | 1, 1 | Validated by developers |
| Finland | Finnish institute for health and welfare (Finnish Institute for Health and Welfare)[66] | 2021-07-04 - 2021-07-10 | National | Moderate | 12 | Adults (18-64 years) | All | Probability | Household and community samples | 58.3% | NA - IgG, IgM - Author designed (IFA) - MULTIPLEXED | 1, 1 | Validated by developers |
| Finland | Finnish institute for health and welfare (Finnish Institute for Health and Welfare)[66] | 2021-06-27 - 2021-07-03 | National | Moderate | 48 | Adults (18-64 years) | All | Probability | Household and community samples | 68.8% | NA - IgG, IgM - Author designed (IFA) - MULTIPLEXED | 1, 1 | Validated by developers |
| Finland | Finnish institute for health and welfare (Finnish Institute for Health and Welfare)[66] | 2021-06-20 - 2021-06-26 | National | Moderate | 44 | Adults (18-64 years) | All | Probability | Household and community samples | 72.7% | NA - IgG, IgM - Author designed (IFA) - MULTIPLEXED | 1, 1 | Validated by developers |
| Finland | Finnish institute for health and welfare (Finnish Institute for Health and Welfare)[66] | 2021-06-13 - 2021-06-19 | National | Moderate | 47 | Adults (18-64 years) | All | Probability | Household and community samples | 57.5% | NA - IgG, IgM - Author designed (IFA) - MULTIPLEXED | 1, 1 | Validated by developers |
| Finland | Finnish institute for health and welfare (Finnish Institute for Health and Welfare)[66] | 2021-06-06 - 2021-06-12 | National | Moderate | 68 | Adults (18-64 years) | All | Probability | Household and community samples | 60.3% | NA - IgG, IgM - Author designed (IFA) - MULTIPLEXED | 1, 1 | Validated by developers |
| Finland | Finnish institute for health and welfare (Finnish Institute for Health and Welfare)[66] | 2021-05-30 - 2021-06-05 | National | Moderate | 39 | Adults (18-64 years) | All | Probability | Household and community samples | 48.7% | NA - IgG, IgM - Author designed (IFA) - MULTIPLEXED | 1, 1 | Validated by developers |
| Finland | Finnish institute for health and welfare (Finnish Institute for Health and Welfare)[66] | 2021-05-23 - 2021-05-29 | National | Moderate | 93 | Adults (18-64 years) | All | Probability | Household and community samples | 49.5% | NA - IgG, IgM - Author designed (IFA) - MULTIPLEXED | 1, 1 | Validated by developers |
| Finland | Finnish institute for health and welfare (Finnish Institute for Health and Welfare)[66] | 2021-05-16 - 2021-05-22 | National | Moderate | 86 | Adults (18-64 years) | All | Probability | Household and community samples | 44.2% | NA - IgG, IgM - Author designed (IFA) - MULTIPLEXED | 1, 1 | Validated by developers |
| Finland | Finnish institute for health and welfare (Finnish Institute for Health and Welfare)[66] | 2021-05-09 - 2021-05-15 | National | Moderate | 52 | Adults (18-64 years) | All | Probability | Household and community samples | 26.9% | NA - IgG, IgM - Author designed (IFA) - MULTIPLEXED | 1, 1 | Validated by developers |
| Finland | Finnish institute for health and welfare (Finnish Institute for Health and Welfare)[66] | 2021-05-02 - 2021-05-08 | National | Moderate | 74 | Adults (18-64 years) | All | Probability | Household and community samples | 28.4% | NA - IgG, IgM - Author designed (IFA) - MULTIPLEXED | 1, 1 | Validated by developers |
| Finland | Finnish institute for health and welfare (Finnish Institute for Health and Welfare)[66] | 2021-04-25 - 2021-05-01 | National | Moderate | 109 | Adults (18-64 years) | All | Probability | Household and community samples | 22.9% | NA - IgG, IgM - Author designed (IFA) - MULTIPLEXED | 1, 1 | Validated by developers |
| Finland | Finnish institute for health and welfare (Finnish Institute for Health and Welfare)[66] | 2021-04-18 - 2021-04-24 | National | Moderate | 102 | Adults (18-64 years) | All | Probability | Household and community samples | 18.6% | NA - IgG, IgM - Author designed (IFA) - MULTIPLEXED | 1, 1 | Validated by developers |
| Finland | Finnish institute for health and welfare (Finnish Institute for Health and Welfare)[66] | 2021-04-11 - 2021-04-17 | National | Moderate | 29 | Adults (18-64 years) | All | Probability | Household and community samples | 13.8% | NA - IgG, IgM - Author designed (IFA) - MULTIPLEXED | 1, 1 | Validated by developers |
| Finland | Finnish institute for health and welfare (Finnish Institute for Health and Welfare)[66] | 2021-04-04 - 2021-04-10 | National | Moderate | 59 | Adults (18-64 years) | All | Probability | Household and community samples | 6.8% | NA - IgG, IgM - Author designed (IFA) - MULTIPLEXED | 1, 1 | Validated by developers |
| Finland | Finnish institute for health and welfare (Finnish Institute for Health and Welfare)[66] | 2021-03-28 - 2021-04-03 | National | Moderate | 60 | Adults (18-64 years) | All | Probability | Household and community samples | 8.3% | NA - IgG, IgM - Author designed (IFA) - MULTIPLEXED | 1, 1 | Validated by developers |
| Finland | Finnish institute for health and welfare (Finnish Institute for Health and Welfare)[66] | 2021-03-21 - 2021-03-27 | National | Moderate | 73 | Adults (18-64 years) | All | Probability | Household and community samples | 10.6% | NA - IgG, IgM - Author designed (IFA) - MULTIPLEXED | 1, 1 | Validated by developers |
| Finland | Finnish institute for health and welfare (Finnish Institute for Health and Welfare)[66] | 2021-03-14 - 2021-03-20 | National | Moderate | 104 | Adults (18-64 years) | All | Probability | Household and community samples | 6.7% | NA - IgG, IgM - Author designed (IFA) - MULTIPLEXED | 1, 1 | Validated by developers |
| Finland | Finnish institute for health and welfare (Finnish Institute for Health and Welfare)[66] | 2021-03-07 - 2021-03-13 | National | Moderate | 64 | Adults (18-64 years) | All | Probability | Household and community samples | 3.1% | NA - IgG, IgM - Author designed (IFA) - MULTIPLEXED | 1, 1 | Validated by developers |
| Finland | Finnish institute for health and welfare (Finnish Institute for Health and Welfare)[66] | 2021-02-28 - 2021-03-06 | National | Moderate | 132 | Adults (18-64 years) | All | Probability | Household and community samples | 2.3% | NA - IgG, IgM - Author designed (IFA) - MULTIPLEXED | 1, 1 | Validated by developers |
| Finland | Finnish institute for health and welfare (Finnish Institute for Health and Welfare)[66] | 2021-02-21 - 2021-02-27 | National | Moderate | 123 | Adults (18-64 years) | All | Probability | Household and community samples | 6.5% | NA - IgG, IgM - Author designed (IFA) - MULTIPLEXED | 1, 1 | Validated by developers |
| Finland | Finnish institute for health and welfare (Finnish Institute for Health and Welfare)[66] | 2021-02-14 - 2021-02-20 | National | Moderate | 69 | Adults (18-64 years) | All | Probability | Household and community samples | 7.2% | NA - IgG, IgM - Author designed (IFA) - MULTIPLEXED | 1, 1 | Validated by developers |
| Finland | Finnish institute for health and welfare (Finnish Institute for Health and Welfare)[66] | 2021-02-07 - 2021-02-13 | National | Moderate | 111 | Adults (18-64 years) | All | Probability | Household and community samples | 5.4% | NA - IgG, IgM - Author designed (IFA) - MULTIPLEXED | 1, 1 | Validated by developers |
| Finland | Finnish institute for health and welfare (Finnish Institute for Health and Welfare)[66] | 2021-01-31 - 2021-02-06 | National | Moderate | 110 | Adults (18-64 years) | All | Probability | Household and community samples | 2.7% | NA - IgG, IgM - Author designed (IFA) - MULTIPLEXED | 1, 1 | Validated by developers |
| Finland | Finnish institute for health and welfare (Finnish Institute for Health and Welfare)[66] | 2021-01-24 - 2021-01-30 | National | Moderate | 30 | Adults (18-64 years) | All | Probability | Household and community samples | 0% | NA - IgG, IgM - Author designed (IFA) - MULTIPLEXED | 1, 1 | Validated by developers |
| Finland | Finnish institute for health and welfare (Finnish Institute for Health and Welfare)[66] | 2021-01-17 - 2021-01-23 | National | Moderate | 5 | Adults (18-64 years) | All | Probability | Household and community samples | 0% | NA - IgG, IgM - Author designed (IFA) - MULTIPLEXED | 1, 1 | Validated by developers |
| Finland | Finnish institute for health and welfare (Finnish Institute for Health and Welfare)[66] | 2021-01-10 - 2021-01-16 | National | Moderate | 6 | Adults (18-64 years) | All | Probability | Household and community samples | 0% | NA - IgG, IgM - Author designed (IFA) - MULTIPLEXED | 1, 1 | Validated by developers |
| Finland | Finnish institute for health and welfare (Finnish Institute for Health and Welfare)[66] | 2021-01-03 - 2021-01-09 | National | Moderate | 12 | Adults (18-64 years) | All | Probability | Household and community samples | 0% | NA - IgG, IgM - Author designed (IFA) - MULTIPLEXED | 1, 1 | Validated by developers |
| Finland | Finnish institute for health and welfare (Finnish Institute for Health and Welfare)[66] | 2020-12-27 - 2021-01-02 | National | Moderate | 26 | Adults (18-64 years) | All | Probability | Household and community samples | 0% | NA - IgG, IgM - Author designed (IFA) - MULTIPLEXED | 1, 1 | Validated by developers |
| Finland | Finnish institute for health and welfare (Finnish Institute for Health and Welfare)[66] | 2020-12-20 - 2020-12-26 | National | Moderate | 65 | Adults (18-64 years) | All | Probability | Household and community samples | 0% | NA - IgG, IgM - Author designed (IFA) - MULTIPLEXED | 1, 1 | Validated by developers |
| Finland | Finnish institute for health and welfare (Finnish Institute for Health and Welfare)[66] | 2020-12-13 - 2020-12-19 | National | Moderate | 115 | Adults (18-64 years) | All | Probability | Household and community samples | 0.9% | NA - IgG, IgM - Author designed (IFA) - MULTIPLEXED | 1, 1 | Validated by developers |
| Finland | Finnish institute for health and welfare (Finnish Institute for Health and Welfare)[66] | 2020-12-06 - 2020-12-12 | National | Moderate | 106 | Adults (18-64 years) | All | Probability | Household and community samples | 0.9% | NA - IgG, IgM - Author designed (IFA) - MULTIPLEXED | 1, 1 | Validated by developers |
| Finland | Finnish institute for health and welfare (Finnish Institute for Health and Welfare)[66] | 2020-11-29 - 2020-12-05 | National | Moderate | 80 | Adults (18-64 years) | All | Probability | Household and community samples | 2.5% | NA - IgG, IgM - Author designed (IFA) - MULTIPLEXED | 1, 1 | Validated by developers |
| Finland | Finnish institute for health and welfare (Finnish Institute for Health and Welfare)[66] | 2020-11-22 - 2020-11-28 | National | Moderate | 85 | Adults (18-64 years) | All | Probability | Household and community samples | 3.5% | NA - IgG, IgM - Author designed (IFA) - MULTIPLEXED | 1, 1 | Validated by developers |
| Finland | Finnish institute for health and welfare (Finnish Institute for Health and Welfare)[66] | 2020-11-15 - 2020-11-21 | National | Moderate | 64 | Adults (18-64 years) | All | Probability | Household and community samples | 3.1% | NA - IgG, IgM - Author designed (IFA) - MULTIPLEXED | 1, 1 | Validated by developers |
| Finland | Finnish institute for health and welfare (Finnish Institute for Health and Welfare)[66] | 2020-11-08 - 2020-11-14 | National | Moderate | 52 | Adults (18-64 years) | All | Probability | Household and community samples | 1.9% | NA - IgG, IgM - Author designed (IFA) - MULTIPLEXED | 1, 1 | Validated by developers |
| Finland | Finnish institute for health and welfare (Finnish Institute for Health and Welfare)[66] | 2020-11-01 - 2020-11-07 | National | Moderate | 55 | Adults (18-64 years) | All | Probability | Household and community samples | 5.4% | NA - IgG, IgM - Author designed (IFA) - MULTIPLEXED | 1, 1 | Validated by developers |
| Finland | Finnish institute for health and welfare (Finnish Institute for Health and Welfare)[66] | 2020-10-25 - 2020-10-31 | National | Moderate | 56 | Adults (18-64 years) | All | Probability | Household and community samples | 1.8% | NA - IgG, IgM - Author designed (IFA) - MULTIPLEXED | 1, 1 | Validated by developers |
| Finland | Finnish institute for health and welfare (Finnish Institute for Health and Welfare)[66] | 2020-10-18 - 2020-10-24 | National | Moderate | 55 | Adults (18-64 years) | All | Probability | Household and community samples | 0% | NA - IgG, IgM - Author designed (IFA) - MULTIPLEXED | 1, 1 | Validated by developers |
| Finland | Finnish institute for health and welfare (Finnish Institute for Health and Welfare)[66] | 2020-10-11 - 2020-10-17 | National | Moderate | 59 | Adults (18-64 years) | All | Probability | Household and community samples | 1.7% | NA - IgG, IgM - Author designed (IFA) - MULTIPLEXED | 1, 1 | Validated by developers |
| Finland | Finnish institute for health and welfare (Finnish Institute for Health and Welfare)[66] | 2020-10-04 - 2020-10-10 | National | Moderate | 51 | Adults (18-64 years) | All | Probability | Household and community samples | 0% | NA - IgG, IgM - Author designed (IFA) - MULTIPLEXED | 1, 1 | Validated by developers |
| Finland | Finnish institute for health and welfare (Finnish Institute for Health and Welfare)[66] | 2020-09-27 - 2020-10-03 | National | Moderate | 60 | Adults (18-64 years) | All | Probability | Household and community samples | 1.7% | NA - IgG, IgM - Author designed (IFA) - MULTIPLEXED | 1, 1 | Validated by developers |
| Finland | Finnish institute for health and welfare (Finnish Institute for Health and Welfare)[66] | 2020-04-19 - 2020-04-25 | National | Moderate | 674 | Adults (18-64 years) | All | Probability | Household and community samples | 0.3% | NA - - Author designed (Neutralization Assay),NA - IgG, IgM - Author designed (IFA) - MULTIPLEXED | N/A | Complex test algorithm: Manual review |
| France | Josiane Warszawski (University of Paris-Saclay)[67] | 2020-05-02 - 2020-06-02 | National | Moderate | 12,114 | Multiple groups | All | Stratified non-probability | Household and community samples | 6.5% | EUROIMMUN - IgG - Anti-SARS-CoV-2 ELISA IgG | 0.9, 1 | Validated by manufacturer |
| France | Stephane Le Vu (Santé publique France)[68] | 2020-05-11 - 2020-05-17 | National | Low | 3,592 | Multiple groups | All | Probability | Residual sera | 5% | NA - - Author designed (Neutralization Assay),NA - IgG - Author designed (ELISA) -Spike,NA - IgG - Author designed (ELISA) - Nucleocapsid | N/A | Complex test algorithm: Manual review |
| France | Stephane Le Vu (Santé publique France)[68] | 2020-04-06 - 2020-04-12 | National | Low | 3,595 | Multiple groups | All | Probability | Residual sera | 4.3% | NA - - Author designed (Neutralization Assay),NA - IgG - Author designed (ELISA) -Spike,NA - IgG - Author designed (ELISA) - Nucleocapsid | N/A | Complex test algorithm: Manual review |
| France | Stephane Le Vu (Santé publique France)[68] | 2020-03-09 - 2020-03-15 | National | Low | 3,834 | Multiple groups | All | Probability | Residual sera | 0.7% | NA - - Author designed (Neutralization Assay),NA - IgG - Author designed (ELISA) -Spike,NA - IgG - Author designed (ELISA) - Nucleocapsid | N/A | Complex test algorithm: Manual review |
| Germany | Clemens Fuest (ifo Institute)[69] | 2020-10-26 - 2020-11-18 | National | Moderate | 9,929 | Multiple groups | All | Convenience | Household and community samples | 1.1% | Roche Diagnostics - IgG, IgM, IgA - Elecsys  Anti‐SARS‐CoV‐2 (N) | 1, 0.995 | Validated by manufacturer |
| Germany | Daniela Gornyk (Helmholtz Centre for Infection Research)[70] | 2020-07-15 - 2020-12-15 | National | Moderate | 13,405 | Multiple groups | All | Probability | Household and community samples | 2% | EUROIMMUN - IgG - Anti-SARS-CoV-2 ELISA IgG | 0.9, 1 | Validated by manufacturer |
| Greece | Michalis Koureas (University of Thessaly)[71] | 2020-12-01 - 2020-12-31 | National | Moderate | 6,231 | Multiple groups | All | Probability | Residual sera | 7.9% | Abbott Laboratories - IgG - Abbott Architect SARS-CoV-2 IgG | 0.99, 1 | Validated by manufacturer |
| Holy See | Massimo Ralli (Università Cattolica del Sacro Cuore)[72] | 2021-01-01 - 2021-01-04 | National | Moderate | 17 | Multiple groups | All | Convenience | Household and community samples | 5.9% | Siemens - IgG, IgM, IgA - Atellica  IM SARS-CoV-2 Total (COV2T) | 0.985, 0.998 | Validated by manufacturer |
| Hungary | Bela Merkely (Semmelweis University)[73] | 2020-05-01 - 2020-05-16 | National | Low | 10,474 | Multiple groups | All | Probability | Household and community samples | 0.7% | Abbott Laboratories - IgG - Abbott Architect SARS-CoV-2 IgG | 0.99, 1 | Validated by manufacturer |
| Israel | Victoria Indenbaum (Tel Aviv University)[74] | 2020-01-01 - 2021-03-31 | National | Moderate | 2,765 | Children and Youth (0-17 years) | All | Convenience | Residual sera | 5.6% | NA - - Author designed (ELISA) -Spike,NA - - Author designed (Neutralization Assay) | N/A | Complex test algorithm: Manual review |
| Israel | Shay Reicher (Ministry of Health)[75] | 2020-06-28 - 2020-09-14 | National | Moderate | 54,357 | Multiple groups | All | Convenience | Household and community samples | 4.6% | Abbott Laboratories - IgG - Abbott Architect SARS-CoV-2 IgG,DiaSorin - IgG - Liaison SARS-CoV-2 S1/S2 IgG | 0.964, 1 | Complex test algorithm: Manual calculation |
| Italy | Instituto Nazionale di Statistica (Italian Ministry of Health)[76] | 2020-05-25 - 2020-07-15 | National | Moderate | 64,660 | Multiple groups | All | Stratified non-probability | Household and community samples | 2.5% | Abbott Laboratories - IgG - Abbott Architect SARS-CoV-2 IgG | 0.99, 1 | Validated by manufacturer |
| Jersey | Statistics Jersey (Statistics Jersey)[77] | 2020-06-21 - 2020-06-27 | National | Low | 1,386 | Multiple groups | All | Probability | Household and community samples | 4.2% | CTK Biotech, Inc. - IgG, IgM - OnSite COVID-19 IgG/IgM Rapid Test | 0.969, 0.994 | Validated by manufacturer |
| Lithuania | Kastytis Smigelskas ( Lithuanian University of Health Sciences)[78] | 2020-08-10 - 2020-09-10 | National | Moderate | 3,089 | Multiple groups | All | Probability | Household and community samples | 1.9% | AMEDA Labordiagnostik GmbH - IgG, IgM - AMP Rapid Test SARS-CoV-2 IgG/IgM | 1, 0.975 | Validated by manufacturer |
| Luxembourg | Chantal Snoeck (Luxembourg Institute of Health)[79] | 2020-04-15 - 2020-05-05 | National | Low | 1,820 | Multiple groups | All | Probability | Household and community samples | 2.1% | EUROIMMUN - IgG - Anti-SARS-CoV-2 ELISA IgG | 0.9, 1 | Validated by manufacturer |
| Norway | Gro Tunheim (Norwegian Institute of Public Health)[80] | 2020-12-27 - 2021-02-13 | National | Moderate | 1,912 | Multiple groups | All | Convenience | Residual sera | 3.2% | NA - - Author designed (type unknown) | 0.96, 0.998 | Validated by independent authors/third party/non-developers |
| Portugal | ISN COVID-19 Group (National Institute of Health)[81] | 2021-09-28 - 2021-11-19 | National | Moderate | 4,545 | Multiple groups | All | Stratified non-probability | Household and community samples | 86.4% | Abbott Laboratories - IgG - Abbott Architect SARS-CoV-2 IgG,Abbott Laboratories - IgG - SARS-CoV-2 IgG II Quant | 1, 0.996 | Complex test algorithm: Manual calculation |
| Portugal | Luisa Canto e Castro (Los Alamos National Laboratory)[82] | 2021-03-01 - 2021-03-17 | National | Low | 2,435 | Multiple groups | All | Probability | Household and community samples | 17.3% | Siemens - IgG, IgM, IgA - Atellica  IM SARS-CoV-2 Total (COV2T) | 0.985, 0.998 | Validated by manufacturer |
| Portugal | Luisa Castro (Los Alamos National Laboratory)[83] | 2020-09-08 - 2020-10-14 | National | Moderate | 13,398 | Multiple groups | All | Convenience | Household and community samples | 2.2% | Siemens - IgG, IgM, IgA - ADVIA Centaur Immunoassay System | 0.981, 0.999 | Validated by independent authors/third party/non-developers |
| Portugal | Irina Kislaya ( Instituto Nacional de Saúde Doutor Ricardo Jorge)[84] | 2020-05-21 - 2020-07-08 | National | Moderate | 2,301 | Multiple groups | All | Stratified non-probability | Residual sera | 2.9% | EUROIMMUN - IgG - Anti-SARS-CoV-2 ELISA IgG,Beijing Wantai Biological - IgM - Wantai SARS-CoV-2 IgM ELISA | 0.987, 1 | Complex test algorithm: Manual calculation |
| Slovenia | Mario Poljak (University of Ljubljana)[85] | 2020-10-17 - 2020-11-10 | National | Low | 1,211 | Multiple groups | All | Probability | Household and community samples | 4.3% | Roche Diagnostics - IgG, IgM, IgA - Elecsys  Anti‐SARS‐CoV‐2 (N),Roche Diagnostics - IgG, IgM, IgA - Elecsys  Anti‐SARS‐CoV‐2 (S) | 1, 0.995 | Complex test algorithm: Manual calculation |
| Slovenia | Mario Poljak (University of Ljubljana)[85] | 2020-04-20 - 2020-05-01 | National | Low | 1,316 | Multiple groups | All | Probability | Household and community samples | 3.1% | EUROIMMUN - IgG - Anti-SARS-CoV-2 ELISA IgG | 0.9, 1 | Validated by manufacturer |
| Spain | Mayte Perez-Olmeda (Instituto de Salud Carlos III)[86] | 2020-11-16 - 2020-11-29 | National | Moderate | 5,827 | Multiple groups | All | Probability | Household and community samples | 5.4% | Beckman Coulter - IgG - Access SARS-CoV-2 IgG Antibody Test | 1, 0.998 | Validated by manufacturer |
| Spain | Ministry of Health (Instituto de Salud Carlos III)[87] | 2020-06-08 - 2020-06-22 | National | Low | 62,167 | Multiple groups | All | Probability | Household and community samples | 5.2% | Zhejiang Orient Gene Biotech - IgG, IgM - COVID-19 IgG/IgM Rapid Test | 0.972, 1 | Validated by manufacturer |
| Spain | Nicolas Prados (IVIRMA Global Headquarters)[88] | 2020-04-27 - 2020-06-26 | National | Moderate | 6,140 | Adults (18-64 years) | All | Convenience | Residual sera | 0.7% | Epitope Diagnostics, Inc. - IgM - EDI™ Novel Coronavirus COVID-19 IgM ELISA Kit | 1, 1 | Validated by manufacturer |
| Sweden | Swedish Public Health Agency (Swedish Public Health Agency)[89] | 2021-09-20 - 2021-10-03 | National | Moderate | 2,959 | Multiple groups | All | Convenience | Residual sera | 75.6% | Sci LifeLab / KTH - - Not available | 0.989, 0.994 | Validated by independent authors/third party/non-developers |
| Sweden | Swedish Public Health Agency (Swedish Public Health Agency)[90] | 2021-05-24 - 2021-06-04 | National | Moderate | 3,949 | Multiple groups | All | Stratified non-probability | Residual sera | 52.2% | Sci LifeLab / KTH - - Not available | 0.989, 0.994 | Validated by independent authors/third party/non-developers |
| Sweden | Swedish Public Health Agency (The Swedish Public Health Agency (Folkhälsomyndigheten))[91] | 2020-11-23 - 2020-12-04 | National | Moderate | 3,183 | Multiple groups | All | Convenience | Blood donors | 7% | Sci LifeLab / KTH - - Not available | 0.994, 0.989 | Validated by manufacturers |
| The United Kingdom | Public Health Scotland (Public Health Scotland)[92] | 2022-02-28 - 2022-04-03 | National | Moderate | 2,499 | Multiple groups | All | Sequential | Blood donors | 98.6% | EUROIMMUN - IgG - Anti-SARS-CoV-2 ELISA IgG | 0.9, 1 | Validated by manufacturer |
| The United Kingdom | Public Health Scotland (Public Health Scotland)[92] | 2022-02-28 - 2022-04-03 | National | Moderate | 3,181 | Adults (18-64 years) | Female | Sequential | Pregnant or parturient women | 95% | Roche Diagnostics - IgG, IgM, IgA - Elecsys  Anti‐SARS‐CoV‐2 (S) | 0.988, 1 | Validated by manufacturer |
| The United Kingdom | Public Health Scotland (Public Health Scotland)[92] | 2022-02-28 - 2022-04-03 | National | Low | 2,885 | Multiple groups | All | Probability | Residual sera | 91.8% | DiaSorin - IgG - Liaison SARS-CoV-2 S1/S2 IgG | 0.974, 0.985 | Validated by manufacturer |
| The United Kingdom | Public Health Scotland (Public Health Scotland)[93] | 2022-01-24 - 2022-02-27 | National | Moderate | 2,499 | Multiple groups | All | Sequential | Blood donors | 98.6% | EUROIMMUN - IgG - Anti-SARS-CoV-2 ELISA IgG | 0.9, 1 | Validated by manufacturer |
| The United Kingdom | Public Health Scotland (Public Health Scotland)[93] | 2022-01-24 - 2022-02-27 | National | Moderate | 3,217 | Adults (18-64 years) | Female | Sequential | Pregnant or parturient women | 94% | Roche Diagnostics - IgG, IgM, IgA - Elecsys  Anti‐SARS‐CoV‐2 (S) | 0.988, 1 | Validated by manufacturer |
| The United Kingdom | Public Health Scotland (Public Health Scotland)[93] | 2022-01-24 - 2022-02-27 | National | Low | 2,884 | Multiple groups | All | Probability | Residual sera | 90.7% | DiaSorin - IgG - Liaison SARS-CoV-2 S1/S2 IgG | 0.974, 0.985 | Validated by manufacturer |
| The United Kingdom | Public Health Scotland (Public Health Scotland)[94] | 2021-12-20 - 2022-01-23 | National | Moderate | 2,499 | Multiple groups | All | Sequential | Blood donors | 98.7% | EUROIMMUN - IgG - Anti-SARS-CoV-2 ELISA IgG | 0.9, 1 | Validated by manufacturer |
| The United Kingdom | Public Health Scotland (Public Health Scotland)[94] | 2021-12-20 - 2022-01-23 | National | Low | 2,863 | Multiple groups | All | Probability | Residual sera | 88.9% | DiaSorin - IgG - Liaison SARS-CoV-2 S1/S2 IgG | 0.974, 0.985 | Validated by manufacturer |
| The United Kingdom | Public Health Scotland (Public Health Scotland)[95] | 2021-11-15 - 2021-12-19 | National | Moderate | 2,493 | Multiple groups | All | Sequential | Blood donors | 96.3% | EUROIMMUN - IgG - Anti-SARS-CoV-2 ELISA IgG | 0.9, 1 | Validated by manufacturer |
| The United Kingdom | Public Health Scotland (Public Health Scotland)[95] | 2021-11-15 - 2021-12-19 | National | Low | 2,815 | Multiple groups | All | Probability | Residual sera | 85.3% | DiaSorin - IgG - Liaison SARS-CoV-2 S1/S2 IgG | 0.974, 0.985 | Validated by manufacturer |
| The United Kingdom | Public Health Scotland (Public Health Scotland)[96] | 2021-10-11 - 2021-11-14 | National | Moderate | 2,496 | Multiple groups | All | Sequential | Blood donors | 94.4% | EUROIMMUN - IgG - Anti-SARS-CoV-2 ELISA IgG | 0.9, 1 | Validated by manufacturer |
| The United Kingdom | Public Health Scotland (Public Health Scotland)[96] | 2021-10-11 - 2021-11-14 | National | Low | 2,882 | Multiple groups | All | Probability | Residual sera | 78.1% | DiaSorin - IgG - Liaison SARS-CoV-2 S1/S2 IgG | 0.974, 0.985 | Validated by manufacturer |
| The United Kingdom | Clarissa Oeser (UK Health Security Agency (UKHSA, formerly PHE))[97] | 2021-09-01 - 2021-10-31 | National | Moderate | 600 | Children and Youth (0-17 years) | All | Convenience | Residual sera | 54.5% | Roche Diagnostics - IgG, IgM, IgA - Elecsys  Anti‐SARS‐CoV‐2 (S) | 0.988, 1 | Validated by manufacturer |
| The United Kingdom | Public Health Scotland (Public Health Scotland)[98] | 2021-09-06 - 2021-10-10 | National | Moderate | 2,494 | Multiple groups | All | Sequential | Blood donors | 95.3% | EUROIMMUN - IgG - Anti-SARS-CoV-2 ELISA IgG | 0.9, 1 | Validated by manufacturer |
| The United Kingdom | Public Health Scotland (Public Health Scotland)[98] | 2021-09-06 - 2021-10-10 | National | Moderate | 3,023 | Adults (18-64 years) | Female | Sequential | Pregnant or parturient women | 18.1% | Roche Diagnostics - IgG, IgM, IgA - Elecsys  Anti‐SARS‐CoV‐2 (N) | 1, 0.995 | Validated by manufacturer |
| The United Kingdom | Public Health Scotland (Public Health Scotland)[98] | 2021-09-06 - 2021-10-10 | National | Low | 3,132 | Multiple groups | All | Probability | Residual sera | 74.3% | DiaSorin - IgG - Liaison SARS-CoV-2 S1/S2 IgG | 0.974, 0.985 | Validated by manufacturer |
| The United Kingdom | Clarissa Oeser (UK Health Security Agency (UKHSA, formerly PHE))[97] | 2021-07-01 - 2021-08-31 | National | Moderate | 685 | Children and Youth (0-17 years) | All | Convenience | Residual sera | 29.5% | Roche Diagnostics - IgG, IgM, IgA - Elecsys  Anti‐SARS‐CoV‐2 (S) | 0.988, 1 | Validated by manufacturer |
| The United Kingdom | Public Health Scotland (Public Health Scotland)[99] | 2021-06-28 - 2021-08-01 | National | Moderate | 2,493 | Multiple groups | All | Sequential | Blood donors | 94.2% | EUROIMMUN - IgG - Anti-SARS-CoV-2 ELISA IgG | 0.9, 1 | Validated by manufacturer |
| The United Kingdom | Public Health Scotland (Public Health Scotland)[99] | 2021-06-28 - 2021-08-01 | National | Moderate | 2,990 | Adults (18-64 years) | Female | Sequential | Pregnant or parturient women | 12.7% | Roche Diagnostics - IgG, IgM, IgA - Elecsys  Anti‐SARS‐CoV‐2 (N) | 1, 0.995 | Validated by manufacturer |
| The United Kingdom | Public Health Scotland (Public Health Scotland)[99] | 2021-06-28 - 2021-08-01 | National | Low | 3,427 | Multiple groups | All | Probability | Residual sera | 69.4% | DiaSorin - IgG - Liaison SARS-CoV-2 S1/S2 IgG | 0.974, 0.985 | Validated by manufacturer |
| The United Kingdom | Public Health Scotland (Public Health Scotland)[100] | 2021-05-24 - 2021-06-27 | National | Moderate | 2,494 | Multiple groups | All | Sequential | Blood donors | 76.8% | EUROIMMUN - IgG - Anti-SARS-CoV-2 ELISA IgG | 0.9, 1 | Validated by manufacturer |
| The United Kingdom | Public Health Scotland (Public Health Scotland)[100] | 2021-05-24 - 2021-06-27 | National | Moderate | 3,052 | Adults (18-64 years) | Female | Sequential | Pregnant or parturient women | 11.2% | Roche Diagnostics - IgG, IgM, IgA - Elecsys  Anti‐SARS‐CoV‐2 (N) | 1, 0.995 | Validated by manufacturer |
| The United Kingdom | Public Health Scotland (Public Health Scotland)[100] | 2021-05-24 - 2021-06-27 | National | Low | 3,554 | Multiple groups | All | Probability | Residual sera | 59.3% | DiaSorin - IgG - Liaison SARS-CoV-2 S1/S2 IgG | 0.974, 0.985 | Validated by manufacturer |
| The United Kingdom | Clarissa Oeser (UK Health Security Agency (UKHSA, formerly PHE))[97] | 2021-05-01 - 2021-06-30 | National | Moderate | 1,186 | Children and Youth (0-17 years) | All | Convenience | Residual sera | 27.7% | Roche Diagnostics - IgG, IgM, IgA - Elecsys  Anti‐SARS‐CoV‐2 (S) | 0.988, 1 | Validated by manufacturer |
| The United Kingdom | Public Health Scotland (Public Health Scotland)[101] | 2021-04-19 - 2021-05-23 | National | Moderate | 2,494 | Multiple groups | All | Sequential | Blood donors | 58.8% | EUROIMMUN - IgG - Anti-SARS-CoV-2 ELISA IgG | 0.9, 1 | Validated by manufacturer |
| The United Kingdom | Public Health Scotland (Public Health Scotland)[101] | 2021-04-19 - 2021-05-23 | National | Moderate | 3,333 | Adults (18-64 years) | Female | Sequential | Pregnant or parturient women | 10% | Roche Diagnostics - IgG, IgM, IgA - Elecsys  Anti‐SARS‐CoV‐2 (N) | 1, 0.995 | Validated by manufacturer |
| The United Kingdom | Public Health Scotland (Public Health Scotland)[101] | 2021-05-03 - 2021-05-09 | National | Low | 713 | Multiple groups | All | Probability | Residual sera | 47.5% | DiaSorin - IgG - Liaison SARS-CoV-2 S1/S2 IgG | 0.974, 0.985 | Validated by manufacturer |
| The United Kingdom | Public Health Scotland (Public Health Scotland)[102] | 2021-04-12 - 2021-04-18 | National | Low | 708 | Multiple groups | All | Probability | Residual sera | 42.8% | DiaSorin - IgG - Liaison SARS-CoV-2 S1/S2 IgG | 0.974, 0.985 | Validated by manufacturer |
| The United Kingdom | Public Health Scotland (Public Health Scotland)[102] | 2021-03-15 - 2021-04-18 | National | Moderate | 2,493 | Multiple groups | All | Sequential | Blood donors | 39.7% | EUROIMMUN - IgG - Anti-SARS-CoV-2 ELISA IgG | 0.9, 1 | Validated by manufacturer |
| The United Kingdom | Public Health Scotland (Public Health Scotland)[102] | 2021-03-15 - 2021-04-18 | National | Moderate | 3,326 | Adults (18-64 years) | Female | Sequential | Pregnant or parturient women | 11.4% | Roche Diagnostics - IgG, IgM, IgA - Elecsys  Anti‐SARS‐CoV‐2 (N) | 1, 0.995 | Validated by manufacturer |
| The United Kingdom | Clarissa Oeser (UK Health Security Agency (UKHSA, formerly PHE))[97] | 2021-03-01 - 2021-04-30 | National | Moderate | 925 | Children and Youth (0-17 years) | All | Convenience | Residual sera | 31.1% | Roche Diagnostics - IgG, IgM, IgA - Elecsys  Anti‐SARS‐CoV‐2 (S) | 0.988, 1 | Validated by manufacturer |
| The United Kingdom | Robert W Aldridge (University College London)[103] | 2021-02-01 - 2021-05-03 | National | Moderate | 10,330 | Multiple groups | All | Convenience | Household and community samples | 8.2% | Roche Diagnostics - IgG, IgM, IgA - Elecsys  Anti‐SARS‐CoV‐2 (N) | 1, 0.995 | Validated by manufacturer |
| The United Kingdom | Heather J Whitaker (Public Health England)[104] | 2021-02-22 - 2021-03-21 | National | Moderate | 7,720 | Multiple groups | All | Convenience | Blood donors | 46.4% | Roche Diagnostics - IgG, IgM, IgA - Elecsys  Anti‐SARS‐CoV‐2 (S) | 0.988, 1 | Validated by manufacturer |
| The United Kingdom | Public Health Scotland (Public Health Scotland)[105] | 2021-02-08 - 2021-03-14 | National | Moderate | 2,492 | Multiple groups | All | Sequential | Blood donors | 22.1% | EUROIMMUN - IgG - Anti-SARS-CoV-2 ELISA IgG | 0.9, 1 | Validated by manufacturer |
| The United Kingdom | Public Health Scotland (Public Health Scotland)[105] | 2021-02-08 - 2021-03-14 | National | Moderate | 3,355 | Adults (18-64 years) | Female | Sequential | Pregnant or parturient women | 8.9% | Roche Diagnostics - IgG, IgM, IgA - Elecsys  Anti‐SARS‐CoV‐2 (N) | 1, 0.995 | Validated by manufacturer |
| The United Kingdom | Clarissa Oeser (UK Health Security Agency (UKHSA, formerly PHE))[97] | 2021-01-01 - 2021-02-28 | National | Moderate | 970 | Children and Youth (0-17 years) | All | Convenience | Residual sera | 20.7% | Roche Diagnostics - IgG, IgM, IgA - Elecsys  Anti‐SARS‐CoV‐2 (S) | 0.988, 1 | Validated by manufacturer |
| The United Kingdom | Public Health Scotland (Public Health Scotland)[106] | 2021-01-04 - 2021-02-07 | National | Moderate | 2,497 | Multiple groups | All | Sequential | Blood donors | 12.1% | EUROIMMUN - IgG - Anti-SARS-CoV-2 ELISA IgG | 0.9, 1 | Validated by manufacturer |
| The United Kingdom | Public Health Scotland (Public Health Scotland)[106] | 2021-01-04 - 2021-02-07 | National | Moderate | 3,478 | Adults (18-64 years) | Female | Sequential | Pregnant or parturient women | 6.9% | Roche Diagnostics - IgG, IgM, IgA - Elecsys  Anti‐SARS‐CoV‐2 (N) | 1, 0.995 | Validated by manufacturer |
| The United Kingdom | Public Health Scotland (Public Health Scotland)[107] | 2020-11-30 - 2021-01-03 | National | Moderate | 2,499 | Multiple groups | All | Sequential | Blood donors | 4.2% | EUROIMMUN - IgG - Anti-SARS-CoV-2 ELISA IgG | 0.9, 1 | Validated by manufacturer |
| The United Kingdom | Public Health Scotland (Public Health Scotland)[107] | 2020-11-30 - 2021-01-03 | National | Moderate | 3,052 | Adults (18-64 years) | Female | Sequential | Pregnant or parturient women | 5.3% | Roche Diagnostics - IgG, IgM, IgA - Elecsys  Anti‐SARS‐CoV‐2 (N) | 1, 0.995 | Validated by manufacturer |
| The United Kingdom | Clarissa Oeser (UK Health Security Agency (UKHSA, formerly PHE))[97] | 2020-11-01 - 2020-12-31 | National | Moderate | 711 | Children and Youth (0-17 years) | All | Convenience | Residual sera | 13.2% | Roche Diagnostics - IgG, IgM, IgA - Elecsys  Anti‐SARS‐CoV‐2 (S) | 0.988, 1 | Validated by manufacturer |
| The United Kingdom | Heather Whitaker (UK Health Security Agency)[108] | 2020-11-01 - 2020-12-31 | National | Moderate | 4,934 | Multiple groups | All | Sequential | Residual sera | 6.6% | EUROIMMUN - IgG - Anti-SARS-CoV-2 ELISA IgG | 0.9, 1 | Validated by manufacturer |
| The United Kingdom | Shamez Ladhani (Public Health England)[109] | 2020-11-17 - 2020-11-30 | National | Moderate | 1,216 | Multiple groups | All | Probability | Household and community samples | 10.4% | Abbott Laboratories - IgG - Abbott Architect SARS-CoV-2 IgG | 0.99, 1 | Validated by manufacturer |
| The United Kingdom | Public Health Scotland (Public Health Scotland)[110] | 2020-10-26 - 2020-11-29 | National | Moderate | 2,499 | Multiple groups | All | Sequential | Blood donors | 4.2% | EUROIMMUN - IgG - Anti-SARS-CoV-2 ELISA IgG | 0.9, 1 | Validated by manufacturer |
| The United Kingdom | Heather Whitaker (UK Health Security Agency)[108] | 2020-09-01 - 2020-10-01 | National | Moderate | 5,478 | Multiple groups | All | Sequential | Residual sera | 5.5% | EUROIMMUN - IgG - Anti-SARS-CoV-2 ELISA IgG | 0.9, 1 | Validated by manufacturer |
| The United Kingdom | Public Health England (Public Health England)[111] | 2020-08-19 - 2020-09-13 | National | Low | 7,888 | Multiple groups | All | Probability | Blood donors | 6.1% | EUROIMMUN - IgG - Anti-SARS-CoV-2 ELISA IgG | 0.9, 1 | Validated by manufacturer |
| The United Kingdom | Public Health England (Public Health England)[111] | 2020-08-13 - 2020-09-06 | National | Low | 7,899 | Multiple groups | All | Probability | Blood donors | 5.5% | EUROIMMUN - IgG - Anti-SARS-CoV-2 ELISA IgG | 0.9, 1 | Validated by manufacturer |
| The United Kingdom | Heather Whitaker (UK Health Security Agency)[108] | 2020-07-01 - 2020-08-31 | National | Moderate | 5,781 | Multiple groups | All | Sequential | Residual sera | 5.2% | EUROIMMUN - IgG - Anti-SARS-CoV-2 ELISA IgG | 0.9, 1 | Validated by manufacturer |
| The United Kingdom | Public Health England (Public Health England)[111] | 2020-07-13 - 2020-08-07 | National | Low | 8,538 | Multiple groups | All | Probability | Blood donors | 5.4% | EUROIMMUN - IgG - Anti-SARS-CoV-2 ELISA IgG | 0.9, 1 | Validated by manufacturer |
| The United Kingdom | Public Health England (Public Health England)[111] | 2020-07-01 - 2020-08-14 | National | Low | 2,153 | Seniors (65+ years) | All | Probability | Residual sera | 3.4% | Abbott Laboratories - IgG - Abbott Architect SARS-CoV-2 IgG | 0.99, 1 | Validated by manufacturer |
| The United Kingdom | Public Health England (Public Health England)[111] | 2020-06-01 - 2020-08-02 | National | Moderate | 285 | Children and Youth (0-17 years) | All | Probability | Household and community samples | 4.9% | Abbott Laboratories - IgG - Abbott Architect SARS-CoV-2 IgG | 0.99, 1 | Validated by manufacturer |
| The United Kingdom | Public Health England (Public Health England)[111] | 2020-06-01 - 2020-08-02 | National | Low | 2,062 | Children and Youth (0-17 years) | All | Probability | Residual sera | 2.4% | Abbott Laboratories - IgG - Abbott Architect SARS-CoV-2 IgG | 0.99, 1 | Validated by manufacturer |
| The United Kingdom | Public Health England (Public Health England)[111] | 2020-06-08 - 2020-07-06 | National | Low | 11,385 | Multiple groups | All | Probability | Blood donors | 7.1% | EUROIMMUN - IgG - Anti-SARS-CoV-2 ELISA IgG | 0.9, 1 | Validated by manufacturer |
| The United Kingdom | Heather Whitaker (UK Health Security Agency)[108] | 2020-05-01 - 2020-06-30 | National | Moderate | 5,455 | Multiple groups | All | Sequential | Residual sera | 4.1% | EUROIMMUN - IgG - Anti-SARS-CoV-2 ELISA IgG | 0.9, 1 | Validated by manufacturer |
| The United Kingdom | Public Health England (Public Health England)[111] | 2020-05-13 - 2020-06-07 | National | Low | 7,777 | Adults (18-64 years) | All | Probability | Blood donors | 8.2% | EUROIMMUN - IgG - Anti-SARS-CoV-2 ELISA IgG | 0.9, 1 | Validated by manufacturer |
| The United Kingdom | Public Health England (Public Health England)[111] | 2020-05-01 - 2020-05-31 | National | Moderate | 1,061 | Children and Youth (0-17 years) | All | Probability | Residual sera | 7.8% | Abbott Laboratories - IgG - Abbott Architect SARS-CoV-2 IgG | 0.99, 1 | Validated by manufacturer |
| The United Kingdom | Public Health England (Public Health England)[111] | 2020-03-16 - 2020-06-30 | National | Low | 6,000 | Seniors (65+ years) | All | Probability | Residual sera | 3.9% | EUROIMMUN - IgG - Anti-SARS-CoV-2 ELISA IgG | 0.9, 1 | Validated by manufacturer |
| The United Kingdom | Public Health England (Public Health England)[111] | 2020-04-01 - 2020-04-30 | National | Moderate | 647 | Children and Youth (0-17 years) | All | Probability | Residual sera | 6.6% | Abbott Laboratories - IgG - Abbott Architect SARS-CoV-2 IgG | 0.99, 1 | Validated by manufacturer |
| The United Kingdom | Public Health England (Public Health England)[111] | 2020-04-01 - 2020-04-30 | National | Moderate | 201 | Children and Youth (0-17 years) | All | Probability | Household and community samples | 4.5% | Abbott Laboratories - IgG - Abbott Architect SARS-CoV-2 IgG | 0.99, 1 | Validated by manufacturer |
| The United Kingdom | Public Health England (Public Health England)[111] | 2020-02-01 - 2020-03-31 | National | Moderate | 106 | Children and Youth (0-17 years) | All | Probability | Household and community samples | 1.4% | Abbott Laboratories - IgG - Abbott Architect SARS-CoV-2 IgG | 0.99, 1 | Validated by manufacturer |

#### South-East Asia region

| Country (Location) | Author (Organization) | Sampling Dates (YMD) | Geographic scope | Overall risk of bias | Sample size | Age | Sex | Sampling method | Sample frame | Seroprevalence (95% CI) | Test Manufacturers - Isotypes - Names | Test Sens, Spec | Sens, Spec Source |
| --- | --- | --- | --- | --- | --- | --- | --- | --- | --- | --- | --- | --- | --- |
| India | Manoj V Murhekar (Indian Council of Medical Research)[112] | 2020-12-18 - 2021-01-06 | National | Low | 28,598 | Multiple groups | All | Probability | Household and community samples | 24.6% | Siemens - IgG, IgM, IgA - Atellica  IM SARS-CoV-2 Total (COV2T),Abbott Laboratories - IgG - Abbott Architect SARS-CoV-2 IgG | 1, 0.998 | Complex test algorithm: Manual calculation |
| India | Manoj V Murhekar (The Indian Council of Medical Research (ICMR))[112] | 2020-08-18 - 2020-09-20 | National | Low | 29,082 | Multiple groups | All | Probability | Household and community samples | 7% | Abbott Laboratories - IgG - Abbott Architect SARS-CoV-2 IgG | 0.99, 1 | Validated by manufacturer |
| Indonesia | Dr. dr. Tri Yunis Miko Wahyono, M.Sc (University of Indonesia)[113] | 2020-12-15 - 2021-03-03 | National | Moderate | 10,161 | Multiple groups | All | Probability | Household and community samples | 20.5% (95% CI 19.7-21.3%) | Beijing Wantai Biological - IgG IgM IgA - Wantai SARS-CoV-2 Total Ab ELISA | 0.967, 0.975 | Validated by manufacturer |
| Nepal | Government of Nepal (Government of Nepal)[114] | 2020-10-09 - 2020-10-22 | National | Low | 3,040 | Multiple groups | All | Probability | Household and community samples | 14.4% | Beijing Wantai Biological - IgG, IgM, IgA - Wantai SARS-CoV-2 Total Ab ELISA | 0.967, 0.975 | Validated by manufacturer |
| Thailand | Sutthichai Nakphook, MD (Ministry of Public Health)[115] | 2020-12-18 - 2021-02-02 | National | Moderate | 1,200 | Multiple groups | All | Probability | Household and community samples | 0.4% (95% CI 0.1-1%) | Beijing Wantai Biological - IgG IgM IgA - Wantai SARS-CoV-2 Total Ab ELISA | 0.967, 0.975 | Validated by manufacturer |

####

#### Western Pacific region

| Country (Location) | Author (Organization) | Sampling Dates (YMD) | Geographic scope | Overall risk of bias | Sample size | Age | Sex | Sampling method | Sample frame | Seroprevalence (95% CI) | Test Manufacturers - Isotypes - Names | Test Sens, Spec | Sens, Spec Source |
| --- | --- | --- | --- | --- | --- | --- | --- | --- | --- | --- | --- | --- | --- |
| Australia | Kaitlyn Vette (University of Sydney)[116] | 2020-06-19 - 2020-08-06 | National | Moderate | 2,972 | Adults (18-64 years) | Female | Convenience | Pregnant or parturient women | 0.5% | Beijing Wantai Biological - IgG, IgM, IgA - Wantai SARS-CoV-2 Total Ab ELISA | 0.967, 0.975 | Validated by manufacturer |
| Australia | Nicholas Coatsworth (Australian National University)[117] | 2020-06-02 - 2020-07-17 | National | Moderate | 2,991 | Multiple groups | All | Sequential | Residual sera | 0.2% | NA - - Author designed (ELISA) - MULTIPLEXED | 1, 0.989 | Validated by independent authors/third party/non-developers |
| China | Zhongjie Li (Chinese Center for Disease Control and Prevention)[118] | 2020-04-10 - 2020-04-18 | National | Moderate | 34,857 | Multiple groups | All | Probability | Household and community samples | 1.2% | Guangzhou Wondfo Biotech Co. Ltd - IgG, IgM - Finecare SARS-CoV-2 Antibody test,Innovita Biological Technology Co. Ltd - IgG - 2019-nCoV IgG Rapid Test,Bioscience Diagnostics - IgG, IgM - Magnetic Chemiluminescence Enzyme Immunoassay Kit,NA - - Author designed (Neutralization Assay) | N/A | Complex test algorithm: Manual review |
| Japan | Takashi Yoshiyama (Government of Japan)[119] | 2020-06-01 - 2020-06-07 | National | Moderate | 7,950 | Multiple groups | All | Probability | Household and community samples | 0.1% | Roche Diagnostics - IgG, IgM, IgA - Elecsys  Anti‐SARS‐CoV‐2 (N),Abbott Laboratories - IgG - Abbott Architect SARS-CoV-2 IgG | 0.99, 1 | Complex test algorithm: Manual calculation |
| Malaysia | Chong Zhuo Lin (Institute for Public Health)[120] | 2020-08-07 - 2020-10-06 | National | Moderate | 5,131 | Multiple groups | All | Probability | Household and community samples | 0.6% (95% CI 0.4-0.8%) | Beijing Wantai Biological - IgG IgM IgA - Wantai SARS-CoV-2 Total Ab ELISA,GenScript - IgG IgM - cPass SARS-CoV-2 Surrogate Virus Neutralization Test ELISA Kit | 0.907, 1 | Complex test algorithm: Manual calculation |
| Mongolia | Battogtokh Chimeddorj (Mongolian National University of Medical Sciences (MNUMS))[121] | 2021-01-18 - 2021-03-15 | National | Moderate | 4,530 | Multiple groups | All | Convenience | Household and community samples | 1.6% (95% CI 1.3-2%) | Beijing Wantai Biological - IgG IgM IgA - Wantai SARS-CoV-2 Total Ab ELISA,Kantaro Biosciences LLC - IgG - COVID-SeroKlir, Kantaro Semi-Quantitative SARS-CoV-2 IgG Antibody Kit | 0.955, 1 | Complex test algorithm: Manual calculation |
| Mongolia | Battogtokh Chimeddorj (Mongolian National University of Medical Sciences (MNUMS))[121] | 2020-10-18 - 2020-12-05 | National | Moderate | 5,000 | Multiple groups | All | Convenience | Household and community samples | 1.4% (95% CI 1.1-1.8%) | Beijing Wantai Biological - IgG IgM IgA - Wantai SARS-CoV-2 Total Ab ELISA,Kantaro Biosciences LLC - IgG - COVID-SeroKlir, Kantaro Semi-Quantitative SARS-CoV-2 IgG Antibody Kit | 0.955, 1 | Complex test algorithm: Manual calculation |
| Papua New Guinea | Moses Laman (Papua New Guinea Institute of Medical Research)[122] | 2020-05-01 - 2020-06-30 | National | Moderate | 12,177 | Multiple groups | All | Convenience | Household and community samples | 2.6% (95% CI 2.4-2.9%) | Hanghzhou AllTest Biotech Co. Ltd - IgG IgM - 2019-nCoV IgG/IgM Rapid Test Cassette | 1, 0.962 | Validated by manufacturer |
| Republic of Korea | Ah-Ra Kim (National Institute of Infectious Diseases)[123] | 2020-11-01 - 2020-12-12 | National | Moderate | 910 | Multiple groups | All | Convenience | Residual sera | 0.1% | Roche Diagnostics - IgG, IgM, IgA - Elecsys  Anti‐SARS‐CoV‐2 (N),Abbott Laboratories - IgG - Abbott Architect SARS-CoV-2 IgG,Beckman Coulter - IgG - Access SARS-CoV-2 IgG Antibody Test,SG medical, Inc. - - R-FIND COVID-19 ELISA,Sugentech, Inc. - IgG, IgM - SGTi-flex COVID-19 IgM/IgG (manual),NA - - Author designed (Neutralization Assay) | N/A | Complex test algorithm: Manual review |
| Republic of Korea | Eun Hee Nah (Korea Association of Health Promotion)[124] | 2020-09-24 - 2020-12-09 | National | Moderate | 4,085 | Multiple groups | All | Probability | Residual sera | 0.4% | Roche Diagnostics - IgG, IgM, IgA - Elecsys  Anti‐SARS‐CoV‐2 (N) | 1, 0.995 | Validated by manufacturer |

## Table B. Additional studies used in meta-analysis and meta-regression.

#### Africa region

| Country (Location) | Author (Organization) | Sampling Dates (YMD) | Geographic scope | Overall risk of bias | Sample size | Age | Sex | Sampling method | Sample frame | Seroprevalence (95% CI) | Test Manufacturers - Isotypes - Names | Test Sens, Spec | Sens, Spec Source |
| --- | --- | --- | --- | --- | --- | --- | --- | --- | --- | --- | --- | --- | --- |
| Democratic Republic of the Congo - HRP (All the 35 health districts of Kinshasa) | Makiala Mandanda Sheila (Institut National de Recherche Biomédicale)[125] | 2021-03-06 - 2021-03-14 | Subnational | Moderate | 2,476 | Multiple groups | All | Probability | Household and community samples | 72.1% (95% CI 70.3-73.9%) | Beijing Wantai Biological - IgG IgM IgA - Wantai SARS-CoV-2 Total Ab ELISA | 0.967, 0.975 | Validated by manufacturer |
| Ethiopia - HRP (Oromia, Harari) | Nega Assefa (The London School of Hygiene & Tropical Medicine)[126] | 2020-04-01 - 2021-03-31 | Subnational | Moderate | 1,447 | Adults (18-64 years) | Female | Probability | Pregnant or parturient women | 5.7% | Beijing Wantai Biological - IgG, IgM, IgA - Wantai Rapid Test for Total Antibody to SARS-CoV-2 | 0.947, 0.989 | Validated by manufacturer |
| Nigeria - HRP (Anambra State) | OgoChukwu Vincent Okpala (Anambra State Ministry of Health)[127] | 2020-12-08 - 2020-12-15 | Subnational | Moderate | 3,142 | Multiple groups | All | Probability | Household and community samples | 17.6% | Hangzhou Realy Tech Co. Ltd - IgG, IgM - Realy-Tech 2019 nCOV/COVID-19 IgG/IgM Rapid Test | 0.967, 0.937 | Validated by manufacturer |
| Nigeria - HRP (Enugu State) | Kristen A. Stafford (UMB); Laura Steinhardt (CDC); Elsie Ilori (NCDC); Rosemary Audu (NIMR) (University of Maryland, Baltimore)[128] | 2020-09-21 - 2020-10-27 | Subnational | Low | 2,147 | Multiple groups | All | Probability | Household and community samples | 25.2% | Abbott Laboratories - IgG - Abbott Architect SARS-CoV-2 IgG,EUROIMMUN AG - IgG - SARS-CoV-2-NCP-IgG ELISA | N/A | Complex test algorithm: Manual review |
| Nigeria - HRP (Gombe State) | Kristen A. Stafford (UMB); Laura Steinhardt (CDC); Elsie Ilori (NCDC); Rosemary Audu (NIMR) (University of Maryland, Baltimore)[128] | 2020-09-21 - 2020-10-27 | Subnational | Low | 3,432 | Multiple groups | All | Probability | Household and community samples | 9.3% | Abbott Laboratories - IgG - Abbott Architect SARS-CoV-2 IgG,EUROIMMUN AG - IgG - SARS-CoV-2-NCP-IgG ELISA | N/A | Complex test algorithm: Manual review |
| Nigeria - HRP (Nasarawa State) | Kristen A. Stafford (UMB); Laura Steinhardt (CDC); Elsie Ilori (NCDC); Rosemary Audu (NIMR) (University of Maryland, Baltimore)[128] | 2020-09-21 - 2020-10-27 | Subnational | Low | 2,657 | Multiple groups | All | Probability | Household and community samples | 18% | Abbott Laboratories - IgG - Abbott Architect SARS-CoV-2 IgG,EUROIMMUN AG - IgG - SARS-CoV-2-NCP-IgG ELISA | N/A | Complex test algorithm: Manual review |
| Nigeria - HRP (Lagos State) | Kristen A. Stafford (UMB); Laura Steinhardt (CDC); Elsie Ilori (NCDC); Rosemary Audu (NIMR) (Nigeria Institute for Medical Research)[128] | 2020-09-21 - 2020-10-27 | Subnational | Low | 2,393 | Multiple groups | All | Probability | Household and community samples | 23.2% | Abbott Laboratories - IgG - Abbott Architect SARS-CoV-2 IgG,EUROIMMUN AG - IgG - SARS-CoV-2-NCP-IgG ELISA | N/A | Complex test algorithm: Manual review |
| Nigeria - HRP (Niger State) | Hussaini Majiya (Ibrahim Badamasi Babangida University)[129] | 2020-06-26 - 2020-06-30 | Subnational | Moderate | 185 | Multiple groups | All | Probability | Household and community samples | 25.4% | Cambridge Network - IgG, IgM - COVID-19 IgG and IgM Rapid Test | 1, 1 | Validated by independent authors/third party/non-developers |
| South Africa (Gauteng) | Jaya George (National Health Laboratory Service)[130] | 2020-10-01 - 2020-10-31 | Subnational | Moderate | 1,664 | Multiple groups | All | Convenience | Residual sera | 28.2% | Roche Diagnostics - IgG, IgM, IgA - Elecsys  Anti‐SARS‐CoV‐2 (N) | 1, 0.995 | Validated by manufacturer |

#### Americas region

| Country (Location) | Author (Organization) | Sampling Dates (YMD) | Geographic scope | Overall risk of bias | Sample size | Age | Sex | Sampling method | Sample frame | Seroprevalence (95% CI) | Test Manufacturers - Isotypes - Names | Test Sens, Spec | Sens, Spec Source |
| --- | --- | --- | --- | --- | --- | --- | --- | --- | --- | --- | --- | --- | --- |
| Brazil - HRP (Rio Grande do Sul) | Aluisio Barros (Universidade Federal de Pelotas)[131] | 2021-04-09 - 2021-04-11 | Subnational | Moderate | 4,499 | Multiple groups | All | Probability | Household and community samples | 18.2% | NA - IgG - Author designed (ELISA) -Spike | 0.95, 0.986 | Validated by developers |
| Brazil - HRP (São Paulo) | Nayara Vale (Universidade Federal de São Paulo)[132] | 2021-02-01 - 2021-02-28 | Subnational | Moderate | 940 | Adults (18-64 years) | All | Probability | Blood donors | 30.9% | Roche Diagnostics - IgG, IgM, IgA - Elecsys  Anti‐SARS‐CoV‐2 (N) | 1, 0.995 | Validated by manufacturer |
| Brazil - HRP (Rio Grande do Sul) | Aluisio Barros (Universidade Federal de Pelotas)[131] | 2021-02-05 - 2021-02-07 | Subnational | Moderate | 4,501 | Multiple groups | All | Probability | Household and community samples | 10% | NA - IgG - Author designed (ELISA) -Spike | 0.95, 0.986 | Validated by developers |
| Brazil - HRP (Greater São Paulo) | José Olimpio Moura de Albuquerque (Secretaria Municipal de Saude de Sao Paulo)[133] | 2021-02-02 - 2021-02-04 | Subnational | Moderate | 1,742 | Multiple groups | All | Probability | Household and community samples | 16% | Celer Technologies Inc. - IgG, IgM - One Step COVID-19 Test | 0.848, 0.99 | Validated by independent authors/third party/non-developers |
| Brazil - HRP (Greater São Paulo) | José Olimpio Moura de Albuquerque (Secretaria Municipal de Saude de Sao Paulo)[133] | 2021-01-19 - 2021-01-21 | Subnational | Moderate | 1,795 | Multiple groups | All | Probability | Household and community samples | 13.9% | Celer Technologies Inc. - IgG, IgM - One Step COVID-19 Test | 0.848, 0.99 | Validated by independent authors/third party/non-developers |
| Brazil - HRP (Greater São Paulo) | José Olimpio Moura de Albuquerque (Secretaria Municipal de Saude de Sao Paulo)[133] | 2021-01-05 - 2021-01-07 | Subnational | Moderate | 1,908 | Multiple groups | All | Probability | Household and community samples | 14.1% | Celer Technologies Inc. - IgG, IgM - One Step COVID-19 Test | 0.848, 0.99 | Validated by independent authors/third party/non-developers |
| Brazil - HRP (Maranhão) | Antonio da Silva (Universidade Federal do Maranhao)[134] | 2020-10-19 - 2020-10-30 | Subnational | Low | 4,563 | Multiple groups | All | Probability | Household and community samples | 38.1% | Roche Diagnostics - IgG, IgM, IgA - Elecsys  Anti‐SARS‐CoV‐2 (N) | 1, 0.995 | Validated by manufacturer |
| Brazil - HRP (São Paulo) | Nayara Vale (Universidade Federal de São Paulo)[132] | 2020-10-01 - 2020-10-31 | Subnational | Moderate | 940 | Adults (18-64 years) | All | Probability | Blood donors | 22% | Roche Diagnostics - IgG, IgM, IgA - Elecsys  Anti‐SARS‐CoV‐2 (N) | 1, 0.995 | Validated by manufacturer |
| Brazil - HRP (Vale do Rio Pardo) | Ana Schneider (Universidade de Santa Cruz do Sul)[135] | 2020-10-04 - 2020-10-17 | Subnational | Moderate | 1,063 | Multiple groups | All | Probability | Household and community samples | 3.4% | Beijing Lepu Medical Technology - IgG, IgM - Leccurate SARS-CoV-2 Antibody Rapid Test Kit (Colloidal Gold Immunochromatography) | 0.989, 0.976 | Validated by manufacturer |
| Brazil - HRP (Greater São Paulo) | José Olimpio Moura de Albuquerque (Secretaria Municipal de Saude de Sao Paulo)[133] | 2020-09-22 - 2020-09-24 | Subnational | Moderate | 2,012 | Multiple groups | All | Probability | Household and community samples | 13.6% | Celer Technologies Inc. - IgG, IgM - One Step COVID-19 Test | 0.848, 0.99 | Validated by independent authors/third party/non-developers |
| Brazil - HRP (Greater São Paulo) | José Olimpio Moura de Albuquerque (Secretaria Municipal de Saude de Sao Paulo)[133] | 2020-09-08 - 2020-09-10 | Subnational | Moderate | 2,125 | Multiple groups | All | Probability | Household and community samples | 11.9% | Celer Technologies Inc. - IgG, IgM - One Step COVID-19 Test | 0.848, 0.99 | Validated by independent authors/third party/non-developers |
| Brazil - HRP (Rio Grande do Sul) | Pedro C. Hallal (Universidade Federal de Pelotas )[136] | 2020-09-04 - 2020-09-06 | Subnational | Moderate | 4,500 | Multiple groups | All | Probability | Household and community samples | 1.6% | Guangzhou Wondfo Biotech Co. Ltd - IgG, IgM, IgA - Wondfo SARS-CoV-2 Antibody Test | 0.864, 0.996 | Validated by manufacturer |
| Brazil - HRP (Greater São Paulo) | José Olimpio Moura de Albuquerque (Secretaria Municipal de Saude de Sao Paulo)[133] | 2020-08-25 - 2020-08-27 | Subnational | Moderate | 2,225 | Multiple groups | All | Probability | Household and community samples | 13.9% | Celer Technologies Inc. - IgG, IgM - One Step COVID-19 Test | 0.848, 0.99 | Validated by independent authors/third party/non-developers |
| Brazil - HRP (Greater São Paulo) | José Olimpio Moura de Albuquerque (Secretaria Municipal de Saude de Sao Paulo)[133] | 2020-08-11 - 2020-08-13 | Subnational | Moderate | 2,447 | Multiple groups | All | Probability | Household and community samples | 11% | Celer Technologies Inc. - IgG, IgM - One Step COVID-19 Test | 0.848, 0.99 | Validated by independent authors/third party/non-developers |
| Brazil - HRP (Minas Gerais) | Daniel Chaves (Fundacao Hemominas)[137] | 2020-03-01 - 2020-12-31 | Subnational | Moderate | 7,837 | Multiple groups | All | Probability | Blood donors | 5.6% | Abbott Laboratories - IgG - Abbott Architect SARS-CoV-2 IgG | 0.99, 1 | Validated by manufacturer |
| Brazil - HRP (Greater São Paulo) | José Olimpio Moura de Albuquerque (Secretaria Municipal de Saude de Sao Paulo)[133] | 2020-07-28 - 2020-07-30 | Subnational | Moderate | 2,529 | Multiple groups | All | Probability | Household and community samples | 10.9% | Celer Technologies Inc. - IgG, IgM - One Step COVID-19 Test | 0.848, 0.99 | Validated by independent authors/third party/non-developers |
| Brazil - HRP (Greater São Paulo) | José Olimpio Moura de Albuquerque (Secretaria Municipal de Saude de Sao Paulo)[133] | 2020-07-13 - 2020-07-16 | Subnational | Moderate | 2,323 | Multiple groups | All | Probability | Household and community samples | 11.1% | Celer Technologies Inc. - IgG, IgM - One Step COVID-19 Test | 0.848, 0.99 | Validated by independent authors/third party/non-developers |
| Brazil - HRP (Greater São Paulo) | José Olimpio Moura de Albuquerque (Secretaria Municipal de Saude de Sao Paulo)[133] | 2020-06-29 - 2020-07-02 | Subnational | Moderate | 2,481 | Multiple groups | All | Probability | Household and community samples | 9.7% | Celer Technologies Inc. - IgG, IgM - One Step COVID-19 Test | 0.848, 0.99 | Validated by independent authors/third party/non-developers |
| Brazil - HRP (São Paulo) | Nayara Vale (Universidade Federal de São Paulo)[132] | 2020-06-01 - 2020-06-30 | Subnational | Moderate | 926 | Adults (18-64 years) | All | Probability | Blood donors | 11.4% | Roche Diagnostics - IgG, IgM, IgA - Elecsys  Anti‐SARS‐CoV‐2 (N) | 1, 0.995 | Validated by manufacturer |
| Brazil - HRP (Greater São Paulo) | José Olimpio Moura de Albuquerque (Secretaria Municipal de Saude de Sao Paulo)[133] | 2020-06-10 - 2020-06-17 | Subnational | Moderate | 2,645 | Multiple groups | All | Probability | Household and community samples | 9.5% | Celer Technologies Inc. - IgG, IgM - One Step COVID-19 Test | 0.848, 0.99 | Validated by independent authors/third party/non-developers |
| Brazil - HRP (Espirito Santo) | Ethel Leonor Noia Maciel (Laboratorio de Epidemiologia)[138] | 2020-05-10 - 2020-06-21 | Subnational | Moderate | 1,693 | Children and Youth (0-17 years) | All | Probability | Household and community samples | 6.1% | Celer Technologies Inc. - IgG, IgM - One Step COVID-19 Test | 0.864, 0.996 | Validated by independent authors/third party/non-developers |
| Colombia - HRP (Department of Cesar, Department of La Guajira) | Gustavo Concha (Medical Mission Institute)[139] | 2021-03-28 - 2021-04-26 | Subnational | Moderate | 452 | Multiple groups | All | Convenience | Household and community samples | 27.7% | PharmACT - IgG, IgM - BELTEST-IT COV-2 Rapid Test | 0.982, 0.997 | Validated by manufacturer |
| Colombia - HRP (Cordoba Department) | Evelin Garay (Colombia-Universidad de Cordoba)[140] | 2020-07-15 - 2020-11-15 | Subnational | Moderate | 2,564 | Multiple groups | All | Stratified non-probability | Household and community samples | 40.8% | Eurofins Ingenasa - IgG, IgM, IgA - INgezim COVID 19 DR | 0.983, 0.992 | Validated by manufacturer |
| Colombia - HRP (Cordoba) | Nelson Alvis Guzma (Universidad de Cordoba)[141] | 2020-03-15 - 2020-10-02 | Subnational | Moderate | 2,447 | Multiple groups | All | Probability | Household and community samples | 40.8% | Eurofins Ingenasa - IgG, IgM, IgA - INgezim COVID 19 DR | 0.983, 0.992 | Validated by manufacturer |
| Peru - HRP (Cusco Region) | Charles Huamani (Universidad Andina del Cusco)[142] | 2020-09-12 - 2020-09-27 | Subnational | Moderate | 1,924 | Multiple groups | All | Probability | Household and community samples | 33.1% | Roche Diagnostics - IgG, IgM, IgA - Elecsys  Anti‐SARS‐CoV‐2 (N) | 1, 0.995 | Validated by manufacturer |
| Canada (Quebec) | Antoine Lewin (Hema Quebec)[143] | 2021-01-25 - 2021-03-11 | Subnational | Moderate | 7,924 | Multiple groups | All | Sequential | Blood donors | 15% | NA - IgG, IgM - Author designed (ELISA) -Spike | 0.989, 0.985 | Validated by developers |
| Canada (Alberta) | Carmen Charlton (Alberta Precision Laboratories)[144] | 2021-01-11 - 2021-01-14 | Subnational | Moderate | 11,049 | Multiple groups | All | Sequential | Residual sera | 3.6% | Abbott Laboratories - IgG - Abbott Architect SARS-CoV-2 IgG,DiaSorin - IgG - Liaison SARS-CoV-2 S1/S2 IgG | 0.964, 1 | Complex test algorithm: Manual calculation |
| Canada (Alberta) | Carmen Charlton (Alberta Precision Laboratories)[144] | 2020-12-07 - 2020-12-10 | Subnational | Moderate | 12,076 | Multiple groups | All | Sequential | Residual sera | 2% | Abbott Laboratories - IgG - Abbott Architect SARS-CoV-2 IgG,DiaSorin - IgG - Liaison SARS-CoV-2 S1/S2 IgG | 0.964, 1 | Complex test algorithm: Manual calculation |
| Canada (Alberta) | Carmen Charlton (Alberta Precision Laboratories)[144] | 2020-11-09 - 2020-11-13 | Subnational | Moderate | 11,471 | Multiple groups | All | Sequential | Residual sera | 1.2% | Abbott Laboratories - IgG - Abbott Architect SARS-CoV-2 IgG,DiaSorin - IgG - Liaison SARS-CoV-2 S1/S2 IgG | 0.964, 1 | Complex test algorithm: Manual calculation |
| Canada (Ontario) | Shelly Bolotin, Vanessa Tran (Public Health Ontario)[145] | 2020-10-01 - 2020-10-30 | Subnational | Moderate | 7,107 | Multiple groups | All | Convenience | Residual sera | 1.2% | Abbott Laboratories - IgG - Abbott Architect SARS-CoV-2 IgG,Ortho Clinical Diagnostics Inc. - IgG - VITROS Immunodiagnostic Products Anti-SARS-CoV-2 IgG | 0.904, 1 | Complex test algorithm: Manual calculation |
| Canada (Alberta) | Carmen Charlton (Alberta Precision Laboratories)[144] | 2020-10-05 - 2020-10-09 | Subnational | Moderate | 13,490 | Multiple groups | All | Sequential | Residual sera | 0.9% | Abbott Laboratories - IgG - Abbott Architect SARS-CoV-2 IgG,DiaSorin - IgG - Liaison SARS-CoV-2 S1/S2 IgG | 0.964, 1 | Complex test algorithm: Manual calculation |
| Canada (Ontario) | Shelly Bolotin, Vanessa Tran (Public Health Ontario)[145] | 2020-09-03 - 2020-09-30 | Subnational | Moderate | 4,901 | Multiple groups | All | Convenience | Residual sera | 0.7% | Abbott Laboratories - IgG - Abbott Architect SARS-CoV-2 IgG,Ortho Clinical Diagnostics Inc. - IgG - VITROS Immunodiagnostic Products Anti-SARS-CoV-2 IgG | 0.904, 1 | Complex test algorithm: Manual calculation |
| Canada (Alberta) | Carmen Charlton (Alberta Precision Laboratories)[144] | 2020-09-07 - 2020-09-11 | Subnational | Moderate | 12,006 | Multiple groups | All | Sequential | Residual sera | 1% | Abbott Laboratories - IgG - Abbott Architect SARS-CoV-2 IgG,DiaSorin - IgG - Liaison SARS-CoV-2 S1/S2 IgG | 0.964, 1 | Complex test algorithm: Manual calculation |
| Canada (Ontario) | Shelly Bolotin, Vanessa Tran (Public Health Ontario)[145] | 2020-08-01 - 2020-08-31 | Subnational | Moderate | 6,789 | Multiple groups | All | Convenience | Residual sera | 1.1% | Abbott Laboratories - IgG - Abbott Architect SARS-CoV-2 IgG,Ortho Clinical Diagnostics Inc. - IgG - VITROS Immunodiagnostic Products Anti-SARS-CoV-2 IgG | 0.904, 1 | Complex test algorithm: Manual calculation |
| Canada (Alberta) | Carmen Charlton (Alberta Precision Laboratories)[144] | 2020-08-04 - 2020-08-11 | Subnational | Moderate | 12,284 | Multiple groups | All | Sequential | Residual sera | 0.7% | Abbott Laboratories - IgG - Abbott Architect SARS-CoV-2 IgG,DiaSorin - IgG - Liaison SARS-CoV-2 S1/S2 IgG | 0.964, 1 | Complex test algorithm: Manual calculation |
| Canada (Ontario) | Shelly Bolotin, Vanessa Tran (Public Health Ontario)[145] | 2020-07-04 - 2020-07-31 | Subnational | Moderate | 7,001 | Multiple groups | All | Convenience | Residual sera | 1.1% | Abbott Laboratories - IgG - Abbott Architect SARS-CoV-2 IgG,Ortho Clinical Diagnostics Inc. - IgG - VITROS Immunodiagnostic Products Anti-SARS-CoV-2 IgG | 0.904, 1 | Complex test algorithm: Manual calculation |
| Canada (Alberta) | Carmen Charlton (Alberta Precision Laboratories)[144] | 2020-07-06 - 2020-07-10 | Subnational | Moderate | 13,175 | Multiple groups | All | Sequential | Residual sera | 0.5% | Abbott Laboratories - IgG - Abbott Architect SARS-CoV-2 IgG,DiaSorin - IgG - Liaison SARS-CoV-2 S1/S2 IgG | 0.964, 1 | Complex test algorithm: Manual calculation |
| Canada (Ontario) | Shelly Bolotin, Vanessa Tran (Public Health Ontario)[145] | 2020-06-05 - 2020-06-30 | Subnational | Moderate | 7,014 | Multiple groups | All | Convenience | Residual sera | 1.1% | Abbott Laboratories - IgG - Abbott Architect SARS-CoV-2 IgG,Ortho Clinical Diagnostics Inc. - IgG - VITROS Immunodiagnostic Products Anti-SARS-CoV-2 IgG | 0.904, 1 | Complex test algorithm: Manual calculation |
| Canada (Alberta) | Carmen Charlton (Alberta Precision Laboratories)[144] | 2020-06-01 - 2020-06-05 | Subnational | Moderate | 8,442 | Multiple groups | All | Sequential | Residual sera | 0.9% | Abbott Laboratories - IgG - Abbott Architect SARS-CoV-2 IgG,DiaSorin - IgG - Liaison SARS-CoV-2 S1/S2 IgG | 0.964, 1 | Complex test algorithm: Manual calculation |
| Canada (Ontario) | Shelly Bolotin, Vanessa Tran (Public Health Ontario)[145] | 2020-05-26 - 2020-05-31 | Subnational | Moderate | 1,061 | Multiple groups | All | Convenience | Residual sera | 1.5% | Abbott Laboratories - IgG - Abbott Architect SARS-CoV-2 IgG,Ortho Clinical Diagnostics Inc. - IgG - VITROS Immunodiagnostic Products Anti-SARS-CoV-2 IgG | 0.904, 1 | Complex test algorithm: Manual calculation |
| Canada (British Columbia) | Danuta Skowronski (British Columbia Centre for Disease Control)[146] | 2020-05-15 - 2020-05-27 | Subnational | Moderate | 870 | Multiple groups | All | Probability | Residual sera | 0.8% | Ortho Clinical Diagnostics Inc. - IgG, IgM, IgA - VITROS Immunodiagnostic Products Anti-SARS-CoV-2 Total | 1, 1 | Validated by manufacturer |
| Canada (Ontario) | Shelly Bolotin, Vanessa Tran (Public Health Ontario)[145] | 2020-03-27 - 2020-04-30 | Subnational | Moderate | 827 | Multiple groups | All | Convenience | Residual sera | 0.5% | Abbott Laboratories - IgG - Abbott Architect SARS-CoV-2 IgG,Ortho Clinical Diagnostics Inc. - IgG - VITROS Immunodiagnostic Products Anti-SARS-CoV-2 IgG | 0.904, 1 | Complex test algorithm: Manual calculation |
| Canada (British Columbia) | Danuta Skowronski (British Columbia Centre for Disease Control)[146] | 2020-03-05 - 2020-03-13 | Subnational | Moderate | 870 | Multiple groups | All | Probability | Residual sera | 0.8% | Ortho Clinical Diagnostics Inc. - IgG, IgM, IgA - VITROS Immunodiagnostic Products Anti-SARS-CoV-2 Total | 1, 1 | Validated by manufacturer |
| Chile - HRP (Cities: Santiago (34 municipalities), Coquimbo-La serena (2 Municipalities) and Talca (1 Municipality)) | Ximena Aguilera (Universidad del Desarrollo, Centro de Epidemiología y Políticas de Salud)[147] | 2021-10-05 - 2021-11-25 | Subnational | Moderate | 2,198 | Multiple groups | All | Probability | Household and community samples | 97.3% (95% CI 96.6-98%) | Beijing Wantai Biological - IgG IgM IgA - Wantai Rapid Test for Total Antibody to SARS-CoV-2 | 0.947, 0.989 | Validated by manufacturer |
| United States of America (Texas) | The University of Texas Health Science Center at Houston (University of Texas Health Science Center at Houston)[148] | 2021-09-20 - 2021-10-04 | Subnational | Moderate | 88,091 | Multiple groups | All | Convenience | Household and community samples | 75.5% | Roche Diagnostics - IgG, IgM, IgA - Elecsys  Anti‐SARS‐CoV‐2 (S) | 0.988, 1 | Validated by manufacturer |
| United States of America (California) | Megha Mehrotra (California Department of Public Health)[149] | 2021-04-20 - 2021-08-01 | Subnational | Low | 7,483 | Multiple groups | All | Probability | Household and community samples | 67% | Enable Biosciences - IgG, IgM, IgA - Enable’s ADAP SARS-CoV-2 total antibody assay | 1, 1 | Validated by manufacturer |
| United States of America (California) | Megha Mehrotra (California Department of Public Health)[149] | 2021-04-20 - 2021-06-16 | Subnational | Low | 1,375 | Children and Youth (0-17 years) | All | Probability | Household and community samples | 41% | Enable Biosciences - IgG, IgM, IgA - Enable’s ADAP SARS-CoV-2 total antibody assay | 1, 1 | Validated by manufacturer |
| United States of America (California) | Alexia Couture (Centers for Disease Control and Prevention)[150] | 2021-05-01 - 2021-05-31 | Subnational | Moderate | 651 | Children and Youth (0-17 years) | All | Convenience | Residual sera | 43.7% | Roche Diagnostics - IgG, IgM, IgA - Elecsys  Anti‐SARS‐CoV‐2 (N) | 1, 0.995 | Validated by manufacturer |
| United States of America (Illinois) | Alexia Couture (Centers for Disease Control and Prevention)[150] | 2021-05-01 - 2021-05-31 | Subnational | Moderate | 942 | Children and Youth (0-17 years) | All | Convenience | Residual sera | 41.5% | Roche Diagnostics - IgG, IgM, IgA - Elecsys  Anti‐SARS‐CoV‐2 (N) | 1, 0.995 | Validated by manufacturer |
| United States of America (North Carolina) | Alexia Couture (Centers for Disease Control and Prevention)[150] | 2021-05-01 - 2021-05-31 | Subnational | Moderate | 648 | Children and Youth (0-17 years) | All | Convenience | Residual sera | 26.3% | Roche Diagnostics - IgG, IgM, IgA - Elecsys  Anti‐SARS‐CoV‐2 (N) | 1, 0.995 | Validated by manufacturer |
| United States of America (Ohio) | Alexia Couture (Centers for Disease Control and Prevention)[150] | 2021-05-01 - 2021-05-31 | Subnational | Moderate | 627 | Children and Youth (0-17 years) | All | Convenience | Residual sera | 37% | Roche Diagnostics - IgG, IgM, IgA - Elecsys  Anti‐SARS‐CoV‐2 (N) | 1, 0.995 | Validated by manufacturer |
| United States of America (South Carolina) | Alexia Couture (Centers for Disease Control and Prevention)[150] | 2021-05-01 - 2021-05-31 | Subnational | Moderate | 618 | Children and Youth (0-17 years) | All | Convenience | Residual sera | 34.8% | Roche Diagnostics - IgG, IgM, IgA - Elecsys  Anti‐SARS‐CoV‐2 (N) | 1, 0.995 | Validated by manufacturer |
| United States of America (California) | Marcus Wong (University of California)[151] | 2021-02-15 - 2021-04-15 | Subnational | Moderate | 4,641 | Multiple groups | All | Convenience | Household and community samples | 31.3% | Ortho Clinical Diagnostics Inc. - IgG, IgM, IgA - VITROS Immunodiagnostic Products Anti-SARS-CoV-2 Total,NA - - Author designed (ELISA) -Spike | N/A | Complex test algorithm: Manual review |
| United States of America (Arkansas) | Joshua Kennedy (University of Arkansas for Medical Sciences)[152] | 2020-11-07 - 2020-12-19 | Subnational | Moderate | 2,405 | Multiple groups | All | Convenience | Residual sera | 7.4% | Beckman Coulter - IgG - Access SARS-CoV-2 IgG Antibody Test,NA - - Author designed (ELISA) - Unknown | N/A | Complex test algorithm: Manual review |
| United States of America (Arkansas) | Victor M. Cardenas (University of Arkansas for Medical Sciences)[153] | 2020-11-01 - 2020-12-15 | Subnational | Moderate | 342 | Multiple groups | All | Probability | Household and community samples | 12.9% | Beckman Coulter - IgG - Access SARS-CoV-2 IgG Antibody Test | 1, 0.998 | Validated by manufacturer |
| United States of America (California) | Alice Pressman (Sutter Health)[154] | 2020-10-01 - 2020-12-31 | Subnational | Moderate | 4,500 | Adults (18-64 years) | Female | Convenience | Pregnant or parturient women | 8.1% | Siemens - IgG, IgM, IgA - Atellica  IM SARS-CoV-2 Total (COV2T) | 0.985, 0.998 | Validated by manufacturer |
| United States of America (California) | Marcus Wong (University of California)[151] | 2020-10-15 - 2020-12-15 | Subnational | Moderate | 5,308 | Multiple groups | All | Convenience | Household and community samples | 0.6% | Ortho Clinical Diagnostics Inc. - IgG, IgM, IgA - VITROS Immunodiagnostic Products Anti-SARS-CoV-2 Total | N/A | Complex test algorithm: Manual review |
| United States of America (Arkansas) | Joshua Kennedy (University of Arkansas for Medical Sciences)[152] | 2020-09-12 - 2020-10-24 | Subnational | Moderate | 2,098 | Multiple groups | All | Convenience | Residual sera | 4.1% | Beckman Coulter - IgG - Access SARS-CoV-2 IgG Antibody Test,NA - - Author designed (ELISA) - Unknown | N/A | Complex test algorithm: Manual review |
| United States of America (Virginia) | Rebecca Levorson (Inova Health System)[155] | 2020-07-31 - 2020-10-13 | Subnational | Moderate | 1,038 | Children and Youth (0-17 years) | All | Convenience | Multiple general populations | 8.5% | Ortho Clinical Diagnostics Inc. - IgG - VITROS Immunodiagnostic Products Anti-SARS-CoV-2 IgG | 1, 1 | Validated by manufacturer |
| United States of America (North Carolina) | Cesar Lopez (University of North Carolina)[156] | 2020-08-09 - 2020-10-03 | Subnational | Moderate | 2,633 | Multiple groups | All | Convenience | Residual sera | 10.8% | NA - IgG - Author designed (ELISA) -Spike | 0.98, 1 | Validated by developers |
| United States of America (Arkansas) | Joshua Kennedy (University of Arkansas for Medical Sciences)[152] | 2020-08-15 - 2020-09-05 | Subnational | Moderate | 1,301 | Multiple groups | All | Convenience | Residual sera | 2.6% | Beckman Coulter - IgG - Access SARS-CoV-2 IgG Antibody Test,NA - - Author designed (ELISA) - Unknown | N/A | Complex test algorithm: Manual review |
| United States of America (California) | Marcus Wong (University of California)[151] | 2020-07-15 - 2020-09-15 | Subnational | Moderate | 4,670 | Multiple groups | All | Convenience | Household and community samples | 0.6% | Ortho Clinical Diagnostics Inc. - IgG, IgM, IgA - VITROS Immunodiagnostic Products Anti-SARS-CoV-2 Total | 1, 1 | Validated by manufacturer |
| United States of America (California) | Cameron Adams (University of California, Berkeley)[157] | 2020-07-04 - 2020-09-01 | Subnational | Moderate | 4,760 | Multiple groups | All | Stratified non-probability | Household and community samples | 1% | Ortho Clinical Diagnostics Inc. - IgG, IgM, IgA - VITROS Immunodiagnostic Products Anti-SARS-CoV-2 Total | 1, 1 | Validated by manufacturer |
| United States of America (Colorado) | Kiersten Kugeler (Centers for Disease Control and Prevention)[158] | 2020-07-22 - 2020-08-08 | Subnational | Moderate | 1,598 | Multiple groups | All | Convenience | Residual sera | 6.8% | Ortho Clinical Diagnostics Inc. - IgG, IgM, IgA - VITROS Immunodiagnostic Products Anti-SARS-CoV-2 Total | 1, 1 | Validated by manufacturer |
| United States of America (Colorado) | Kiersten Kugeler (Centers for Disease Control and Prevention)[158] | 2020-07-22 - 2020-08-08 | Subnational | Moderate | 381 | Multiple groups | All | Probability | Household and community samples | 8% | Ortho Clinical Diagnostics Inc. - IgG, IgM, IgA - VITROS Immunodiagnostic Products Anti-SARS-CoV-2 Total | 1, 1 | Validated by manufacturer |
| United States of America (North Carolina) | Cesar Lopez (University of North Carolina)[156] | 2020-06-14 - 2020-08-08 | Subnational | Moderate | 3,528 | Multiple groups | All | Convenience | Residual sera | 10.5% | NA - IgG - Author designed (ELISA) -Spike | 0.98, 1 | Validated by developers |
| United States of America (Virginia) | Elizabeth T Rogawski McQuade (University of Virginia )[159] | 2020-06-01 - 2020-08-14 | Subnational | Moderate | 4,675 | Multiple groups | All | Convenience | Residual sera | 2.2% | Abbott Laboratories - IgG - Abbott Architect SARS-CoV-2 IgG | 0.99, 1 | Validated by manufacturer |
| United States of America (Connecticut) | Shiwani Mahajan (Yale School of Medicine)[160] | 2020-06-23 - 2020-07-22 | Subnational | Moderate | 148 | Multiple groups | All | Probability | Household and community samples | 6.4% | Ortho Clinical Diagnostics Inc. - IgG - VITROS Immunodiagnostic Products Anti-SARS-CoV-2 IgG | 1, 1 | Validated by manufacturer |
| United States of America (Connecticut) | Shiwani Mahajan (Yale School of Medicine)[160] | 2020-06-23 - 2020-07-22 | Subnational | Moderate | 171 | Multiple groups | All | Probability | Household and community samples | 19.9% | Ortho Clinical Diagnostics Inc. - IgG - VITROS Immunodiagnostic Products Anti-SARS-CoV-2 IgG | 1, 1 | Validated by manufacturer |
| United States of America (New York) | Lylach Haizler Cohen (Donald and Barbara Zucker School of Medicine)[161] | 2020-05-27 - 2020-07-24 | Subnational | Moderate | 1,671 | Adults (18-64 years) | Female | Convenience | Pregnant or parturient women | 16.1% | Roche Diagnostics - IgG, IgM, IgA - Elecsys  Anti‐SARS‐CoV‐2 (N) | 1, 0.995 | Validated by manufacturer |
| United States of America (Connecticut) | Shiwani Mahajan (Yale School of Medicine)[160] | 2020-06-04 - 2020-06-23 | Subnational | Moderate | 567 | Multiple groups | All | Probability | Household and community samples | 4% | Ortho Clinical Diagnostics Inc. - IgG - VITROS Immunodiagnostic Products Anti-SARS-CoV-2 IgG | 1, 1 | Validated by manufacturer |
| United States of America (Connecticut) | Travis Lim (Centers for Disease and Control )[162] | 2020-03-23 - 2020-08-14 | Subnational | Low | 8,929 | Multiple groups | All | Probability | Residual sera | 5.7% | NA - IgG, IgM, IgA - Author designed (ELISA) -Spike | 0.96, 0.993 | Validated by developers |
| United States of America (Florida) | Travis Lim (Centers for Disease and Control )[162] | 2020-03-23 - 2020-08-14 | Subnational | Low | 7,787 | Multiple groups | All | Probability | Residual sera | 4.8% | NA - IgG, IgM, IgA - Author designed (ELISA) -Spike | 0.96, 0.993 | Validated by developers |
| United States of America (Louisiana) | Travis Lim (Centers for Disease and Control )[162] | 2020-03-23 - 2020-08-14 | Subnational | Low | 2,849 | Multiple groups | All | Probability | Residual sera | 7.7% | NA - IgG, IgM, IgA - Author designed (ELISA) -Spike | 0.96, 0.993 | Validated by developers |
| United States of America (Minnesota) | Travis Lim (Centers for Disease and Control )[162] | 2020-03-23 - 2020-08-14 | Subnational | Low | 7,944 | Multiple groups | All | Probability | Residual sera | 4.9% | NA - IgG, IgM, IgA - Author designed (ELISA) -Spike | 0.96, 0.993 | Validated by developers |
| United States of America (Missouri) | Travis Lim (Centers for Disease and Control )[162] | 2020-03-23 - 2020-08-14 | Subnational | Low | 9,356 | Multiple groups | All | Probability | Residual sera | 2.5% | NA - IgG, IgM, IgA - Author designed (ELISA) -Spike | 0.96, 0.993 | Validated by developers |
| United States of America (Utah) | Travis Lim (Centers for Disease and Control )[162] | 2020-03-23 - 2020-08-14 | Subnational | Low | 8,769 | Multiple groups | All | Probability | Residual sera | 2.7% | NA - IgG, IgM, IgA - Author designed (ELISA) -Spike | 0.96, 0.993 | Validated by developers |
| United States of America (Washington) | Travis Lim (Centers for Disease and Control )[162] | 2020-03-23 - 2020-08-14 | Subnational | Low | 7,544 | Multiple groups | All | Probability | Residual sera | 2% | NA - IgG, IgM, IgA - Author designed (ELISA) -Spike | 0.96, 0.993 | Validated by developers |
| United States of America (Utah) | Matthew Samore (University of Utah)[163] | 2020-05-04 - 2020-06-30 | Subnational | Low | 8,108 | Multiple groups | All | Probability | Household and community samples | 1.1% | Abbott Laboratories - IgG - Abbott Architect SARS-CoV-2 IgG | 0.99, 1 | Validated by manufacturer |
| United States of America (Oregon) | Melissa Sutton (Oregon Health Authority)[164] | 2020-05-11 - 2020-06-15 | Subnational | Moderate | 897 | Multiple groups | All | Probability | Residual sera | 1% | Abbott Laboratories - IgG - Abbott Architect SARS-CoV-2 IgG | 0.99, 1 | Validated by manufacturer |
| United States of America (North Carolina) | Cesar Lopez (University of North Carolina)[156] | 2020-04-19 - 2020-06-13 | Subnational | Moderate | 3,463 | Multiple groups | All | Convenience | Residual sera | 5.3% | NA - IgG - Author designed (ELISA) -Spike | 0.98, 1 | Validated by developers |
| United States of America (Rhode Island) | Philip A Chan (Brown University)[165] | 2020-05-05 - 2020-05-22 | Subnational | Low | 1,032 | Multiple groups | All | Probability | Household and community samples | 2.9% | Abbott Laboratories - IgG - Abbott Architect SARS-CoV-2 IgG | 0.99, 1 | Validated by manufacturer |
| United States of America (Indiana) | Nir Menachemi (Indiana University Fairbanks School of Public Health)[166] | 2020-04-25 - 2020-04-29 | Subnational | Low | 3,518 | Multiple groups | All | Probability | Household and community samples | 1.1% | NA - IgG - Author designed (ELISA) -Unknown | 1, 0.996 |  |

#### Eastern Mediterranean region

| Country (Location) | Author (Organization) | Sampling Dates (YMD) | Geographic scope | Overall risk of bias | Sample size | Age | Sex | Sampling method | Sample frame | Seroprevalence (95% CI) | Test Manufacturers - Isotypes - Names | Test Sens, Spec | Sens, Spec Source |
| --- | --- | --- | --- | --- | --- | --- | --- | --- | --- | --- | --- | --- | --- |
| Iran (Islamic Republic of) - HRP (Guilan) | Maryam Shakiba (Tehran University of Medical Sciences)[167] | 2020-04-11 - 2020-04-19 | Subnational | Moderate | 528 | Multiple groups | All | Probability | Household and community samples | 22.1% | VivaChek Biotech (Hangzhou) Co. Ltd - IgG, IgM - VivaDiag COVID-19 IgM/IgG Rapid Test | 0.971, 1 | Validated by manufacturer |
| Iran (Islamic Republic of) - HRP (Mazandaran) | Seyed Abbas Mousavi (Mazandaran University of Medical Sciences)[168] | 2020-03-15 - 2020-05-15 | Subnational | Low | 1,588 | Multiple groups | All | Probability | Household and community samples | 17.3% | Pishtaz Diagnostics Iran - IgG, IgM - ELISA kit Pishtaz Teb Diagnostics | 0.941, 0.983 | Validated by manufacturer |
| occupied Palestinian territory, including east Jerusalem - HRP (West Bank) | Beesan Maraqa (An-Najah National University)[169] | 2020-11-01 - 2020-12-31 | Subnational | Moderate | 1,269 | Multiple groups | All | Probability | Residual sera | 24% | Roche Diagnostics - IgG, IgM, IgA - Elecsys  Anti‐SARS‐CoV‐2 (N) | 1, 0.995 | Validated by manufacturer |
| United Arab Emirates (Emirate of Abu Dhabi) | Ahmed R Alsuwaidi (Abu Dhabi Public Health Center)[170] | 2020-07-19 - 2020-08-14 | Subnational | Moderate | 8,831 | Multiple groups | All | Probability | Household and community samples | 11.3% | DiaSorin - IgG - Liaison SARS-CoV-2 S1/S2 IgG,Roche Diagnostics - IgG, IgM, IgA - Elecsys  Anti‐SARS‐CoV‐2 (N) | 0.974, 1 | Complex test algorithm: Manual calculation |

#### Europe region

| Country (Location) | Author (Organization) | Sampling Dates (YMD) | Geographic scope | Overall risk of bias | Sample size | Age | Sex | Sampling method | Sample frame | Seroprevalence (95% CI) | Test Manufacturers - Isotypes - Names | Test Sens, Spec | Sens, Spec Source |
| --- | --- | --- | --- | --- | --- | --- | --- | --- | --- | --- | --- | --- | --- |
| Bosnia and Herzegovina (Republic of Srpska) | Biljana Mijovic (University of Banja Luka)[171] | 2020-12-01 - 2021-01-15 | Subnational | Moderate | 1,855 | Multiple groups | All | Probability | Household and community samples | 40.3% | Beijing Wantai Biological - IgG, IgM, IgA - Wantai SARS-CoV-2 Total Ab ELISA | 0.967, 0.975 | Validated by manufacturer |
| Bosnia and Herzegovina (District Sarajevo) | Sanjin Musa (Institute for Public Health of the Federation of Bosnia and Herzegovina)[172] | 2020-11-02 - 2020-12-07 | Subnational | Moderate | 1,015 | Multiple groups | All | Probability | Blood donors | 19.1% (95% CI 16.6-21.6%) | Abbott Laboratories - IgG - Abbott Architect SARS-CoV-2 IgG,Abbott Laboratories - IgG - SARS-CoV-2 IgG II Quant | 0.984, 1 | Complex test algorithm: Manual calculation |
| Georgia (4 Districts:4 highly touristic districts of Georgia (Borjomi, Martvili, Khelvachauri and Kobuleti)) | Khatuna Zakhashvili (National Center for Disease Control and Public Health)[173] | 2020-12-01 - 2020-12-14 | Subnational | Moderate | 1,219 | Multiple groups | All | Probability | Household and community samples | 44.1% (95% CI 41.2-46.9%) | Beijing Wantai Biological - IgG IgM IgA - Wantai Rapid Test for Total Antibody to SARS-CoV-2 | 0.947, 0.989 | Validated by manufacturer |
| Georgia (4 Districts:4 highly touristic districts of Georgia (Borjomi, Martvili, Khelvachauri and Kobuleti)) | Khatuna Zakhashvili (National Center for Disease Control and Public Health)[173] | 2020-08-01 - 2020-08-14 | Subnational | Moderate | 1,218 | Multiple groups | All | Probability | Household and community samples | 0.5% (95% CI 0.2-1.1%) | Beijing Wantai Biological - IgG IgM IgA - Wantai Rapid Test for Total Antibody to SARS-CoV-2 | 0.947, 0.989 | Validated by manufacturer |
| Denmark (Capital Region and Region Zealand) | Maren J H Rytter (Slagelse Sygehus)[174] | 2020-06-22 - 2020-07-03 | Subnational | Moderate | 750 | Adults (18-64 years) | All | Probability | Blood donors | 2% | Zhuhai Livzon Diagnostics Inc - IgG, IgM - 2019-nCoV IgG/IgM Antibody Detection Kit | 0.906, 0.992 | Validated by manufacturer |
| Denmark (Capital Region of Denmark) | Kasper Iversen (University of Copenhagen)[175] | 2020-04-15 - 2020-04-22 | Subnational | Moderate | 4,672 | Adults (18-64 years) | All | Sequential | Blood donors | 3% | Zhuhai Livzon Diagnostics Inc - IgG, IgM - 2019-nCoV IgG/IgM Antibody Detection Kit | 0.906, 0.992 | Validated by manufacturer |
| Faroe Islands (Faroe Islands) | Maria Skaalum Petersen (University of the Faroe Islands)[176] | 2020-04-27 - 2020-05-01 | Subnational | Moderate | 1,075 | Adults (18-64 years) | All | Probability | Household and community samples | 0.6% | Beijing Wantai Biological - IgG, IgM, IgA - Wantai SARS-CoV-2 Total Ab ELISA | 0.967, 0.975 | Validated by manufacturer |
| France (Corsica) | Lisandru Capai (Université de Corse)[177] | 2021-02-01 - 2021-02-12 | Subnational | Moderate | 1,254 | Multiple groups | All | Convenience | Residual sera | 8.5% | EUROIMMUN - IgG - Anti-SARS-CoV-2 ELISA IgG | 0.9, 1 | Validated by manufacturer |
| Germany (North Rhine-Westphalia) | Insa Backhaus (Heinrich Heine University)[178] | 2020-11-02 - 2020-11-27 | Subnational | Moderate | 2,186 | Adults (18-64 years) | All | Probability | Household and community samples | 3.3% | Roche Diagnostics - IgG, IgM, IgA - Elecsys  Anti‐SARS‐CoV‐2 (N) | 1, 0.995 | Validated by manufacturer |
| Germany (Bavaria) | Otto Laub (University of Regensburg)[179] | 2020-05-22 - 2020-07-22 | Subnational | Moderate | 2,832 | Children and Youth (0-17 years) | All | Probability | Multiple general populations | 4.8% | NA - IgG - Author designed (ELISA) -Spike,Roche Diagnostics - IgG, IgM, IgA - Elecsys  Anti‐SARS‐CoV‐2 (N),NA - - Author designed (Neutralization Assay) | N/A | Complex test algorithm: Manual review |
| Germany (North Rhine-Westphalia, Lower Saxony, Hesse) | Bastian Fischer (Heart and Diabetes Center NRW)[180] | 2020-03-09 - 2020-06-03 | Subnational | Moderate | 3,186 | Multiple groups | All | Sequential | Residual sera | 0.9% | Abbott Laboratories - IgG - Abbott Architect SARS-CoV-2 IgG,EUROIMMUN - IgG - Anti-SARS-CoV-2 ELISA IgG,DiaSorin - IgG - Liaison SARS-CoV-2 S1/S2 IgG | N/A | Complex test algorithm: Manual review |
| Italy (Campania (Southern Italy)) | Federica Calo (University of Campania)[181] | 2020-06-01 - 2020-06-10 | Subnational | Moderate | 143 | Multiple groups | All | Sequential | Blood donors | 2.8% | Beijing Wantai Biological - IgG, IgM, IgA - Wantai SARS-CoV-2 Total Ab ELISA | 0.967, 0.975 | Validated by manufacturer |
| Italy (Trento) | Paola Stefanelli (Azienda Provinciale per i Servizi Sanitari)[182] | 2020-05-05 - 2020-05-15 | Subnational | Moderate | 6,075 | Multiple groups | All | Convenience | Household and community samples | 23.1% | Abbott Laboratories - IgG - Abbott Architect SARS-CoV-2 IgG | 0.99, 1 | Validated by manufacturer |
| Jersey (Jersey) | Statistics Jersey (Government of Jersey)[183] | 2020-04-29 - 2020-05-05 | Subnational | Low | 855 | Multiple groups | All | Probability | Household and community samples | 2.6% | Healgen - IgG, IgM - COVID-19 IgG/IgM Rapid Test Cassette | 0.938, 0.985 | Validated by manufacturer |
| Jersey (Jersey) | Statistics Jersey (Statistics Jersey)[184] | 2020-05-15 - 2020-05-27 | Subnational | Low | 1,062 | Multiple groups | All | Probability | Household and community samples | 4.4% | CTK Biotech, Inc. - IgG, IgM - OnSite COVID-19 IgG/IgM Rapid Test | 0.969, 0.994 | Validated by manufacturer |
| Spain (Catalonia) | Marianna Karachaliou (Barcelona Institute For Global Health)[185] | 2020-06-30 - 2020-11-15 | Subnational | Moderate | 4,740 | Multiple groups | All | Convenience | Household and community samples | 15.3% | NA - - Author designed (Multiplex) | N/A | Complex test algorithm: Manual review |
| Spain (Cantabria) | Paula Iruzubieta (University of Cantabria)[186] | 2020-04-27 - 2020-05-29 | Subnational | Moderate | 1,022 | Multiple groups | All | Probability | Household and community samples | 3.1% | Hangzhou Clongene Biotech Co. Ltd - IgG, IgM - Lungene 2019-nCoV IgG/IgM Rapid Test | 0.911, 0.965 | Validated by manufacturer |
| Switzerland (Ticino) | Ottavio Beretta (Ufficio del medico cantonale)[187] | 2021-05-03 - 2021-06-13 | Subnational | Moderate | 722 | Multiple groups | All | Probability | Household and community samples | 22.4% | Technogenetics - IgG, IgM - Rapid Test SARS-CoV-2 IgM/IgG Gold | 0.951, 0.989 | Validated by manufacturer |
| Switzerland (Canton of Fribourg) | Emna El May (Université de Fribourg)[188] | 2020-11-30 - 2021-02-05 | Subnational | Moderate | 449 | Multiple groups | All | Probability | Household and community samples | 19% | NA - - Author designed (Luminex) | 0.966, 0.997 | Validated by developers |
| Switzerland (Ticino) | Ottavio Beretta (Ufficio del medico cantonale)[187] | 2020-11-02 - 2020-12-27 | Subnational | Moderate | 870 | Multiple groups | All | Probability | Household and community samples | 14.4% | Technogenetics - IgG, IgM - Rapid Test SARS-CoV-2 IgM/IgG Gold | 0.951, 0.989 | Validated by manufacturer |
| Switzerland (Canton of Fribourg) | Daniela Anker (Université de Fribourg)[189] | 2020-07-08 - 2020-10-14 | Subnational | Moderate | 418 | Multiple groups | All | Probability | Household and community samples | 8% | NA - - Author designed (Luminex) | 0.966, 0.997 | Validated by developers |
| Switzerland (Ticino) | Ottavio Beretta (Ufficio del medico cantonale)[187] | 2020-08-03 - 2020-08-03 | Subnational | Moderate | 905 | Multiple groups | All | Probability | Household and community samples | 8.9% | Technogenetics - IgG, IgM - Rapid Test SARS-CoV-2 IgM/IgG Gold | 0.951, 0.989 | Validated by manufacturer |
| Switzerland (Canton of Zurich) | Erin West (University of Zurich)[190] | 2020-06-17 - 2020-09-14 | Subnational | Moderate | 472 | Adults (18-64 years) | All | Probability | Household and community samples | 4% | Swiss Federal Institute of Technology - IgG, IgA - Sensitive Anti-SARS-CoV-2 Spike Trimer Immunoglobulin Serological (SenASTrIS) | 0.966, 0.997 | Validated by manufacturers |
| Switzerland (Canton of Geneva) | Aude Richard (Geneva University Hospitals)[191] | 2020-06-01 - 2020-06-30 | Subnational | Low | 3,407 | Multiple groups | All | Probability | Household and community samples | 6.3% | EUROIMMUN - IgG - Anti-SARS-CoV-2 ELISA IgG | 0.9, 1 | Validated by manufacturer |
| Switzerland (Ticino) | Ottavio Beretta (Ufficio del medico cantonale)[187] | 2020-05-11 - 2020-06-28 | Subnational | Moderate | 934 | Multiple groups | All | Probability | Household and community samples | 9.6% | Technogenetics - IgG, IgM - Rapid Test SARS-CoV-2 IgM/IgG Gold | 0.951, 0.989 | Validated by manufacturer |
| Switzerland (Vaud) | Craig Fenwick (Lausanne University Hospital and University of Lausanne)[192] | 2020-05-04 - 2020-06-27 | Subnational | Moderate | 311 | Multiple groups | All | Probability | Household and community samples | 3.9% | Roche Diagnostics - IgG, IgM, IgA - Elecsys  Anti‐SARS‐CoV‐2 (N) | 1, 0.995 | Validated by manufacturer |
| Switzerland (Geneva) | Silvia Stringhini (Geneva University Hospitals)[193] | 2020-05-04 - 2020-05-09 | Subnational | Moderate | 775 | Multiple groups | All | Probability | Household and community samples | 10.8% | EUROIMMUN - IgG - Anti-SARS-CoV-2 ELISA IgG | 0.9, 1 | Validated by manufacturer |
| The United Kingdom (England) | Office for National Statistics (Office For National Statistics)[194] | 2021-02-04 - 2021-03-03 | Subnational | Low | 34,623 | Multiple groups | All | Probability | Household and community samples | 34.6% | NA - IgG - Author designed (ELISA) -Spike | 0.99, 0.991 | Validated by independent authors/third party/non-developers |
| The United Kingdom (Northern Ireland) | Office for National Statistics (Office For National Statistics)[194] | 2021-02-04 - 2021-03-03 | Subnational | Low | 632 | Multiple groups | All | Probability | Household and community samples | 31.2% | NA - IgG - Author designed (ELISA) -Spike | 0.99, 0.991 | Validated by independent authors/third party/non-developers |
| The United Kingdom (Scotland) | Office for National Statistics (Office For National Statistics)[194] | 2021-02-04 - 2021-03-03 | Subnational | Low | 2,737 | Multiple groups | All | Probability | Household and community samples | 22.3% | NA - IgG - Author designed (ELISA) -Spike | 0.99, 0.991 | Validated by independent authors/third party/non-developers |
| The United Kingdom (Wales) | Office for National Statistics (Office For National Statistics)[194] | 2021-02-04 - 2021-03-03 | Subnational | Low | 1,224 | Multiple groups | All | Probability | Household and community samples | 30.5% | NA - IgG - Author designed (ELISA) -Spike | 0.99, 0.991 | Validated by independent authors/third party/non-developers |
| The United Kingdom (England) | Office for National Statistics (Office of National Statistics)[195] | 2020-12-18 - 2021-01-14 | Subnational | Moderate | 24,150 | Multiple groups | All | Probability | Household and community samples | 15.3% | NA - IgG - Author designed (ELISA) -Spike | 0.99, 0.991 | Validated by independent authors/third party/non-developers |
| The United Kingdom (Northern Ireland) | Office for National Statistics (Office of National Statistics)[195] | 2020-12-18 - 2021-01-14 | Subnational | Moderate | 313 | Multiple groups | All | Probability | Household and community samples | 12% | NA - IgG - Author designed (ELISA) -Spike | 0.99, 0.991 | Validated by independent authors/third party/non-developers |
| The United Kingdom (Scotland) | Office for National Statistics (Office of National Statistics)[195] | 2020-12-18 - 2021-01-14 | Subnational | Moderate | 1,832 | Multiple groups | All | Probability | Household and community samples | 10.7% | NA - IgG - Author designed (ELISA) -Spike | 0.99, 0.991 | Validated by independent authors/third party/non-developers |
| The United Kingdom (Wales) | Office for National Statistics (Office of National Statistics)[195] | 2020-12-18 - 2021-01-14 | Subnational | Moderate | 732 | Multiple groups | All | Probability | Household and community samples | 11.5% | NA - IgG - Author designed (ELISA) -Spike | 0.99, 0.991 | Validated by independent authors/third party/non-developers |
| The United Kingdom (England) | Office for National Statistics (Office of National Statistics)[195] | 2020-11-20 - 2020-12-17 | Subnational | Moderate | 21,684 | Multiple groups | All | Probability | Household and community samples | 10.1% | NA - IgG - Author designed (ELISA) -Spike | 0.99, 0.991 | Validated by independent authors/third party/non-developers |
| The United Kingdom (Northern Ireland) | Office for National Statistics (Office of National Statistics)[195] | 2020-11-20 - 2020-12-17 | Subnational | Moderate | 248 | Multiple groups | All | Probability | Household and community samples | 3.7% | NA - IgG - Author designed (ELISA) -Spike | 0.99, 0.991 | Validated by independent authors/third party/non-developers |
| The United Kingdom (Scotland) | Office for National Statistics (Office of National Statistics)[195] | 2020-11-20 - 2020-12-17 | Subnational | Moderate | 1,034 | Multiple groups | All | Probability | Household and community samples | 5.9% | NA - IgG - Author designed (ELISA) -Spike | 0.99, 0.991 | Validated by independent authors/third party/non-developers |
| The United Kingdom (Wales) | Office for National Statistics (Office of National Statistics)[195] | 2020-11-20 - 2020-12-17 | Subnational | Moderate | 631 | Multiple groups | All | Probability | Household and community samples | 7.2% | NA - IgG - Author designed (ELISA) -Spike | 0.99, 0.991 | Validated by independent authors/third party/non-developers |
| The United Kingdom (Scotland) | Office for National Statistics (Office of National Statistics)[195] | 2020-10-23 - 2020-11-19 | Subnational | Moderate | 397 | Multiple groups | All | Probability | Household and community samples | 7.8% | NA - IgG - Author designed (ELISA) -Spike | 0.99, 0.991 | Validated by independent authors/third party/non-developers |
| The United Kingdom (Northern Ireland) | Office for National Statistics (Office of National Statistics)[195] | 2020-10-23 - 2020-11-19 | Subnational | Moderate | 333 | Multiple groups | All | Probability | Household and community samples | 3.5% | NA - IgG - Author designed (ELISA) -Spike | 0.99, 0.991 | Validated by independent authors/third party/non-developers |
| The United Kingdom (Wales) | Office for National Statistics (Office of National Statistics)[195] | 2020-10-23 - 2020-11-19 | Subnational | Moderate | 497 | Multiple groups | All | Probability | Household and community samples | 6.1% | NA - IgG - Author designed (ELISA) -Spike | 0.99, 0.991 | Validated by independent authors/third party/non-developers |
| The United Kingdom (England) | Office for National Statistics (Office of National Statistics)[195] | 2020-10-23 - 2020-11-19 | Subnational | Moderate | 15,834 | Multiple groups | All | Probability | Household and community samples | 8.7% | NA - IgG - Author designed (ELISA) -Spike | 0.99, 0.991 | Validated by independent authors/third party/non-developers |
| The United Kingdom (England) | Office for National Statistics (Office of National Statistics)[195] | 2020-09-25 - 2020-10-22 | Subnational | Moderate | 15,687 | Multiple groups | All | Probability | Household and community samples | 6.9% | NA - IgG - Author designed (ELISA) -Spike | 0.99, 0.991 | Validated by independent authors/third party/non-developers |
| The United Kingdom (Northern Ireland) | Office for National Statistics (Office of National Statistics)[195] | 2020-09-25 - 2020-10-22 | Subnational | Moderate | 249 | Multiple groups | All | Probability | Household and community samples | 2.8% | NA - IgG - Author designed (ELISA) -Spike | 0.99, 0.991 | Validated by independent authors/third party/non-developers |
| The United Kingdom (Scotland) | Office for National Statistics (Office of National Statistics)[195] | 2020-09-25 - 2020-10-22 | Subnational | Moderate | 383 | Multiple groups | All | Probability | Household and community samples | 6.8% | NA - IgG - Author designed (ELISA) -Spike | 0.99, 0.991 | Validated by independent authors/third party/non-developers |
| The United Kingdom (Wales) | Office for National Statistics (Office of National Statistics)[195] | 2020-09-25 - 2020-10-22 | Subnational | Moderate | 473 | Multiple groups | All | Probability | Household and community samples | 4.7% | NA - IgG - Author designed (ELISA) -Spike | 0.99, 0.991 | Validated by independent authors/third party/non-developers |
| The United Kingdom (Wales) | Office for National Statistics (Office of National Statistics)[195] | 2020-08-28 - 2020-09-24 | Subnational | Moderate | 236 | Multiple groups | All | Probability | Household and community samples | 4.7% | NA - IgG - Author designed (ELISA) -Spike | 0.99, 0.991 | Validated by independent authors/third party/non-developers |
| The United Kingdom (England) | Office for National Statistics (Office of National Statistics)[195] | 2020-08-28 - 2020-09-24 | Subnational | Moderate | 8,742 | Multiple groups | All | Probability | Household and community samples | 5.8% | NA - IgG - Author designed (ELISA) -Spike | 0.99, 0.991 | Validated by independent authors/third party/non-developers |
| The United Kingdom (England) | Office for National Statistics (Office of National Statistics)[195] | 2020-07-31 - 2020-08-27 | Subnational | Moderate | 6,189 | Multiple groups | All | Probability | Household and community samples | 5.6% | NA - IgG - Author designed (ELISA) -Spike | 0.99, 0.991 | Validated by independent authors/third party/non-developers |
| The United Kingdom (England) | Office for National Statistics (Office of National Statistics)[195] | 2020-07-03 - 2020-07-30 | Subnational | Moderate | 3,236 | Multiple groups | All | Probability | Household and community samples | 6.5% | NA - IgG - Author designed (ELISA) -Spike | 0.99, 0.991 | Validated by independent authors/third party/non-developers |
| The United Kingdom (England) | Office for National Statistics (Office of National Statistics)[195] | 2020-06-05 - 2020-07-02 | Subnational | Moderate | 2,392 | Multiple groups | All | Probability | Household and community samples | 4.2% | NA - - Author designed (ELISA) -Spike | 0.99, 0.991 | Validated by independent authors/third party/non-developers |
| The United Kingdom (England) | Office for National Statistics (Office of National Statistics)[195] | 2020-05-08 - 2020-06-04 | Subnational | Low | 1,575 | Multiple groups | All | Probability | Household and community samples | 6.3% | NA - IgG - Author designed (ELISA) -Spike | 0.99, 0.991 | Validated by independent authors/third party/non-developers |
| The United Kingdom (England) | Public Health England (Public Health England)[111] | 2020-04-24 - 2020-04-26 | Subnational | Low | 865 | Adults (18-64 years) | All | Probability | Blood donors | 5% | EUROIMMUN - IgG - Anti-SARS-CoV-2 ELISA IgG | 0.9, 1 | Validated by manufacturer |
| The United Kingdom (England) | Public Health England (Public Health England)[111] | 2020-04-23 - 2020-04-24 | Subnational | Low | 1,043 | Adults (18-64 years) | All | Probability | Blood donors | 7.3% | EUROIMMUN - IgG - Anti-SARS-CoV-2 ELISA IgG | 0.9, 1 | Validated by manufacturer |
| The United Kingdom (England) | Public Health England (Public Health England)[111] | 2020-04-15 - 2020-04-20 | Subnational | Low | 936 | Adults (18-64 years) | All | Probability | Blood donors | 6.2% | EUROIMMUN - IgG - Anti-SARS-CoV-2 ELISA IgG | 0.9, 1 | Validated by manufacturer |
| The United Kingdom (Scotland) | Craig P Thompson (University of Oxford)[196] | 2020-03-17 - 2020-05-18 | Subnational | Moderate | 3,500 | Adults (18-64 years) | All | Probability | Blood donors | 3.2% | NA - - Author designed (Neutralization Assay) | 0.94, 1 | Validated by developers |
| The United Kingdom (England) | Public Health England (Public Health England)[111] | 2020-04-14 - 2020-04-16 | Subnational | Low | 1,017 | Adults (18-64 years) | All | Probability | Blood donors | 4.6% | EUROIMMUN - IgG - Anti-SARS-CoV-2 ELISA IgG | 0.9, 1 | Validated by manufacturer |
| The United Kingdom (England) | Public Health England (Public Health England)[111] | 2020-04-09 - 2020-04-13 | Subnational | Low | 1,085 | Adults (18-64 years) | All | Probability | Blood donors | 11.1% | EUROIMMUN - IgG - Anti-SARS-CoV-2 ELISA IgG | 0.9, 1 | Validated by manufacturer |
| The United Kingdom (England) | Public Health England (Public Health England)[111] | 2020-04-02 - 2020-04-03 | Subnational | Low | 916 | Adults (18-64 years) | All | Probability | Blood donors | 2.4% | EUROIMMUN - IgG - Anti-SARS-CoV-2 ELISA IgG | 0.9, 1 | Validated by manufacturer |
| The United Kingdom (England) | Public Health England (Public Health England)[111] | 2020-03-26 - 2020-03-27 | Subnational | Low | 757 | Adults (18-64 years) | All | Probability | Blood donors | 2.6% | EUROIMMUN - IgG - Anti-SARS-CoV-2 ELISA IgG | 0.9, 1 | Validated by manufacturer |

#### South-East Asia region

| Country (Location) | Author (Organization) | Sampling Dates (YMD) | Geographic scope | Overall risk of bias | Sample size | Age | Sex | Sampling method | Sample frame | Seroprevalence (95% CI) | Test Manufacturers - Isotypes - Names | Test Sens, Spec | Sens, Spec Source |
| --- | --- | --- | --- | --- | --- | --- | --- | --- | --- | --- | --- | --- | --- |
| India (Delhi) | Pragya Sharma (Maulana Azad Medical College)[197] | 2021-09-24 - 2021-10-14 | Subnational | Low | 27,811 | Multiple groups | All | Probability | Household and community samples | 88% | Ortho Clinical Diagnostics Inc. - IgG - VITROS Immunodiagnostic Products Anti-SARS-CoV-2 IgG | 1, 1 | Validated by manufacturer |
| India (Kerala) | Government of Kerala (Government of Kerala Department of Health)[198] | 2021-09-01 - 2021-09-30 | Subnational | Moderate | 1,459 | Children and Youth (0-17 years) | All | Probability | Household and community samples | 40.2% | Siemens - IgG, IgM, IgA - Atellica  IM SARS-CoV-2 Total (COV2T),Abbott Laboratories - IgG - Abbott Architect SARS-CoV-2 IgG | 1, 0.998 | Complex test algorithm: Manual calculation |
| India (Kerala) | Government of Kerala (Government of Kerala Department of Health)[198] | 2021-09-01 - 2021-09-30 | Subnational | Moderate | 1,476 | Multiple groups | All | Probability | Household and community samples | 87.7% | Siemens - IgG, IgM, IgA - Atellica  IM SARS-CoV-2 Total (COV2T),Abbott Laboratories - IgG - Abbott Architect SARS-CoV-2 IgG | 1, 0.998 | Complex test algorithm: Manual calculation |
| India (Kerala) | Government of Kerala (Government of Kerala Department of Health)[198] | 2021-09-01 - 2021-09-30 | Subnational | Moderate | 4,429 | Multiple groups | All | Probability | Household and community samples | 82.6% | Siemens - IgG, IgM, IgA - Atellica  IM SARS-CoV-2 Total (COV2T),Abbott Laboratories - IgG - Abbott Architect SARS-CoV-2 IgG | 1, 0.998 | Complex test algorithm: Manual calculation |
| India (Kerala) | Government of Kerala (Government of Kerala Department of Health)[198] | 2021-09-01 - 2021-09-30 | Subnational | Moderate | 1,706 | Multiple groups | All | Probability | Persons living in slums | 85.3% | Siemens - IgG, IgM, IgA - Atellica  IM SARS-CoV-2 Total (COV2T),Abbott Laboratories - IgG - Abbott Architect SARS-CoV-2 IgG | 1, 0.998 | Complex test algorithm: Manual calculation |
| India (Kerala) | Government of Kerala (Government of Kerala Department of Health)[198] | 2021-09-01 - 2021-09-30 | Subnational | Moderate | 1,521 | Multiple groups | All | Probability | Household and community samples | 78.2% | Siemens - IgG, IgM, IgA - Atellica  IM SARS-CoV-2 Total (COV2T),Abbott Laboratories - IgG - Abbott Architect SARS-CoV-2 IgG | 1, 0.998 | Complex test algorithm: Manual calculation |
| India (Kerala) | Government of Kerala (Government of Kerala Department of Health)[198] | 2021-08-15 - 2021-09-15 | Subnational | Moderate | 2,274 | Adults (18-64 years) | Female | Convenience | Pregnant or parturient women | 65.4% | Siemens - IgG, IgM, IgA - Atellica  IM SARS-CoV-2 Total (COV2T),Abbott Laboratories - IgG - Abbott Architect SARS-CoV-2 IgG | 1, 0.998 | Complex test algorithm: Manual calculation |
| India (1. National Capital Region (New Delhi & Faridabad) 2.Gorakhpur, Uttar Pradesh 3. Bhubaneshwar, Odisha 4. Pondicherry 5.Agartala, Tripura) | Dr Puneet Misra (All India Institute of Medical Sciences, New Delhi)[199] | 2021-03-15 - 2021-06-17 | Subnational | Moderate | 5,123 | Multiple groups | All | Probability | Household and community samples | 64.4% (95% CI 63.1-65.7%) | Beijing Wantai Biological - IgG IgM IgA - Wantai SARS-CoV-2 Total Ab ELISA | 0.967, 0.975 | Validated by manufacturer |
| India (Delhi) | Nandini Sharma (Maulana Azad Medical College)[200] | 2021-01-11 - 2021-01-22 | Subnational | Low | 27,436 | Multiple groups | All | Probability | Household and community samples | 50.5% | Ortho Clinical Diagnostics Inc. - IgG - VITROS Immunodiagnostic Products Anti-SARS-CoV-2 IgG | 1, 1 | Validated by manufacturer |
| India (Kashmir) | Muhammad Khan (Government Medical College Srinagar)[201] | 2020-10-17 - 2020-11-04 | Subnational | Low | 6,230 | Multiple groups | All | Probability | Household and community samples | 36.9% | Abbott Laboratories - IgG - Abbott Architect SARS-CoV-2 IgG | 0.99, 1 | Validated by manufacturer |
| India (NCT Delhi) | Nandini Sharma (Government of National Capital Territory of Delhi)[202] | 2020-10-15 - 2020-10-21 | Subnational | Low | 14,905 | Multiple groups | All | Probability | Household and community samples | 25.2% | Calbiotech Inc. - IgG - ErbaLisa COVID-19 IgG ELISA Kit | 0.983, 0.981 | Validated by manufacturer |
| India (Puducherry) | Sitanshu Sekhar Kar (Jawaharlal Institute)[203] | 2020-10-12 - 2020-10-16 | Subnational | Moderate | 900 | Multiple groups | All | Probability | Household and community samples | 34.5% | Roche Diagnostics - IgG, IgM, IgA - Elecsys  Anti‐SARS‐CoV‐2 (N) | 1, 0.995 | Validated by manufacturer |
| India (Puducherry) | Sitanshu Sekhar Kar (Jawaharlal Institute)[203] | 2020-09-10 - 2020-09-16 | Subnational | Moderate | 898 | Multiple groups | All | Probability | Household and community samples | 20.7% | Roche Diagnostics - IgG, IgM, IgA - Elecsys  Anti‐SARS‐CoV‐2 (N) | 1, 0.995 | Validated by manufacturer |
| India (Uttar Pradesh) | Vasanthakumar Namasivayam (Government of Uttar Pradesh)[204] | 2020-09-04 - 2020-09-10 | Subnational | Low | 16,012 | Multiple groups | All | Probability | Household and community samples | 22.1% | Zydus Diagnostics - IgG - Covid Kavach™ Anti-SARS CoV-2 IgG Antibody Detection ELISA | 0.987, 1 | Validated by manufacturer |
| India (NCT Delhi) | Nandini Sharma (Government of National Capital Territory of Delhi)[202] | 2020-09-01 - 2020-09-07 | Subnational | Low | 16,953 | Multiple groups | All | Probability | Household and community samples | 24.6% | Calbiotech Inc. - IgG - ErbaLisa COVID-19 IgG ELISA Kit | 0.983, 0.981 | Validated by manufacturer |
| India (Puducherry) | Sitanshu Sekhar Kar (Jawaharlal Institute)[203] | 2020-08-11 - 2020-08-16 | Subnational | Moderate | 869 | Multiple groups | All | Probability | Household and community samples | 4.9% | Roche Diagnostics - IgG, IgM, IgA - Elecsys  Anti‐SARS‐CoV‐2 (N) | 1, 0.995 | Validated by manufacturer |
| India (NCT Delhi) | Nandini Sharma (Government of National Capital Territory of Delhi)[202] | 2020-08-01 - 2020-08-07 | Subnational | Low | 14,779 | Multiple groups | All | Probability | Household and community samples | 28.8% | J. Mitra & Co. Pvt. Ltd - IgG - COVID Kawach IgG Microlisa | 0.921, 0.977 | Validated by developers |
| India (West Bengal) | Parthasarathi Satpati (Medical College Kolkata)[205] | 2020-07-26 - 2020-08-08 | Subnational | Moderate | 458 | Multiple groups | All | Probability | Household and community samples | 4.2% | Calbiotech Inc. - IgG - ErbaLisa COVID-19 IgG ELISA Kit | 0.983, 0.981 | Validated by manufacturer |
| India (Srinagar) | S Muhammad Salim Khan (Government Medical College Srinagar)[206] | 2020-07-01 - 2020-07-15 | Subnational | Moderate | 2,906 | Multiple groups | All | Convenience | Residual sera | 3.6% | Abbott Laboratories - IgG - Abbott Architect SARS-CoV-2 IgG | 0.99, 1 | Validated by manufacturer |
| India (NCT Delhi) | Ministry of Health and Family Welfare (Indian Center for Disease Control)[207] | 2020-06-27 - 2020-07-10 | Subnational | Moderate | 21,387 | Multiple groups | All | Probability | Household and community samples | 23.5% | Zydus Diagnostics - IgG - Covid Kavach™ Anti-SARS CoV-2 IgG Antibody Detection ELISA | 0.987, 1 | Validated by manufacturer |
| Indonesia (East Java) | Ni Luh Ayu Megasari (Kobe University)[208] | 2020-06-15 - 2020-12-15 | Subnational | Moderate | 1,819 | Multiple groups | All | Convenience | Household and community samples | 11.4% | Nanjing Vazyme Medical Technology Co. Ltd - IgG, IgM - 2019-Novel Coronavirus (2019-nCoV) IgG/IgM Detection Kit | 0.967, 0.688 | Validated by manufacturer |

#### Western Pacific region

| Country (Location) | Author (Organization) | Sampling Dates (YMD) | Geographic scope | Overall risk of bias | Sample size | Age | Sex | Sampling method | Sample frame | Seroprevalence (95% CI) | Test Manufacturers - Isotypes - Names | Test Sens, Spec | Sens, Spec Source |
| --- | --- | --- | --- | --- | --- | --- | --- | --- | --- | --- | --- | --- | --- |
| China (Hong Kong) | Lin-Lei Chen (The University of Hong Kong)[209] | 2021-11-15 - 2021-12-15 | Subnational | Moderate | 300 | Multiple groups | All | Convenience | Residual sera | 30.3% | Shenzhen Yhlo Biotech Co. Ltd - IgG - iFlash-SARS-CoV-2 S1 IgG | 0.9, 0.98 | Validated by manufacturer |
| China (Wuhan) | Zhenyu He (Chinese Academy of Medical Sciences & Peking Union Medical College)[210] | 2020-04-14 - 2020-04-15 | Subnational | Low | 9,542 | Multiple groups | All | Probability | Household and community samples | 6.9% | Roche Diagnostics - IgG, IgM, IgA - Elecsys  Anti‐SARS‐CoV‐2 (N) | 1, 0.995 | Validated by manufacturer |
| Japan (Kanagawa Prefecture) | Atsushi Goto (Yokohama City University)[211] | 2022-01-30 - 2022-02-28 | Subnational | Low | 1,277 | Multiple groups | All | Probability | Household and community samples | 94.2% | TOSOH - IgG - AIA-CL SARS-CoV-2 SP-IgG | 1, 1 | Validated by manufacturer |
| Japan (Hiroshima Prefecture) | Prefecture of Hiroshima (Hiroshima University )[212] | 2020-10-15 - 2020-11-15 | Subnational | Moderate | 2,396 | Multiple groups | All | Probability | Household and community samples | 0.2% | Roche Diagnostics - IgG, IgM, IgA - Elecsys  Anti‐SARS‐CoV‐2 (N) | 1, 0.995 | Validated by manufacturer |
| Malaysia (Selangor) | I-Ching Sam (University of Malaya)[213] | 2020-12-15 - 2021-04-15 | Subnational | Moderate | 653 | Multiple groups | All | Convenience | Residual sera | 4.1% | Beijing Wantai Biological - IgG, IgM, IgA - Wantai SARS-CoV-2 Total Ab ELISA | 0.967, 0.975 | Validated by manufacturer |

## Table C. Additional studies used in descriptive analysis.

#### Africa region

| Country (Location) | Author (Organization) | Sampling Dates (YMD) | Geographic scope | Overall risk of bias | Sample size | Age | Sex | Sampling method | Sample frame | Seroprevalence (95% CI) | Test Manufacturers - Isotypes - Names | Test Sens, Spec | Sens, Spec Source |
| --- | --- | --- | --- | --- | --- | --- | --- | --- | --- | --- | --- | --- | --- |
| Angola - HRP (Luanda) | Cruz Sebastiao (Instituto Nacional de Investigação em Saúde)[214] | 2020-09-01 - 2020-09-30 | Local | High | 55 | Adults (18-64 years) | All | Convenience | Blood donors | 20% | Biomerieux - IgG, IgM, IgA - VIDAS Multiparametric immunoassay system for medium throughput | 1, 1 | Validated by manufacturer |
| Burkina Faso - HRP (Ouagadougou & Bobo-Dioulasso) | Dr Isidore Traore (National Institute of Public Health)[215] | 2021-04-03 - 2021-04-16 | Local | Moderate | 5,240 | Multiple groups | All | Probability | Household and community samples | 67.8% (95% CI 66.5-69.1%) | Beijing Wantai Biological - IgG IgM IgA - Wantai SARS-CoV-2 Total Ab ELISA | 0.967, 0.975 | Validated by manufacturer |
| Cameroon - HRP (Yaoundé) | Kene Nwosu (Hopital Central de Yaounde)[216] | 2020-10-14 - 2020-11-26 | Local | Low | 971 | Multiple groups | All | Probability | Household and community samples | 31.3% | Abbott Laboratories - IgG, IgM - Panbio COVID-19 IgG/IgM rapid test device | 0.978, 0.928 | Validated by manufacturer |
| Central African Republic - HRP (Bangui, the capital of the Central African Republic) | Alexandre Manirakiza (Institut Pasteur of Bangui)[217] | 2021-07-12 - 2021-08-20 | Local | Moderate | 799 | Multiple groups | All | Probability | Household and community samples | 75.5% (95% CI 72.3-78.4%) | Beijing Wantai Biological - IgG IgM IgA - Wantai SARS-CoV-2 Total Ab ELISA | 0.967, 0.975 | Validated by manufacturer |
| Congo - HRP | Novy Bonguili (Laboratoire National de Santé Publique)[218] | 2020-01-01 - 2020-02-15 | National | High | 484 | Multiple groups | All | Convenience | Representative patient population | 5% | NA - IgG, IgM - Author designed (IFA) - NON-MULTIPLEXED | 1, 0.975 | Validated by independent authors/third party/non-developers |
| Democratic Republic of the Congo - HRP (Kinshasa) | Antoine Nkuba (Institut National de Recherche Biomédicale)[219] | 2020-10-22 - 2020-11-08 | Local | Low | 1,080 | Multiple groups | All | Probability | Household and community samples | 16.6% | NA - - Author designed (Luminex) | 1, 0.997 | Validated by developers |
| Ethiopia - HRP (Addis Ababa) | Esayas Gudina (University of Munich)[220] | 2021-04-08 - 2021-04-10 | Local | High | 176 | Multiple groups | All | Convenience | Household and community samples | 72.7% | Roche Diagnostics - IgG, IgM, IgA - Elecsys  Anti‐SARS‐CoV‐2 (N) | 1, 0.995 | Validated by manufacturer |
| Ethiopia - HRP (Addis Ababa) | Esayas Gudina (University of Munich)[220] | 2021-04-01 - 2021-04-03 | Local | High | 188 | Multiple groups | All | Convenience | Household and community samples | 54.8% | Roche Diagnostics - IgG, IgM, IgA - Elecsys  Anti‐SARS‐CoV‐2 (N) | 1, 0.995 | Validated by manufacturer |
| Ethiopia - HRP (Jimma) | Esayas Gudina (University of Munich)[220] | 2021-02-04 - 2021-03-16 | Local | High | 100 | Multiple groups | All | Convenience | Household and community samples | 31% | Roche Diagnostics - IgG, IgM, IgA - Elecsys  Anti‐SARS‐CoV‐2 (N) | 1, 0.995 | Validated by manufacturer |
| Ethiopia - HRP (Jimma) | Esayas Gudina (University of Munich)[220] | 2021-02-02 - 2021-03-15 | Local | High | 166 | Multiple groups | All | Convenience | Household and community samples | 45.2% | Roche Diagnostics - IgG, IgM, IgA - Elecsys  Anti‐SARS‐CoV‐2 (N) | 1, 0.995 | Validated by manufacturer |
| Ethiopia - HRP (Addis Ababa) | Esayas Gudina (University of Munich)[220] | 2021-02-11 - 2021-02-26 | Local | High | 151 | Multiple groups | All | Convenience | Household and community samples | 58.2% | Roche Diagnostics - IgG, IgM, IgA - Elecsys  Anti‐SARS‐CoV‐2 (N) | 1, 0.995 | Validated by manufacturer |
| Ethiopia - HRP (Addis Ababa) | Esayas Gudina (University of Munich)[220] | 2021-02-01 - 2021-03-07 | Local | High | 149 | Multiple groups | All | Convenience | Household and community samples | 45.7% | Roche Diagnostics - IgG, IgM, IgA - Elecsys  Anti‐SARS‐CoV‐2 (N) | 1, 0.995 | Validated by manufacturer |
| Ethiopia - HRP (Jimma) | Esayas Gudina (University of Munich)[220] | 2021-01-21 - 2021-02-25 | Local | High | 133 | Multiple groups | All | Convenience | Household and community samples | 26.3% | Roche Diagnostics - IgG, IgM, IgA - Elecsys  Anti‐SARS‐CoV‐2 (N) | 1, 0.995 | Validated by manufacturer |
| Ethiopia - HRP (Jimma) | Esayas Gudina (University of Munich)[220] | 2021-01-02 - 2021-02-04 | Local | High | 191 | Multiple groups | All | Convenience | Household and community samples | 40.8% | Roche Diagnostics - IgG, IgM, IgA - Elecsys  Anti‐SARS‐CoV‐2 (N) | 1, 0.995 | Validated by manufacturer |
| Ethiopia - HRP (Addis Ababa) | Esayas Gudina (University of Munich)[220] | 2021-01-02 - 2021-01-22 | Local | High | 218 | Multiple groups | All | Convenience | Household and community samples | 54.2% | Roche Diagnostics - IgG, IgM, IgA - Elecsys  Anti‐SARS‐CoV‐2 (N) | 1, 0.995 | Validated by manufacturer |
| Ethiopia - HRP (Addis Ababa) | Esayas Gudina (University of Munich)[220] | 2020-12-05 - 2021-02-04 | Local | High | 224 | Multiple groups | All | Convenience | Household and community samples | 39.7% | Roche Diagnostics - IgG, IgM, IgA - Elecsys  Anti‐SARS‐CoV‐2 (N) | 1, 0.995 | Validated by manufacturer |
| Ethiopia - HRP (Jimma) | Esayas Gudina (University of Munich)[220] | 2020-12-01 - 2021-02-01 | Local | High | 297 | Multiple groups | All | Convenience | Household and community samples | 32.3% | Roche Diagnostics - IgG, IgM, IgA - Elecsys  Anti‐SARS‐CoV‐2 (N) | 1, 0.995 | Validated by manufacturer |
| Ethiopia - HRP (Jimma) | Esayas Gudina (University of Munich)[220] | 2020-12-03 - 2021-01-27 | Local | High | 238 | Multiple groups | All | Convenience | Household and community samples | 18% | Roche Diagnostics - IgG, IgM, IgA - Elecsys  Anti‐SARS‐CoV‐2 (N) | 1, 0.995 | Validated by manufacturer |
| Ethiopia - HRP (Addis Ababa) | Saro Abdella (Ethiopian Public Health Institute)[221] | 2020-07-22 - 2020-09-02 | Local | Low | 956 | Multiple groups | All | Probability | Household and community samples | 4.7% | Core Technology Co. Ltd - IgG, IgM - Coretests COVID-19 IgM/IgG Ab Test | 0.939, 0.981 | Validated by manufacturer |
| Ethiopia - HRP (Jimma) | Saro Abdella (Ethiopian Public Health Institute)[221] | 2020-07-22 - 2020-09-02 | Local | Low | 900 | Multiple groups | All | Probability | Household and community samples | 1.2% | Core Technology Co. Ltd - IgG, IgM - Coretests COVID-19 IgM/IgG Ab Test | 0.939, 0.981 | Validated by manufacturer |
| Ethiopia - HRP (Dire Dawa city) | Tamrat Shaweno (Jimma University Institute of Health)[222] | 2020-06-15 - 2020-07-30 | Local | Moderate | 684 | Multiple groups | All | Probability | Household and community samples | 3.2% | Abbott Laboratories - IgG - Abbott Architect SARS-CoV-2 IgG | 0.99, 1 | Validated by manufacturer |
| Ethiopia - HRP (Addis Ababa) | John Kempen (Kuwait University)[223] | 2020-05-18 - 2020-05-21 | Local | High | 99 | Multiple groups | All | Convenience | Residual sera | 3% | Abbott Laboratories - IgG - Abbott Architect SARS-CoV-2 IgG | 0.99, 1 | Validated by manufacturer |
| Gabon (Libreville) | Amandine Mveang Nzoghe (Centre Hospitalier Universitaire (CHU) ‐ Mère‐ Enfant)[224] | 2020-07-15 - 2020-10-15 | Local | High | 1,495 | Multiple groups | All | Convenience | Residual sera | 36.2% | Biomerieux - IgG, IgM, IgA - VIDAS Multiparametric immunoassay system for medium throughput,Roche Diagnostics - IgG, IgM, IgA - Elecsys  Anti‐SARS‐CoV‐2 (N) | 1, 0.995 | Complex test algorithm: Manual calculation |
| Kenya - HRP (Busia) | R Lucinde (KEMRI-Wellcome Trust Research Programme)[225] | 2021-09-20 - 2021-10-22 | Local | High | 297 | Adults (18-64 years) | Female | Sequential | Pregnant or parturient women | 65.7% | NA - - Author designed (ELISA) -Spike | 0.927, 0.99 | Validated by developers |
| Kenya - HRP (Kilifi) | R Lucinde (KEMRI-Wellcome Trust Research Programme)[225] | 2021-09-01 - 2021-10-31 | Local | High | 305 | Adults (18-64 years) | Female | Sequential | Pregnant or parturient women | 58.4% | NA - - Author designed (ELISA) -Spike | 0.927, 0.99 | Validated by developers |
| Kenya - HRP (Nairobi) | R Lucinde (KEMRI-Wellcome Trust Research Programme)[225] | 2021-09-07 - 2021-10-19 | Local | High | 245 | Adults (18-64 years) | Female | Sequential | Pregnant or parturient women | 78.8% | NA - - Author designed (ELISA) -Spike | 0.927, 0.99 | Validated by developers |
| Kenya - HRP | James Nyagwange (KEMRI-Wellcome Trust Research Programme)[226] | 2021-08-01 - 2021-08-31 | National | High | 176 | Adults (18-64 years) | All | Convenience | Blood donors | 83% | Beijing Wantai Biological - IgG, IgM, IgA - Wantai SARS-CoV-2 Total Ab ELISA | 0.967, 0.975 | Validated by manufacturer |
| Kenya - HRP (Kilifi) | R Lucinde (KEMRI-Wellcome Trust Research Programme)[225] | 2021-07-01 - 2021-08-31 | Local | High | 260 | Adults (18-64 years) | Female | Sequential | Pregnant or parturient women | 56.9% | NA - - Author designed (ELISA) -Spike | 0.927, 0.99 | Validated by developers |
| Kenya - HRP (Kilifi) | R Lucinde (KEMRI-Wellcome Trust Research Programme)[225] | 2021-05-01 - 2021-06-30 | Local | High | 382 | Adults (18-64 years) | Female | Sequential | Pregnant or parturient women | 27.2% | NA - - Author designed (ELISA) -Spike | 0.927, 0.99 | Validated by developers |
| Kenya - HRP (Kisumu) | Anthony Etyang (KEMRI-Wellcome Trust Research Programme)[227] | 2021-05-01 - 2021-05-31 | Local | Low | 217 | Multiple groups | All | Probability | Household and community samples | 39.2% | NA - - Author designed (ELISA) -Spike | 0.927, 0.99 | Validated by independent authors/third party/non-developers |
| Kenya - HRP (Nairobi) | Anthony Etyang (KEMRI-Wellcome Trust Research Programme)[227] | 2021-05-01 - 2021-05-31 | Local | Low | 113 | Multiple groups | All | Probability | Household and community samples | 47.8% | NA - - Author designed (ELISA) -Spike | 0.927, 0.99 | Validated by independent authors/third party/non-developers |
| Kenya - HRP (Busia) | R Lucinde (KEMRI-Wellcome Trust Research Programme)[225] | 2021-04-15 - 2021-05-21 | Local | High | 270 | Adults (18-64 years) | Female | Sequential | Pregnant or parturient women | 28.9% | NA - - Author designed (ELISA) -Spike | 0.927, 0.99 | Validated by developers |
| Kenya - HRP (Kilifi) | Anthony Etyang (KEMRI-Wellcome Trust Research Programme)[227] | 2021-04-01 - 2021-04-30 | Local | Low | 145 | Multiple groups | All | Probability | Household and community samples | 24.1% | NA - - Author designed (ELISA) -Spike | 0.927, 0.99 | Validated by independent authors/third party/non-developers |
| Kenya - HRP (Kilifi) | R Lucinde (KEMRI-Wellcome Trust Research Programme)[225] | 2021-03-01 - 2021-04-30 | Local | High | 260 | Adults (18-64 years) | Female | Sequential | Pregnant or parturient women | 21.1% | NA - - Author designed (ELISA) -Spike | 0.927, 0.99 | Validated by developers |
| Kenya - HRP (Nairobi) | R Lucinde (KEMRI-Wellcome Trust Research Programme)[225] | 2021-01-27 - 2021-03-11 | Local | High | 265 | Adults (18-64 years) | Female | Sequential | Pregnant or parturient women | 30.2% | NA - - Author designed (ELISA) -Spike | 0.927, 0.99 | Validated by developers |
| Kenya - HRP (Nairobi) | Patrick Munywoki (Kenya Medical Research Institute)[228] | 2020-11-27 - 2020-12-05 | Local | Moderate | 511 | Multiple groups | All | Probability | Household and community samples | 43.3% | Beijing Wantai Biological - IgG, IgM, IgA - Wantai SARS-CoV-2 Total Ab ELISA | 0.967, 0.975 | Validated by manufacturer |
| Kenya - HRP (Kilifi) | R Lucinde (KEMRI-Wellcome Trust Research Programme)[225] | 2020-11-01 - 2020-12-31 | Local | High | 236 | Adults (18-64 years) | Female | Sequential | Pregnant or parturient women | 13.6% | NA - - Author designed (ELISA) -Spike | 0.927, 0.99 | Validated by developers |
| Kenya - HRP (Kilifi) | R Lucinde (KEMRI-Wellcome Trust)[229] | 2020-11-01 - 2020-11-24 | Local | High | 154 | Adults (18-64 years) | Female | Sequential | Pregnant or parturient women | 10.4% | NA - IgG - Author designed (ELISA) -Spike | 0.927, 0.99 | Validated by independent authors/third party/non-developers |
| Kenya - HRP | Isaac Ngere (Washington State University)[230] | 2020-11-02 - 2020-11-23 | Local | Low | 1,164 | Multiple groups | All | Probability | Household and community samples | 32.7% | Beijing Wantai Biological - IgG, IgM, IgA - Wantai SARS-CoV-2 Total Ab ELISA | 0.967, 0.975 | Validated by manufacturer |
| Kenya - HRP (Kilifi) | R Lucinde (KEMRI-Wellcome Trust)[229] | 2020-10-01 - 2020-10-31 | Local | High | 183 | Adults (18-64 years) | Female | Sequential | Pregnant or parturient women | 1.6% | NA - IgG - Author designed (ELISA) -Spike | 0.927, 0.99 | Validated by independent authors/third party/non-developers |
| Kenya - HRP (Kilifi) | R Lucinde (KEMRI-Wellcome Trust)[229] | 2020-09-18 - 2020-09-30 | Local | High | 82 | Adults (18-64 years) | Female | Sequential | Pregnant or parturient women | 0% | NA - IgG - Author designed (ELISA) -Spike | 0.927, 0.99 | Validated by independent authors/third party/non-developers |
| Kenya - HRP (Nairobi) | R Lucinde (KEMRI-Wellcome Trust)[229] | 2020-07-30 - 2020-08-25 | Local | High | 196 | Adults (18-64 years) | Female | Sequential | Pregnant or parturient women | 46.4% | NA - IgG - Author designed (ELISA) -Spike | 0.927, 0.99 | Validated by independent authors/third party/non-developers |
| Kenya - HRP | James Nyagwange (KEMRI-Wellcome Trust Research Programme)[226] | 2020-05-01 - 2020-05-31 | National | High | 176 | Adults (18-64 years) | All | Convenience | Blood donors | 2.3% | Beijing Wantai Biological - IgG, IgM, IgA - Wantai SARS-CoV-2 Total Ab ELISA | 0.967, 0.975 | Validated by manufacturer |
| Kenya - HRP (Western Region) | Trevor Crowell (Walter Reed Army Institute of Research)[231] | 2020-01-01 - 2020-03-19 | Subnational | High | 582 | Adults (18-64 years) | All | Convenience | Residual sera | 3.3% | Bio-rad - IgG, IgM, IgA - Platelia SARS-CoV-2 Total Ab assay | 0.993, 0.98 | Validated by manufacturer |
| Madagascar (Antananarivo) | Solohery Razafimahatratra (Institut Pasteur de Madagascar)[232] | 2021-05-01 - 2021-05-26 | Local | High | 500 | Adults (18-64 years) | All | Sequential | Blood donors | 64.8% | Beijing Wantai Biological - IgG, IgM, IgA - Wantai SARS-CoV-2 Total Ab ELISA | 0.967, 0.975 | Validated by manufacturer |
| Madagascar (Antananarivo) | Solohery Razafimahatratra (Institut Pasteur de Madagascar)[232] | 2021-04-01 - 2021-04-30 | Local | High | 493 | Adults (18-64 years) | All | Sequential | Blood donors | 58.4% | Beijing Wantai Biological - IgG, IgM, IgA - Wantai SARS-CoV-2 Total Ab ELISA | 0.967, 0.975 | Validated by manufacturer |
| Madagascar (Antananarivo) | Solohery Razafimahatratra (Institut Pasteur de Madagascar)[232] | 2021-03-01 - 2021-03-31 | Local | High | 497 | Adults (18-64 years) | All | Sequential | Blood donors | 49.9% | Beijing Wantai Biological - IgG, IgM, IgA - Wantai SARS-CoV-2 Total Ab ELISA | 0.967, 0.975 | Validated by manufacturer |
| Madagascar (Antananarivo) | Solohery Razafimahatratra (Institut Pasteur de Madagascar)[232] | 2021-02-01 - 2021-02-28 | Local | High | 319 | Adults (18-64 years) | All | Sequential | Blood donors | 50.5% | Beijing Wantai Biological - IgG, IgM, IgA - Wantai SARS-CoV-2 Total Ab ELISA | 0.967, 0.975 | Validated by manufacturer |
| Madagascar (Antananarivo) | Solohery Razafimahatratra (Institut Pasteur de Madagascar)[232] | 2021-01-01 - 2021-01-31 | Local | High | 389 | Adults (18-64 years) | All | Sequential | Blood donors | 44.7% | Beijing Wantai Biological - IgG, IgM, IgA - Wantai SARS-CoV-2 Total Ab ELISA | 0.967, 0.975 | Validated by manufacturer |
| Madagascar (Antananarivo) | Solohery Razafimahatratra (Institut Pasteur de Madagascar)[232] | 2020-12-01 - 2020-12-31 | Local | High | 177 | Adults (18-64 years) | All | Sequential | Blood donors | 22% | ID.Vet - IgG - ID Screen | 0.952, 0.998 | Validated by manufacturer |
| Madagascar (Antananarivo) | Matthieu Schoenhals (Institut Pasteur de Madagascar)[233] | 2020-11-01 - 2020-11-30 | Local | High | 502 | Adults (18-64 years) | All | Convenience | Blood donors | 31.3% | ID.Vet - IgG - ID Screen | 0.952, 0.998 | Validated by manufacturer |
| Madagascar (Fianarantsoa) | Matthieu Schoenhals (Institut Pasteur de Madagascar)[233] | 2020-11-01 - 2020-11-30 | Local | High | 172 | Adults (18-64 years) | All | Convenience | Blood donors | 12.8% | ID.Vet - IgG - ID Screen | 0.952, 0.998 | Validated by manufacturer |
| Madagascar (Mahajanga) | Matthieu Schoenhals (Institut Pasteur de Madagascar)[233] | 2020-11-01 - 2020-11-30 | Local | High | 30 | Adults (18-64 years) | All | Convenience | Blood donors | 16.7% | ID.Vet - IgG - ID Screen | 0.952, 0.998 | Validated by manufacturer |
| Madagascar (Toamasina) | Matthieu Schoenhals (Institut Pasteur de Madagascar)[233] | 2020-11-01 - 2020-11-30 | Local | High | 282 | Adults (18-64 years) | All | Convenience | Blood donors | 18.4% | ID.Vet - IgG - ID Screen | 0.952, 0.998 | Validated by manufacturer |
| Madagascar (Toliara) | Matthieu Schoenhals (Institut Pasteur de Madagascar)[233] | 2020-11-01 - 2020-11-30 | Local | High | 199 | Adults (18-64 years) | All | Convenience | Blood donors | 18.1% | ID.Vet - IgG - ID Screen | 0.952, 0.998 | Validated by manufacturer |
| Madagascar (Antananarivo) | Solohery Razafimahatratra (Institut Pasteur de Madagascar)[232] | 2020-11-01 - 2020-11-30 | Local | High | 503 | Adults (18-64 years) | All | Sequential | Blood donors | 31.2% | ID.Vet - IgG - ID Screen | 0.952, 0.998 | Validated by manufacturer |
| Madagascar (Antananarivo) | Matthieu Schoenhals (Institut Pasteur de Madagascar)[233] | 2020-10-01 - 2020-10-31 | Local | High | 502 | Adults (18-64 years) | All | Convenience | Blood donors | 36% | ID.Vet - IgG - ID Screen | 0.952, 0.998 | Validated by manufacturer |
| Madagascar (Fianarantsoa) | Matthieu Schoenhals (Institut Pasteur de Madagascar)[233] | 2020-10-01 - 2020-10-31 | Local | High | 152 | Adults (18-64 years) | All | Convenience | Blood donors | 15.1% | ID.Vet - IgG - ID Screen | 0.952, 0.998 | Validated by manufacturer |
| Madagascar (Mahajanga) | Matthieu Schoenhals (Institut Pasteur de Madagascar)[233] | 2020-10-01 - 2020-10-31 | Local | High | 26 | Adults (18-64 years) | All | Convenience | Blood donors | 26.9% | ID.Vet - IgG - ID Screen | 0.952, 0.998 | Validated by manufacturer |
| Madagascar (Toamasina) | Matthieu Schoenhals (Institut Pasteur de Madagascar)[233] | 2020-10-01 - 2020-10-31 | Local | High | 199 | Adults (18-64 years) | All | Convenience | Blood donors | 26.1% | ID.Vet - IgG - ID Screen | 0.952, 0.998 | Validated by manufacturer |
| Madagascar (Toliara) | Matthieu Schoenhals (Institut Pasteur de Madagascar)[233] | 2020-10-01 - 2020-10-31 | Local | High | 139 | Adults (18-64 years) | All | Convenience | Blood donors | 22.3% | ID.Vet - IgG - ID Screen | 0.952, 0.998 | Validated by manufacturer |
| Madagascar (Antananarivo) | Solohery Razafimahatratra (Institut Pasteur de Madagascar)[232] | 2020-10-01 - 2020-10-31 | Local | High | 497 | Adults (18-64 years) | All | Sequential | Blood donors | 35.8% | ID.Vet - IgG - ID Screen | 0.952, 0.998 | Validated by manufacturer |
| Madagascar (Antananarivo) | Matthieu Schoenhals (Institut Pasteur de Madagascar)[233] | 2020-09-01 - 2020-09-30 | Local | High | 481 | Adults (18-64 years) | All | Convenience | Blood donors | 38.5% | ID.Vet - IgG - ID Screen | 0.952, 0.998 | Validated by manufacturer |
| Madagascar (Fianarantsoa) | Matthieu Schoenhals (Institut Pasteur de Madagascar)[233] | 2020-09-01 - 2020-09-30 | Local | High | 173 | Adults (18-64 years) | All | Convenience | Blood donors | 18.5% | ID.Vet - IgG - ID Screen | 0.952, 0.998 | Validated by manufacturer |
| Madagascar (Mahajanga) | Matthieu Schoenhals (Institut Pasteur de Madagascar)[233] | 2020-09-01 - 2020-09-30 | Local | High | 53 | Adults (18-64 years) | All | Convenience | Blood donors | 37.7% | ID.Vet - IgG - ID Screen | 0.952, 0.998 | Validated by manufacturer |
| Madagascar (Toamasina) | Matthieu Schoenhals (Institut Pasteur de Madagascar)[233] | 2020-09-01 - 2020-09-30 | Local | High | 213 | Adults (18-64 years) | All | Convenience | Blood donors | 29.1% | ID.Vet - IgG - ID Screen | 0.952, 0.998 | Validated by manufacturer |
| Madagascar (Toliara) | Matthieu Schoenhals (Institut Pasteur de Madagascar)[233] | 2020-09-01 - 2020-09-30 | Local | High | 185 | Adults (18-64 years) | All | Convenience | Blood donors | 20.5% | ID.Vet - IgG - ID Screen | 0.952, 0.998 | Validated by manufacturer |
| Madagascar (Fianarantsoa) | Matthieu Schoenhals (Institut Pasteur de Madagascar)[233] | 2020-08-01 - 2020-08-31 | Local | High | 120 | Adults (18-64 years) | All | Convenience | Blood donors | 9.2% | ID.Vet - IgG - ID Screen | 0.952, 0.998 | Validated by manufacturer |
| Madagascar (Mahajanga) | Matthieu Schoenhals (Institut Pasteur de Madagascar)[233] | 2020-08-01 - 2020-08-31 | Local | High | 107 | Adults (18-64 years) | All | Convenience | Blood donors | 35.5% | ID.Vet - IgG - ID Screen | 0.952, 0.998 | Validated by manufacturer |
| Madagascar (Toamasina) | Matthieu Schoenhals (Institut Pasteur de Madagascar)[233] | 2020-08-01 - 2020-08-31 | Local | High | 227 | Adults (18-64 years) | All | Convenience | Blood donors | 40.1% | ID.Vet - IgG - ID Screen | 0.952, 0.998 | Validated by manufacturer |
| Madagascar (Toliara) | Matthieu Schoenhals (Institut Pasteur de Madagascar)[233] | 2020-08-01 - 2020-08-31 | Local | High | 167 | Adults (18-64 years) | All | Convenience | Blood donors | 7.2% | ID.Vet - IgG - ID Screen | 0.952, 0.998 | Validated by manufacturer |
| Madagascar (Antananarivo) | Matthieu Schoenhals (Institut Pasteur de Madagascar)[233] | 2020-08-01 - 2020-08-30 | Local | High | 432 | Adults (18-64 years) | All | Convenience | Blood donors | 35.2% | ID.Vet - IgG - ID Screen | 0.952, 0.998 | Validated by manufacturer |
| Madagascar (Antananarivo) | Matthieu Schoenhals (Institut Pasteur de Madagascar)[233] | 2020-07-01 - 2020-07-31 | Local | High | 428 | Adults (18-64 years) | All | Convenience | Blood donors | 29% | ID.Vet - IgG - ID Screen | 0.952, 0.998 | Validated by manufacturer |
| Madagascar (Mahajanga) | Matthieu Schoenhals (Institut Pasteur de Madagascar)[233] | 2020-07-01 - 2020-07-31 | Local | High | 98 | Adults (18-64 years) | All | Convenience | Blood donors | 1% | ID.Vet - IgG - ID Screen | 0.952, 0.998 | Validated by manufacturer |
| Madagascar (Toamasina) | Matthieu Schoenhals (Institut Pasteur de Madagascar)[233] | 2020-07-01 - 2020-07-31 | Local | High | 182 | Adults (18-64 years) | All | Convenience | Blood donors | 43.4% | ID.Vet - IgG - ID Screen | 0.952, 0.998 | Validated by manufacturer |
| Madagascar (Toliara) | Matthieu Schoenhals (Institut Pasteur de Madagascar)[233] | 2020-07-01 - 2020-07-31 | Local | High | 147 | Adults (18-64 years) | All | Convenience | Blood donors | 0.7% | ID.Vet - IgG - ID Screen | 0.952, 0.998 | Validated by manufacturer |
| Madagascar (Fianarantsoa) | Matthieu Schoenhals (Institut Pasteur de Madagascar)[233] | 2020-07-01 - 2020-07-30 | Local | High | 142 | Adults (18-64 years) | All | Convenience | Blood donors | 0% | ID.Vet - IgG - ID Screen | 0.952, 0.998 | Validated by manufacturer |
| Madagascar (Antananarivo) | Matthieu Schoenhals (Institut Pasteur de Madagascar)[233] | 2020-06-01 - 2020-06-30 | Local | High | 308 | Adults (18-64 years) | All | Convenience | Blood donors | 3.3% | ID.Vet - IgG - ID Screen | 0.952, 0.998 | Validated by manufacturer |
| Madagascar (Fianarantsoa) | Matthieu Schoenhals (Institut Pasteur de Madagascar)[233] | 2020-06-01 - 2020-06-30 | Local | High | 129 | Adults (18-64 years) | All | Convenience | Blood donors | 0.8% | ID.Vet - IgG - ID Screen | 0.952, 0.998 | Validated by manufacturer |
| Madagascar (Mahajanga) | Matthieu Schoenhals (Institut Pasteur de Madagascar)[233] | 2020-06-01 - 2020-06-30 | Local | High | 50 | Adults (18-64 years) | All | Convenience | Blood donors | 0% | ID.Vet - IgG - ID Screen | 0.952, 0.998 | Validated by manufacturer |
| Madagascar (Toamasina) | Matthieu Schoenhals (Institut Pasteur de Madagascar)[233] | 2020-06-01 - 2020-06-30 | Local | High | 82 | Adults (18-64 years) | All | Convenience | Blood donors | 39% | ID.Vet - IgG - ID Screen | 0.952, 0.998 | Validated by manufacturer |
| Madagascar (Toliara) | Matthieu Schoenhals (Institut Pasteur de Madagascar)[233] | 2020-06-01 - 2020-06-30 | Local | High | 87 | Adults (18-64 years) | All | Convenience | Blood donors | 1.2% | ID.Vet - IgG - ID Screen | 0.952, 0.998 | Validated by manufacturer |
| Madagascar (Antananarivo) | Matthieu Schoenhals (Institut Pasteur de Madagascar)[233] | 2020-05-01 - 2020-05-31 | Local | High | 598 | Adults (18-64 years) | All | Convenience | Blood donors | 0.2% | ID.Vet - IgG - ID Screen | 0.952, 0.998 | Validated by manufacturer |
| Madagascar (Antananarivo) | Matthieu Schoenhals (Institut Pasteur de Madagascar)[233] | 2020-04-01 - 2020-04-30 | Local | High | 157 | Adults (18-64 years) | All | Convenience | Blood donors | 0% | ID.Vet - IgG - ID Screen | 0.952, 0.998 | Validated by manufacturer |
| Madagascar (Antananarivo) | Matthieu Schoenhals (Institut Pasteur de Madagascar)[233] | 2020-03-01 - 2020-03-30 | Local | High | 496 | Adults (18-64 years) | All | Convenience | Blood donors | 0.8% | ID.Vet - IgG - ID Screen | 0.952, 0.998 | Validated by manufacturer |
| Mali - HRP (Bancoumana) | Issaka Sagara (National Institutes of Health)[234] | 2020-12-28 - 2021-01-29 | Local | Moderate | 904 | Multiple groups | All | Convenience | Household and community samples | 35.5% | NA - - Author designed (ELISA) -Spike | 0.739, 0.994 | Validated by independent authors/third party/non-developers |
| Mali - HRP (Bamako) | Issaka Sagara (National Institutes of Health)[234] | 2020-12-21 - 2021-01-26 | Local | Moderate | 528 | Multiple groups | All | Convenience | Household and community samples | 44.9% | NA - - Author designed (ELISA) -Spike | 0.739, 0.994 | Validated by independent authors/third party/non-developers |
| Mali - HRP (Doneguebougou) | Issaka Sagara (National Institutes of Health)[234] | 2020-12-14 - 2021-01-15 | Local | Moderate | 1,088 | Multiple groups | All | Convenience | Household and community samples | 25.8% | NA - - Author designed (ELISA) -Spike | 0.739, 0.994 | Validated by independent authors/third party/non-developers |
| Mali - HRP (Bamako) | Issaka Sagara (National Institutes of Health)[234] | 2020-07-29 - 2020-10-16 | Local | Moderate | 587 | Multiple groups | All | Convenience | Household and community samples | 13.1% | NA - - Author designed (ELISA) -Spike | 0.739, 0.994 | Validated by independent authors/third party/non-developers |
| Mali - HRP (Bancoumana) | Issaka Sagara (National Institutes of Health)[234] | 2020-07-29 - 2020-09-24 | Local | Moderate | 963 | Multiple groups | All | Convenience | Household and community samples | 5.3% | NA - - Author designed (ELISA) -Spike | 0.739, 0.994 | Validated by independent authors/third party/non-developers |
| Mali - HRP (Doneguebougou) | Issaka Sagara (National Institutes of Health)[234] | 2020-07-28 - 2020-08-27 | Local | Moderate | 1,109 | Multiple groups | All | Convenience | Household and community samples | 4.1% | NA - - Author designed (ELISA) -Spike | 0.739, 0.994 | Validated by independent authors/third party/non-developers |
| Mozambique - HRP (Massinga) | Mussagy Mahomed (Republica de Mocambique Ministerio da Saude)[235] | 2020-11-26 - 2020-12-03 | Local | Moderate | 1,577 | Multiple groups | All | Probability | Household and community samples | 7.4% | Qingdao Hightop Biotech Co. Ltd - IgG, IgM - Hightop COVID-19 IgM/IgG Ab Rapid Test Kit | 0.942, 0.939 | Validated by manufacturer |
| Mozambique - HRP (Maxixe) | Mussagy Mahomed (Republica de Mocambique Ministerio da Saude)[235] | 2020-11-07 - 2020-11-21 | Local | Moderate | 3,974 | Multiple groups | All | Probability | Household and community samples | 5.5% | Qingdao Hightop Biotech Co. Ltd - IgG, IgM - Hightop COVID-19 IgM/IgG Ab Rapid Test Kit | 0.942, 0.939 | Validated by manufacturer |
| Mozambique - HRP (Chimoio) | Arlete Mahumane (Republica de Mocambique Ministerio da Saude)[236] | 2020-11-02 - 2020-11-17 | Local | Moderate | 9,756 | Multiple groups | All | Probability | Household and community samples | 1.4% | Qingdao Hightop Biotech Co. Ltd - IgG, IgM - Hightop COVID-19 IgM/IgG Ab Rapid Test Kit | 0.942, 0.939 | Validated by manufacturer |
| Mozambique - HRP (Lichinga) | Jeronimo Langa (Republica de Mocambique Ministerio da Saude)[237] | 2020-09-28 - 2020-10-09 | Local | Moderate | 1,635 | Multiple groups | All | Probability | Household and community samples | 0.3% | Qingdao Hightop Biotech Co. Ltd - IgG, IgM - Hightop COVID-19 IgM/IgG Ab Rapid Test Kit | 0.942, 0.939 | Validated by manufacturer |
| Mozambique - HRP (Tete) | Mussagy Mahomed (Republica de Mocambique Ministerio da Saude)[238] | 2020-08-31 - 2020-10-12 | Local | Moderate | 1,946 | Multiple groups | All | Probability | Household and community samples | 0.7% | Qingdao Hightop Biotech Co. Ltd - IgG, IgM - Hightop COVID-19 IgM/IgG Ab Rapid Test Kit | 0.942, 0.939 | Validated by manufacturer |
| Mozambique - HRP (Pemba) | Paulo Arnaldo (República de Moçambique Ministério da Saúde)[239] | 2020-07-06 - 2020-07-13 | Local | Moderate | 1,360 | Multiple groups | All | Probability | Household and community samples | 2.5% | Qingdao Hightop Biotech Co. Ltd - IgG, IgM - Hightop COVID-19 IgM/IgG Ab Rapid Test Kit | 0.942, 0.939 | Validated by manufacturer |
| Mozambique - HRP (Nampula) | Eduardo Samo Gudo (Republica de Mocambique Ministerio da Saude)[240] | 2020-06-17 - 2020-06-30 | Local | Moderate | 1,769 | Multiple groups | All | Probability | Household and community samples | 5% | SD Biosensor - IgG, IgM - Standard Q COVID-19 IgM/IgG Duo rapid immunochromatography test kit | 0.991, 0.951 | Validated by manufacturer |
| Nigeria - HRP (Abuja, Enugu) | Ijeoma Ifeorah (Molecular Pathology Institute)[241] | 2020-08-01 - 2020-08-31 | Local | High | 113 | Adults (18-64 years) | All | Convenience | Blood donors | 42% | NovaTec Immundiagnostics GmbH - IgG, IgM - NovaLisa  SARS-CoV-2 IgG/IgM | 1, 0.98 | Validated by manufacturer |
| South Africa (Bushbuckridge Municipality) | Jackie Kleynhans (National Health Laboratory Service)[242] | 2021-11-15 - 2021-11-27 | Local | Moderate | 550 | Multiple groups | All | Probability | Household and community samples | 59.8% | Roche Diagnostics - IgG, IgM, IgA - Elecsys  Anti‐SARS‐CoV‐2 (N) | 1, 0.995 | Validated by manufacturer |
| South Africa (Matlosana Municipality) | Jackie Kleynhans (National Health Laboratory Service)[242] | 2021-11-15 - 2021-11-27 | Local | Moderate | 479 | Multiple groups | All | Probability | Household and community samples | 69.9% | Roche Diagnostics - IgG, IgM, IgA - Elecsys  Anti‐SARS‐CoV‐2 (N) | 1, 0.995 | Validated by manufacturer |
| South Africa | Russel Cable (Stellenbosch University)[243] | 2021-11-08 - 2021-11-12 | National | High | 3,395 | Multiple groups | All | Convenience | Blood donors | 71.1% | Roche Diagnostics - IgG, IgM, IgA - Elecsys  Anti‐SARS‐CoV‐2 (N) | 1, 0.995 | Validated by manufacturer |
| South Africa (Bushbuckridge Municipality) | Jackie Kleynhans (National Health Laboratory Service)[242] | 2021-09-13 - 2021-09-25 | Local | Moderate | 548 | Multiple groups | All | Probability | Household and community samples | 59.3% | Roche Diagnostics - IgG, IgM, IgA - Elecsys  Anti‐SARS‐CoV‐2 (N) | 1, 0.995 | Validated by manufacturer |
| South Africa (Matlosana Municipality) | Jackie Kleynhans (National Health Laboratory Service)[242] | 2021-09-13 - 2021-09-25 | Local | Moderate | 493 | Multiple groups | All | Probability | Household and community samples | 68.8% | Roche Diagnostics - IgG, IgM, IgA - Elecsys  Anti‐SARS‐CoV‐2 (N) | 1, 0.995 | Validated by manufacturer |
| South Africa (Bushbuckridge Municipality) | Jackie Kleynhans (National Health Laboratory Service)[242] | 2021-07-19 - 2021-08-05 | Local | Moderate | 578 | Multiple groups | All | Probability | Household and community samples | 38.9% | Roche Diagnostics - IgG, IgM, IgA - Elecsys  Anti‐SARS‐CoV‐2 (N) | 1, 0.995 | Validated by manufacturer |
| South Africa (Matlosana Municipality) | Jackie Kleynhans (National Health Laboratory Service)[242] | 2021-07-19 - 2021-08-05 | Local | Moderate | 499 | Multiple groups | All | Probability | Household and community samples | 55.7% | Roche Diagnostics - IgG, IgM, IgA - Elecsys  Anti‐SARS‐CoV‐2 (N) | 1, 0.995 | Validated by manufacturer |
| South Africa (Bushbuckridge Municipality) | Jackie Kleynhans (National Health Laboratory Service)[242] | 2021-05-20 - 2021-06-09 | Local | Moderate | 579 | Multiple groups | All | Probability | Household and community samples | 26.1% | Roche Diagnostics - IgG, IgM, IgA - Elecsys  Anti‐SARS‐CoV‐2 (N) | 1, 0.995 | Validated by manufacturer |
| South Africa (Matlosana Municipality) | Jackie Kleynhans (National Health Laboratory Service)[242] | 2021-05-20 - 2021-06-09 | Local | Moderate | 505 | Multiple groups | All | Probability | Household and community samples | 48.9% | Roche Diagnostics - IgG, IgM, IgA - Elecsys  Anti‐SARS‐CoV‐2 (N) | 1, 0.995 | Validated by manufacturer |
| South Africa (Hillbrow Community Healthcare Centre and Esselen Street Clinic, Region F, City of Johannesburg, Gauteng, South Africa) | Lee Fairlie (Wits Reproductive Health and HIV Institute)[244] | 2021-03-17 - 2021-06-09 | Local | High | 500 | Multiple groups | All | Convenience | Pregnant or parturient women | 64% (95% CI 59.6-68.2%) | Beijing Wantai Biological - IgG IgM IgA - Wantai SARS-CoV-2 Total Ab ELISA,Roche Diagnostics - IgG IgM IgA - Elecsys  Anti‐SARS‐CoV‐2 (N) | 1, 0.97 | Complex test algorithm: Manual calculation |
| South Africa (Agincourt) | Jackie Kleynhans (National Institute for Communicable Diseases)[245] | 2021-03-22 - 2021-04-11 | Local | Moderate | 586 | Multiple groups | All | Probability | Household and community samples | 25.3% (95% CI 21.8-29%) | Roche Diagnostics - IgG IgM IgA - Elecsys  Anti‐SARS‐CoV‐2 (N) | 1, 0.995 | Validated by manufacturer |
| South Africa (Klerksdorp) | Jackie Kleynhans (National Institute for Communicable Diseases)[245] | 2021-03-22 - 2021-04-11 | Local | Moderate | 505 | Multiple groups | All | Probability | Household and community samples | 40.4% (95% CI 36.1-44.8%) | Roche Diagnostics - IgG IgM IgA - Elecsys  Anti‐SARS‐CoV‐2 (N) | 1, 0.995 | Validated by manufacturer |
| South Africa (Agincourt) | Jackie Kleynhans (National Institute for Communicable Diseases)[245] | 2021-01-25 - 2021-02-21 | Local | Moderate | 586 | Multiple groups | All | Probability | Household and community samples | 21.5% (95% CI 18.1-24.9%) | Roche Diagnostics - IgG IgM IgA - Elecsys  Anti‐SARS‐CoV‐2 (N) | 1, 0.995 | Validated by manufacturer |
| South Africa (Klerksdorp) | Jackie Kleynhans (National Institute for Communicable Diseases)[245] | 2021-01-25 - 2021-02-21 | Local | Moderate | 509 | Multiple groups | All | Probability | Household and community samples | 35.4% (95% CI 31-39.5%) | Roche Diagnostics - IgG IgM IgA - Elecsys  Anti‐SARS‐CoV‐2 (N) | 1, 0.995 | Validated by manufacturer |
| South Africa (Agincourt) | Jackie Kleynhans (National Institute for Communicable Diseases)[245] | 2020-11-23 - 2020-12-12 | Local | Moderate | 566 | Multiple groups | All | Probability | Household and community samples | 7.1% (95% CI 5.1-9.5%) | Roche Diagnostics - IgG IgM IgA - Elecsys  Anti‐SARS‐CoV‐2 (N) | 1, 0.995 | Validated by manufacturer |
| South Africa (Klerksdorp) | Jackie Kleynhans (National Institute for Communicable Diseases)[245] | 2020-11-23 - 2020-12-12 | Local | Moderate | 523 | Multiple groups | All | Probability | Household and community samples | 27% (95% CI 23.2-31%) | Roche Diagnostics - IgG IgM IgA - Elecsys  Anti‐SARS‐CoV‐2 (N) | 1, 0.995 | Validated by manufacturer |
| South Africa (Agincourt) | Jackie Kleynhans (National Institute for Communicable Diseases)[245] | 2020-09-21 - 2020-10-10 | Local | Moderate | 490 | Multiple groups | All | Probability | Household and community samples | 4.9% (95% CI 3.2-7.2%) | Roche Diagnostics - IgG IgM IgA - Elecsys  Anti‐SARS‐CoV‐2 (N) | 1, 0.995 | Validated by manufacturer |
| South Africa (Klerksdorp) | Jackie Kleynhans (National Institute for Communicable Diseases)[245] | 2020-09-21 - 2020-10-10 | Local | Moderate | 498 | Multiple groups | All | Probability | Household and community samples | 21.7% (95% CI 18.1-25.6%) | Roche Diagnostics - IgG IgM IgA - Elecsys  Anti‐SARS‐CoV‐2 (N) | 1, 0.995 | Validated by manufacturer |
| South Africa (Agincourt) | Jackie Kleynhans (National Institute for Communicable Diseases)[245] | 2020-07-20 - 2020-09-17 | Local | Moderate | 439 | Multiple groups | All | Probability | Household and community samples | 1.1% (95% CI 0.4-2.6%) | Roche Diagnostics - IgG IgM IgA - Elecsys  Anti‐SARS‐CoV‐2 (N) | 1, 0.995 | Validated by manufacturer |
| South Africa (Klerksdorp) | Jackie Kleynhans (National Institute for Communicable Diseases)[245] | 2020-07-20 - 2020-09-17 | Local | Moderate | 501 | Multiple groups | All | Probability | Household and community samples | 14.4% (95% CI 11.4-17.8%) | Roche Diagnostics - IgG IgM IgA - Elecsys  Anti‐SARS‐CoV‐2 (N) | 1, 0.995 | Validated by manufacturer |
| South Africa (Cape Town) | Marvin Hsiao ( University of Cape Town)[246] | 2020-06-15 - 2020-08-07 | Local | High | 2,791 | Adults (18-64 years) | All | Sequential | Residual sera | 40.2% | Roche Diagnostics - IgG, IgM, IgA - Elecsys  Anti‐SARS‐CoV‐2 (N) | 1, 0.995 | Validated by manufacturer |
| South Sudan - HRP (Juba) | Kirsten E Wiens (World Health Organization)[247] | 2020-08-10 - 2020-09-11 | Local | Low | 2,214 | Multiple groups | All | Probability | Household and community samples | 22.3% | NA - IgG - Author designed (ELISA) -Spike | 0.655, 1 | Validated by developers |
| Zambia - HRP (Zambian Central Province) | Kwame Shanaube (Zambart)[248] | 2020-10-15 - 2021-03-15 | Local | Moderate | 2,977 | Multiple groups | All | Probability | Household and community samples | 13.5% | Abbott Laboratories - IgG - Abbott Architect SARS-CoV-2 IgG | 0.99, 1 | Validated by manufacturer |
| Zimbabwe - HRP (Harare) | Arun Fryatt (Biomedical Research and Training Institute)[249] | 2021-02-10 - 2021-04-17 | Local | Moderate | 1,530 | Multiple groups | All | Probability | Household and community samples | 53% | NA - - Author designed (type unknown) | 0.992, 0.9865 | Validated by developers |
| Zimbabwe - HRP (Harare) | Arun Fryatt (Biomedical Research and Training Institute)[249] | 2020-11-20 - 2020-12-20 | Local | Moderate | 620 | Multiple groups | All | Probability | Household and community samples | 19% | NA - - Author designed (type unknown) | 0.992, 0.9865 | Validated by developers |

#### Americas region

| Country (Location) | Author (Organization) | Sampling Dates (YMD) | Geographic scope | Overall risk of bias | Sample size | Age | Sex | Sampling method | Sample frame | Seroprevalence (95% CI) | Test Manufacturers - Isotypes - Names | Test Sens, Spec | Sens, Spec Source |
| --- | --- | --- | --- | --- | --- | --- | --- | --- | --- | --- | --- | --- | --- |
| Argentina - HRP (Quilmes) | Laura Munoz (Ministerio de Salud de la Provincia de Buenos Aires)[250] | 2020-07-15 - 2020-07-16 | Local | High | 284 | Multiple groups | All | Stratified non-probability | Household and community samples | 14.8% | COVIDAR - IgG - SEROKIT-ELISA COVIDAR IgG | 0.95, 1 | Validated by manufacturers |
| Argentina - HRP (Buenos Aires) | Silvana Figar (Ministry of Health of the City of Buenos Aires)[251] | 2020-06-10 - 2020-06-26 | Local | Low | 873 | Multiple groups | All | Probability | Persons living in slums | 53.4% | CONICET - IgG, IgM - COVIDAR IgM and IgG ELISA | 0.9, 1 | Validated by independent authors/third party/non-developers |
| Brazil - HRP (São Paulo) | Beatriz Tess (Inteligência em Pesquisa e Consultoria)[252] | 2021-04-22 - 2021-05-01 | Local | Moderate | 1,187 | Multiple groups | All | Probability | Household and community samples | 41.6% | Abbott Laboratories - IgG - Abbott Architect SARS-CoV-2 IgG,Roche Diagnostics - IgG, IgM, IgA - Elecsys  Anti‐SARS‐CoV‐2 (N) | 0.99, 1 | Complex test algorithm: Manual calculation |
| Brazil - HRP (Para) | Keise Pereira (Universidade Federal do Para)[253] | 2021-04-01 - 2021-04-30 | Local | High | 140 | Multiple groups | All | Convenience | Household and community samples | 52.1% | EUROIMMUN - IgG - Anti-SARS-CoV-2 ELISA IgG | 0.9, 1 | Validated by manufacturer |
| Brazil - HRP (São Paulo) | City of Sao Paulo (City of São Paulo)[254] | 2021-02-16 - 2021-02-18 | Local | Moderate | 1,793 | Multiple groups | All | Probability | Household and community samples | 25% | Guangzhou Wondfo Biotech Co. Ltd - IgG, IgM - Finecare SARS-CoV-2 Antibody test,EUROIMMUN - IgG - Anti-SARS-CoV-2 ELISA IgG | 0.987, 0.991 | Complex test algorithm: Manual calculation |
| Brazil - HRP (Salvador) | Mariam Fofana (Yale School of Public Health)[255] | 2020-11-18 - 2021-02-26 | Local | Moderate | 2,035 | Multiple groups | All | Probability | Persons living in slums | 46.4% | EUROIMMUN - IgG - Anti-SARS-CoV-2 ELISA IgG | 0.9, 1 | Validated by manufacturer |
| Brazil - HRP (Belém) | Hilton Pereira da Silva (Universidade Federal do Pará)[256] | 2020-09-15 - 2021-01-15 | Local | Moderate | 101 | Multiple groups | All | Probability | Household and community samples | 83.2% | EUROIMMUN - IgG - Anti-SARS-CoV-2 ELISA IgG | 0.9, 1 | Validated by manufacturer |
| Brazil - HRP (São Paulo) | Joao Luiz Miraglia (Hospital Israelita Albert Einstein)[257] | 2020-09-15 - 2020-12-15 | Local | Moderate | 272 | Multiple groups | All | Probability | Household and community samples | 45.2% | Roche Diagnostics - IgG, IgM, IgA - Elecsys  Anti‐SARS‐CoV‐2 (N) | 1, 0.995 | Validated by manufacturer |
| Brazil - HRP (Manaus) | Gemilson Soares Pontes (Instituto Nacional de Pesquisas da Amazônia)[258] | 2020-10-10 - 2020-11-14 | Local | High | 280 | Multiple groups | All | Sequential | Household and community samples | 64.6% | EUROIMMUN - IgA - Anti-SARS-CoV-2 ELISA IgA,EUROIMMUN - IgG - Anti-SARS-CoV-2 ELISA IgG | 0.997, 0.983 | Complex test algorithm: Manual calculation |
| Brazil - HRP (Sao Paulo) | Lewis Buss (Universidade de Sao Paulo)[259] | 2020-10-12 - 2020-10-24 | Local | Moderate | 877 | Multiple groups | All | Sequential | Blood donors | 19.5% | Abbott Laboratories - IgG - Abbott Architect SARS-CoV-2 IgG | 0.99, 1 | Validated by manufacturer |
| Brazil - HRP (Manaus) | Lewis Buss (Universidade de Sao Paulo)[259] | 2020-10-10 - 2020-10-17 | Local | Moderate | 882 | Multiple groups | All | Sequential | Blood donors | 43.1% | Abbott Laboratories - IgG - Abbott Architect SARS-CoV-2 IgG | 0.99, 1 | Validated by manufacturer |
| Brazil - HRP (Sao Paulo) | Lewis Buss (Universidade de Sao Paulo)[259] | 2020-09-07 - 2020-09-29 | Local | Moderate | 933 | Multiple groups | All | Sequential | Blood donors | 16.7% | Abbott Laboratories - IgG - Abbott Architect SARS-CoV-2 IgG | 0.99, 1 | Validated by manufacturer |
| Brazil - HRP (Manaus) | Lewis Buss (Universidade de Sao Paulo)[259] | 2020-09-05 - 2020-09-14 | Local | Moderate | 868 | Multiple groups | All | Sequential | Blood donors | 45.4% | Abbott Laboratories - IgG - Abbott Architect SARS-CoV-2 IgG | 0.99, 1 | Validated by manufacturer |
| Brazil - HRP (Victória) | Carolina Ferrugini (Federal University of Espírito Santo)[260] | 2020-07-15 - 2020-10-15 | Local | High | 261 | Adults (18-64 years) | Female | Sequential | Pregnant or parturient women | 27.2% | ECO Diagnostica LTDA - IgG, IgM - COVID-19 IgG/IgM ECO Test | 0.971, 0.987 | Validated by manufacturer |
| Brazil - HRP (Sao Paulo) | Lewis Buss (Universidade de Sao Paulo)[259] | 2020-08-10 - 2020-08-29 | Local | Moderate | 906 | Multiple groups | All | Sequential | Blood donors | 17.2% | Abbott Laboratories - IgG - Abbott Architect SARS-CoV-2 IgG | 0.99, 1 | Validated by manufacturer |
| Brazil - HRP (Manaus) | Lewis Buss (Universidade de Sao Paulo)[259] | 2020-08-08 - 2020-08-19 | Local | Moderate | 881 | Multiple groups | All | Sequential | Blood donors | 44% | Abbott Laboratories - IgG - Abbott Architect SARS-CoV-2 IgG | 0.99, 1 | Validated by manufacturer |
| Brazil - HRP (Rio de Janeiro) | Pamella Lugon (Oswaldo Cruz Foundation)[261] | 2020-05-18 - 2020-09-24 | Local | High | 515 | Multiple groups | All | Convenience | Multiple general populations | 29.3% | Abbott Laboratories - IgG - Abbott Architect SARS-CoV-2 IgG | 0.99, 1 | Validated by manufacturer |
| Brazil - HRP (Sao Paulo) | Lewis Buss (Universidade de Sao Paulo)[259] | 2020-07-13 - 2020-07-25 | Local | Moderate | 879 | Multiple groups | All | Sequential | Blood donors | 12.8% | Abbott Laboratories - IgG - Abbott Architect SARS-CoV-2 IgG | 0.99, 1 | Validated by manufacturer |
| Brazil - HRP (Sao Paulo) | Luanda Mara da Silva Oliveira (Universidade de São Paulo)[262] | 2020-06-30 - 2020-08-04 | Local | High | 439 | Multiple groups | All | Convenience | Residual sera | 13.9% | DiaSorin - IgG - Liaison SARS-CoV-2 S1/S2 IgG,Roche Diagnostics - IgG, IgM, IgA - Elecsys  Anti‐SARS‐CoV‐2 (N) | 0.974, 1 | Complex test algorithm: Manual calculation |
| Brazil - HRP (Betim) | Ana Silva (Universidade Federal de Minas Gerais)[263] | 2020-07-13 - 2020-07-15 | Local | Moderate | 1,080 | Multiple groups | All | Probability | Household and community samples | 2.6% | Guangzhou Wondfo Biotech Co. Ltd - IgG, IgM - Finecare SARS-CoV-2 Antibody test | 0.872, 0.991 | Validated by manufacturer |
| Brazil - HRP (Joinville) | Henrique Diegoli (City Hall of Joinville)[264] | 2020-06-15 - 2020-08-07 | Local | Moderate | 4,403 | Multiple groups | All | Probability | Household and community samples | 4.8% | Celer Technologies Inc. - IgG, IgM - One Step COVID-19 Test | 0.8643, 0.9957 | Validated by independent authors/third party/non-developers |
| Brazil - HRP (Manaus) | Lewis Buss (Universidade de Sao Paulo)[259] | 2020-07-06 - 2020-07-15 | Local | Moderate | 1,147 | Multiple groups | All | Sequential | Blood donors | 46.9% | Abbott Laboratories - IgG - Abbott Architect SARS-CoV-2 IgG | 0.99, 1 | Validated by manufacturer |
| Brazil - HRP (Rio de Janeiro) | Alanna Calheiros Santos (Oswaldo Cruz Foundation)[265] | 2020-01-07 - 2021-01-11 | Local | High | 31 | Adults (18-64 years) | Female | Convenience | Pregnant or parturient women | 19.4% | Roche Diagnostics - IgG, IgM, IgA - Elecsys  Anti‐SARS‐CoV‐2 (N) | 1, 0.995 | Validated by manufacturer |
| Brazil - HRP (Carmópolis) | Breno Bernardes-Souza (Federal University of Ouro Preto)[266] | 2020-06-27 - 2020-06-28 | Local | Moderate | 400 | Multiple groups | All | Probability | Household and community samples | 1.8% | Qingdao Hightop Biotech Co. Ltd - IgG, IgM - Hightop COVID-19 IgM/IgG Ab Rapid Test Kit | 0.942, 0.939 | Validated by manufacturer |
| Brazil - HRP (Sao Paulo) | Lewis Buss (Universidade de Sao Paulo)[259] | 2020-06-08 - 2020-06-20 | Local | Moderate | 880 | Multiple groups | All | Sequential | Blood donors | 15% | Abbott Laboratories - IgG - Abbott Architect SARS-CoV-2 IgG | 0.99, 1 | Validated by manufacturer |
| Brazil - HRP (Ribeirão Preto (RP)) | Edson Zangiacomi Martinez (Universidade de São Paulo)[267] | 2020-06-11 - 2020-06-14 | Local | Moderate | 646 | Multiple groups | All | Probability | Household and community samples | 2.8% | Guangzhou Wondfo Biotech Co. Ltd - IgG, IgM, IgA - Wondfo SARS-CoV-2 Antibody Test | 0.864, 0.996 | Validated by manufacturer |
| Brazil - HRP (Manaus) | Lewis Buss (Universidade de Sao Paulo)[259] | 2020-06-05 - 2020-06-15 | Local | Moderate | 911 | Multiple groups | All | Sequential | Blood donors | 52.7% | Abbott Laboratories - IgG - Abbott Architect SARS-CoV-2 IgG | 0.99, 1 | Validated by manufacturer |
| Brazil - HRP (Nepomuceno) | Breno Bernardes-Souza (Federal University of Ouro Preto)[266] | 2020-05-30 - 2020-05-31 | Local | Moderate | 400 | Multiple groups | All | Probability | Household and community samples | 0.5% | Qingdao Hightop Biotech Co. Ltd - IgG, IgM - Hightop COVID-19 IgM/IgG Ab Rapid Test Kit | 0.942, 0.939 | Validated by manufacturer |
| Brazil - HRP (Sao Paulo) | Lewis Buss (Universidade de Sao Paulo)[259] | 2020-05-08 - 2020-05-21 | Local | Moderate | 826 | Multiple groups | All | Sequential | Blood donors | 7.5% | Abbott Laboratories - IgG - Abbott Architect SARS-CoV-2 IgG | 0.99, 1 | Validated by manufacturer |
| Brazil - HRP (Manaus) | Lewis Buss (Universidade de Sao Paulo)[259] | 2020-05-05 - 2020-05-14 | Local | Moderate | 901 | Multiple groups | All | Sequential | Blood donors | 42.1% | Abbott Laboratories - IgG - Abbott Architect SARS-CoV-2 IgG | 0.99, 1 | Validated by manufacturer |
| Brazil - HRP | Beatriz Tess (Universidade de Sao Paulo)[268] | 2020-05-04 - 2020-05-12 | Subnational | High | 463 | Multiple groups | All | Stratified non-probability | Multiple general populations | 6% | Snibe Co. Ltd (Shenzhen New Industries Biomedical Engineering Co. Ltd) - IgG, IgM - MAGLUMI 2019-nCoV IgM/IgG,Roche Diagnostics - IgG, IgM, IgA - Elecsys  Anti‐SARS‐CoV‐2 (N) | 1, 0.982 | Complex test algorithm: Manual calculation |
| Brazil - HRP (Ribeirão Preto (RP)) | Edson Zangiacomi Martinez (Universidade de São Paulo)[267] | 2020-05-01 - 2020-05-03 | Local | Moderate | 709 | Multiple groups | All | Probability | Household and community samples | 1.3% | Guangzhou Wondfo Biotech Co. Ltd - IgG, IgM, IgA - Wondfo SARS-CoV-2 Antibody Test | 0.864, 0.996 | Validated by manufacturer |
| Brazil - HRP (Sao Paulo) | Lewis Buss (Universidade de Sao Paulo)[259] | 2020-04-08 - 2020-04-30 | Local | Moderate | 900 | Multiple groups | All | Sequential | Blood donors | 6.8% | Abbott Laboratories - IgG - Abbott Architect SARS-CoV-2 IgG | 0.99, 1 | Validated by manufacturer |
| Brazil - HRP (Manaus) | Lewis Buss (Universidade de Sao Paulo)[259] | 2020-04-06 - 2020-04-17 | Local | Moderate | 829 | Multiple groups | All | Sequential | Blood donors | 7.7% | Abbott Laboratories - IgG - Abbott Architect SARS-CoV-2 IgG | 0.99, 1 | Validated by manufacturer |
| Brazil - HRP (Sao Paulo) | Lewis Buss (Universidade de Sao Paulo)[259] | 2020-03-09 - 2020-03-21 | Local | Moderate | 2,454 | Multiple groups | All | Sequential | Blood donors | 5.8% | Abbott Laboratories - IgG - Abbott Architect SARS-CoV-2 IgG | 0.99, 1 | Validated by manufacturer |
| Brazil - HRP (Manaus) | Lewis Buss (Universidade de Sao Paulo)[259] | 2020-03-06 - 2020-03-12 | Local | Moderate | 832 | Multiple groups | All | Sequential | Blood donors | 2.6% | Abbott Laboratories - IgG - Abbott Architect SARS-CoV-2 IgG | 0.99, 1 | Validated by manufacturer |
| Brazil - HRP (Aracaju) | Ricardo Queiroz Gurgel (Federal University of Sergipe)[269] | 2020-01-15 - 2020-04-15 | Local | High | 987 | Multiple groups | All | Sequential | Residual sera | 1.6% | NA - - Not reported/ Unable to specify,Boditech Med Inc. - IgG, IgM - Ichroma COVID-19 Ab Test | N/A | Complex test algorithm: Manual review |
| Brazil - HRP (Manaus) | Lewis Buss (Universidade de Sao Paulo)[259] | 2020-02-07 - 2020-02-13 | Local | Moderate | 821 | Multiple groups | All | Sequential | Blood donors | 3.6% | Abbott Laboratories - IgG - Abbott Architect SARS-CoV-2 IgG | 0.99, 1 | Validated by manufacturer |
| Brazil - HRP (Sao Paulo) | Lewis Buss (Universidade de Sao Paulo)[259] | 2020-02-08 - 2020-02-29 | Local | Moderate | 799 | Multiple groups | All | Sequential | Blood donors | 4.2% | Abbott Laboratories - IgG - Abbott Architect SARS-CoV-2 IgG | 0.99, 1 | Validated by manufacturer |
| Colombia - HRP (Mitú) | Hector Serrano-Coll (Universidad de Córdoba)[270] | 2020-12-01 - 2020-12-31 | Local | High | 589 | Multiple groups | All | Convenience | Household and community samples | 57.6% | Eurofins Ingenasa - IgG, IgM, IgA - INgezim COVID 19 DR | 0.983, 0.992 | Validated by manufacturer |
| Colombia - HRP (Monteria) | Salim Mattar (Universidad de Córdoba)[271] | 2020-08-04 - 2020-09-01 | Local | Low | 1,368 | Multiple groups | All | Probability | Household and community samples | 55.3% | Eurofins Ingenasa - IgG, IgM, IgA - INgezim COVID 19 DR | 0.983, 0.992 | Validated by manufacturer |
| Ecuador - HRP (Atahualpa) | Oscar Del Brutto (Universidad Espíritu Santo)[272] | 2020-05-01 - 2021-04-30 | Local | High | 673 | Adults (18-64 years) | All | Convenience | Household and community samples | 63.7% | Biohit Health Care Ltd - IgG, IgM - BIOHIT SARS-CoV-2 antibody test | 0.975, 1 | Validated by independent authors/third party/non-developers |
| Ecuador - HRP (Azuay) | David Acurio Paez (University of Antwerp)[273] | 2020-08-11 - 2020-11-01 | Local | Moderate | 2,457 | Multiple groups | All | Probability | Household and community samples | 13.2% | SD Biosensor - IgG, IgM - Standard Q COVID-19 IgM/IgG Duo rapid immunochromatography test kit | 0.991, 0.951 | Validated by manufacturer |
| French Guiana | Claude Flamand (Institut Pasteur in French Guiana)[274] | 2020-07-15 - 2020-07-23 | National | High | 480 | Multiple groups | All | Convenience | Residual sera | 13.1% | EUROIMMUN - IgG - Anti-SARS-CoV-2 ELISA IgG | 0.9, 1 | Validated by manufacturer |
| Jamaica (Kingston) | TeAnne Chisolm (University of the West Indies)[275] | 2020-11-01 - 2020-11-30 | Local | High | 77 | Adults (18-64 years) | Female | Sequential | Pregnant or parturient women | 24% | Abbott Laboratories - IgG - Abbott Architect SARS-CoV-2 IgG | 0.99, 1 | Validated by manufacturer |
| Mexico - HRP (Campeche) | Victor Monteon (Universidad Autonoma Campeche)[276] | 2021-08-15 - 2021-09-15 | Subnational | High | 479 | Multiple groups | All | Sequential | Blood donors | 69.1% | NA - - Author designed (ELISA) -Spike | 0.94, 1 | Validated by developers |
| Mexico - HRP | Jose Munoz-Medina (Instituto de Salud para el Bienestar)[277] | 2020-12-01 - 2020-12-31 | National | High | 5,359 | Multiple groups | All | Stratified non-probability | Residual sera | 30.6% | DiaSorin - IgG - Liaison SARS-CoV-2 S1/S2 IgG,GenScript - IgG, IgM - cPass SARS-CoV-2 Surrogate Virus Neutralization Test ELISA Kit | 0.914, 1 | Complex test algorithm: Manual calculation |
| Mexico - HRP (Veracruz City) | Jose Maria Remes-Troche (Universidad Veracruzana)[278] | 2020-06-01 - 2020-07-31 | Local | High | 2,174 | Multiple groups | All | Sequential | Residual sera | 29.5% | Abbott Laboratories - IgG - Abbott Architect SARS-CoV-2 IgG | 0.99, 1 | Validated by manufacturer |
| Mexico - HRP (Nuevo Leon) | Natalia Martinez-Acu a (Autonomous University of Nuevo Leon)[279] | 2020-01-01 - 2020-12-15 | Subnational | High | 2,068 | Adults (18-64 years) | All | Sequential | Blood donors | 4.8% | Abbott Laboratories - IgG - Abbott Architect SARS-CoV-2 IgG | 0.99, 1 | Validated by manufacturer |
| Peru - HRP (La Riconanda) | Benoit Champigneulle (Grenoble Alpes University)[280] | 2020-10-11 - 2020-10-18 | Local | High | 159 | Multiple groups | All | Sequential | Household and community samples | 48.4% | AAZ LMB - IgG, IgM - COVID-PRESTO | 1, 1 | Validated by independent authors/third party/non-developers |
| Peru - HRP (Jaen) | Yordi Tarazona-Castro (Universidad Peruana de Ciencias Aplicadas)[281] | 2020-04-15 - 2021-03-15 | Local | High | 464 | Multiple groups | All | Sequential | Representative patient population | 35.8% | ImmunoDiagnostics - IgG, IgM - SARS-CoV-2 NP IgG/IgM ELISA Kit | 0.925, 0.933 | Validated by manufacturer |
| Peru - HRP (Iquitos) | Carlos Alvarez-Antonio (Universidad de Ingeniería y Tecnología)[282] | 2020-08-13 - 2020-08-18 | Local | Low | 621 | Multiple groups | All | Probability | Household and community samples | 68% | Zhejiang Orient Gene Biotech - IgG, IgM - COVID-19 IgG/IgM Rapid Test | 0.972, 1 | Validated by manufacturer |
| Peru - HRP (Iquitos) | Carlos Alvarez-Antonio (Universidad de Ingeniería y Tecnología)[282] | 2020-07-13 - 2020-07-18 | Local | Low | 716 | Multiple groups | All | Probability | Household and community samples | 73.7% | Zhejiang Orient Gene Biotech - IgG, IgM - COVID-19 IgG/IgM Rapid Test | 0.972, 1 | Validated by manufacturer |
| Peru - HRP (Lima metropolitan area) | Mary F Reyes Vega (Peruvian Ministry of Health)[283] | 2020-06-28 - 2020-07-09 | Local | Low | 3,212 | Multiple groups | All | Probability | Household and community samples | 21.4% | SD Biosensor - IgG, IgM - Standard Q COVID-19 IgM/IgG Duo rapid immunochromatography test kit | 0.991, 0.951 | Validated by manufacturer |
| Peru - HRP (Lima) | Enrique Guevara Rios (National Maternal Perinatal Institut)[284] | 2020-04-15 - 2020-05-15 | Local | Moderate | 2,419 | Adults (18-64 years) | Female | Sequential | Pregnant or parturient women | 7% | NA - - Not reported/ Unable to specify | 0.976, 0.988 | Validated by manufacturers |
| Canada (Vancouver) | David Goldfarb (University of British Colombia)[285] | 2021-01-01 - 2021-05-31 | Local | High | 5,417 | Adults (18-64 years) | All | Convenience | Blood donors | 2.6% | Roche Diagnostics - IgG, IgM, IgA - Elecsys  Anti‐SARS‐CoV‐2 (N) | 1, 0.995 | Validated by manufacturer |
| Canada | Xuyang Tang (University of Toronto)[286] | 2020-12-01 - 2021-03-31 | National | High | 6,955 | Multiple groups | All | Convenience | Household and community samples | 6.5% | NA - IgG - Author designed (ELISA) -Spike,NA - IgG - Author designed (ELISA) - Nucleocapsid | N/A | Complex test algorithm: Manual review |
| Canada | Ashleigh Tuite (Canadian Blood Services)[287] | 2020-04-15 - 2021-03-15 | National | High | 17,999 | Multiple groups | All | Convenience | Blood donors | 2.1% | Abbott Laboratories - IgG - Abbott Architect SARS-CoV-2 IgG,NA - IgG - Author designed (ELISA) - Nucleocapsid,NA - IgG - Author designed (ELISA) -Spike | N/A | Complex test algorithm: Manual review |
| Canada (Edmonton) | Emilie Manny (University of Alberta)[288] | 2020-08-14 - 2020-10-23 | Local | High | 565 | Children and Youth (0-17 years) | All | Convenience | Household and community samples | 1.6% | Abbott Laboratories - IgG - Abbott Architect SARS-CoV-2 IgG | 0.99, 1 | Validated by manufacturer |
| Canada (Québec) | Hema-Quebec (Héma-Québec)[289] | 2020-05-25 - 2020-07-09 | Subnational | High | 7,691 | Adults (18-64 years) | All | Convenience | Blood donors | 2.2% | NA - IgG, IgM, IgA - Author designed (ELISA) -Spike | 0.989, 0.985 | Validated by developers |
| Canada (British Columbia) | Guadalein Tanunliong (University of British Columbia)[290] | 2020-03-15 - 2020-05-31 | Subnational | High | 488 | Multiple groups | All | Probability | Residual sera | 1.8% | Meso Scale Discovery - IgG - Meso Scale Discovery multiplex assay | 1, 1 | Validated by independent authors/third party/non-developers |
| Panama - HRP (Panama City, Colon) | Alcibiades Villarreal (Instituto de Investigaciones Científicas y Servicios de Alta Tecnología)[291] | 2020-04-30 - 2020-07-07 | Local | High | 255 | Multiple groups | All | Convenience | Blood donors | 13.3% | NA - IgG, IgM - Author designed (ELISA) - Nucleocapsid | 0.8701, 0.9889 | Validated by developers |
| United States of America | Kristie Clarke (Centers for Disease Control and Prevention (CDC))[292] | 2022-02-01 - 2022-02-28 | National | High | 45,810 | Multiple groups | All | Convenience | Household and community samples | 57.7% | Roche Diagnostics - IgG, IgM, IgA - Elecsys  Anti‐SARS‐CoV‐2 (N) | 1, 0.995 | Validated by manufacturer |
| United States of America | Kristie Clarke (US Center for Disease Control)[293] | 2021-09-15 - 2022-02-15 | National | High | 71,490 | Children and Youth (0-17 years) | All | Stratified non-probability | Residual sera | 52% | Roche Diagnostics - IgG, IgM, IgA - Elecsys  Anti‐SARS‐CoV‐2 (N) | 1, 0.995 | Validated by manufacturer |
| United States of America (New Jersey) | Alexia Couture (Centers for Disease Control and Prevention)[150] | 2021-05-01 - 2021-05-31 | Subnational | High | 393 | Children and Youth (0-17 years) | All | Convenience | Residual sera | 44% | Roche Diagnostics - IgG, IgM, IgA - Elecsys  Anti‐SARS‐CoV‐2 (N) | 1, 0.995 | Validated by manufacturer |
| United States of America (Nevada) | Alexia Couture (Centers for Disease Control and Prevention)[150] | 2021-05-01 - 2021-05-31 | Subnational | High | 237 | Children and Youth (0-17 years) | All | Convenience | Residual sera | 30.4% | Roche Diagnostics - IgG, IgM, IgA - Elecsys  Anti‐SARS‐CoV‐2 (N) | 1, 0.995 | Validated by manufacturer |
| United States of America (Tennessee) | Alexia Couture (Centers for Disease Control and Prevention)[150] | 2021-05-01 - 2021-05-31 | Subnational | High | 192 | Children and Youth (0-17 years) | All | Convenience | Residual sera | 36.5% | Roche Diagnostics - IgG, IgM, IgA - Elecsys  Anti‐SARS‐CoV‐2 (N) | 1, 0.995 | Validated by manufacturer |
| United States of America (California) | Neeraj Sood (University of Southern California)[294] | 2021-04-09 - 2021-04-25 | Local | Low | 1,335 | Multiple groups | All | Probability | Household and community samples | 66.9% | Luminex Corporation - IgG - xMAP  SARS‑CoV‑2 Multi-Antigen IgG Assay | 0.981, 0.992 | Validated by manufacturer |
| United States of America (San Francisco) | Isobel Routledge (University of California San Francisco)[295] | 2021-02-04 - 2021-02-17 | Local | High | 915 | Multiple groups | All | Stratified non-probability | Residual sera | 28.4% | Ortho Clinical Diagnostics Inc. - IgG, IgM, IgA - VITROS Immunodiagnostic Products Anti-SARS-CoV-2 Total | 1, 1 | Validated by manufacturer |
| United States of America (Pittsburgh) | Lingqing Xu (University of Pittsburgh)[296] | 2021-02-01 - 2021-02-05 | Local | High | 194 | Multiple groups | All | Sequential | Residual sera | 16.5% | NA - - Author designed (Neutralization Assay),NA - IgG - Author designed (ELISA) - Nucleocapsid | N/A | Complex test algorithm: Manual review |
| United States of America (St Louis) | Brittany K. Smith (Washington University School of Medicine)[297] | 2020-12-24 - 2021-01-21 | Local | High | 1,001 | Multiple groups | All | Convenience | Residual sera | 20.7% | NA - IgG - Author designed (ELISA) -Spike | 0.982, 0.987 | Validated by developers |
| United States of America (Seattle) | Sylvia LaCourse (University of Washington)[298] | 2020-09-09 - 2021-05-07 | Local | Moderate | 1,304 | Adults (18-64 years) | Female | Convenience | Pregnant or parturient women | 4.8% | Abbott Laboratories - IgG - Abbott Architect SARS-CoV-2 IgG | 0.99, 1 | Validated by manufacturer |
| United States of America (Illinois) | Ryan Kilpatrick (University of Texas Medical Branch)[299] | 2020-11-15 - 2021-01-15 | Local | High | 1,008 | Multiple groups | All | Convenience | Household and community samples | 4.5% | Abbott Laboratories - IgG - Abbott Architect SARS-CoV-2 IgG | 0.99, 1 | Validated by manufacturer |
| United States of America | Robert Stout (Clinical Reference Laboratory Inc.)[300] | 2020-12-01 - 2020-12-06 | National | High | 14,605 | Multiple groups | All | Convenience | Residual sera | 10.4% | Roche Diagnostics - IgG, IgM, IgA - Elecsys  Anti‐SARS‐CoV‐2 (N) | 1, 0.995 | Validated by manufacturer |
| United States of America | Roger Dodd (American Red Cross)[301] | 2020-11-29 - 2020-11-30 | National | High | 13,268 | Multiple groups | All | Sequential | Blood donors | 9.7% | Ortho Clinical Diagnostics Inc. - IgG, IgM, IgA - VITROS Immunodiagnostic Products Anti-SARS-CoV-2 Total | 1, 1 | Validated by manufacturer |
| United States of America (St Louis) | Brittany K. Smith (Washington University School of Medicine)[297] | 2020-10-27 - 2020-11-30 | Local | High | 1,013 | Multiple groups | All | Convenience | Residual sera | 11.7% | NA - IgG - Author designed (ELISA) -Spike | 0.982, 0.987 | Validated by developers |
| United States of America (New York) | Cara Staszewski (North Shore University Hospital)[302] | 2020-10-15 - 2020-11-15 | Local | High | 149 | Adults (18-64 years) | Female | Sequential | Pregnant or parturient women | 14.1% | Roche Diagnostics - IgG, IgM, IgA - Elecsys  Anti‐SARS‐CoV‐2 (S) | 0.988, 1 | Validated by manufacturer |
| United States of America (Pennsylvania) | Callum Arnold (Pennsylvania State University)[303] | 2020-08-07 - 2020-12-20 | Local | High | 345 | Adults (18-64 years) | All | Convenience | Household and community samples | 8.2% | NA - IgG - Author designed (ELISA) -Spike | 0.933, 0.99 | Validated by independent authors/third party/non-developers |
| United States of America (Arizona) | Megan Jehn (Arizona State University)[304] | 2020-09-12 - 2020-10-01 | Local | Moderate | 260 | Multiple groups | All | Probability | Household and community samples | 11.5% | Roche Diagnostics - IgG, IgM, IgA - Elecsys  Anti‐SARS‐CoV‐2 (S) | 0.988, 1 | Validated by manufacturer |
| United States of America (New Mexico) | Fabrizio Bonelli (Dia Sorin Inc)[305] | 2020-09-01 - 2020-09-30 | Subnational | High | 1,931 | Multiple groups | All | Sequential | Residual sera | 2% | DiaSorin - IgG - Liaison SARS-CoV-2 S1/S2 IgG,Beckman Coulter - IgG - Access SARS-CoV-2 IgG Antibody Test | 1, 0.983 | Complex test algorithm: Manual calculation |
| United States of America (New York) | Fabrizio Bonelli (Dia Sorin Inc)[305] | 2020-09-01 - 2020-09-30 | Subnational | High | 1,491 | Multiple groups | All | Sequential | Residual sera | 24.4% | Siemens - IgG, IgM, IgA - ADVIA Centaur Immunoassay System,DiaSorin - IgG - Liaison SARS-CoV-2 S1/S2 IgG | N/A | Complex test algorithm: Manual review |
| United States of America | Robert Stout (Clinical Reference Laboratory Inc)[306] | 2020-09-01 - 2020-09-30 | National | High | 61,910 | Multiple groups | All | Sequential | Household and community samples | 6.6% | Roche Diagnostics - IgG, IgM, IgA - Elecsys  Anti‐SARS‐CoV‐2 (N) | 1, 0.995 | Validated by manufacturer |
| United States of America (St. Louis) | Sumanth Gandra (Washington University School of Medicine)[307] | 2020-09-01 - 2020-09-30 | Local | High | 226 | Multiple groups | All | Convenience | Residual sera | 8.4% | Abbott Laboratories - IgG - Abbott Architect SARS-CoV-2 IgG | 0.99, 1 | Validated by manufacturer |
| United States of America (Houston) | Elaine Symanski (Houston Health Department)[308] | 2020-09-01 - 2020-09-19 | Local | Low | 685 | Multiple groups | All | Probability | Household and community samples | 11.5% | Bio-rad - IgG, IgM, IgA - Platelia SARS-CoV-2 Total Ab assay,NA - IgG - Author designed (ELISA) -Spike | N/A | Complex test algorithm: Manual review |
| United States of America (Washington D.C) | Burak Bahar (Children’s National Hospital)[309] | 2020-07-07 - 2020-10-29 | Local | High | 385 | Children and Youth (0-17 years) | All | Convenience | Residual sera | 9.9% | DiaSorin - IgG - Liaison SARS-CoV-2 S1/S2 IgG | 0.974, 0.985 | Validated by manufacturer |
| United States of America (Denver) | Xiaofan Jia (University of Colorado)[310] | 2020-07-08 - 2020-10-23 | Local | High | 102 | Adults (18-64 years) | All | Convenience | Household and community samples | 3.9% | NA - - Author designed (CLIA) | 1, 0.999 | Validated by independent authors/third party/non-developers |
| United States of America (Denver) | Xiaofan Jia (University of Colorado)[310] | 2020-07-08 - 2020-10-23 | Local | High | 562 | Children and Youth (0-17 years) | All | Convenience | Household and community samples | 2.8% | NA - - Author designed (CLIA) | 1, 0.999 | Validated by independent authors/third party/non-developers |
| United States of America (New York City) | Teresa Janevic (University of Pennsylvania)[311] | 2020-04-15 - 2020-12-15 | Local | Moderate | 967 | Adults (18-64 years) | Female | Convenience | Pregnant or parturient women | 20% | NA - - Author designed (ELISA) -Spike | 0.95, 1 | Validated by developers |
| United States of America (Washington DC) | Adrienne Sherman (Government of the District of Columbia)[312] | 2020-07-27 - 2020-08-21 | Local | High | 671 | Multiple groups | All | Convenience | Household and community samples | 7.6% | DiaSorin - IgG - Liaison SARS-CoV-2 S1/S2 IgG | 0.974, 0.985 | Validated by manufacturer |
| United States of America (New York City) | Jannae Parrott (New York City Department of Health and Mental Hygiene)[313] | 2020-06-01 - 2020-10-09 | Local | Low | 1,074 | Multiple groups | All | Probability | Household and community samples | 24.3% | DiaSorin - IgG - Liaison SARS-CoV-2 S1/S2 IgG | 0.974, 0.985 | Validated by manufacturer |
| United States of America (St Louis) | Brittany K. Smith (Washington University School of Medicine)[297] | 2020-07-22 - 2020-08-16 | Local | High | 1,052 | Multiple groups | All | Convenience | Residual sera | 5.9% | NA - IgG - Author designed (ELISA) -Spike | 0.982, 0.987 | Validated by developers |
| United States of America (California) | Tim Bruckner (University of California Irvine)[314] | 2020-07-10 - 2020-08-16 | Local | Low | 2,979 | Multiple groups | All | Probability | Household and community samples | 11.5% | NA - IgG, IgM - Author designed (IFA) - MULTIPLEXED | 0.94, 1 | Validated by independent authors/third party/non-developers |
| United States of America (Baton Rouge) | Amy K Feehan (Louisiana State University)[315] | 2020-07-15 - 2020-07-31 | Local | Low | 2,138 | Multiple groups | All | Probability | Household and community samples | 3.6% | Abbott Laboratories - IgG - Abbott Architect SARS-CoV-2 IgG | 0.99, 1 | Validated by manufacturer |
| United States of America (Fullerton) | Jason Yamaki (St. Jude Medical Center)[316] | 2020-07-15 - 2020-07-27 | Local | High | 865 | Multiple groups | All | Sequential | Residual sera | 9.4% | Abbott Laboratories - IgG - Abbott Architect SARS-CoV-2 IgG | 0.99, 1 | Validated by manufacturer |
| United States of America (Lantana, West Palm Beach, Delray Beach, Belle Glade) | Charles Gonik (Wayne State University School of Medicine)[317] | 2020-06-29 - 2020-08-05 | Local | Moderate | 618 | Adults (18-64 years) | Female | Convenience | Pregnant or parturient women | 26.4% | Abbott Laboratories - IgG - Abbott Architect SARS-CoV-2 IgG | 0.99, 1 | Validated by manufacturer |
| United States of America (Iowa) | Haley Steffen (University of Iowa)[318] | 2020-05-01 - 2020-09-22 | Local | Moderate | 1,000 | Adults (18-64 years) | Female | Sequential | Pregnant or parturient women | 5.8% | DiaSorin - IgG - Liaison SARS-CoV-2 S1/S2 IgG,Roche Diagnostics - IgG, IgM, IgA - Elecsys  Anti‐SARS‐CoV‐2 (N),EUROIMMUN - IgG - Anti-SARS-CoV-2 ELISA IgG | N/A | Complex test algorithm: Manual review |
| United States of America (New York City) | Nina Molenaar ( Icahn School of Medicine)[319] | 2020-04-20 - 2020-09-22 | Local | High | 708 | Adults (18-64 years) | Female | Convenience | Pregnant or parturient women | 16.4% | NA - - Author designed (ELISA) -Spike | 0.95, 1 | Validated by developers |
| United States of America (New York City) | Daniel Stadlbauer (Icahn School of Medicine at Mount Sinai)[320] | 2020-06-29 - 2020-07-05 | Local | High | 311 | Multiple groups | All | Sequential | Residual sera | 22.2% | NA - IgG - Author designed (ELISA) -Spike | 0.95, 1 | Validated by developers |
| United States of America (New York City) | Daniel Stadlbauer (Icahn School of Medicine at Mount Sinai)[320] | 2020-06-29 - 2020-07-05 | Local | High | 182 | Multiple groups | All | Sequential | Residual sera | 23.1% | NA - IgG - Author designed (ELISA) -Spike | 0.95, 1 | Validated by developers |
| United States of America | McKaylee Robertson (City University of New York)[321] | 2020-05-15 - 2020-08-15 | National | High | 4,459 | Multiple groups | All | Convenience | Household and community samples | 6.8% | Bio-rad - IgG, IgM, IgA - Platelia SARS-CoV-2 Total Ab assay | 0.993, 0.98 | Validated by manufacturer |
| United States of America (New York City) | Kathy Kamath (Serimmune)[322] | 2020-06-24 - 2020-07-07 | Local | High | 69 | Multiple groups | All | Sequential | Blood donors | 11.6% | Serimmune - IgG, IgM, IgA - Serum Epitope Repertoire Analysis (SERA) | 0.91, 0.993 | Validated by manufacturers |
| United States of America (pennsylvania) | Glenn J. Rapsinski (UPMC Children’s Hospital of Pittsburgh)[323] | 2020-06-22 - 2020-07-03 | Subnational | High | 1,196 | Children and Youth (0-17 years) | All | Sequential | Residual sera | 0.9% | EUROIMMUN - IgG - Anti-SARS-CoV-2 ELISA IgG,Beckman Coulter - IgG - Access SARS-CoV-2 IgG Antibody Test,Siemens - IgG, IgM, IgA - ADVIA Centaur Immunoassay System | N/A | Complex test algorithm: Manual review |
| United States of America (Long Island) | Olga Morozova (Stony Brook University)[324] | 2020-03-25 - 2020-09-18 | Local | High | 1,042 | Multiple groups | All | Convenience | Household and community samples | 21% | Abbott Laboratories - IgG - Abbott Architect SARS-CoV-2 IgG | 0.99, 1 | Validated by manufacturer |
| United States of America (California) | Travis Lim (Centers for Disease and Control )[162] | 2020-03-23 - 2020-08-14 | Local | Low | 4,980 | Multiple groups | All | Probability | Residual sera | 1.9% | NA - IgG - Author designed (ELISA) -Spike | 0.96, 0.993 | Validated by developers |
| United States of America (New York City) | Travis Lim (Centers for Disease and Control )[162] | 2020-03-23 - 2020-08-14 | Local | Low | 7,746 | Multiple groups | All | Probability | Residual sera | 17.2% | NA - IgG, IgM, IgA - Author designed (ELISA) -Spike | 0.96, 0.993 | Validated by developers |
| United States of America (Philadelphia) | Travis Lim (Centers for Disease and Control )[162] | 2020-03-23 - 2020-08-14 | Local | Low | 8,657 | Multiple groups | All | Probability | Residual sera | 4.3% | NA - IgG, IgM, IgA - Author designed (ELISA) -Spike | 0.96, 0.993 | Validated by developers |
| United States of America (North Carolina) | Amir Barzin (University of North Carolina Chapel Hill)[325] | 2020-06-01 - 2020-06-04 | Subnational | High | 1,449 | Adults (18-64 years) | All | Convenience | Residual sera | 0% | Abbott Laboratories - IgG - Abbott Architect SARS-CoV-2 IgG | 0.99, 1 | Validated by manufacturer |
| United States of America (Pittsburgh) | Daniel Geisler (University of Pittsburgh School of Medicine)[326] | 2020-04-27 - 2020-07-04 | Local | High | 2,338 | Children and Youth (0-17 years) | All | Sequential | Residual sera | 3.6% | EUROIMMUN - IgG - Anti-SARS-CoV-2 ELISA IgG | 0.9, 1 | Validated by manufacturer |
| United States of America (New York) | Josh Reifer (Sherman Abrams Laboratory )[327] | 2020-05-15 - 2020-06-15 | Local | High | 28,523 | Multiple groups | All | Convenience | Residual sera | 44% | DiaSorin - IgG - Liaison SARS-CoV-2 S1/S2 IgG | 0.974, 0.985 | Validated by manufacturer |
| United States of America (New York) | Cara Staszewski (North Shore University Hospital)[302] | 2020-05-15 - 2020-06-15 | Local | High | 149 | Adults (18-64 years) | Female | Sequential | Pregnant or parturient women | 16.1% | Gold Standard Diagnostic - IgG - SARS-CoV-2 IgG ELISA Assay | 1, 1 | Validated by manufacturer |
| United States of America | Robert Stout (Clinical Reference Laboratory Inc.)[300] | 2020-05-12 - 2020-06-15 | National | High | 50,072 | Multiple groups | All | Convenience | Residual sera | 3% | Roche Diagnostics - IgG, IgM, IgA - Elecsys  Anti‐SARS‐CoV‐2 (N) | 1, 0.995 | Validated by manufacturer |
| United States of America (Long Island) | Minesh Khatri (New York University Long Island School of Medicine)[328] | 2020-02-01 - 2020-08-25 | Local | High | 343 | Multiple groups | All | Convenience | Residual sera | 25.7% | Abbott Laboratories - IgG - Abbott Architect SARS-CoV-2 IgG | 0.99, 1 | Validated by manufacturer |
| United States of America (Louisiana) | Amy K Feehan (Oschner Clinic Foundation)[329] | 2020-05-09 - 2020-05-15 | Local | Low | 2,640 | Adults (18-64 years) | All | Probability | Household and community samples | 6.9% | Abbott Laboratories - IgG - Abbott Architect SARS-CoV-2 IgG | 0.99, 1 | Validated by manufacturer |
| United States of America (San Francisco) | Isobel Routledge (University of California San Francisco)[330] | 2020-03-28 - 2020-06-26 | Local | Moderate | 4,735 | Multiple groups | All | Stratified non-probability | Residual sera | 4.1% | NA - - Author designed (Luminex),NA - IgG - Author designed (ELISA) -Unknown | N/A | Complex test algorithm: Manual review |
| United States of America (pennsylvania) | Glenn J. Rapsinski (UPMC Children’s Hospital of Pittsburgh)[323] | 2020-04-27 - 2020-05-19 | Subnational | High | 1,142 | Children and Youth (0-17 years) | All | Sequential | Residual sera | 0.5% | EUROIMMUN - IgG - Anti-SARS-CoV-2 ELISA IgG,Beckman Coulter - IgG - Access SARS-CoV-2 IgG Antibody Test,Siemens - IgG, IgM, IgA - ADVIA Centaur Immunoassay System | N/A | Complex test algorithm: Manual review |
| United States of America (Philadelphia) | Dustin Flannery (University of Pennsylvania)[331] | 2020-04-04 - 2020-06-03 | Local | Moderate | 1,293 | Adults (18-64 years) | Female | Sequential | Pregnant or parturient women | 6.2% | NA - IgG, IgM - Author designed (ELISA) -Spike | 1, 0.989 | Validated by independent authors/third party/non-developers |
| United States of America (St Louis) | Brittany Smith (Washington University School of Medicine)[332] | 2020-04-27 - 2020-05-12 | Local | High | 503 | Multiple groups | All | Sequential | Residual sera | 4.2% | NA - IgG - Author designed (ELISA) -Spike | 0.982, 0.987 | Validated by independent authors/third party/non-developers |
| United States of America (Rhode Island) | Daniel Nesbitt (Lindsley F Kimball Research Institute)[333] | 2020-04-27 - 2020-05-11 | Subnational | High | 1,996 | Multiple groups | All | Sequential | Blood donors | 0.7% | Ortho Clinical Diagnostics Inc. - IgG - VITROS Immunodiagnostic Products Anti-SARS-CoV-2 IgG | 1, 1 | Validated by manufacturer |
| United States of America (Georgia) | Holly Biggs (CDC COVID-19 Response Team)[334] | 2020-04-28 - 2020-05-03 | Local | Low | 696 | Multiple groups | All | Probability | Household and community samples | 2.7% | Ortho Clinical Diagnostics Inc. - IgG, IgM, IgA - VITROS Immunodiagnostic Products Anti-SARS-CoV-2 Total | 1, 1 | Validated by manufacturer |
| United States of America (Atlanta) | Kristina Bajema (Centers for Disease Control and Prevention)[335] | 2020-04-27 - 2020-05-01 | Local | High | 1,343 | Multiple groups | All | Convenience | Residual sera | 4.2% | NA - IgG, IgM, IgA - Author designed (ELISA) -Spike | 0.96, 0.993 | Validated by developers |
| United States of America (St Louis) | Brittany Smith (Washington University School of Medicine)[332] | 2020-04-14 - 2020-05-08 | Local | High | 555 | Children and Youth (0-17 years) | All | Sequential | Residual sera | 2.7% | NA - IgG - Author designed (ELISA) -Spike | 0.982, 0.987 | Validated by independent authors/third party/non-developers |
| United States of America (Baltimore) | Oliver Laeyendecker (Johns Hopkins University )[336] | 2020-03-16 - 2020-05-31 | Local | High | 3,255 | Multiple groups | All | Sequential | Residual sera | 3.5% | Hangzhou Biotest Biotech Co. Ltd - IgG, IgM - RightSign COVID-19 IgG/IgM Rapid Test Cassette,EUROIMMUN - IgG - Anti-SARS-CoV-2 ELISA IgG | 0.991, 1 | Complex test algorithm: Manual calculation |
| United States of America (California) | Neeraj Sood (Stanford University School of Medicine)[337] | 2020-04-10 - 2020-04-14 | Local | Low | 863 | Multiple groups | All | Probability | Household and community samples | 4.3% | Premier Biotech - IgG, IgM - COVID-19 IgG/IgM Rapid Test Cassette | 1, 1 | Validated by manufacturer |
| United States of America (Seattle) | Jennifer Rathe (University of Washington)[338] | 2020-03-26 - 2020-04-15 | Local | High | 100 | Multiple groups | All | Probability | Household and community samples | 4% | NA - - Author designed (Neutralization Assay),Abbott Laboratories - IgG - Abbott Architect SARS-CoV-2 IgG,NA - IgG, IgM, IgA - Author designed (ELISA) -Spike | N/A | Complex test algorithm: Manual review |
| United States of America (California) | Eran Bendavid (Stanford University School of Medicine)[339] | 2020-04-03 - 2020-04-04 | Local | High | 3,328 | Multiple groups | All | Stratified non-probability | Household and community samples | 1.5% | Premier Biotech - IgG, IgM - COVID-19 IgG/IgM Rapid Test Cassette | 1, 1 | Validated by manufacturer |
| United States of America (Seattle) | Adam S Dingens (Fred Hutchinson Cancer Research Center)[340] | 2020-03-03 - 2020-04-24 | Local | High | 1,076 | Children and Youth (0-17 years) | All | Convenience | Residual sera | 0.9% | Abbott Laboratories - IgG - Abbott Architect SARS-CoV-2 IgG,NA - IgG - Author designed (ELISA) -Spike | N/A | Complex test algorithm: Manual review |
| United States of America (San Francisco) | Dianna Ng (University of California, San Francisco)[341] | 2020-03-01 - 2020-03-31 | Local | High | 1,000 | Multiple groups | All | Sequential | Blood donors | 0.1% | Abbott Laboratories - IgG - Abbott Architect SARS-CoV-2 IgG,Ortho Clinical Diagnostics Inc. - IgG - VITROS Immunodiagnostic Products Anti-SARS-CoV-2 IgG,NA - - Author designed (Neutralization Assay) | N/A | Complex test algorithm: Manual review |

#### Eastern Mediterranean region

| Country (Location) | Author (Organization) | Sampling Dates (YMD) | Geographic scope | Overall risk of bias | Sample size | Age | Sex | Sampling method | Sample frame | Seroprevalence (95% CI) | Test Manufacturers - Isotypes - Names | Test Sens, Spec | Sens, Spec Source |
| --- | --- | --- | --- | --- | --- | --- | --- | --- | --- | --- | --- | --- | --- |
| Egypt - HRP (Cairo) | Sahar Abdelmaksoud (Ain-Shams University)[342] | 2020-09-06 - 2020-10-31 | Local | High | 100 | Adults (18-64 years) | All | Probability | Blood donors | 38% | AMEDA Labordiagnostik GmbH - IgG, IgM - AMP Rapid Test SARS-CoV-2 IgG/IgM | 1, 0.975 | Validated by manufacturer |
| Egypt - HRP (Cairo) | Samia Girgis (Ain-Shams University)[343] | 2020-05-05 - 2020-10-31 | Local | High | 2,927 | Multiple groups | All | Sequential | Residual sera | 30% | Roche Diagnostics - IgG, IgM, IgA - Elecsys  Anti‐SARS‐CoV‐2 (N) | 1, 0.995 | Validated by manufacturer |
| Iran (Islamic Republic of) - HRP (Zahedan) | Zahra Mohammadi (Tehran University of Medical Sciences)[344] | 2020-07-01 - 2020-07-31 | Local | High | 134 | Adults (18-64 years) | Male | Probability | Household and community samples | 4.8% | Pishtaz Diagnostics Iran - IgG, IgM - ELISA kit Pishtaz Teb Diagnostics | 0.941, 0.983 | Validated by manufacturer |
| Iran (Islamic Republic of) - HRP (Ardabil) | Hamed Zandian (Ardabil University of Medical Sciences)[345] | 2020-05-20 - 2020-06-07 | Local | Moderate | 1,013 | Multiple groups | All | Probability | Household and community samples | 12.1% | Pishtaz Diagnostics Iran - IgG, IgM - ELISA kit Pishtaz Teb Diagnostics | 0.941, 0.983 | Validated by manufacturer |
| Iraq - HRP (Duhok) | Nawfal Hussein (University of Duhok)[346] | 2021-01-10 - 2021-01-30 | Local | Moderate | 743 | Multiple groups | All | Probability | Household and community samples | 62.6% | Roche Diagnostics - IgG, IgM, IgA - Elecsys  Anti‐SARS‐CoV‐2 (N) | 1, 0.995 | Validated by manufacturer |
| Jordan - HRP | Maher Sughayer (King Hussein Cancer Center)[347] | 2021-06-01 - 2021-06-30 | National | High | 536 | Adults (18-64 years) | All | Sequential | Blood donors | 74.4% | Roche Diagnostics - IgG, IgM, IgA - Elecsys  Anti‐SARS‐CoV‐2 (S) | 0.988, 1 | Validated by manufacturer |
| Jordan - HRP | Maher Sughayer (King Hussein Cancer Center)[348] | 2021-01-28 - 2021-02-05 | National | High | 292 | Adults (18-64 years) | All | Sequential | Blood donors | 27.4% | Roche Diagnostics - IgG, IgM, IgA - Elecsys  Anti‐SARS‐CoV‐2 (N) | 1, 0.995 | Validated by manufacturer |
| Jordan - HRP | Maher Sughayer (King Hussein Cancer Center)[348] | 2020-09-01 - 2020-09-15 | National | High | 348 | Adults (18-64 years) | All | Sequential | Blood donors | 0% | Roche Diagnostics - IgG, IgM, IgA - Elecsys  Anti‐SARS‐CoV‐2 (N) | 1, 0.995 | Validated by manufacturer |
| Jordan - HRP | Maher Sughayer (King Hussein Cancer Center)[348] | 2020-01-15 - 2020-06-15 | National | High | 736 | Adults (18-64 years) | All | Sequential | Blood donors | 0% | Roche Diagnostics - IgG, IgM, IgA - Elecsys  Anti‐SARS‐CoV‐2 (N) | 1, 0.995 | Validated by manufacturer |
| occupied Palestinian territory, including east Jerusalem - HRP (West Bank) | Nouar Qutob (Arab American University )[349] | 2020-06-15 - 2020-06-30 | Local | Low | 1,355 | Multiple groups | All | Probability | Household and community samples | 0% | Roche Diagnostics - IgG, IgM, IgA - Elecsys  Anti‐SARS‐CoV‐2 (N) | 1, 0.995 | Validated by manufacturer |
| occupied Palestinian territory, including east Jerusalem - HRP (West Bank) | Nouar Qutob (Arab American University )[349] | 2020-05-01 - 2020-07-09 | Local | High | 1,136 | Multiple groups | All | Convenience | Residual sera | 0.3% | Roche Diagnostics - IgG, IgM, IgA - Elecsys  Anti‐SARS‐CoV‐2 (N) | 1, 0.995 | Validated by manufacturer |
| Pakistan - HRP (District East) | Muhammed Imran Nisar (Aga Khan University)[350] | 2020-08-17 - 2020-08-22 | Local | Moderate | 500 | Multiple groups | All | Probability | Household and community samples | 24% | Roche Diagnostics - IgG, IgM, IgA - Elecsys  Anti‐SARS‐CoV‐2 (N) | 1, 0.995 | Validated by manufacturer |
| Pakistan - HRP (District Malir) | Muhammed Imran Nisar (Aga Khan University)[350] | 2020-08-17 - 2020-08-22 | Local | Moderate | 501 | Multiple groups | All | Probability | Household and community samples | 16% | Roche Diagnostics - IgG, IgM, IgA - Elecsys  Anti‐SARS‐CoV‐2 (N) | 1, 0.995 | Validated by manufacturer |
| Pakistan - HRP | Sheeba Ansari (Liaquat University)[351] | 2020-06-15 - 2020-08-15 | Local | High | 728 | Multiple groups | All | Convenience | Residual sera | 10.8% | NA - - Not reported/ Unable to specify | 1, 0.998 | Validated by manufacturers |
| Pakistan - HRP (District East) | Muhammed Imran Nisar (Aga Khan University)[350] | 2020-06-25 - 2020-07-11 | Local | Moderate | 500 | Multiple groups | All | Probability | Household and community samples | 20% | Roche Diagnostics - IgG, IgM, IgA - Elecsys  Anti‐SARS‐CoV‐2 (N) | 1, 0.995 | Validated by manufacturer |
| Pakistan - HRP (District Malir) | Muhammed Imran Nisar (Aga Khan University)[350] | 2020-06-25 - 2020-07-11 | Local | Moderate | 504 | Multiple groups | All | Probability | Household and community samples | 12.7% | Roche Diagnostics - IgG, IgM, IgA - Elecsys  Anti‐SARS‐CoV‐2 (N) | 1, 0.995 | Validated by manufacturer |
| Pakistan - HRP | Wajiha Javed (Getz Pharma)[352] | 2020-06-22 - 2020-07-06 | Subnational | High | 24,210 | Adults (18-64 years) | All | Probability | Household and community samples | 17.5% | Getz Pharma - IgG, IgM - IgG/IgM Test Kit (Colloidal gold) | 0.953, 0.987 | Validated by manufacturer |
| Pakistan - HRP (District East) | Muhammed Imran Nisar (Aga Khan University)[350] | 2020-04-15 - 2020-04-25 | Local | Moderate | 500 | Multiple groups | All | Probability | Household and community samples | 0.4% | Roche Diagnostics - IgG, IgM, IgA - Elecsys  Anti‐SARS‐CoV‐2 (N) | 1, 0.995 | Validated by manufacturer |
| Pakistan - HRP (District Malir) | Muhammed Imran Nisar (Aga Khan University)[350] | 2020-04-15 - 2020-04-25 | Local | Moderate | 500 | Multiple groups | All | Probability | Household and community samples | 0% | Roche Diagnostics - IgG, IgM, IgA - Elecsys  Anti‐SARS‐CoV‐2 (N) | 1, 0.995 | Validated by manufacturer |
| Saudi Arabia (Riyadh) | Mohammed Alosaimi (King Saud University)[353] | 2020-11-22 - 2020-12-17 | Local | High | 515 | Multiple groups | All | Convenience | Blood donors | 12.2% | Abbott Laboratories - IgG - Abbott Architect SARS-CoV-2 IgG | 0.99, 1 | Validated by manufacturer |
| Saudi Arabia | Sami Almudarra (Saudi Ministry of Health)[354] | 2020-08-01 - 2020-08-31 | Local | Moderate | 5,629 | Multiple groups | All | Probability | Household and community samples | 50.2% | Roche Diagnostics - IgG, IgM, IgA - Elecsys  Anti‐SARS‐CoV‐2 (N) | 1, 0.995 | Validated by manufacturer |
| United Arab Emirates (Dubai) | May Raouf (Dubai Health Authority)[355] | 2020-08-15 - 2020-12-15 | Local | Moderate | 1,418 | Adults (18-64 years) | All | Probability | Blood donors | 13.5% | Abbott Laboratories - IgG - Abbott Architect SARS-CoV-2 IgG | 0.99, 1 | Validated by manufacturer |
| Yemen - HRP (Aden) | Abdulla Bin-Ghouth (World Health Organization Country Office, Yemen)[356] | 2020-11-15 - 2020-12-15 | Local | Moderate | 2,001 | Multiple groups | All | Probability | Household and community samples | 27.4% | Healgen - IgG, IgM - COVID-19 IgG/IgM Rapid Test Cassette,Beijing Wantai Biological - IgG, IgM, IgA - Wantai SARS-CoV-2 Total Ab ELISA | 0.907, 1 | Complex test algorithm: Manual calculation |

#### Europe region

| Country (Location) | Author (Organization) | Sampling Dates (YMD) | Geographic scope | Overall risk of bias | Sample size | Age | Sex | Sampling method | Sample frame | Seroprevalence (95% CI) | Test Manufacturers - Isotypes - Names | Test Sens, Spec | Sens, Spec Source |
| --- | --- | --- | --- | --- | --- | --- | --- | --- | --- | --- | --- | --- | --- |
| Albania (Tirana) | Genc Sulcebe (University of Medicine and University Hospital Center of Tirana)[357] | 2020-12-21 - 2020-12-28 | Local | Moderate | 815 | Adults (18-64 years) | All | Probability | Household and community samples | 48.2% | EUROIMMUN - IgG - Anti-SARS-CoV-2 ELISA IgG | 0.9, 1 | Validated by manufacturer |
| Albania (Tirana) | Genc Sulcebe (University of Medicine and University Hospital Center of Tirana)[357] | 2020-06-27 - 2020-07-03 | Local | High | 266 | Adults (18-64 years) | All | Probability | Household and community samples | 7.5% | EUROIMMUN - IgG - Anti-SARS-CoV-2 ELISA IgG | 0.9, 1 | Validated by manufacturer |
| Bosnia and Herzegovina (Sarajevo) | Adna Asic (International Burch University)[358] | 2020-04-21 - 2020-07-17 | Local | High | 1,121 | Multiple groups | All | Convenience | Residual sera | 4.3% | Dynamiker Biotechnology (Tianjin) Co. Ltd - IgG, IgM - 2019-nCoV IgG/IgM Rapid Test | 0.957, 0.991 | Validated by manufacturer |
| Bosnia and Herzegovina (Sarajevo) | Adna Asic (International Burch University)[358] | 2020-04-15 - 2020-05-15 | Local | High | 1,720 | Multiple groups | All | Convenience | Residual sera | 3.4% | Artron Laboratories Inc. - IgG, IgM - One Step Novel Coronavirus (COVID-19) IgM/IgG Antibody Test | 0.972, 0.978 | Validated by manufacturer |
| Bulgaria (Varna) | Denitsa Tsaneva-Damyanova (Medical Diagnostic Laboratory “STATUS” )[359] | 2020-03-26 - 2020-04-20 | Local | High | 586 | Multiple groups | All | Convenience | Residual sera | 4.8% | Zhejiang Orient Gene Biotech - IgG, IgM - COVID-19 IgG/IgM Rapid Test | 0.972, 1 | Validated by manufacturer |
| Kazakhstan | Yuliya Semenova (Nazarbayev University School of Medicine)[360] | 2020-07-16 - 2021-07-07 | National | High | 85,346 | Multiple groups | All | Sequential | Residual sera | 63% | Vector BEST - IgG, IgM - SARS- CoV-2-IgG-EIA-BEST and SARS-CoV-2- IgM-EIA-BEST | 0.9, 1 | Validated by independent authors/third party/non-developers |
| Kazakhstan (Almaty) | Manar Smagul (Kazakh National Medical University)[361] | 2020-10-24 - 2021-01-11 | Local | Low | 4,461 | Multiple groups | All | Probability | Household and community samples | 57% | Beijing Wantai Biological - IgG, IgM, IgA - Wantai SARS-CoV-2 Total Ab ELISA | 0.967, 0.975 | Validated by manufacturer |
| Kazakhstan (Kostanay) | Manar Smagul (Kazakh National Medical University)[361] | 2020-10-24 - 2021-01-11 | Local | Low | 614 | Multiple groups | All | Probability | Household and community samples | 39.4% | Beijing Wantai Biological - IgG, IgM, IgA - Wantai SARS-CoV-2 Total Ab ELISA | 0.967, 0.975 | Validated by manufacturer |
| Kazakhstan (Oskemen) | Manar Smagul (Kazakh National Medical University)[361] | 2020-10-24 - 2021-01-11 | Local | Low | 664 | Multiple groups | All | Probability | Household and community samples | 60.7% | Beijing Wantai Biological - IgG, IgM, IgA - Wantai SARS-CoV-2 Total Ab ELISA | 0.967, 0.975 | Validated by manufacturer |
| North Macedonia (Skopje) | Tatjana Makarovska Bojadjieva ( Institute for Transfusion Medicine)[362] | 2020-11-15 - 2021-03-15 | Local | High | 9,773 | Multiple groups | All | Sequential | Blood donors | 25% | Abbott Laboratories - IgG - Abbott Architect SARS-CoV-2 IgG | 0.99, 1 | Validated by manufacturer |
| Russian Federation (Krasnodar) | Vladimir Gorodin (Kuban State Medical University)[363] | 2020-10-26 - 2020-12-30 | Local | High | 806 | Adults (18-64 years) | All | Convenience | Household and community samples | 13.2% | Beckman Coulter - IgG - Access SARS-CoV-2 IgG Antibody Test | 1, 0.998 | Validated by manufacturer |
| Russian Federation (Saint Petersburg) | Anton Barchuk (Smorodinova Research Institute of Influenza)[364] | 2020-10-12 - 2020-12-06 | Local | Low | 1,322 | Multiple groups | All | Probability | Household and community samples | 21% | Genetico - IgG, IgM, IgA - CoronaPass total antibodies test | 0.911, 0.9 | Validated by manufacturer |
| Russian Federation (Saint Petersburg) | Anton Barchuk (Smorodinova Research Institute of Influenza)[364] | 2020-07-20 - 2020-08-08 | Local | Moderate | 474 | Multiple groups | All | Probability | Household and community samples | 12.2% | Genetico - IgG, IgM, IgA - CoronaPass total antibodies test | 0.911, 0.9 | Validated by manufacturer |
| Russian Federation (Saint Petersburg) | Anton Barchuk (European University at St. Petersburg )[365] | 2020-05-27 - 2020-06-26 | Local | Low | 990 | Multiple groups | All | Probability | Household and community samples | 7.4% | Abbott Laboratories - IgG - Abbott Architect SARS-CoV-2 IgG | 0.99, 1 | Validated by manufacturer |
| Turkey - HRP (Ankara) | Ozge Yucel Celik (Etlik Zübeyde Hanim Women’s Health Care, Training, and Research Hospital)[366] | 2020-06-01 - 2020-07-31 | Local | High | 230 | Adults (18-64 years) | All | Sequential | Pregnant or parturient women | 0% | Assure Tech. (Hangzhou) Co. Ltd - IgG, IgM - COVID-19 IgG/IgM Rapid Test Device | 1, 0.988 | Validated by manufacturer |
| Turkey - HRP (Ankara) | Ozge Yucel Celik (Etlik Zübeyde Hanim Women’s Health Care, Training, and Research Hospital)[366] | 2020-06-01 - 2020-07-31 | Local | High | 205 | Adults (18-64 years) | All | Sequential | Pregnant or parturient women | 2.9% | Assure Tech. (Hangzhou) Co. Ltd - IgG, IgM - COVID-19 IgG/IgM Rapid Test Device | 1, 0.988 | Validated by manufacturer |
| Austria (Tyrol) | Anita Siller (Medical University of Innsbruck )[367] | 2021-03-19 - 2021-09-30 | Subnational | High | 19,792 | Multiple groups | All | Sequential | Blood donors | 63.2% | Abbott Laboratories - IgG - SARS-CoV-2 IgG II Quant | 0.994, 0.996 | Validated by manufacturer |
| Austria (Vienna) | M. Riesenhuber (Universitätsklinik für Innere Medizin II )[368] | 2021-02-08 - 2021-02-22 | Local | High | 3,082 | Multiple groups | All | Sequential | Residual sera | 9.3% | Roche Diagnostics - IgG, IgM, IgA - Elecsys  Anti‐SARS‐CoV‐2 (N) | 1, 0.995 | Validated by manufacturer |
| Austria (Weißenkirchen in der Wachau) | Heike Rebholz (Danube Private University)[369] | 2021-02-13 - 2021-02-13 | Local | High | 552 | Multiple groups | All | Convenience | Household and community samples | 25% | EUROIMMUN - IgG - Anti-SARS-CoV-2 ELISA IgG | 0.9, 1 | Validated by manufacturer |
| Austria | Lisa Weidner ( University Hospital of Salzburg)[370] | 2020-11-05 - 2020-12-04 | National | High | 3,311 | Multiple groups | All | Convenience | Blood donors | 5.4% | Roche Diagnostics - IgG, IgM, IgA - Elecsys  Anti‐SARS‐CoV‐2 (N) | 1, 0.995 | Validated by manufacturer |
| Austria (Ischgl) | Wegene Borena (Medical University Of Innsbruck)[371] | 2020-11-02 - 2020-11-08 | Local | Moderate | 801 | Adults (18-64 years) | All | Convenience | Household and community samples | 45.4% | EUROIMMUN - IgA - Anti-SARS-CoV-2 ELISA IgA,EUROIMMUN - IgG - Anti-SARS-CoV-2 ELISA IgG,Abbott Laboratories - IgG - Abbott Architect SARS-CoV-2 IgG,Roche Diagnostics - IgG, IgM, IgA - Elecsys  Anti‐SARS‐CoV‐2 (N),NA - - Author designed (Neutralization Assay) | N/A | Complex test algorithm: Manual review |
| Austria (Weißenkirchen) | Dennis Ladage (Danube Private University)[372] | 2020-06-01 - 2020-06-30 | Local | Moderate | 835 | Multiple groups | All | Convenience | Household and community samples | 8.5% | EUROIMMUN - IgG - Anti-SARS-CoV-2 ELISA IgG | 0.9, 1 | Validated by manufacturer |
| Austria (Ischgl) | Jens Lehmann ( Medical University of Innsbruck)[373] | 2020-04-21 - 2020-04-27 | Local | High | 451 | Multiple groups | All | Probability | Household and community samples | 43.7% | Abbott Laboratories - IgG - Abbott Architect SARS-CoV-2 IgG,EUROIMMUN - IgA - Anti-SARS-CoV-2 ELISA IgA,EUROIMMUN - IgG - Anti-SARS-CoV-2 ELISA IgG,NA - - Author designed (Neutralization Assay) | N/A | Complex test algorithm: Manual review |
| Belgium (Ghent) | Laura Heireman (Ghent University Hospital)[374] | 2020-01-15 - 2020-11-15 | Local | High | 265 | Adults (18-64 years) | Female | Convenience | Pregnant or parturient women | 1.5% | EUROIMMUN - IgG - Anti-SARS-CoV-2 ELISA IgG,Epitope Diagnostics, Inc. - IgG - EDI™ Novel Coronavirus COVID-19 IgG ELISA Kit | 0.998, 0.998 | Complex test algorithm: Manual calculation |
| Belgium | Peter van Dam (Antwerp University Hospital)[375] | 2020-03-24 - 2020-05-31 | Local | High | 90 | Multiple groups | All | Sequential | Residual sera | 5.6% | DiaSorin - IgG - Liaison SARS-CoV-2 S1/S2 IgG | 0.974, 0.985 | Validated by manufacturer |
| Croatia | Tatjana Vilibic-Cavlek (Croatian Institute of Public Health)[376] | 2020-12-15 - 2021-02-15 | National | High | 1,436 | Multiple groups | All | Convenience | Multiple general populations | 25.1% | Vircell S.L. - IgG - COVID-19 ELISA IgG,NA - - Author designed (Neutralization Assay) | N/A | Complex test algorithm: Manual review |
| Croatia | Tatjana Vilibic-Cavlek (Croatian Institute of Public Health)[376] | 2020-05-15 - 2020-07-15 | National | High | 1,088 | Multiple groups | All | Convenience | Multiple general populations | 2.2% | Vircell S.L. - IgG - COVID-19 ELISA IgG,NA - - Author designed (Neutralization Assay) | N/A | Complex test algorithm: Manual review |
| Croatia (Zagreb) | Jasna Lenicek Krleza (Children’s Hospital Zagreb)[377] | 2020-05-13 - 2020-05-29 | Local | High | 240 | Children and Youth (0-17 years) | All | Convenience | Residual sera | 2.9% | Vircell S.L. - IgG - COVID-19 ELISA IgG,Vircell S.L. - IgM, IgA - COVID-19 ELISA IgM/IgA,NA - - Author designed (Neutralization Assay) | N/A | Complex test algorithm: Manual review |
| Czechia | Pavel Piler (Masaryk University)[378] | 2021-02-01 - 2021-03-31 | National | High | 19,548 | Multiple groups | All | Convenience | Household and community samples | 51.4% | DiaSorin - IgG - Liaison SARS-CoV-2 S1/S2 IgG,Abbott Laboratories - IgG - SARS-CoV-2 IgG II Quant | 1, 0.981 | Complex test algorithm: Manual calculation |
| Czechia | Pavel Piler (Masaryk University)[378] | 2020-12-01 - 2021-01-31 | National | High | 6,880 | Multiple groups | All | Convenience | Household and community samples | 43.4% | DiaSorin - IgG - Liaison SARS-CoV-2 S1/S2 IgG,Abbott Laboratories - IgG - SARS-CoV-2 IgG II Quant | 1, 0.981 | Complex test algorithm: Manual calculation |
| Czechia | Pavel Piler (Masaryk University)[378] | 2020-10-01 - 2020-11-30 | National | High | 3,626 | Multiple groups | All | Convenience | Household and community samples | 28.3% | DiaSorin - IgG - Liaison SARS-CoV-2 S1/S2 IgG,Abbott Laboratories - IgG - SARS-CoV-2 IgG II Quant | 1, 0.981 | Complex test algorithm: Manual calculation |
| Czechia (Prague) | Marketa Bloomfield (Charles University and Thomayer’s Hospital)[379] | 2020-07-03 - 2020-08-19 | Local | High | 200 | Children and Youth (0-17 years) | All | Sequential | Residual sera | 0% | Roche Diagnostics - IgG, IgM, IgA - Elecsys  Anti‐SARS‐CoV‐2 (N) | 1, 0.995 | Validated by manufacturer |
| Denmark (Capital Region and Region Zealand) | Maren J H Rytter (Slagelse Sygehus)[174] | 2020-06-22 - 2020-07-03 | Subnational | High | 1,033 | Children and Youth (0-17 years) | All | Convenience | Residual sera | 1.6% | Zhuhai Livzon Diagnostics Inc - IgG, IgM - 2019-nCoV IgG/IgM Antibody Detection Kit | 0.906, 0.992 | Validated by manufacturer |
| Denmark (Eastern Central Denmark Region) | Senne Jespersen (Aarhus University Hospital)[380] | 2020-06-17 - 2020-06-30 | Subnational | High | 180 | Adults (18-64 years) | All | Convenience | Blood donors | 0.6% | Beijing Wantai Biological - IgG, IgM, IgA - Wantai Rapid Test for Total Antibody to SARS-CoV-2 | 0.947, 0.989 | Validated by manufacturer |
| Denmark (Western Central Denmark Region) | Senne Jespersen (Aarhus University Hospital)[380] | 2020-06-17 - 2020-06-30 | Subnational | High | 180 | Adults (18-64 years) | All | Convenience | Blood donors | 1.2% | Beijing Wantai Biological - IgG, IgM, IgA - Wantai Rapid Test for Total Antibody to SARS-CoV-2 | 0.947, 0.989 | Validated by manufacturer |
| Denmark | Ole Birger Pedersen (Copenhagen University Hospital Rigshospitalet)[64] | 2020-06-01 - 2020-06-26 | National | High | 1,110 | Adults (18-64 years) | All | Sequential | Blood donors | 3% | Beijing Wantai Biological - IgG, IgM, IgA - Wantai SARS-CoV-2 Total Ab ELISA | 0.967, 0.975 | Validated by manufacturer |
| Denmark (Copenhagen) | Pia Egerup (Copenhagen University Hospital Hvidovre)[381] | 2020-04-04 - 2020-07-03 | Local | Moderate | 1,313 | Adults (18-64 years) | Female | Sequential | Pregnant or parturient women | 2.1% | Shenzhen Yhlo Biotech Co. Ltd - IgG, IgM - iFlash-SARS-CoV-2 IgM/IgG | 0.94, 0.993 | Validated by independent authors/third party/non-developers |
| Estonia | Hiie Soeorg (University of Tartu)[382] | 2021-02-08 - 2021-03-25 | National | High | 2,517 | Multiple groups | All | Stratified non-probability | Residual sera | 20.1% | Abbott Laboratories - IgG - SARS-CoV-2 IgG II Quant | 0.994, 0.996 | Validated by manufacturer |
| Estonia | Piret Veerus (University of Tartu)[383] | 2020-05-04 - 2020-06-10 | National | High | 433 | Adults (18-64 years) | Female | Convenience | Pregnant or parturient women | 0.5% | Abbott Laboratories - IgG - Abbott Architect SARS-CoV-2 IgG | 0.99, 1 | Validated by manufacturer |
| Finland | Finnish institute for health and welfare (Finnish Institute for Health and Welfare)[66] | 2020-09-20 - 2020-09-26 | National | High | 56 | Adults (18-64 years) | All | Probability | Household and community samples | 1.8% | NA - - Author designed (Neutralization Assay),NA - - Author designed (Multiplex) | N/A | Complex test algorithm: Manual review |
| Finland | Finnish institute for health and welfare (Finnish Institute for Health and Welfare)[66] | 2020-09-13 - 2020-09-19 | National | High | 48 | Adults (18-64 years) | All | Probability | Household and community samples | 2.1% | NA - - Author designed (Neutralization Assay),NA - - Author designed (Multiplex) | N/A | Complex test algorithm: Manual review |
| Finland | Finnish institute for health and welfare (Finnish Institute for Health and Welfare)[66] | 2020-09-06 - 2020-09-12 | National | High | 54 | Adults (18-64 years) | All | Probability | Household and community samples | 0% | NA - - Author designed (Neutralization Assay),NA - - Author designed (Multiplex) | N/A | Complex test algorithm: Manual review |
| Finland | Finnish institute for health and welfare (Finnish Institute for Health and Welfare)[66] | 2020-08-30 - 2020-09-05 | National | High | 63 | Adults (18-64 years) | All | Probability | Household and community samples | 0% | NA - - Author designed (Neutralization Assay),NA - - Author designed (Multiplex) | N/A | Complex test algorithm: Manual review |
| Finland | Finnish institute for health and welfare (Finnish Institute for Health and Welfare)[66] | 2020-08-23 - 2020-08-29 | National | High | 48 | Adults (18-64 years) | All | Probability | Household and community samples | 2.1% | NA - - Author designed (Neutralization Assay),NA - - Author designed (Multiplex) | N/A | Complex test algorithm: Manual review |
| Finland | Finnish institute for health and welfare (Finnish Institute for Health and Welfare)[66] | 2020-08-16 - 2020-08-22 | National | High | 63 | Adults (18-64 years) | All | Probability | Household and community samples | 0% | NA - - Author designed (Neutralization Assay),NA - - Author designed (Multiplex) | N/A | Complex test algorithm: Manual review |
| Finland | Finnish institute for health and welfare (Finnish Institute for Health and Welfare)[66] | 2020-08-09 - 2020-08-15 | National | High | 64 | Adults (18-64 years) | All | Probability | Household and community samples | 0% | NA - - Author designed (Neutralization Assay),NA - - Author designed (Multiplex) | N/A | Complex test algorithm: Manual review |
| Finland | Finnish institute for health and welfare (Finnish Institute for Health and Welfare)[66] | 2020-08-02 - 2020-08-08 | National | High | 49 | Adults (18-64 years) | All | Probability | Household and community samples | 2% | NA - - Author designed (Neutralization Assay),NA - - Author designed (Multiplex) | N/A | Complex test algorithm: Manual review |
| Finland | Finnish institute for health and welfare (Finnish Institute for Health and Welfare)[66] | 2020-07-26 - 2020-08-01 | National | High | 75 | Adults (18-64 years) | All | Probability | Household and community samples | 1.3% | NA - - Author designed (Neutralization Assay),NA - - Author designed (Multiplex) | N/A | Complex test algorithm: Manual review |
| Finland | Finnish institute for health and welfare (Finnish Institute for Health and Welfare)[66] | 2020-07-19 - 2020-07-25 | National | High | 9 | Adults (18-64 years) | All | Probability | Household and community samples | 11.1% | NA - - Author designed (Neutralization Assay),NA - - Author designed (Multiplex) | N/A | Complex test algorithm: Manual review |
| Finland | Finnish institute for health and welfare (Finnish Institute for Health and Welfare)[66] | 2020-07-12 - 2020-07-18 | National | High | 7 | Adults (18-64 years) | All | Probability | Household and community samples | 0% | NA - - Author designed (Neutralization Assay),NA - - Author designed (Multiplex) | N/A | Complex test algorithm: Manual review |
| Finland | Finnish institute for health and welfare (Finnish Institute for Health and Welfare)[66] | 2020-07-05 - 2020-07-11 | National | High | 51 | Adults (18-64 years) | All | Probability | Household and community samples | 0% | NA - - Author designed (Neutralization Assay),NA - - Author designed (Multiplex) | N/A | Complex test algorithm: Manual review |
| Finland | Finnish institute for health and welfare (Finnish Institute for Health and Welfare)[66] | 2020-06-28 - 2020-07-04 | National | High | 85 | Adults (18-64 years) | All | Probability | Household and community samples | 0% | NA - - Author designed (Neutralization Assay),NA - - Author designed (Multiplex) | N/A | Complex test algorithm: Manual review |
| Finland | Finnish institute for health and welfare (Finnish Institute for Health and Welfare)[66] | 2020-06-21 - 2020-06-27 | National | High | 32 | Adults (18-64 years) | All | Probability | Household and community samples | 0% | NA - - Author designed (Neutralization Assay),NA - - Author designed (Multiplex) | N/A | Complex test algorithm: Manual review |
| Finland | Finnish institute for health and welfare (Finnish Institute for Health and Welfare)[66] | 2020-06-14 - 2020-06-20 | National | High | 78 | Adults (18-64 years) | All | Probability | Household and community samples | 0% | NA - - Author designed (Neutralization Assay),NA - - Author designed (Multiplex) | N/A | Complex test algorithm: Manual review |
| Finland | Finnish institute for health and welfare (Finnish Institute for Health and Welfare)[66] | 2020-06-07 - 2020-06-13 | National | High | 174 | Adults (18-64 years) | All | Probability | Household and community samples | 0% | NA - - Author designed (Neutralization Assay),NA - - Author designed (Multiplex) | N/A | Complex test algorithm: Manual review |
| Finland | Finnish institute for health and welfare (Finnish Institute for Health and Welfare)[66] | 2020-05-31 - 2020-06-06 | National | High | 214 | Adults (18-64 years) | All | Probability | Household and community samples | 0.5% | NA - - Author designed (Neutralization Assay),NA - - Author designed (Multiplex) | N/A | Complex test algorithm: Manual review |
| Finland | Finnish institute for health and welfare (Finnish Institute for Health and Welfare)[66] | 2020-05-24 - 2020-05-30 | National | High | 178 | Adults (18-64 years) | All | Probability | Household and community samples | 0% | NA - - Author designed (Neutralization Assay),NA - IgG, IgM - Author designed (IFA) - MULTIPLEXED | N/A | Complex test algorithm: Manual review |
| Finland | Finnish institute for health and welfare (Finnish Institute for Health and Welfare)[66] | 2020-05-17 - 2020-05-23 | National | High | 210 | Adults (18-64 years) | All | Probability | Household and community samples | 0.5% | NA - - Author designed (Neutralization Assay),NA - IgG, IgM - Author designed (IFA) - MULTIPLEXED | N/A | Complex test algorithm: Manual review |
| Finland | Finnish institute for health and welfare (Finnish Institute for Health and Welfare)[66] | 2020-05-10 - 2020-05-16 | National | High | 401 | Adults (18-64 years) | All | Probability | Household and community samples | 0.2% | NA - - Author designed (Neutralization Assay),NA - IgG, IgM - Author designed (IFA) - MULTIPLEXED | N/A | Complex test algorithm: Manual review |
| Finland | Finnish institute for health and welfare (Finnish Institute for Health and Welfare)[66] | 2020-05-03 - 2020-05-09 | National | High | 514 | Adults (18-64 years) | All | Probability | Household and community samples | 0% | NA - - Author designed (Neutralization Assay),NA - IgG, IgM - Author designed (IFA) - MULTIPLEXED | N/A | Complex test algorithm: Manual review |
| Finland | Finnish institute for health and welfare (Finnish Institute for Health and Welfare)[66] | 2020-04-26 - 2020-05-02 | National | High | 426 | Adults (18-64 years) | All | Probability | Household and community samples | 0.5% | NA - - Author designed (Neutralization Assay),NA - IgG, IgM - Author designed (IFA) - MULTIPLEXED | N/A | Complex test algorithm: Manual review |
| Finland | Finnish institute for health and welfare (Finnish Institute for Health and Welfare)[66] | 2020-04-12 - 2020-04-18 | National | High | 362 | Adults (18-64 years) | All | Probability | Household and community samples | 0.3% | NA - - Author designed (Neutralization Assay),NA - IgG, IgM - Author designed (IFA) - MULTIPLEXED | N/A | Complex test algorithm: Manual review |
| France (Paris) | Anael Ayrolles (Paris University)[384] | 2020-12-15 - 2021-04-15 | Local | High | 52 | Children and Youth (0-17 years) | All | Convenience | Residual sera | 34.6% | Abbott Laboratories - IgG - AdviseDX SARS-CoV-2 anti-IgG II assay | 0.981, 0.996 | Validated by manufacturer |
| France | Fabrice Carrat (Unité des Virus Émergents)[385] | 2020-09-01 - 2020-09-30 | National | High | 5,770 | Multiple groups | All | Convenience | Household and community samples | 3.9% | EUROIMMUN - IgG - Anti-SARS-CoV-2 ELISA IgG | 0.9, 1 | Validated by manufacturer |
| France (Nancy) | Anne Petit (Université de Lorraine)[386] | 2020-06-26 - 2020-07-24 | Local | Moderate | 2,006 | Multiple groups | All | Convenience | Household and community samples | 2.1% | Bio-rad - IgG, IgM, IgA - Platelia SARS-CoV-2 Total Ab assay,Biosynex - IgG, IgM - Biosynex COVID-19 BSS assay,EUROIMMUN - IgA - Anti-SARS-CoV-2 ELISA IgA | N/A | Complex test algorithm: Manual review |
| France (Perpignan) | Adeline Beaumont (Perpignan Hospital Center)[387] | 2020-06-29 - 2020-07-17 | Local | Moderate | 700 | Multiple groups | All | Probability | Household and community samples | 35.4% | Roche Diagnostics - IgG, IgM, IgA - Elecsys  Anti‐SARS‐CoV‐2 (S) | 0.988, 1 | Validated by manufacturer |
| France | Tom Woudenberg (Institut Pasteur)[388] | 2020-05-01 - 2020-08-15 | National | High | 1,306 | Multiple groups | All | Convenience | Residual sera | 8.5% | NA - - Author designed (Luminex) | 0.98, 0.99 | Validated by developers |
| France (Paris) | Vassilis Tsatsarisa (Université Paris-Saclay)[389] | 2020-04-29 - 2020-06-26 | Local | High | 529 | Adults (18-64 years) | Female | Sequential | Pregnant or parturient women | 4.7% | Abbott Laboratories - IgG - Abbott Architect SARS-CoV-2 IgG | 0.99, 1 | Validated by manufacturer |
| France (Paris) | Jeremie Mattern (Paris Saclay University)[390] | 2020-05-04 - 2020-05-31 | Local | High | 249 | Adults (18-64 years) | Female | Sequential | Pregnant or parturient women | 8% | Abbott Laboratories - IgG - Abbott Architect SARS-CoV-2 IgG | 0.99, 1 | Validated by manufacturer |
| France (Corsica) | Lisandru Capai (Université de Corse)[391] | 2020-04-16 - 2020-06-15 | Subnational | High | 1,973 | Multiple groups | All | Sequential | Residual sera | 5.5% | EUROIMMUN - IgG - Anti-SARS-CoV-2 ELISA IgG | 0.9, 1 | Validated by manufacturer |
| France (Paris) | Robert Cohen (Centre Hospitalier Intercommunal de Créteil)[392] | 2020-04-14 - 2020-05-12 | Local | High | 605 | Children and Youth (0-17 years) | All | Sequential | Residual sera | 10.7% | Biosynex - IgG, IgM - Biosynex COVID-19 BSS assay | 0.975, 1 | Validated by manufacturer |
| Germany (Erlangen) | Felix Wachter (Friedrich Alexander University)[393] | 2021-06-15 - 2021-07-15 | Local | High | 946 | Children and Youth (0-17 years) | All | Sequential | Residual sera | 14.6% | Roche Diagnostics - IgG, IgM, IgA - Elecsys  Anti‐SARS‐CoV‐2 (S) | 0.988, 1 | Validated by manufacturer |
| Germany | Bastian Fischer (Institut fur Laboratoriums- und Transfusionsmedizin)[394] | 2021-06-01 - 2021-06-30 | National | High | 996 | Adults (18-64 years) | All | Convenience | Blood donors | 2.3% | EUROIMMUN - IgG - Anti-SARS-CoV-2 ELISA IgG,EUROIMMUN - IgG - Anti-SARS-CoV-2 NCP ELISA (IgG) | 0.851, 1 | Complex test algorithm: Manual calculation |
| Germany (Gangelt) | Enrico Richter (German Center for Infection Research)[395] | 2021-04-01 - 2021-04-30 | Local | Moderate | 406 | Multiple groups | All | Probability | Household and community samples | 35.9% | EUROIMMUN - IgG - Anti-SARS-CoV-2 ELISA IgG | 0.9, 1 | Validated by manufacturer |
| Germany (Erlangen) | Felix Wachter (Friedrich Alexander University)[393] | 2021-04-01 - 2021-04-30 | Local | High | 808 | Children and Youth (0-17 years) | All | Sequential | Residual sera | 9.2% | Roche Diagnostics - IgG, IgM, IgA - Elecsys  Anti‐SARS‐CoV‐2 (S) | 0.988, 1 | Validated by manufacturer |
| Germany (Gangelt) | Enrico Richter (German Center for Infection Research)[395] | 2021-01-01 - 2021-01-31 | Local | Moderate | 488 | Multiple groups | All | Probability | Household and community samples | 21% | EUROIMMUN - IgG - Anti-SARS-CoV-2 ELISA IgG | 0.9, 1 | Validated by manufacturer |
| Germany (Munich) | Katja Radon (German Research Center for Environmental Health)[396] | 2020-11-02 - 2021-01-31 | Local | Low | 4,433 | Multiple groups | All | Probability | Household and community samples | 3.1% | Roche Diagnostics - IgG, IgM, IgA - Elecsys  Anti‐SARS‐CoV‐2 (N) | 1, 0.995 | Validated by manufacturer |
| Germany (Berlin-Mitte) | Robert Koch Institut (Robert Koch Institut)[397] | 2020-11-17 - 2020-12-05 | Local | Low | 2,287 | Multiple groups | All | Probability | Household and community samples | 2.4% | EUROIMMUN - IgG - Anti-SARS-CoV-2 NCP ELISA (IgG),NA - - Author designed (Neutralization Assay) | N/A | Complex test algorithm: Manual review |
| Germany (Erlangen) | Felix Wachter (Friedrich Alexander University)[393] | 2020-10-15 - 2020-11-15 | Local | High | 872 | Children and Youth (0-17 years) | All | Sequential | Residual sera | 1.4% | Roche Diagnostics - IgG, IgM, IgA - Elecsys  Anti‐SARS‐CoV‐2 (S) | 0.988, 1 | Validated by manufacturer |
| Germany (Gangelt) | Enrico Richter (German Center for Infection Research)[395] | 2020-10-01 - 2020-10-31 | Local | Moderate | 587 | Multiple groups | All | Probability | Household and community samples | 18.1% | EUROIMMUN - IgG - Anti-SARS-CoV-2 ELISA IgG | 0.9, 1 | Validated by manufacturer |
| Germany (Ruhr) | Folke Brinkmann (Ruhr University Bochum)[398] | 2020-06-15 - 2021-02-15 | Subnational | High | 2,145 | Children and Youth (0-17 years) | All | Convenience | Residual sera | 2.6% | Roche Diagnostics - IgG, IgM, IgA - Elecsys  Anti‐SARS‐CoV‐2 (N) | 1, 0.995 | Validated by manufacturer |
| Germany (Straubing) | Robert Koch Institute (Robert Koch Institute)[399] | 2020-09-08 - 2020-09-26 | Local | Low | 2,361 | Multiple groups | All | Probability | Household and community samples | 1.8% | EUROIMMUN - IgG - Anti-SARS-CoV-2 ELISA IgG,NA - - Author designed (Neutralization Assay) | N/A | Complex test algorithm: Manual review |
| Germany (Würzburg) | Felizitas Eichner (Universität Würzburg)[400] | 2020-06-01 - 2020-10-31 | Local | Moderate | 3,034 | Multiple groups | All | Stratified non-probability | Household and community samples | 1.1% | Roche Diagnostics - IgG, IgM, IgA - Elecsys  Anti‐SARS‐CoV‐2 (N) | 1, 0.995 | Validated by manufacturer |
| Germany (Bavaria) | Markus Hippich ( German Research Center for Environmental Health)[401] | 2020-07-01 - 2020-07-31 | Subnational | High | 2,746 | Children and Youth (0-17 years) | All | Convenience | Residual sera | 0.9% | NA - - Author designed (CLIA) | 0.973, 1 | Validated by independent authors/third party/non-developers |
| Germany (Bavaria) | Ralf Wagner (Friedrich-Alexander University)[402] | 2020-06-28 - 2020-07-10 | Local | Moderate | 4,201 | Multiple groups | All | Probability | Household and community samples | 8.6% | Shenzhen Yhlo Biotech Co. Ltd - IgG, IgM - iFlash-SARS-CoV-2 IgM/IgG,Roche Diagnostics - IgG, IgM, IgA - Elecsys  Anti‐SARS‐CoV‐2 (N),NA - IgG - Author designed (ELISA) -Spike | N/A | Complex test algorithm: Manual review |
| Germany (Hamburg) | Gabor Dunay (University Medical Center Hamburg-Eppendorf)[403] | 2020-05-11 - 2020-06-30 | Local | High | 4,657 | Children and Youth (0-17 years) | All | Convenience | Household and community samples | 1.4% | Roche Diagnostics - IgG, IgM, IgA - Elecsys  Anti‐SARS‐CoV‐2 (N),DiaSorin - IgG - Liaison SARS-CoV-2 S1/S2 IgG | 0.974, 1 | Complex test algorithm: Manual calculation |
| Germany (Kupferzell) | Claudia Santos Hovener (Robert Koch Institute)[404] | 2020-05-20 - 2020-06-09 | Local | Moderate | 2,203 | Multiple groups | All | Probability | Household and community samples | 11.3% | EUROIMMUN - IgG - Anti-SARS-CoV-2 ELISA IgG | 0.9, 1 | Validated by manufacturer |
| Germany (Bonn) | N Ahmad Aziz (University of Bonn)[405] | 2020-04-24 - 2020-06-30 | Local | High | 4,755 | Multiple groups | All | Convenience | Household and community samples | 1% | EUROIMMUN - IgG - Anti-SARS-CoV-2 ELISA IgG | 0.9, 1 | Validated by manufacturer |
| Germany (Munich) | Michael Pritsch (University Hospital LMU Munich)[406] | 2020-04-05 - 2020-06-12 | Local | Low | 5,313 | Multiple groups | All | Probability | Household and community samples | 1.9% | Roche Diagnostics - IgG, IgM, IgA - Elecsys  Anti‐SARS‐CoV‐2 (N) | 1, 0.995 | Validated by manufacturer |
| Germany (Southwest) | Stefan Runkel (University Medical Center of The Johannes Gutenberg-University Mainz)[407] | 2020-03-15 - 2020-06-15 | Subnational | High | 3,880 | Multiple groups | All | Convenience | Blood donors | 0.9% | Abbott Laboratories - IgG - Abbott Architect SARS-CoV-2 IgG | 0.99, 1 | Validated by manufacturer |
| Germany (Jena) | Janine Zollkau (Universitätsklinikum Jena)[408] | 2020-04-06 - 2020-05-13 | Local | High | 180 | Multiple groups | Female | Sequential | Pregnant or parturient women | 0.6% | Epitope Diagnostics, Inc. - IgG - EDI™ Novel Coronavirus COVID-19 IgG ELISA Kit | 0.984, 0.998 | Validated by manufacturer |
| Germany (Regensberg) | Sebastian Hausler (Hospital St Hedwig of the Order of St John)[409] | 2020-04-09 - 2020-04-30 | Local | High | 151 | Adults (18-64 years) | Female | Sequential | Pregnant or parturient women | 2% | EUROIMMUN - IgG - Anti-SARS-CoV-2 ELISA IgG,EUROIMMUN - IgA - Anti-SARS-CoV-2 ELISA IgA | 0.997, 0.983 | Complex test algorithm: Manual calculation |
| Germany (Gangelt) | Hendrik Streeck (University of Bonn)[410] | 2020-03-31 - 2020-04-06 | Local | Moderate | 919 | Multiple groups | All | Probability | Household and community samples | 13.6% | EUROIMMUN - IgG - Anti-SARS-CoV-2 ELISA IgG | 0.9, 1 | Validated by manufacturer |
| Greece (Athens) | Helena Maltezou (University of Athens)[411] | 2021-06-01 - 2021-06-22 | Local | High | 901 | Multiple groups | All | Convenience | Residual sera | 55.7% | Abbott Laboratories - IgG - SARS-CoV-2 IgG II Quant | 0.994, 0.996 | Validated by manufacturer |
| Greece (Deskati) | Ourania Kotsiou (University of Thessaly)[412] | 2021-06-06 - 2021-06-06 | Local | High | 69 | Multiple groups | All | Convenience | Household and community samples | 79.7% | Abbott Laboratories - IgG - SARS-CoV-2 IgG II Quant | 0.994, 0.996 | Validated by manufacturer |
| Greece (Athens) | Helena Maltezou (University of Athens)[411] | 2021-03-01 - 2021-03-22 | Local | High | 764 | Multiple groups | All | Convenience | Residual sera | 11.6% | Abbott Laboratories - IgG - SARS-CoV-2 IgG II Quant | 0.994, 0.996 | Validated by manufacturer |
| Greece (Deskati) | Ourania S. Kotsiou (University of Thessaly)[413] | 2021-01-29 - 2021-01-29 | Local | High | 388 | Multiple groups | All | Convenience | Household and community samples | 17.8% | Prognosis Biotech - IgG, IgM, IgA - Rapid Test 2019-nCoV Total Ig,Abbott Laboratories - IgG - Abbott Architect SARS-CoV-2 IgG | 0.999, 1 | Complex test algorithm: Manual calculation |
| Greece | Zacharoula Bogogiannidou (University of Thessaly)[414] | 2020-04-01 - 2020-04-30 | National | High | 4,511 | Multiple groups | All | Convenience | Residual sera | 0.4% | Abbott Laboratories - IgG - Abbott Architect SARS-CoV-2 IgG | 0.99, 1 | Validated by manufacturer |
| Iceland | Daniel Gudbjartsson (deCODE Genetics)[415] | 2020-04-27 - 2020-06-12 | National | High | 23,452 | Multiple groups | All | Convenience | Household and community samples | 0.3% | Roche Diagnostics - IgG, IgM, IgA - Elecsys  Anti‐SARS‐CoV‐2 (S),Beijing Wantai Biological - IgG, IgM, IgA - Wantai SARS-CoV-2 Total Ab ELISA | 0.955, 1 | Complex test algorithm: Manual calculation |
| Ireland | Laura Heavey (Health Protection Surveillance Centre)[416] | 2020-06-22 - 2020-07-16 | Local | Low | 913 | Multiple groups | All | Probability | Household and community samples | 3.1% | Abbott Laboratories - IgG - Abbott Architect SARS-CoV-2 IgG,Beijing Wantai Biological - IgG, IgM, IgA - Wantai SARS-CoV-2 Total Ab ELISA | 0.957, 1 | Complex test algorithm: Manual calculation |
| Ireland | Laura Heavey (Health Protection Surveillance Centre)[416] | 2020-06-22 - 2020-07-16 | Local | Low | 820 | Multiple groups | All | Probability | Household and community samples | 0.6% | Abbott Laboratories - IgG - Abbott Architect SARS-CoV-2 IgG,Beijing Wantai Biological - IgG, IgM, IgA - Wantai SARS-CoV-2 Total Ab ELISA | 0.957, 1 | Complex test algorithm: Manual calculation |
| Israel (Jerusalem) | Adin Breuer (Shaare Zedek Medical Center)[417] | 2020-10-18 - 2021-01-12 | Local | Moderate | 1,138 | Children and Youth (0-17 years) | All | Convenience | Residual sera | 10% | Abbott Laboratories - IgG - Abbott Architect SARS-CoV-2 IgG,DiaSorin - IgG - Liaison SARS-CoV-2 S1/S2 IgG | 0.964, 1 | Complex test algorithm: Manual calculation |
| Israel (Modiin) | Yael Gozlan (Israel Ministry of Health)[418] | 2020-09-01 - 2020-09-30 | Local | High | 283 | Multiple groups | All | Convenience | Household and community samples | 16.6% | Abbott Laboratories - IgG - Abbott Architect SARS-CoV-2 IgG,DiaSorin - IgG - Liaison SARS-CoV-2 S1/S2 IgG,NA - IgG - Author designed (ELISA) -Spike | N/A | Complex test algorithm: Manual review |
| Israel | Anat Ekka Zohar (Maccabi Healthcare Services)[419] | 2020-06-28 - 2020-07-27 | National | High | 15,459 | Multiple groups | All | Convenience | Residual sera | 1% | Abbott Laboratories - IgG - Abbott Architect SARS-CoV-2 IgG,DiaSorin - IgG - LIAISON  SARS-CoV-2 TrimericS IgG | 0.977, 1 | Complex test algorithm: Manual calculation |
| Italy (Foggia) | Francescopaolo Antonucci (University Hospital “Ospedali Riuniti” Foggia)[420] | 2021-03-01 - 2021-03-15 | Local | Moderate | 484 | Multiple groups | All | Convenience | Blood donors | 18.8% | Ortho Clinical Diagnostics Inc. - IgG - VITROS Immunodiagnostic Products Anti-SARS-CoV-2 IgG,Ortho Clinical Diagnostics Inc. - IgG, IgM, IgA - VITROS Immunodiagnostic Products Anti-SARS-CoV-2 Total | 1, 1 | Complex test algorithm: Manual calculation |
| Italy (Campania) | Immacolata Polvere (Università degli Studi del Sannio)[421] | 2020-09-01 - 2020-09-30 | Local | Moderate | 1,383 | Seniors (65+ years) | All | Convenience | Household and community samples | 4.7% | Beijing Lepu Medical Technology - IgG, IgM - Leccurate SARS-CoV-2 Antibody Rapid Test Kit (Colloidal Gold Immunochromatography) | 0.989, 0.976 | Validated by manufacturer |
| Italy (Gardena Valley ( Ortisei, Santa Cristina, and Selva, the main municipalities)) | Roberto Melotti (Hospital of Bolzano)[422] | 2020-05-26 - 2020-06-08 | Local | Low | 2,106 | Multiple groups | All | Probability | Household and community samples | 26.2% | Abbott Laboratories - IgG - Abbott Architect SARS-CoV-2 IgG | 0.99, 1 | Validated by manufacturer |
| Italy (Castiglione D’Adda) | Gabriele Pagani (University of Milan)[423] | 2020-05-18 - 2020-06-07 | Local | Moderate | 4,143 | Multiple groups | All | Probability | Household and community samples | 22.2% | PRIMA Lab S.A. - IgG, IgM - PRIMA COVID-19 IgG/IgM Rapid Test | 0.92, 0.98 | Validated by manufacturer |
| Italy (Ariano Irpino) | Pellegrino Cerino (The Zoo-Prophylactic Institute of Southern Italy)[424] | 2020-05-16 - 2020-05-19 | Local | Moderate | 13,218 | Multiple groups | All | Convenience | Household and community samples | 5.6% | Roche Diagnostics - IgG, IgM, IgA - Elecsys  Anti‐SARS‐CoV‐2 (N) | 1, 0.995 | Validated by manufacturer |
| Italy (Foggia) | Jose Ramon Fiore (University of Foggia)[425] | 2020-05-01 - 2020-05-31 | Local | Moderate | 904 | Adults (18-64 years) | All | Convenience | Blood donors | 1% | Snibe Co. Ltd (Shenzhen New Industries Biomedical Engineering Co. Ltd) - IgG, IgM - MAGLUMI 2019-nCoV IgM/IgG | 0.94, 0.987 | Validated by manufacturer |
| Italy (Vo) | Ilaria Dorigatti (University of Padova)[426] | 2020-05-01 - 2020-05-31 | Local | Moderate | 2,602 | Multiple groups | All | Probability | Household and community samples | 6.2% | Abbott Laboratories - IgG - Abbott Architect SARS-CoV-2 IgG,Roche Diagnostics - IgG, IgM, IgA - Elecsys  Anti‐SARS‐CoV‐2 (N),DiaSorin - IgG - Liaison SARS-CoV-2 S1/S2 IgG | N/A | Complex test algorithm: Manual review |
| Italy (Lodi) | Irene Cassaniti (IRCCS Policlinico)[427] | 2020-03-18 - 2020-06-24 | Local | High | 1,922 | Multiple groups | All | Convenience | Blood donors | 19.7% | DiaSorin - IgG - Liaison SARS-CoV-2 S1/S2 IgG | 0.974, 0.985 | Validated by manufacturer |
| Italy (Verona) | Massimo Guerriero (Instituto di Ricovero e Cura A Caratterre)[428] | 2020-04-25 - 2020-05-08 | Local | Moderate | 1,515 | Multiple groups | All | Probability | Household and community samples | 2.7% | Abbott Laboratories - IgG - Abbott Architect SARS-CoV-2 IgG | 0.99, 1 | Validated by manufacturer |
| Italy (Lodi) | Alessandro Baracco (A.S.S.T.Lodi)[429] | 2020-04-23 - 2020-05-05 | Local | Moderate | 1,792 | Multiple groups | All | Probability | Household and community samples | 29.8% | DiaSorin - IgG - Liaison SARS-CoV-2 S1/S2 IgG | 0.974, 0.985 | Validated by manufacturer |
| Italy (Prato) | Anna Cavaliere (Santo Stefano Hospital)[430] | 2020-04-04 - 2020-05-16 | Local | High | 134 | Adults (18-64 years) | Female | Sequential | Pregnant or parturient women | 4.5% | ACRO Biotech Inc - IgG, IgM - Acro Biotech COVID-19 IgM/IgG Rapid POC test | 0.977, 0.99 | Validated by manufacturer |
| Italy (Villa Caldari) | Francesa Cito (Istituto Zooprofilattico Sperimentale dell’Abruzzo e del Molise G. Caporale )[431] | 2020-04-18 - 2020-04-19 | Local | Moderate | 667 | Multiple groups | All | Probability | Household and community samples | 10.9% | Beijing Wantai Biological - IgG, IgM, IgA - Wantai SARS-CoV-2 Total Ab ELISA | 0.967, 0.975 | Validated by manufacturer |
| Italy (Milan) | Luca Valenti ( Fondazione IRCCS Ca’ Granda Ospedale Maggiore )[432] | 2020-03-30 - 2020-04-08 | Local | High | 60 | Adults (18-64 years) | All | Convenience | Blood donors | 6.7% | PRIMA Lab S.A. - IgG, IgM - PRIMA COVID-19 IgG/IgM Rapid Test | 0.92, 0.98 | Validated by manufacturer |
| Italy (Arezzo) | Alessandro Pancrazzi (Ospedale San Donato)[433] | 2020-03-17 - 2020-03-21 | Local | High | 516 | Adults (18-64 years) | All | Sequential | Residual sera | 13% | ACRO Biotech Inc - IgG, IgM - Acro Biotech COVID-19 IgM/IgG Rapid POC test | 0.977, 0.99 | Validated by manufacturer |
| Netherlands (Amsterdam) | Liza Coyer (University of Amsterdam)[434] | 2020-11-23 - 2021-06-04 | Local | High | 2,075 | Multiple groups | All | Convenience | Household and community samples | 23.6% | Beijing Wantai Biological - IgG, IgM, IgA - Wantai SARS-CoV-2 Total Ab ELISA | 0.967, 0.975 | Validated by manufacturer |
| Netherlands (Limburg) | Demi Pagen (South Limburg Public Health Service)[435] | 2020-10-28 - 2020-12-23 | Subnational | High | 10,001 | Multiple groups | All | Convenience | Household and community samples | 19.5% | Beijing Wantai Biological - IgG, IgM, IgA - Wantai SARS-CoV-2 Total Ab ELISA | 0.967, 0.975 | Validated by manufacturer |
| Netherlands (Rotterdam) | I.L.M. Rotee ( Franciscus Gasthuis & Vlietland)[436] | 2020-10-19 - 2020-12-19 | Local | High | 240 | Children and Youth (0-17 years) | All | Sequential | Residual sera | 13.8% | Beijing Wantai Biological - IgG, IgM, IgA - Wantai SARS-CoV-2 Total Ab ELISA | 0.967, 0.975 | Validated by manufacturer |
| Netherlands (Rotterdam) | I.L.M. Rotee (Franciscus Gasthuis & Vlietland)[436] | 2020-07-19 - 2020-09-19 | Local | High | 209 | Children and Youth (0-17 years) | All | Sequential | Residual sera | 4.1% | Beijing Wantai Biological - IgG, IgM, IgA - Wantai SARS-CoV-2 Total Ab ELISA | 0.967, 0.975 | Validated by manufacturer |
| Netherlands (Amsterdam) | Liza Coyer (University of Amsterdam)[437] | 2020-06-24 - 2020-10-09 | Local | Moderate | 2,483 | Multiple groups | All | Probability | Household and community samples | 9.1% | Beijing Wantai Biological - IgG, IgM, IgA - Wantai SARS-CoV-2 Total Ab ELISA | 0.967, 0.975 | Validated by manufacturer |
| Netherlands (Amsterdam) | Maya Keuning (University of Amsterdam)[438] | 2020-04-12 - 2020-10-02 | Local | High | 487 | Children and Youth (0-17 years) | All | Convenience | Residual sera | 3.3% | Beijing Wantai Biological - IgG, IgM, IgA - Wantai SARS-CoV-2 Total Ab ELISA | 0.967, 0.975 | Validated by manufacturer |
| Netherlands | Ed Slot (Amsterdam UMC)[439] | 2020-04-01 - 2020-04-15 | National | High | 7,361 | Multiple groups | All | Sequential | Blood donors | 3.4% | Beijing Wantai Biological - IgG, IgM, IgA - Wantai Rapid Test for Total Antibody to SARS-CoV-2 | 0.947, 0.989 | Validated by manufacturer |
| Norway (Lorenskog) | Anne Eskild (University of Oslo)[440] | 2020-12-01 - 2020-12-31 | Local | Moderate | 418 | Adults (18-64 years) | Female | Sequential | Pregnant or parturient women | 5% | Roche Diagnostics - IgG, IgM, IgA - Elecsys  Anti‐SARS‐CoV‐2 (N),DiaSorin - IgG - Liaison SARS-CoV-2 S1/S2 IgG | 1, 0.98 | Complex test algorithm: Manual calculation |
| Poland (Katowice, Gliwice and Sosnowiec) | Jan Zejda (Slaski Uniwersytet Medyczny)[441] | 2020-10-15 - 2020-11-15 | Local | Moderate | 1,167 | Multiple groups | All | Probability | Household and community samples | 11.4% | EUROIMMUN - IgG - Anti-SARS-CoV-2 ELISA IgG | 0.9, 1 | Validated by manufacturer |
| Romania | Tudor Olariu (Victor Babes University of Medicine and Pharmacy)[442] | 2021-07-02 - 2021-09-02 | Local | High | 2,395 | Adults (18-64 years) | All | Sequential | Blood donors | 41% | Roche Diagnostics - IgG, IgM, IgA - Elecsys  Anti‐SARS‐CoV‐2 (N) | 1, 0.995 | Validated by manufacturer |
| Romania (Timisoara) | Tudor Olariu (Victor Babes University of Medicine and Pharmacy Timisoara)[443] | 2021-03-10 - 2021-06-10 | Local | High | 379 | Children and Youth (0-17 years) | All | Sequential | Residual sera | 46.7% | Roche Diagnostics - IgG, IgM, IgA - Elecsys  Anti‐SARS‐CoV‐2 (N) | 1, 0.995 | Validated by manufacturer |
| Romania | Tudor Rares Olariu (Victor Babes University of Medicine and Pharmacy)[444] | 2020-07-08 - 2020-09-01 | Local | Moderate | 2,115 | Adults (18-64 years) | All | Sequential | Blood donors | 1.5% | Roche Diagnostics - IgG, IgM, IgA - Elecsys  Anti‐SARS‐CoV‐2 (N) | 1, 0.995 | Validated by manufacturer |
| Spain (Catalonia) | Marianna Karachaliou (Instituto de Salud Global Barcelona)[445] | 2021-05-26 - 2021-07-21 | Subnational | High | 1,076 | Multiple groups | All | Convenience | Blood donors | 92.6% | NA - - Author designed (Multiplex) | 0.9578, 1 | Validated by developers |
| Spain (Madrid) | Vicente Soriano (UNIR Health Sciences School and Medical Centre)[446] | 2020-09-15 - 2020-12-24 | Local | High | 601 | Multiple groups | All | Sequential | Residual sera | 7.7% | PCL Inc - IgG, IgM - PCL COVID19 IgG/IgM Rapid Gold | 0.967, 1 | Validated by manufacturer |
| Spain (Catalonia) | Marianna Karachaliou (Barcelona Institute For Global Health)[185] | 2020-06-30 - 2020-11-15 | Subnational | High | 260 | Children and Youth (0-17 years) | All | Convenience | Household and community samples | 11.5% | NA - - Author designed (Multiplex) | N/A | Complex test algorithm: Manual review |
| Spain (Catalonia) | Manolis Kogevinas (Barcelona Institute for Global Health)[447] | 2020-05-17 - 2020-11-15 | Subnational | High | 4,103 | Adults (18-64 years) | All | Convenience | Household and community samples | 18.1% | NA - - Author designed (Luminex) | 0.9578, 1 | Validated by developers |
| Spain (Madrid) | Maria Vinuela (University Hospital Gregorio Marañón)[448] | 2020-05-06 - 2020-05-21 | Local | High | 100 | Adults (18-64 years) | Female | Sequential | Pregnant or parturient women | 13% | Abbott Laboratories - IgG - Abbott Architect SARS-CoV-2 IgG | 0.99, 1 | Validated by manufacturer |
| Spain (Madrid) | Vicente Soriano (UNIR Health Sciences School and Medical Centre)[446] | 2020-03-15 - 2020-06-30 | Local | High | 968 | Multiple groups | All | Sequential | Residual sera | 12.6% | PCL Inc - IgG, IgM - PCL COVID19 IgG/IgM Rapid Gold | 0.967, 1 | Validated by manufacturer |
| Spain (Barcelona) | Francesca Crovetto (Universitat de Barcelona)[449] | 2020-03-15 - 2020-05-31 | Local | High | 2,225 | Adults (18-64 years) | Female | Sequential | Pregnant or parturient women | 14.1% | Vircell S.L. - IgG - COVID-19 ELISA IgG,Ortho Clinical Diagnostics Inc. - IgG, IgM, IgA - VITROS Immunodiagnostic Products Anti-SARS-CoV-2 Total,NA - - Author designed (Luminex) | N/A | Complex test algorithm: Manual review |
| Spain (Madrid) | R. Alenda (Centro de Transfusión de la Comunidad de Madrid)[450] | 2020-03-15 - 2020-03-22 | Local | High | 89 | Multiple groups | All | Sequential | Blood donors | 0% | Quotient - IgG, IgM - MosaiQ COVID-19 Antibody Microarray | 1, 0.998 | Validated by manufacturer |
| Sweden (Stockholm) | Ake Lundkvist (Uppsala University )[451] | 2020-06-17 - 2020-06-18 | Local | High | 123 | Adults (18-64 years) | All | Probability | Household and community samples | 4.1% | Zhejiang Orient Gene Biotech - IgG, IgM - COVID-19 IgG/IgM Rapid Test | 0.972, 1 | Validated by manufacturer |
| Sweden (Stockholm) | Ake Lundkvist (Uppsala University )[451] | 2020-06-17 - 2020-06-18 | Local | High | 90 | Adults (18-64 years) | All | Probability | Household and community samples | 30% | Zhejiang Orient Gene Biotech - IgG, IgM - COVID-19 IgG/IgM Rapid Test | 0.972, 1 | Validated by manufacturer |
| Switzerland (Fribourg) | Emna El May (Swiss School of Public Health)[452] | 2021-05-20 - 2021-08-13 | Subnational | High | 504 | Multiple groups | All | Probability | Household and community samples | 78% | Swiss Federal Institute of Technology - IgG, IgA - Sensitive Anti-SARS-CoV-2 Spike Trimer Immunoglobulin Serological (SenASTrIS) | 0.966, 0.997 | Validated by independent authors/third party/non-developers |
| Switzerland (Geneva) | Silvia Stringhini (Geneva University Hospital )[453] | 2021-06-01 - 2021-07-07 | Local | Moderate | 3,355 | Multiple groups | All | Probability | Household and community samples | 27% | Roche Diagnostics - IgG, IgM, IgA - Elecsys  Anti‐SARS‐CoV‐2 (N) | N/A | Complex test algorithm: Manual review |
| Switzerland (Geneva) | Silvia Stringhini (Geneva University Hospitals)[453] | 2020-11-23 - 2020-12-23 | Local | Low | 4,000 | Multiple groups | All | Probability | Household and community samples | 20.5% | Roche Diagnostics - IgG, IgM, IgA - Elecsys  Anti‐SARS‐CoV‐2 (S) | 0.988, 1 | Validated by manufacturer |
| Switzerland (Southern Switzerland) | Elena Giuliano (Università della Svizzera Italiana)[454] | 2020-07-15 - 2020-12-15 | Subnational | High | 301 | Adults (18-64 years) | All | Sequential | Household and community samples | 3.3% | EUROIMMUN - IgG - Anti-SARS-CoV-2 ELISA IgG | 0.9, 1 | Validated by manufacturer |
| Switzerland (Zurich) | Marc Emmenegger (University of Zurich)[455] | 2020-07-01 - 2020-07-15 | Local | High | 1,067 | Adults (18-64 years) | All | Sequential | Blood donors | 0.7% | NA - - Author designed (ELISA) - MULTIPLEXED | 1, 1 | Validated by developers |
| Switzerland (Zurich) | Agne Ulyte (University of Zurich)[456] | 2020-06-16 - 2020-07-09 | Local | Low | 857 | Adults (18-64 years) | All | Probability | Household and community samples | 3.6% | NA - - Author designed (Luminex) | 0.983, 0.984 | Validated by developers |
| Switzerland (Geneva) | Stephanie Baggio (Geneva University Hospitals)[457] | 2020-06-01 - 2020-06-30 | Local | Moderate | 1,167 | Multiple groups | All | Convenience | Residual sera | 5.9% | EUROIMMUN - IgG - Anti-SARS-CoV-2 ELISA IgG | 0.9, 1 | Validated by manufacturer |
| Switzerland (Zurich) | Marc Emmenegger (University of Zurich)[455] | 2020-05-15 - 2020-07-15 | Local | High | 12,831 | Adults (18-64 years) | All | Sequential | Residual sera | 0.9% | NA - - Author designed (ELISA) - MULTIPLEXED | 1, 1 | Validated by developers |
| Switzerland (Grisons) | Gregorio Milani (Università della Svizzera Italiana)[458] | 2020-05-15 - 2020-05-31 | Local | High | 423 | Multiple groups | All | Convenience | Household and community samples | 2.6% | EUROIMMUN - IgG - Anti-SARS-CoV-2 ELISA IgG | 0.9, 1 | Validated by manufacturer |
| Switzerland (Zurich) | Marc Emmenegger (University of Zurich)[455] | 2020-04-01 - 2020-04-30 | Local | High | 1,469 | Adults (18-64 years) | All | Sequential | Blood donors | 1.2% | NA - - Author designed (ELISA) - MULTIPLEXED | 1, 1 | Validated by developers |
| Switzerland (Zurich) | Marc Emmenegger (University of Zurich)[455] | 2020-04-01 - 2020-04-30 | Local | High | 4,275 | Adults (18-64 years) | All | Sequential | Residual sera | 1.4% | NA - - Author designed (ELISA) - MULTIPLEXED | 1, 1 | Validated by developers |
| Switzerland (Zurich) | Marc Emmenegger (University of Zurich)[455] | 2020-03-15 - 2020-03-31 | Local | High | 3,806 | Adults (18-64 years) | All | Sequential | Residual sera | 0.3% | NA - - Author designed (ELISA) - MULTIPLEXED | 1, 1 | Validated by developers |
| The United Kingdom | Public Health Scotland (Public Health Scotland)[94] | 2021-12-20 - 2022-01-23 | National | High | 2,914 | Adults (18-64 years) | Female | Sequential | Pregnant or parturient women | 91% | Roche Diagnostics - IgG, IgM, IgA - Elecsys  Anti‐SARS‐CoV‐2 (S) | 0.988, 1 | Validated by manufacturer |
| The United Kingdom | Public Health Scotland (Public Health Scotland)[95] | 2021-11-15 - 2021-12-19 | National | High | 3,060 | Adults (18-64 years) | Female | Sequential | Pregnant or parturient women | 87.2% | Roche Diagnostics - IgG, IgM, IgA - Elecsys  Anti‐SARS‐CoV‐2 (S) | 0.988, 1 | Validated by manufacturer |
| The United Kingdom | Public Health Scotland (Public Health Scotland)[96] | 2021-10-11 - 2021-11-14 | National | High | 2,890 | Adults (18-64 years) | Female | Sequential | Pregnant or parturient women | 86.2% | Roche Diagnostics - IgG, IgM, IgA - Elecsys  Anti‐SARS‐CoV‐2 (S) | 0.988, 1 | Validated by manufacturer |
| The United Kingdom | Daniel Major-Smith (University of Bristol)[459] | 2021-05-01 - 2021-06-28 | Local | High | 66 | Adults (18-64 years) | All | Convenience | Household and community samples | 36.4% | Roche Diagnostics - IgG, IgM, IgA - Elecsys  Anti‐SARS‐CoV‐2 (S) | 0.988, 1 | Validated by manufacturer |
| The United Kingdom | Daniel Major-Smith (University of Bristol)[459] | 2021-04-08 - 2021-06-28 | Local | High | 2,555 | Adults (18-64 years) | All | Convenience | Household and community samples | 96.4% | Roche Diagnostics - IgG, IgM, IgA - Elecsys  Anti‐SARS‐CoV‐2 (S) | 0.988, 1 | Validated by manufacturer |
| The United Kingdom | Sarah Beale (University College London)[460] | 2021-02-01 - 2021-06-08 | National | High | 3,775 | Multiple groups | All | Convenience | Household and community samples | 12.1% | Roche Diagnostics - IgG, IgM, IgA - Elecsys  Anti‐SARS‐CoV‐2 (N) | 1, 0.995 | Validated by manufacturer |
| The United Kingdom (London) | Katherine Gaskell (London School of Hygiene & Tropical Medicine)[461] | 2020-10-19 - 2020-12-07 | Local | Moderate | 1,242 | Multiple groups | All | Probability | Household and community samples | 64.3% | NA - - Not reported/ Unable to specify | 0.962, 0.974 | Validated by independent authors/third party/non-developers |
| The United Kingdom | Clarissa Oeser (UK Health Security Agency (UKHSA, formerly PHE))[97] | 2020-09-01 - 2020-10-31 | National | High | 119 | Children and Youth (0-17 years) | All | Convenience | Residual sera | 6.7% | Roche Diagnostics - IgG, IgM, IgA - Elecsys  Anti‐SARS‐CoV‐2 (S) | 0.988, 1 | Validated by manufacturer |
| The United Kingdom (Blackpool) | Jane Oakey (NHS Foundation Trust )[462] | 2020-08-01 - 2020-08-31 | Local | High | 225 | Multiple groups | All | Convenience | Residual sera | 5.3% | Abbott Laboratories - IgG - Abbott Architect SARS-CoV-2 IgG | 0.99, 1 | Validated by manufacturer |
| The United Kingdom (Scotland) | Hani Abo-Leyah (University of Dundee)[463] | 2020-05-28 - 2020-09-02 | Subnational | High | 231 | Multiple groups | All | Sequential | Residual sera | 4.8% | Siemens - IgG, IgM, IgA - Atellica  IM SARS-CoV-2 Total (COV2T) | 0.985, 0.998 | Validated by manufacturer |
| The United Kingdom (South East England) | Philippa M Wells (King’s College London)[464] | 2020-04-27 - 2020-06-02 | Subnational | High | 431 | Multiple groups | All | Convenience | Household and community samples | 12% | NA - - Author designed (ELISA) - MULTIPLEXED | 0.9, 1 | Validated by independent authors/third party/non-developers |
| The United Kingdom (Oxford) | Sheila F Lumley (University of Oxford)[465] | 2020-04-14 - 2020-06-15 | Local | Moderate | 1,000 | Adults (18-64 years) | Female | Sequential | Pregnant or parturient women | 5.3% | NA - IgG - Author designed (ELISA) -Spike | 0.991, 0.99 | Validated by independent authors/third party/non-developers |
| The United Kingdom (Dublin) | Kate Glennon (University College Dublin)[466] | 2020-05-04 - 2020-05-15 | Local | High | 598 | Adults (18-64 years) | Female | Convenience | Pregnant or parturient women | 1.7% | Abbott Laboratories - IgG - Abbott Architect SARS-CoV-2 IgG,Roche Diagnostics - IgG, IgM, IgA - Elecsys  Anti‐SARS‐CoV‐2 (N) | 0.99, 1 | Complex test algorithm: Manual calculation |
| The United Kingdom (London) | Gayatri Amirthalingam (Public Health England)[467] | 2020-05-01 - 2020-05-03 | Local | Moderate | 974 | Multiple groups | All | Convenience | Blood donors | 13% | EUROIMMUN - IgG - Anti-SARS-CoV-2 ELISA IgG | 0.9, 1 | Validated by manufacturer |
| The United Kingdom (Leicester) | Paul William Bird (University of Leicester)[468] | 2020-03-16 - 2020-06-03 | Local | Moderate | 1,779 | Multiple groups | All | Probability | Household and community samples | 11.3% | DiaSorin - IgG - Liaison SARS-CoV-2 S1/S2 IgG | 0.974, 0.985 | Validated by manufacturer |
| The United Kingdom (Greater Glasgow area) | Ellen C Hughes (University of Glasgow)[469] | 2020-03-16 - 2020-05-24 | Local | Low | 6,635 | Multiple groups | All | Probability | Residual sera | 7.8% | NA - IgG - Author designed (ELISA) -Spike | 0.9531, 0.972 | Validated by developers |
| The United Kingdom | Public Health England (Public Health England)[111] | 2020-02-01 - 2020-03-31 | National | High | 443 | Children and Youth (0-17 years) | All | Probability | Residual sera | 4.9% | Abbott Laboratories - IgG - Abbott Architect SARS-CoV-2 IgG | 0.99, 1 | Validated by manufacturer |

#### South-East Asia region

| Country (Location) | Author (Organization) | Sampling Dates (YMD) | Geographic scope | Overall risk of bias | Sample size | Age | Sex | Sampling method | Sample frame | Seroprevalence (95% CI) | Test Manufacturers - Isotypes - Names | Test Sens, Spec | Sens, Spec Source |
| --- | --- | --- | --- | --- | --- | --- | --- | --- | --- | --- | --- | --- | --- |
| Bangladesh - HRP (Sitakunda subdistrict) | Taufiqur Bhuiyan (icddr,b [International Centre for Diarrhoeal Disease Research, Bangladesh])[470] | 2021-03-27 - 2021-06-13 | Local | Low | 2,307 | Multiple groups | All | Probability | Household and community samples | 62.5% | Beijing Wantai Biological - IgG, IgM, IgA - Wantai SARS-CoV-2 Total Ab ELISA | 0.967, 0.975 | Validated by manufacturer |
| Bangladesh - HRP (Dhaka) | Zabed Ahmed (Bangladesh Reference Institute for Chemical Measurements )[471] | 2020-10-15 - 2021-02-15 | Local | High | 239 | Adults (18-64 years) | All | Convenience | Household and community samples | 42.2% | Erba Manneim - IgG - ErbaLisa  COVID-19 IgG semi quantitative kit | 0.991, 0.993 | Validated by manufacturer |
| Bangladesh - HRP (FDMN populations in Cox’s Bazar Rohiyanga camps) | Prof. Dr. Tahmina Shirin (IEDCR)[472] | 2020-12-01 - 2020-12-30 | Local | Moderate | 3,446 | Multiple groups | All | Probability | Household and community samples | 58% | Beijing Wantai Biological - IgG IgM IgA - Wantai SARS-CoV-2 Total Ab ELISA | 0.967, 0.975 | Validated by manufacturer |
| India (Vellore) | Divya Dayanand (Christian Medical College)[473] | 2021-07-01 - 2021-07-31 | Local | Low | 1,205 | Multiple groups | All | Probability | Household and community samples | 71.6% | InBios International, Inc. - IgG - anti-SARS-CoV-2 IgM and IgG | 0.919, 0.99 | Validated by manufacturer |
| India (Athagar) | Pujarini Dash (Indian Council of Medical Research–Regional Medical Research Centre)[474] | 2021-03-15 - 2021-04-15 | Local | Moderate | 887 | Multiple groups | All | Probability | Household and community samples | 60.3% | Roche Diagnostics - IgG, IgM, IgA - Elecsys  Anti‐SARS‐CoV‐2 (N) | 1, 0.995 | Validated by manufacturer |
| India (Badamba) | Pujarini Dash (Indian Council of Medical Research–Regional Medical Research Centre)[474] | 2021-03-15 - 2021-04-15 | Local | Moderate | 862 | Multiple groups | All | Probability | Household and community samples | 55.5% | Roche Diagnostics - IgG, IgM, IgA - Elecsys  Anti‐SARS‐CoV‐2 (N) | 1, 0.995 | Validated by manufacturer |
| India (Narsinghpur) | Pujarini Dash (Indian Council of Medical Research–Regional Medical Research Centre)[474] | 2021-03-15 - 2021-04-15 | Local | Moderate | 988 | Multiple groups | All | Probability | Household and community samples | 39.8% | Roche Diagnostics - IgG, IgM, IgA - Elecsys  Anti‐SARS‐CoV‐2 (N) | 1, 0.995 | Validated by manufacturer |
| India (Tigiria) | Pujarini Dash (Indian Council of Medical Research–Regional Medical Research Centre)[474] | 2021-03-15 - 2021-04-15 | Local | Moderate | 885 | Multiple groups | All | Probability | Household and community samples | 63.3% | Roche Diagnostics - IgG, IgM, IgA - Elecsys  Anti‐SARS‐CoV‐2 (N) | 1, 0.995 | Validated by manufacturer |
| India (Chennai) | Matt Hitchings (The Johns Hopkins Bloomberg School of Public Health)[475] | 2021-01-15 - 2021-05-15 | Local | Moderate | 4,629 | Multiple groups | All | Probability | Household and community samples | 62% | Abbott Laboratories - IgG - AdviseDX SARS-CoV-2 anti-IgG II assay | 0.981, 0.996 | Validated by manufacturer |
| India (Ahmedabad) | Om Prakash (Nathiba Hargovandas Lakhmichand (NHL) Municipal Medical College)[476] | 2021-02-22 - 2021-03-01 | Local | Moderate | 10,136 | Multiple groups | All | Stratified non-probability | Household and community samples | 27.9% | Zydus Diagnostics - IgG - Covid Kavach™ Anti-SARS CoV-2 IgG Antibody Detection ELISA | 0.987, 1 | Validated by manufacturer |
| India (Hyderabad) | Avula Laxmaiah (CSIR-CentreForCellularAndMolecularBiology)[477] | 2021-01-08 - 2021-01-24 | Local | Moderate | 9,363 | Multiple groups | All | Probability | Household and community samples | 54.2% | Roche Diagnostics - IgG, IgM, IgA - Elecsys  Anti‐SARS‐CoV‐2 (N) | 1, 0.995 | Validated by manufacturer |
| India (Vellore) | Divya Dayanand (Christian Medical College)[473] | 2021-01-01 - 2021-01-31 | Local | Low | 1,228 | Multiple groups | All | Probability | Household and community samples | 28.5% | InBios International, Inc. - IgG - anti-SARS-CoV-2 IgM and IgG | 0.919, 0.99 | Validated by manufacturer |
| India (South Andaman district) | Deepak Kumar (Andaman and Nicobar Islands Institute of Medical Sciences)[478] | 2020-12-15 - 2021-02-14 | Local | Moderate | 4,089 | Multiple groups | All | Probability | Household and community samples | 39.7% | Erba Manneim - IgG - ErbaLisa  COVID-19 IgG semi quantitative kit | 0.991, 0.993 | Validated by manufacturer |
| India (Jabalpur) | Sudarshan Ramaswamy (National Centre for Disease Control)[479] | 2020-12-11 - 2020-12-21 | Local | Low | 9,279 | Multiple groups | All | Probability | Household and community samples | 28.7% | Zydus Diagnostics - IgG - Covid Kavach™ Anti-SARS CoV-2 IgG Antibody Detection ELISA | 0.987, 1 | Validated by manufacturer |
| India (Cuttack) | Smita Mahapatra (Sriram Chandra Bhanja Medical College and Hospital)[480] | 2020-11-01 - 2021-01-31 | Local | High | 1,032 | Adults (18-64 years) | All | Convenience | Blood donors | 35.9% | Abbott Laboratories - IgG - Abbott Architect SARS-CoV-2 IgG | 0.99, 1 | Validated by manufacturer |
| India (New Delhi) | Alpesh Goyal (All India Institue of Medical Sciences)[481] | 2020-08-15 - 2021-02-15 | Local | High | 240 | Adults (18-64 years) | All | Convenience | Multiple general populations | 45.4% | DiaSorin - IgG - Liaison SARS-CoV-2 S1/S2 IgG | 0.974, 0.985 | Validated by manufacturer |
| India (Tamil Nadu) | Rita Isaac (University of Edinburgh)[482] | 2020-11-06 - 2020-11-20 | Local | Moderate | 200 | Multiple groups | All | Probability | Household and community samples | 22% | Siemens - IgG, IgM, IgA - Atellica  IM SARS-CoV-2 Total (COV2T),Roche Diagnostics - IgG, IgM, IgA - Elecsys  Anti‐SARS‐CoV‐2 (N) | 1, 0.993 | Complex test algorithm: Manual calculation |
| India (Ahmedabad) | Om Prakash (Municipal Medical College)[483] | 2020-10-15 - 2020-10-31 | Local | Moderate | 17,009 | Multiple groups | All | Convenience | Household and community samples | 24.2% | Zydus Diagnostics - IgG - Covid Kavach™ Anti-SARS CoV-2 IgG Antibody Detection ELISA | 0.987, 1 | Validated by manufacturer |
| India (Srinagar) | Taha Ayub (Government Medical College Srinagar)[484] | 2020-10-17 - 2020-10-20 | Local | Moderate | 2,418 | Multiple groups | All | Probability | Household and community samples | 40.6% | Abbott Laboratories - IgG - Abbott Architect SARS-CoV-2 IgG | 0.99, 1 | Validated by manufacturer |
| India (Pimpri-Chinchwad) | Amitav Banerjee (Dr. D. Y. Patil Medical College, Hospital and Research Centre)[485] | 2020-10-07 - 2020-10-17 | Local | Moderate | 5,000 | Multiple groups | All | Probability | Household and community samples | 34% | Abbott Laboratories - IgG - Abbott Architect SARS-CoV-2 IgG | 0.99, 1 | Validated by manufacturer |
| India (Chennai) | Muthusamy Kumar (National Institute of Epidemiology)[486] | 2020-10-08 - 2020-10-15 | Local | Low | 6,366 | Multiple groups | All | Probability | Household and community samples | 30.4% | Abbott Laboratories - IgG - Abbott Architect SARS-CoV-2 IgG | 0.99, 1 | Validated by manufacturer |
| India (Bhubaneswar) | Jaya Singh Kshatri (Indian Council of Medical Research)[487] | 2020-09-01 - 2020-09-30 | Local | Low | 1,403 | Multiple groups | All | Probability | Household and community samples | 49% | Roche Diagnostics - IgG, IgM, IgA - Elecsys  Anti‐SARS‐CoV‐2 (N) | 1, 0.995 | Validated by manufacturer |
| India (Ahmedabad) | Om Prakash (AMC MET Medical College)[488] | 2020-08-15 - 2020-08-31 | Local | Moderate | 10,310 | Multiple groups | All | Probability | Household and community samples | 23.2% | Zydus Diagnostics - IgG - Covid Kavach™ Anti-SARS CoV-2 IgG Antibody Detection ELISA | 0.987, 1 | Validated by manufacturer |
| India (Mumbai) | Ullas Kolthur Seetharam (NITI-Aayog)[489] | 2020-08-16 - 2020-08-29 | Local | Low | 2,176 | Multiple groups | All | Probability | Household and community samples | 17.5% | Abbott Laboratories - IgG - Abbott Architect SARS-CoV-2 IgG | 0.99, 1 | Validated by manufacturer |
| India (Mumbai) | Ullas Kolthur Seetharam (NITI-Aayog)[489] | 2020-08-16 - 2020-08-29 | Local | Low | 3,024 | Multiple groups | All | Probability | Persons living in slums | 44.9% | Abbott Laboratories - IgG - Abbott Architect SARS-CoV-2 IgG | 0.99, 1 | Validated by manufacturer |
| India (Indore) | Salil Sakalle (National Centre for Disease Control)[490] | 2020-08-11 - 2020-08-23 | Local | Low | 7,103 | Multiple groups | All | Probability | Household and community samples | 7.7% | Zydus Diagnostics - IgG - Covid Kavach™ Anti-SARS CoV-2 IgG Antibody Detection ELISA | 0.987, 1 | Validated by manufacturer |
| India (Berhampur) | Jaya Singh Kshatri (ICMR-Regional Medical Research Centre)[491] | 2020-08-01 - 2020-08-31 | Local | Low | 1,375 | Multiple groups | All | Probability | Household and community samples | 31.1% | Roche Diagnostics - IgG, IgM, IgA - Elecsys  Anti‐SARS‐CoV‐2 (N) | 1, 0.995 | Validated by manufacturer |
| India (Bhubaneswar) | Jaya Singh Kshatri (ICMR-Regional Medical Research Centre)[491] | 2020-08-01 - 2020-08-31 | Local | Low | 1,319 | Multiple groups | All | Probability | Household and community samples | 5.2% | Roche Diagnostics - IgG, IgM, IgA - Elecsys  Anti‐SARS‐CoV‐2 (N) | 1, 0.995 | Validated by manufacturer |
| India (Rourkela) | Jaya Singh Kshatri (ICMR-Regional Medical Research Centre)[491] | 2020-08-01 - 2020-08-31 | Local | Low | 1,452 | Multiple groups | All | Probability | Household and community samples | 24.6% | Roche Diagnostics - IgG, IgM, IgA - Elecsys  Anti‐SARS‐CoV‐2 (N) | 1, 0.995 | Validated by manufacturer |
| India (Malegaon) | Pallavi Saple (The Municipality of Malegaon )[492] | 2020-07-25 - 2020-08-20 | Local | High | 336 | Multiple groups | All | Stratified non-probability | Household and community samples | 40% | NA - - Not reported/ Unable to specify | 0.93, 1 | Validated by independent authors/third party/non-developers |
| India (Tamil Nadu) | Rita Isaac (University of Edinburgh)[482] | 2020-07-06 - 2020-08-20 | Local | Moderate | 501 | Multiple groups | All | Probability | Household and community samples | 2.2% | Siemens - IgG, IgM, IgA - Atellica  IM SARS-CoV-2 Total (COV2T),Roche Diagnostics - IgG, IgM, IgA - Elecsys  Anti‐SARS‐CoV‐2 (N) | 1, 0.993 | Complex test algorithm: Manual calculation |
| India (Chennai) | Sriram Selvaraju (National Institute of Epidemiology)[493] | 2020-07-01 - 2020-07-31 | Local | Low | 12,405 | Multiple groups | All | Probability | Household and community samples | 18.7% | Abbott Laboratories - IgG - Abbott Architect SARS-CoV-2 IgG | 0.99, 1 | Validated by manufacturer |
| India (Mumbai) | Anup Malani (Tata Institute for Fundamental Research)[494] | 2020-06-29 - 2020-07-19 | Local | Low | 2,702 | Multiple groups | All | Probability | Household and community samples | 16% | Abbott Laboratories - IgG - Abbott Architect SARS-CoV-2 IgG | 0.99, 1 | Validated by manufacturer |
| India (Mumbai) | Anup Malani (Tata Institute for Fundamental Research)[494] | 2020-06-29 - 2020-07-19 | Local | Low | 4,202 | Multiple groups | All | Probability | Persons living in slums | 54.1% | Abbott Laboratories - IgG - Abbott Architect SARS-CoV-2 IgG | 0.99, 1 | Validated by manufacturer |
| India (Ahmedabad) | Om Prakash ( Municipal Medical College )[495] | 2020-06-16 - 2020-07-07 | Local | Moderate | 29,891 | Multiple groups | All | Convenience | Residual sera | 17.6% | Zydus Diagnostics - IgG - Covid Kavach™ Anti-SARS CoV-2 IgG Antibody Detection ELISA | 0.987, 1 | Validated by manufacturer |
| India | Hem Chandra Pandey (All India Institute of Medical Sciences )[496] | 2020-04-15 - 2020-07-15 | Local | High | 1,191 | Adults (18-64 years) | All | Convenience | Blood donors | 9.5% | Abbott Laboratories - IgG - Abbott Architect SARS-CoV-2 IgG | 0.99, 1 | Validated by manufacturer |
| India (Karnataka) | Sindhulina Chandrasingh (Bangalore Baptist Hospital)[497] | 2020-05-15 - 2020-06-15 | Subnational | High | 210 | Multiple groups | All | Convenience | Blood donors | 0% | Roche Diagnostics - IgG, IgM, IgA - Elecsys  Anti‐SARS‐CoV‐2 (N) | 1, 0.995 | Validated by manufacturer |
| India (Mumbai) | Viswanath Billa (University of Pittsburgh Medical Center)[498] | 2020-03-24 - 2020-05-17 | Local | High | 110 | Multiple groups | All | Convenience | Residual sera | 37.3% | EUROIMMUN - IgG - Anti-SARS-CoV-2 ELISA IgG | 0.9, 1 | Validated by manufacturer |
| Maldives (Male’) | Raheema Abdul-Raheem (Maldives National University)[499] | 2020-10-15 - 2020-11-16 | Local | Moderate | 1,940 | Multiple groups | All | Probability | Household and community samples | 13.4% | Ortho Clinical Diagnostics Inc. - IgG, IgM, IgA - VITROS Immunodiagnostic Products Anti-SARS-CoV-2 Total | 1, 1 | Validated by manufacturer |

#### Western Pacific region

| Country (Location) | Author (Organization) | Sampling Dates (YMD) | Geographic scope | Overall risk of bias | Sample size | Age | Sex | Sampling method | Sample frame | Seroprevalence (95% CI) | Test Manufacturers - Isotypes - Names | Test Sens, Spec | Sens, Spec Source |
| --- | --- | --- | --- | --- | --- | --- | --- | --- | --- | --- | --- | --- | --- |
| Australia (Sydney, Melbourne, Brisbane, Adelaide, Perth) | Kristine Macartney, Nicholas Wood, Archana Koirala (Australian National Centre for Immunisation Research and Surveillance)[@] | 2020-11-01 - 2021-03-13 | Subnational | High | 1,678 | Multiple groups | All | Convenience | Residual sera | 0.4% (95% CI 0.2-0.9%) | Beijing Wantai Biological - IgG IgM IgA - Wantai SARS-CoV-2 Total Ab ELISA | 0.967, 0.975 | Validated by manufacturer |
| Australia (Metropolitan Melbourne) | Dorothy Machalek (University of New South Wales)[500] | 2020-11-23 - 2020-12-17 | Local | Low | 4,799 | Adults (18-64 years) | All | Probability | Blood donors | 1.6% | Beijing Wantai Biological - IgG, IgM, IgA - Wantai SARS-CoV-2 Total Ab ELISA | 0.967, 0.975 | Validated by manufacturer |
| Australia (Melbourne) | Dorothy Machalek (The Kirby Institue at the University of New South Wales and Australian National Centre for Immunisation Research and Surveillance)[501] | 2020-11-23 - 2020-12-17 | Local | High | 4,799 | Multiple groups | All | Convenience | Blood donors | 1.6% (95% CI 1.3-2%) | Beijing Wantai Biological - IgG IgM IgA - Wantai SARS-CoV-2 Total Ab ELISA | 0.967, 0.975 | Validated by manufacturer |
| Australia (Sydney) | Heather F Gidding (Children’s Hospital at Westmead)[502] | 2020-05-04 - 2020-05-23 | Local | Moderate | 560 | Adults (18-64 years) | Female | Probability | Pregnant or parturient women | 1% | NA - IgG, IgM - Author designed (IFA) - MULTIPLEXED | 0.907, 0.993 | Validated by independent authors/third party/non-developers |
| Australia (Sydney) | Heather F Gidding (Children’s Hospital at Westmead)[502] | 2020-04-20 - 2020-05-22 | Local | Moderate | 3,231 | Multiple groups | All | Probability | Residual sera | 0.6% | NA - - Author designed (IFA) - Unknown | 0.907, 0.993 | Validated by independent authors/third party/non-developers |
| China (Taipei) | Hsiang-Ling Ho (Taipei Veterans General Hospital)[503] | 2020-07-06 - 2020-07-08 | Local | Moderate | 4,988 | Multiple groups | All | Sequential | Residual sera | 0.1% | Academia Sinica - IgG, IgM - Academia Sinica ELISA assay,Roche Diagnostics - IgG, IgM, IgA - Elecsys  Anti‐SARS‐CoV‐2 (N) | 1, 1 | Complex test algorithm: Manual calculation |
| China (Wuhan) | Lu He (Renmin Hospital of Wuhan University)[504] | 2020-05-15 - 2020-05-29 | Local | Moderate | 4,454 | Multiple groups | All | Convenience | Household and community samples | 2.9% | Zhuhai Livzon Diagnostics Inc - IgG, IgM - 2019-nCoV IgG/IgM Antibody Detection Kit | 0.906, 0.992 | Validated by manufacturer |
| China (Shenzen) | Zhen Zhang (Shenzhen Center for Disease Control and Prevention)[505] | 2020-04-17 - 2020-04-23 | Local | High | 350 | Multiple groups | All | Convenience | Household and community samples | 0.3% | Beijing Wantai Biological - IgG, IgM, IgA - Wantai SARS-CoV-2 Total Ab ELISA | 0.967, 0.975 | Validated by manufacturer |
| China (Shenzen) | Zhen Zhang (Shenzhen Center for Disease Control and Prevention)[505] | 2020-04-17 - 2020-04-23 | Local | High | 50 | Multiple groups | All | Convenience | Household and community samples | 0% | Beijing Wantai Biological - IgG, IgM, IgA - Wantai SARS-CoV-2 Total Ab ELISA | 0.967, 0.975 | Validated by manufacturer |
| China (Guangzhou) | Ru Xu (Institute of Blood Transfusion)[506] | 2020-03-23 - 2020-04-02 | Local | Moderate | 2,199 | Adults (18-64 years) | All | Probability | Blood donors | 0.3% | Beijing Wantai Biological - IgG, IgM, IgA - Wantai SARS-CoV-2 Total Ab ELISA | 0.967, 0.975 | Validated by manufacturer |
| China (Liaoning) | Jin Zhang (Shengjing Hospital of China Medical University)[507] | 2020-01-21 - 2020-02-16 | Subnational | High | 225 | Multiple groups | All | Convenience | Residual sera | 0.4% | Shenzhen Yhlo Biotech Co. Ltd - IgG, IgM - iFlash-SARS-CoV-2 IgM/IgG | 1, 0.9956 | Validated by independent authors/third party/non-developers |
| Japan (Yokohama) | Kosuke Mori (Yokohama Municipal Citizen’s Hospital)[508] | 2021-02-01 - 2021-05-31 | Local | High | 1,070 | Multiple groups | All | Convenience | Residual sera | 3.3% | Abbott Laboratories - IgG - SARS-CoV-2 IgG II Quant | 0.994, 0.996 | Validated by manufacturer |
| Japan (Okinawa Prefecture) | Kenji Mizumoto (Okinawa Prefecture Epidemiological Statistics and Analysis Committee)[509] | 2021-02-01 - 2021-02-28 | Local | Moderate | 582 | Multiple groups | All | Convenience | Household and community samples | 1.4% | NA - IgG - Author designed (ELISA) -Spike | 0.925, 1 | Validated by independent authors/third party/non-developers |
| Japan (Okinawa Prefecture) | Kenji Mizumoto (Okinawa Prefecture Epidemiological Statistics and Analysis Committee)[509] | 2021-02-01 - 2021-02-28 | Local | Moderate | 123 | Multiple groups | All | Convenience | Household and community samples | 1.6% | NA - IgG - Author designed (ELISA) -Spike | 0.925, 1 | Validated by independent authors/third party/non-developers |
| Japan (Okinawa Prefecture) | Kenji Mizumoto (Okinawa Prefecture Epidemiological Statistics and Analysis Committee)[509] | 2020-10-01 - 2020-12-31 | Local | Moderate | 1,448 | Multiple groups | All | Convenience | Household and community samples | 0.6% | NA - IgG - Author designed (ELISA) -Spike | 0.925, 1 | Validated by independent authors/third party/non-developers |
| Japan (Okinawa Prefecture) | Kenji Mizumoto (Okinawa Prefecture Epidemiological Statistics and Analysis Committee)[509] | 2020-10-01 - 2020-12-31 | Local | Moderate | 144 | Multiple groups | All | Convenience | Household and community samples | 0% | NA - IgG - Author designed (ELISA) -Spike | 0.925, 1 | Validated by independent authors/third party/non-developers |
| Japan (Okinawa Prefecture) | Kenji Mizumoto (Okinawa Prefecture Epidemiological Statistics and Analysis Committee)[509] | 2020-07-01 - 2020-07-31 | Local | Moderate | 392 | Multiple groups | All | Convenience | Household and community samples | 0% | NA - IgG - Author designed (ELISA) -Spike | 0.925, 1 | Validated by independent authors/third party/non-developers |
| Japan (Yamagata) | Keita Morikane (Yamagata University)[510] | 2020-06-01 - 2020-06-04 | Local | High | 1,009 | Multiple groups | All | Sequential | Residual sera | 0.5% | Roche Diagnostics - IgG, IgM, IgA - Elecsys  Anti‐SARS‐CoV‐2 (N) | 1, 0.995 | Validated by manufacturer |
| Republic of Korea | Kwangmin Lee (Korea Disease Control and Prevention Agency)[511] | 2020-08-14 - 2020-10-31 | National | High | 1,379 | Multiple groups | All | Convenience | Residual sera | 0.2% | NA - - Author designed (Neutralization Assay) | 0.933, 0.971 | Validated by independent authors/third party/non-developers |
| Republic of Korea | Kwangmin Lee (Korea Disease Control and Prevention Agency)[511] | 2020-06-10 - 2020-08-13 | National | High | 1,440 | Multiple groups | All | Convenience | Residual sera | 0.1% | NA - - Author designed (Neutralization Assay) | 0.933, 0.971 | Validated by independent authors/third party/non-developers |
| Republic of Korea (Chungbuk Province) | Hye Won Jeong (Chungbuk National University)[512] | 2020-05-01 - 2020-07-17 | Local | Low | 3,981 | Multiple groups | All | Probability | Residual sera | 0.8% | NA - - Author designed (IFA) - Unknown | 0.995, 1 |  |
| Republic of Korea (Daegu) | Hye Won Jeong (Chungbuk National University)[512] | 2020-05-01 - 2020-07-17 | Local | Low | 3,268 | Multiple groups | All | Probability | Residual sera | 1.2% | NA - - Author designed (IFA) - Unknown | 0.995, 1 | Validated by developers |
| Republic of Korea | Kwangmin Lee (Korea Disease Control and Prevention Agency)[511] | 2020-04-21 - 2020-06-16 | National | High | 1,500 | Multiple groups | All | Convenience | Residual sera | 0% | NA - - Author designed (Neutralization Assay) | 0.933, 0.971 | Validated by independent authors/third party/non-developers |
| Singapore | Hannah E Clapham (National University of Singapore)[513] | 2020-11-15 - 2020-12-15 | National | High | 937 | Multiple groups | All | Convenience | Household and community samples | 0.2% | GenScript - IgG, IgM - cPass SARS-CoV-2 Surrogate Virus Neutralization Test ELISA Kit | 0.938, 0.994 | Validated by manufacturer |

##

## Table D. Risk of bias breakdown for all studies.

#### Africa region

| Country | Author | Item 1: Appropriate sample frame | Item 2: Probability sampling method | Item 3: Adequate sample size | Item 4: Subjects & setting described | Item 5: Good coverage of sample | Item 6: Sens>=90%, Spec>=97% | Item 7: Same test for all subjects | Item 8: Appropriate statistical analysis | Item 9: Adequate response rate | Overall risk of bias |
| --- | --- | --- | --- | --- | --- | --- | --- | --- | --- | --- | --- |
| Angola | Cruz Sebastiao [214] | No | No | No | Yes | No | Yes | Yes | No | N/A | High |
| Burkina Faso | Dr Isidore Traore [215] | Yes | Yes | Yes | Yes | Yes | Yes | Yes | No | Yes | Moderate |
| Cameroon | Kene Nwosu [216] | Yes | Yes | Yes | Yes | Yes | No | Yes | Yes | Yes | Low |
| Cameroon | Tiffany Harris [1] | Yes | No | Yes | Yes | Yes | Yes | Yes | Yes | Yes | Moderate |
| Central African Republic | Alexandre Manirakiza [217] | Yes | Yes | Yes | Yes | Yes | Yes | Yes | No | Unclear | Moderate |
| Congo | Novy Bonguili [218] | No | No | Yes | Yes | No | Unclear | Yes | No | N/A | High |
| Democratic Republic of the Congo | Antoine Nkuba [219] | Yes | Yes | Yes | Yes | Yes | Unclear | Yes | Yes | Yes | Low |
| Democratic Republic of the Congo | Makiala Mandanda Sheila [125] | Yes | Yes | Yes | Yes | Unclear | Yes | Yes | No | Yes | Moderate |
| Ethiopia | Nega Assefa [126] | No | Yes | Yes | No | Unclear | Yes | Yes | No | Unclear | Moderate |
| Ethiopia | Esayas Gudina [220] | Yes | No | No | No | Unclear | Yes | Yes | No | Unclear | High |
| Ethiopia | Esayas Gudina [220] | Yes | No | No | No | Unclear | Yes | Yes | No | Unclear | High |
| Ethiopia | Esayas Gudina [220] | Yes | No | No | No | Unclear | Yes | Yes | No | Unclear | High |
| Ethiopia | Esayas Gudina [220] | Yes | No | No | No | Unclear | Yes | Yes | No | Unclear | High |
| Ethiopia | Esayas Gudina [220] | Yes | No | No | No | Unclear | Yes | Yes | No | Unclear | High |
| Ethiopia | Esayas Gudina [220] | Yes | No | No | No | Unclear | Yes | Yes | No | Unclear | High |
| Ethiopia | Esayas Gudina [220] | Yes | No | No | No | Unclear | Yes | Yes | No | Unclear | High |
| Ethiopia | Esayas Gudina [220] | Yes | No | No | No | Unclear | Yes | Yes | No | Unclear | High |
| Ethiopia | Esayas Gudina [220] | Yes | No | No | No | Unclear | Yes | Yes | No | Unclear | High |
| Ethiopia | Esayas Gudina [220] | Yes | No | No | No | Unclear | Yes | Yes | No | Unclear | High |
| Ethiopia | Esayas Gudina [220] | Yes | No | No | No | Unclear | Yes | Yes | No | Unclear | High |
| Ethiopia | Esayas Gudina [220] | Yes | No | No | No | Unclear | Yes | Yes | No | Unclear | High |
| Ethiopia | Enyew Birru Tadesse [2] | Yes | Yes | Yes | Yes | Yes | Yes | Yes | Yes | Unclear | Low |
| Ethiopia | Tamrat Shaweno [222] | Yes | Yes | Yes | Yes | Yes | Yes | Yes | No | Yes | Moderate |
| Ethiopia | Saro Abdella [221] | Yes | Yes | Yes | Yes | Yes | Yes | Yes | Yes | Unclear | Low |
| Ethiopia | Saro Abdella [221] | Yes | Yes | Yes | Yes | Yes | Yes | Yes | Yes | Unclear | Low |
| Ethiopia | John Kempen [223] | No | No | No | Yes | No | Yes | Yes | No | Unclear | High |
| Gabon | Amandine Mveang Nzoghe [224] | No | No | Yes | No | Unclear | Yes | Yes | No | Unclear | High |
| Gabon | Dr Paulin ESSONE NDONG, Pr Edgar Brice NGOUNGOU, Pr Ayola Akim ADEGNIKA [3] | Yes | Yes | Yes | Yes | Unclear | Yes | Yes | No | Unclear | Moderate |
| Ghana | Irene Owusu Donkor [4] | Yes | Yes | Yes | Yes | Yes | Yes | Yes | No | Yes | Moderate |
| Ghana | Irene Owusu Donkor [4] | Yes | Yes | Yes | Yes | Yes | Yes | Yes | No | Yes | Moderate |
| Ghana | Irene Owusu Donkor [4] | Yes | Yes | Yes | Yes | Yes | Yes | Yes | No | Yes | Moderate |
| Ghana | Irene Owusu Donkor [4] | Yes | Yes | Yes | Yes | Yes | Yes | Yes | No | Yes | Moderate |
| Kenya | R Lucinde [225] | No | No | No | No | Yes | Unclear | Yes | No | N/A | High |
| Kenya | R Lucinde [225] | No | No | No | No | Yes | Unclear | Yes | No | N/A | High |
| Kenya | R Lucinde [225] | No | No | No | No | Yes | Unclear | Yes | No | N/A | High |
| Kenya | R Lucinde [225] | No | No | No | No | Yes | Unclear | Yes | No | N/A | High |
| Kenya | R Lucinde [225] | No | No | No | No | Yes | Unclear | Yes | No | N/A | High |
| Kenya | R Lucinde [225] | No | No | No | No | Yes | Unclear | Yes | No | N/A | High |
| Kenya | R Lucinde [225] | No | No | No | No | Yes | Unclear | Yes | No | N/A | High |
| Kenya | R Lucinde [225] | No | No | No | No | Yes | Unclear | Yes | No | N/A | High |
| Kenya | R Lucinde [225] | No | No | No | No | Yes | Unclear | Yes | No | N/A | High |
| Kenya | Anthony Etyang [227] | Yes | Yes | Yes | Yes | Yes | Unclear | Yes | Yes | Unclear | Low |
| Kenya | Anthony Etyang [227] | Yes | Yes | Yes | Yes | Yes | Unclear | Yes | Yes | Unclear | Low |
| Kenya | Anthony Etyang [227] | Yes | Yes | Yes | Yes | Yes | Unclear | Yes | Yes | Unclear | Low |
| Kenya | James Nyagwange [226] | No | No | No | No | No | Yes | Yes | No | N/A | High |
| Kenya | James Nyagwange [226] | No | No | No | No | No | Yes | Yes | No | N/A | High |
| Kenya | Isaac Ngere [230] | Yes | Yes | Yes | Yes | Yes | Yes | Yes | Yes | Yes | Low |
| Kenya | Sophie Uyoga [5] | No | No | Yes | Yes | No | Unclear | Yes | Yes | N/A | Moderate |
[truncated: 312,930 more chars]
