# Supplementary material for: Global SARS-CoV-2 seroprevalence from January 2020 to April 2022: A systematic review and meta-analysis of standardized population-based studies
Source: PLoS Med. 2022 Nov 10;19(11):e1004107. doi: 10.1371/journal.pmed.1004107 (PMC9648705; doi:10.1371/journal.pmed.1004107)
Supplement: S1 Acknowledgements — (DOCX) [file pmed.1004107.s002.docx]

S1 Acknowledgements

Unity Studies Collaborator Group

**Unity Studies Collaborator Group: Ximena Aguilera^19^, Sheikh Al-Shoteri^20^, Eman A Aly^8^, Mauricio Apablaza^21^, Rosemary A Audu^22^, Amal Barakat^8,23^, Abdulla S Bin-Ghouth^24^, Enyew Birru^25^, Dejan Bokonjic^26^, Shelly Bolotin^27,28^, Henry K Bosa^29,30^, Emily L Boucher^31^, Elma Catovic-Baralija^32^, Alexei Ceban^33^, Annie Chauma-Mwale^34^, Judy Chen^35^, Battogtokh Chimeddorj^36^, Pui Shan Chung^10^,  Cheryl Cohen^37^, Tienhan S Dabakuyo-Yonli^38^, Gabriel R Deveaux^31^, Boly Diop^39^, Titus H Divala^40^, Emily K Dokubo^41^, Irene O Donkor^42^, Claire Donnici^31^, Nathan Duarte^43^, Natalie A Duarte^44^, Timothy G Evans^7, 14^, Lee Fairlie^45^, Ousmane Faye^46^, Gudrun S Freidl^11^, Claudia González^19^, Tiffany G Harris^47,48^, Belinda L Herring^3^, Sopon Iamsirithaworn^49^, Gloria Icaza^50^, Rhoda Ila^51^, Natasha Ilincic^44^, Elsie A Ilori^52^, Francis Y Inbanathan^9^, Vicki Indenbaum^53^, John Kaldor^54^, Dayoung Kim^31^, Olatunji M Kolawole^55^, Jambo C Kondwani^56,57^, Tatiana Kuchuk^58^, Pritesh J Lalwani^59^, Moses Laman^60^, Evelyn Lavu^60^‡, Juliana Leite^12^, Michael Liu^61^, Emma Loeschnik^62^, Kristine Macartney^63^, Dorothy A Machalek^54,64^, Sheila Makiala-Mandanda^65,66^, Henri-Pierre Mallet^67^, Alexandre Manirakiza^68^, Pilly Mapira^51^, Pinyi N Mawien^69^, Puneet Misra^70^, Sanjin Musa^71,72^, Portia C Mutevedzi^73,74^, Osama A Najjar^75^, Sutthichai Nakphook^49^, Kim C Noel^16^, Zuridin Nurmatov^76^, Maria Ome-Kaius^60^, Eric M Osoro^77, 78^, Krishna P Paudel^79^, Sara Perlman-Arrow^7^, Sharif E Qaddomi^8,80^, Hude Quan^81^, Alissar Rady^82^, Hannah P Rahim^83^,  Muriel Ramírez-Santana^84^, Izzat Y Rayyan^8,80^, Angel Rodriguez^12^, Karampreet Sachathep^85,86^, Mitchell Segal^13^, Anabel Selemon^31^, Tahmina Shirin^87^, Kristen A Stafford^88,89^, Laura Steinhardt^41^, Vanessa Tran^27,90^, Isidore T Traore^91,92^, Pablo Vial^93^, Tri Yunis M Wahyono^94^, Tyler Williamson^2,81^, Cedric P Yansouni^95,96^, Caseng Zhang^97^, Chong Zhuo Lin^98^ Didier Koumavii^99^

^19^Centro de Epidemiología y Políticas de Salud, Facultad de Medicina Clínica Alemana, Universidad del Desarrollo, Chile

^20^Aden University, Yemen

^21^Facultad de Gobierno, Universidad del Desarrollo, Chile

^22^Nigerian Institute of Medical Research, Nigeria

^23^Infectious Hazard Preparedness, WHO Health Emergencies Programme

^24^Hadhramout University, Al Mukalla, Yemen

^25^Ethiopian Public Health Institute, Addis Ababa, Ethiopia

^26^University of East Sarajevo Faculty of Medicine Foča, Bosnia and Herzegovina (Republic of Srpska)

^27^Public Health Ontario, Toronto, Ontario, Canada

^28^Dalla Lana School of Public Health, University of Toronto, Ontario, Canada

^29^Ministry of Health, Uganda

^30^Kellogg College, University of Oxford, England

^31^Cumming School of Medicine, University of Calgary, Calgary, Alberta, Canada

^32^Department for Blood Transmissible Disease Testing, Institute of Transfusion Medicine of the Federation of Bosnia and Herzegovina, Sarajevo, Bosnia and Herzegovina (Federation)

^33^World Health Organization, Country Office in the Republic of Moldova

^34^Public Health Institute of Malawi, Ministry of Health, Malawi

^35^Faculty of Medicine and Health Sciences, McGill University, Montreal, Quebec, Canada

^36^Department of Microbiology and Infection Prevention Control, School of Biomedicine, Mongolian National University of Medical Sciences, Ulaanbaatar, Mongolia

^37^Center for Respiratory Disease and Meningitis, National Institute for Communicable Diseases, Johanessburg, South Africa

^38^Epidemiology and Quality of Life Research Unit, INSERM U1231, Georges François Leclerc Centre – UNICANCER, Dijon, France

^39^Surveillance Division, Prevention Directorate, Ministry of Health and Social Action, Dakar, Senegal

^40^Kamuzu University of Health Sciences, University in Blantyre, Malawi

^41^U.S. Centers for Disease Control and Prevention, Atlanta, United States of America

^42^Noguchi Memorial Institute for Medical Research, Accra, Ghana

^43^Faculty of Engineering, McGill University, Quebec, Canada

^44^University of Toronto, Toronto, Ontario, Canada

^45^WITS Reproductive Health and HIV Institute, Faculty of Health Sciences, University of the Witwatersrand, South Africa

^46^Virology Department, Institut Pasteur de Dakar, Dakar, Senegal

^47^The International Center for AIDS Care and Treatment Programs (ICAP), Department of Epidemiology, Mailman School of Public Health, Columbia University, New York, United States of America

^48^Department of Epidemiology, Mailman School of Public Health, Columbia University

^49^Department of Disease Control, Ministry of Public Health, Thailand

^50^Instituto de Matemáticas, Universidad de Talca, Chile

^51^School of Medicine and Health Sciences, University of Papua New Guinea

^52^Nigeria Centre for Disease Control, Nigeria

^53^Central Virology Laboratory and Sheba Medical Center, Ministry of Health, Tel-Hashomer, Israel

^54^The Kirby Institute, University of New South Wales, Kensington, Australia

^55^Department of Microbiology, Faculty of Life Sciences, University of Ilorin, Ilorin, Nigeria

^56^Malawi-Liverpool-Wellcome Clinical Research Programme

^57^Liverpool School of Tropical Medicine

^58^Research and Production Center "Preventive Medicine" of the Ministry of Health of the Kyrgyz Republic, Kyrgyzstan

^59^Instituto Leônidas e Maria Deane (ILMD), Fiocruz Amazônia, Manaus, Amazonas, Brazil

^60^Papua New Guinea Institute of Medical Research, Goroka, Papua New Guinea

^61^Harvard Medical School, Boston, Massachusetts

^62^Department of Epidemiology and Biostatistics, Schulich School of Medicine and Dentistry, Western University, Ontario, Canada

^63^National Centre for Immunisation Research and Surveillance, Sydney, Australia

^64^Centre for Women’s Infectious Diseases, The Royal Women’s Hospital, Victoria, Australia

^65^Institut National de Recherche Biomédicale, Kinshasa, Democratic Republic of the Congo

^66^Université de Kinshasa, Democratic Republic of Congo

^67^Agence de Régulation de l'Action Sanitaire et Sociale de Polynésie française

^68^Institut Pasteur of Bangui, Central African Republic

^69^Preventive Health Services, Ministry of Health, Juba, South Sudan

^70^All India Institute of Medical Sciences, New Delhi, India

^71^Department of Epidemiology, Institute for Public Health of the Federation of Bosnia and Herzegovina, Bosnia and Herzegovina

^72^Sarajevo Medical School, University Sarajevo School of Science and Technology, Bosnia and Herzegovina

^73^South African Medical Research Council Vaccines and Infectious Diseases Analytics Research Unit, Faculty of Health Sciences, University of the Witwatersrand, Johannesburg, South Africa

^74^School of Pathology, Faculty of Health Sciences, University of Witwatersrand, South Africa

^75^Palestinian National Ministry of Health, Occupied Palestine Territory

^76^Scientific and Production Association for Preventive Medicine, Ministry of Health of the Kyrgyz Republic, Kyrgyzstan

^77^Washington State University, Global Health Kenya, Nairobi, Kenya

^78^Paul G. Allen School of Global Health, Washington State University, United States of America

^79^Epidemiology and Disease Control Division, Ministry of Health and Population, Nepal

^80^The Palestinian National Institute for Public Health, Ramallah, Occupied Palestine Territory

^81^Department of Community Health Sciences, University of Calgary, Calgary, Alberta, Canada

^82^World Health Organization, Country Office Beirut, Lebanon

^83^Boston Consulting Group

^84^Public Health Department, Facultad de Medicina, Universidad Católica del Norte, Coquimbo, Chile

^85^The International Center for AIDS Care and Treatment Programs (ICAP), Columbia University, New York, United States of America

^86^Department of Population and Family Health, Mailman School of Public Health, New York, United States of America

^87^Institute of Epidemiology, Disease Control and Research (IEDCR), Bangladesh

^88^Center for International Health, Education, and Biosecurity, University of Maryland School of Medicine, Baltimore, United States of America

^89^Division of Epidemiology and Prevention, Institute of Human Virology, University of Maryland School of Medicine

^90^Department of Laboratory Medicine and Pathobiology, University of Toronto, Toronto, Canada

^91^Programme de Recherche sur les maladies infectieuses, Centre MURAZ, Bobo-Dioulasso, Burkina Faso

^92^Institut Supérieur des Sciences de la Santé, Université Nazi Boni, Bobo-Dioulasso, Burkina Faso

^93^Instituto de Ciencias e Innovación en Medicina, Facultad de Medicina Clínica Alemana, Universidad del Desarrollo, Chile

^94^Department of Epidemiology, Faculty of Public Health, University of Indonesia, Depok, Indonesia

^95^J.D. MacLean Centre for Tropical Diseases, McGill University Health Centre, Quebec, Canada

^96^Divisions of Infectious Diseases and Medical Microbiology, McGill University Health Centre, Quebec, Canada

^97^Faculty of Health Sciences, McMaster University, Ontario, Canada

^98^Institute for Public Health, National Institutes of Health, Ministry of Health, Malaysia

^99^ Faculty of Public Health, University of Lomé

‡Deceased
