# Supplementary material for: Global SARS-CoV-2 seroprevalence from January 2020 to April 2022: A systematic review and meta-analysis of standardized population-based studies
Source: PLoS Med. 2022 Nov 10;19(11):e1004107. doi: 10.1371/journal.pmed.1004107 (PMC9648705; doi:10.1371/journal.pmed.1004107)
Supplement: Attachment — Submitted filename: bergen.pdf [file pmed.1004107.s003.pdf]

This paper reports a systematic review with meta–analysis of studies of seroprevalence of SARS–CoV–2 infection.

I was asked for a statistical report and I interpret that to include all aspects of the design and conduct of the study.

Points of detail

Page 4 Does this mean that the same two people did all three tasks (which seems to me undesirable in terms of reducing bias) or were they shared out amongst pairs of researchers? Many papers I read give the initials of the people involved.

Page 5 Why use a multi–stage approach of meta–analysing first and then fitting a model to the summary estimates rather than fitting a one–stage model?

Page 5 The authors have a lot of data so I wonder why smoothing over a three month window was thought necessary.

Page 6 Again I wonder about the reason for using a two–stage approach to the ratios here.

Page 6 What scientific question caused the authors to discard variables? Selecting a subset of variables in a way driven by the data leads to a model which is unlikely to replicate (Babyak, 2004).

Page 7 Does machine readable mean machine translatable? Otherwise it is not clear to me.

Page 7 Excluding more than 1000 studies for not fitting the SEROPREV protocol means much data is being omitted. The article really needs to give us more justification for this. There may be perfectly good reasons but we should know why so much is excluded.

Page 8 The maps are very hard to read. This particularly an issue for the Caribbean. I would be tempted to provide separate maps for suitably smaller regions.

Page 9 '[. . .] the seroprevalence estimated typically would not influence an attempt to publish.' How do the authors know this?

Page 9 Are there really 803 studies or 803 samples?

Page 9 I have some doubts as to whether all of the populations sampled are really population samples. Blood donors are usually atypical whether

altruistic or paid. Pregnant women differ in age from even the generality of women. People living in slums are different in many ways.

Page 11 and 12 Supplement S 4.2 appears to be about risk of bias assessment and not about the smooth fit. I cannot find those details anywhere although with 300+ pages this is not to surprising.

Page 13 Given the methods used elsewhere the switch here to Krukal–Wallis seems surprising. The distributions in Figure S3 look fairly symmetrical so ANOVA for age groups and a mean difference with confidence interval for sex would seem acceptable.

Page 13 Multivariate is used here but multivariable on page 14 (Hidalgo and Goodman, 2013). I suspect multivariable is meant but I suppose a multivariate model is possible. Multivariate means multiple variables on the left hand side.

Page 14 Is τ^2 the estimated variance of the distribution of effects? If so I think a confidence interval would be much preferable to giving a standard error for it given its lack of symmetry.

Supplement Table of Contents Some of these seem to have lost their anchor and other page references are > 10000 for some reason known only to Microsoft. While this is being fixed perhaps the page numbers can be converted to live links so the reader can jump to the required place?

Supplement S2.1 I think much of this is too important to relegate to the supplement. Specifically the details of how sources not in English were dealt with and the inclusion and exclusion criteria are important topics.

Supplement S3.2 This reveals which R packages were used. I think they should be cited in the main paper alongside the R system itself. It is the only credit authors get. (Disclaimer: I am an R package author although not of these packages.)

Supplement Table S 11 Variables left out seem on the face of it to be important ones: study population and age. I think this needs more justification.

Points of more substance

Heterogeneity and intervals

The authors present confidence intervals for their summary estimates. This tells us how precisely the summary estimate has been estimated but in a context of gloal prevalence estimates it would seem desirable to also give us prediction intervals (Riley et al., 2011).

Meta–regression and summary estimates

If the meta–regression shows that one or more moderators was important then presenting an overall summary estimate is not representative of what is happening. It is averaging apples and pears. This occurs throughout starting in the abstract. Either present the coefficient of the regression in the case of a continuous moderator or values for each level of a categorical moderator.

Summary

As my comments indicate I feel the authors have told us what they did but not why. I also have some points about the meta–analysis to consider.

Michael Dewey

References

M A Babyak. What you see may not be what you get: a brief, nontechnical introduction to overfitting in regression–type models. *Psychosomatic Medicine*, 66:411–421, 2004.

B Hidalgo and M Goodman. Multivariate or multivariable regression. *American Journal of Public Health*, 103:39–40, 2013.

R D Riley, J P T Higgins, and J J Deeks. Interpretation of random effects meta–analyses. *British Medical Journal*, 342:964–967, 2011.
