# Supplementary material for: Global SARS-CoV-2 seroprevalence from January 2020 to April 2022: A systematic review and meta-analysis of standardized population-based studies
Source: PLoS Med. 2022 Nov 10;19(11):e1004107. doi: 10.1371/journal.pmed.1004107 (PMC9648705; doi:10.1371/journal.pmed.1004107)
Supplement: Attachment — Submitted filename: 2022-04-28 Global MA responses to reviewers.docx [file pmed.1004107.s004.docx]

**RESPONSES TO REVIEWERS:**

Requests from the editors:

From the academic editor:

1. Seroprevalence studies are sometimes used for other pathogens to gauge progress towards herd immunity. Can the co-authors please elaborate in the text on the utility of serosurveillance for herd immunity for COVID-19 given our knowledge of the virus (and its evolution across different variants)

**Response:** SARS-CoV-2 antibody positivity does not imply protection from subsequent infection, meaning that seroprevalence studies are not an appropriate measure to gauge progress towards herd immunity for SARS-CoV-2. We have also added some text explaining this in our paper in paragraph two of the introduction, page 3, lines 120-122: “*Although seroprevalence studies provide crucial information on population-leve infection dynamics, it is important to note that it does not imply protection against infection and therefore is not an appropriate measure to gauge progress towards herd immunity”*

2. It was good to see WHO recommend serosurveillance in the conclusion. Could the WHO team please provide more detail on what is the public health use of serosurveillance data as part of a national COVID-19 surveillance strategy? Also, what would be the strength of this recommendation, given the delays needed to administer the surveys and the ferocious pace of transmission we've seen in some of these waves that could lead to the survey data being stale by the time they are available to decision-makers? A strong rationale is needed to put the next unit of currency into this rather than improving case surveillance as part of overall laboratory and health system strengthening

**Response:** Thank you for your thoughtful comment. We have added the following passages to our discussion (page 18, lines 598-612) and conclusion to provide more detail on WHO’s recommendations for the use of public health serosurveillance and the strength of this recommendation respectively:

*“Serosurveillance provides key epidemiologic information that crucially supplements other routine data sources in populations. In populations with reported high vaccine coverage, seroprevalence studies provide a supplement to vaccine coverage data and are an important tool for the evaluation of vaccination programs. In populations with low vaccine coverage, its provide an estimate of cumulative incidence of past SARS-CoV-2 infection (including asymptomatic and mild disease), true case fatality ratio, and avoid many of the limitations of passive disease reporting systems which can be unreliable due to under-diagnosis and under-notification. Seroprevalence data can be used to compare seroprevalence between different groups (e.g. age, sex, geography, etc.) to identify susceptible sub-populations and thus inform decisions regarding the implementation of counter measures such as vaccination programs and PHSM .”*

*“.”
[...]
“A key challenge in implementing serosurveillance has been timeliness of study implementation, data analysis and reporting - As such, it will be important for public health decision makers to prioritize investment and establish emergency-mode procedures to facilitate timely study implementation early on in future outbreak or variant emergence responses as part of the overall surveillance strategy. There is also a need to continue to build national capacity with WHO and other partners to enable rapid and high quality study implementation and communication of findings in a format friendly to decision-makers. ”*

Note: These recommendations are based on a WHO operational brief entitled [“Interpreting SARS-CoV-2 seroprevalence studies for public health decision making](https://drive.google.com/file/d/1VjKIPWohEaabXWd_zipg9D1jRlRJIpaQ/view?usp=sharing)” (07 December 2021) which has been shared with regional offices and study teams and findings from another paper authored by members of the research team [1]. WHO COVID-19 surveillance guidance ([here](https://www.who.int/publications/i/item/WHO-2019-nCoV-SurveillanceGuidance-2022.1)) is covering how to best use and interpret seroprevalence for public health decision making and an updated version of this guidance is to be published in June 2022.

**[1]** Duarte N, Yanes-Lane M, Arora RK, Bobrovitz N, Liu M, Bego MG, Yan T, Cao C, Gurry C, Hankins CA, Cheng MP. Adapting serosurveys for the SARS-CoV-2 vaccine era. InOpen forum infectious diseases 2022 Feb (Vol. 9, No. 2, p. ofab632). US: Oxford University Press.

3. Can the authors unpack the implications of different assays being used across studies further (including elaborating on why many would argue against combining studies using different assays). The limitation cited in the discussion is helpful but insufficient.

**Response:** Thank you for mentioning this limitation, we appreciate the thoughtful comment. We have added the following text to the limitations section (page 18, lines 575-580) explaining the potential objection, justifying why we have combined results across assays, and providing a sensitivity analysis showing that when we normalize for assay performance results remain similar:

“*Some argue against combining studies using different assays, because assay performances can vary considerably leading to potential bias in the results. With the moderate seroprevalence values generally observed in our results (roughly 20% to 80%), we expect limited bias to be introduced b the different assays. Nevertheless, we conducted a sensitivity analysis adjusting estimates from individual studies with assay performance whenever available and found that global and regional estimates remained similar.”*

4. The authors mention the underascertainment of cases may explain the difference between seroprevalence rates and estimated infection rates from case surveillance data. This may also be due to some countries using less sensitive definitions than others (see <https://wwwnc.cdc.gov/eid/article/28/1/21-1082_article>)

**Response:** Thank you for your comment, we agree this is an interesting point and we have added it into our discussion of underascertainment along with the suggested citation (page 16, lines 511-514).

5. It was good to see in the covering letter the authors were willing to update the data. July 2021 was long ago (pre Delta wave in many countries and pre Omicron globally). It would be really nice to track the data closer to the end of May 2022 (where Omicron has been able to reach most countries and we would have a more accurate estimate of current global exposure).

**Response:** Thank you for your suggestion. We agree this would be very valuable. We have updated our data to capture studies published up to Friday, May 20 - i.e., within three weeks of re-submission. With this said, these data remain subject to the sometimes-considerable lag between when seroprevalence studies sample participants and publish results.

We recognize the value of data on post-omicron seroprevalence. As of our search on May 20, 2022, there were 14 seroprevalence studies that sampled participants in 2022 and met our inclusion criteria, originating from the Americas: High income, Europe: High income, and Western Pacific. For these regions with sufficient data, we have updated regional estimates of seroprevalence into Q1 2022. We have also used additional data from serosurveys sampling participants in Q3 and Q4 2021 to refine our regional estimates of seroprevalence in these quarters.

To produce representative estimates of global seroprevalence for a given time period, we require seroprevalence studies which sampled participants in a majority of the world’s regions during that time period. In our May 20, 2022 search, we identified seroprevalence studies sampling participants in a majority of regions through September 2021, and have updated our estimates of global seroprevalence to Q3 2021 considering this. In the author summary, we also highlight trends in seroprevalence at the regional level through February 2022 (i.e., Q1 2022), to provide the readers of this manuscript with the most recent estimates of seroprevalence possible with the data currently published (page 3, lines 94-103):

*“We meta-analyzed standardized SARS-CoV-2 seroprevalence studies to estimate the proportion of the global population with antibodies against the virus causing COVID-19. By September 2021, global SARS-CoV-2 seroprevalence from infection or vaccination was 59.2%, 95% CI [56.1-62.2%]. Overall seroprevalence rose steeply in 2021 due to infection in some regions (e.g., 26.6% [24.6-28.8] to 86.7% [84.6-88.5%] in Africa) and vaccination and infection in others (e.g., 9.6% [8.3-11.0%] to 95.9% [92.6-97.8%] in Europe high-income countries). After the emergence of Omicron in February 2022, infection-induced seroprevalence had risen to 47.9% [41.0-54.9%] in EUR HIC and 33.7% [31.6-36.0%] in AMR HIC.”*

Other editorial points:

6. Search: Please update the review to the present time, if possible. Throughout, please clarify the beginning and end dates of the search.

**Response:** We have updated our data to capture studies published within three weeks of re-submission. The new search date is January 1st 2020 to May 20th, 2022. We have added additional mentions of our search dates throughout the paper, in the results and discussion section as well as the methods.

7. Title: Please revise your title according to PLOS Medicine's style. Your title must be nondeclarative and not a question. It should begin with main concept if possible. "Effect of" should be used only if causality can be inferred, i.e., for an RCT. Please place the study design ("A randomized controlled trial," "A retrospective study," "A modelling study," etc.) in the subtitle (ie, after a colon).

**Response:** We have revised the title accordingly (following the directions listed on [PLOS Medicine submission guidelines](https://journals.plos.org/plosmedicine/s/submission-guidelines#:~:text=PLOS%20uses%20%E2%80%9CVancouver%E2%80%9D%20style%2C,in%20the%20ICMJE%20sample%20references.) for a full and short title), as follows:

**Full title:**

Global SARS-CoV-2 seroprevalence: a systematic review and meta-analysis of standardized population-based studies from Jan 2020 - May 2022

**Short title:** Global SARS-CoV-2 seroprevalence from Jan 2020-May 2022

8. Line numbers: Please include line numbers, running continuously throughout the text, with the revised version.

**Response:** We have added in-line numbers throughout.

9. Abstract: Please structure your abstract using the PLOS Medicine headings (Background, Methods and Findings, Conclusions).

**Response:** Suggestion has been incorporated.

10. Abstract: It may be helpful to provide a sentence with some background for the WHO UNITY protocol.

**Response:** We have added an additional line contextualizing the UNITY protocols in the introduction of the abstract (lines 45-48)*: “We meta-analyzed SARS-CoV-2 seroprevalence studies, standardized to those described in WHO’s Unity protocol for general population seroepidemiological investigations”:*

11. Abstract: Methods and Findings: Please specify the inclusion and exclusion criteria for included studies, and the study designs included.

**Response:** We have briefly summarized the inclusion and exclusion criteria in the abstract (lines 53-56). Please note we have also now included the PROSPERO registration in the abstract where more detail can be found:

: *“We included general population cross-sectional and cohort studies meeting an assay quality threshold (90% sensitivity, 97% specificity; exceptions for humanitarian settings). We excluded studies with an unclear or closed population sample frame.”*

12. Abstract: Methods and Findings: Please describe the demographic subgroups of interest, such as age and sex, in this sentence: “...meta-analyzed differences in seroprevalence between demographic subgroups…”

**Response:** Incorporated as suggested into the abstract, see lines 60-61.

13. Abstract: Methods and Findings: Please mention the study registration as: “The review protocol was registered in PROSPERO under registration number CRD42020183634.” or similar.

**Response:** Thank you, we have added this in the abstract, see line 53.

14. Abstract: Methods and Findings: Please describe how risk of bias was assessed.

**Response:** We have added a line clarifying this (lines 56-57): “ [...] *were extracted and critically appraised in duplicate, with Risk of Bias being evaluated using a modified Joanna Briggs Institute checklist.”*

15. Abstract: Methods and Findings: Please report the numbers of participants in the included studies. Please summarize the time periods covered by the included studies.

**Response:** We have added these points to the abstract (line 64).

16. Abstract: Methods and Findings: Please clarify the meaning of “Q2” at first use in the text.

**Response:** We have put both the months and quarter in the abstract (line 71) and throughout the text.

17. Abstract: Methods and Findings: Please present results for the main outcomes, with summary estimates, 95% CIs and p values where applicable. If possible, please mention the number of included studies and participants for each result.

**Response:** We have added 95% CIs and p-values to the abstract where applicable. We have also added the number of included studies and participants overall, and provided further detail on each dataset in the main text.

18. Abstract: Methods and Findings: In the last sentence of the Abstract Methods and Findings section, please describe the main limitation(s) of the study's methodology.

**Response:** We have added a note on our limitations as the final sentence of this section in the abstract as suggested (lines 61-62) as follows: “*The main limitations of our methodology include that some estimates were driven by certain countries or populations being over-represented*”

19. Abstract: Conclusions: Please address the study implications without overreaching what can be concluded from the data; the phrase "In this study, we observed ..." may be useful.

**Response:** Thank you for your comment, we have incorporated the wording you suggested at line 78.

20. Author summary: At this stage, we ask that you include a short, non-technical Author Summary of your research to make findings accessible to a wide audience that includes both scientists and non-scientists. The Author Summary should immediately follow the Abstract in your revised manuscript. This text is subject to editorial change and should be distinct from the scientific abstract. Please see our author guidelines for more information: <https://journals.plos.org/plosmedicine/s/revising-your-manuscript#loc-author-summary>

**Response:** Thank you, we have now added an author summary section at page 2-3, lines 84-109 and welcome further editorial suggestions to this paragraph.

21. Main text: Please use square brackets for in-text citations, placed before the sentence punctuation. Where more than one reference is indicated, please do not include spaces, for example [1].

**Response:** Revised throughout.

22. Methods: “We conducted a systematic review of seroprevalence studies (hereafter “studies”) published from 1 January 2020 to 29 October 2021.” Please clarify the dates of the search. In the abstract, and end date of December 30, 2021 is mentioned.

**Response:** Thank you for noting this error in our text. We have updated the search dates with our updated search to May 20 2022 here (page 4, lines 156-157) and elsewhere.

23. Methods: Thank you for the statement regarding the MOOSE guidelines. Please also report your SRMA according to the PRISMA guidelines provided at the EQUATOR site.

<http://www.equator-network.org/reporting-guidelines/prisma/>

**Response:** Added to supplement.

Please provide the completed PRISMA and MOOSE checklists. When completing the checklist, please use section and paragraph numbers, rather than page numbers.

**Response:** Updated with section and line numbers for ease of review.

Please add the following statement, or similar, to the Methods: "This study is reported as per the Preferred Reporting Items for Systematic Reviews and Meta-Analyses (PRISMA) guideline (S1 Checklist)."

**Response:** We have incorporated your suggestion with wording as above to the methods section at page 4-5, lines 170-172.

24. Methods: Please mention any language restrictions for included studies.

**Response:** We moved a description of our language restrictions from our Supplementary Methods to the main text (page 5, lines 175-177) of the article to address your comment, as follows:

*“We have study team members proficient in English, French, Portuguese, Spanish, and Cyrillic languages - articles in all other languages were translated using Google Translate where possible.”*

25. Methods: Please describe any consideration of publication bias.

**Response:** Thank you for your comment, we have added the following clarification to our method section, first paragraph (page 4, lines 159-161): “*We attempted to mitigate possible publication bias by including both published articles and unpublished literature such as grey literature, preprints, institutional reports, and media reports (full strategy in Supplementary file S.3.1)..”*

26. Methods: Please explicitly mention whether the protocol and analysis plan were established prior to the conduct of the review. Please include the relevant prospectively written document with your revised manuscript as a Supporting Information file to be published alongside your study, and cite it in the Methods section. A legend for this file should be included at the end of your manuscript. Please identify any changes from the protocol in the Methods.

**Response:** The protocol was established prior to the conduct of the review - it is registered in PROSPERO and we have added explicit mention of this in the first paragraph of the Methods (page 4, lines 168-170).

*“This systematic review and meta-analysis protocol was registered with PROSPERO (CRD42020183634) prior to the conduct of the review (Supplement S6), reported according to the Preferred Reporting Items Systematic review and Meta-Analyses (PRISMA) guideline (Supplementary file S1 Checklists), and search and extraction conducted per the previously established SeroTracker protocol* .” We have also included the prospectively written protocol in the Supplement S6.

27. Methods: Please mention any methods used to explore possible contributions to high heterogeneity observed in the results.

**Response:** We used subgroup analysis and meta-regression to explore possible causes of heterogeneity among study results. We have clarified this in the methods (page 7, lines 266-267): “*To explore possible causes of heterogeneity among study results, we constructed a Poisson generalized linear mixed-effects model with log link function using the glmer function from the lme4 package in R”.*

28. Results: Bottom of page 7: When reporting percentages of studies in descriptive analyses conducted in LMIC/vulnerable HRP, please provide numerators and denominators. Please similarly address the reporting of percentages throughout.

**Response:** We have incorporated reporting of percentages / numerators and denominators where missing throughout our paper.

29. Results: For the reporting of comparisons of seroprevalence between age groups and by sex, please report p values. Please also note on page 13 that these results are reported in figure 4, rather than figure 3.

**Response:** We have added the p values. Thank you for noting the figure number error, we have corrected this in the text.

30. Discussion: Please present and organize the Discussion as follows: a short, clear summary of the article's findings; what the study adds to existing research and where and why the results may differ from previous research; strengths and limitations of the study; implications and next steps for research, clinical practice, and/or public policy; one-paragraph conclusion.

**Response:** Thank you for your comment. We have re-organized the discussion to match this request, and added the following sub-headings for clarity: *Summary; Research in context; Strengths and limitations; Implications and next steps; Conclusion.*

31. References: Please use the "Vancouver" style for reference formatting, and see our website for other reference guidelines <https://journals.plos.org/plosmedicine/s/submission-guidelines#loc-references>

Please check the formatting of each reference, including journal title abbreviations. For example PLOS ONE should be PLoS One. Please remove [Internet] throughout.

**Response:** Thank you for your comment - we have formatted our references according to your guidance throughout.

32. Figure and Table legend: In the legends, please fully describe all abbreviations used within each figure and table.

**Response:** We have added descriptions of abbreviations in each figure and table.

33. Figure 2: Please confirm that the appropriate usage rights apply to the use of this map, or please illustrate the included member states using a different map or strategy. PLOS applies the Creative Commons Attribution (CC BY) license to all the works we publish (CC BY 4.0). The maps must be free of logos, copyright symbols / “All rights reserved” text. Please see our guidelines for map images: <https://journals.plos.org/plosmedicine/s/figures#loc-maps>

**Response:** We confirm that the data belongs in the public domain. All logos, copyright symbols, and “all rights reserved” text have been removed.

34. Figure 3: Please include p values.

**Response:** We assume this refers to Figure 4 reporting of comparisons of seroprevalence between age groups and by sex as mentioned above. We have added p values to the figure.

35. PRISMA Checklist: Please refer to section and paragraph numbers when completing the checklist (for example: Methods, paragraph 1). Please include the MOOSE Checklist, if it is your intention to do so.

**Response:** Section and paragraph numbers have been incorporated. We intended to include only the PRISMA checklist and have updated the text accordingly.

36. S.2.1 Supplementary Methods: As mentioned by reviewer 2, we suggest moving key details of the search strategy and inclusion and exclusion criteria to the main text, especially a clear description of language restrictions and the included sample populations. It would also be helpful to note if the proportions of datasets not yet published/publicly available in the included studies are reported in the results.

**Response:** Thank you for your comment. At your suggestion, we have moved the additional details on our methods (Search strategy and inclusion/exclusion criteria) to the main text (page 5, lines 181-190).

*“We included cross-sectional or longitudinal cohort studies with the objective of estimating SARS-CoV-2 seroprevalence in the general population. Restricting inclusion to direct population samples such as household surveys would have led to very little data in some regions and times, as these studies are expensive and difficult to conduct. Thus, household and community samples were included, as well as studies where a robust sampling frame was described that approximates to a wider population, such as individuals attending medical services (blood donors, pregnant mothers, primary care attendees) or residual sera taken from patients for a variety of other investigations. Finally, we also included people residing in slum dwellings, and some patient populations in humanitarian settings where the patient population in question was extensive enough to be considered a proxy sample frame (evaluated on a case-by-case basis)”*

Table S1 and supplement S.2.2 on the full Unity criteria document have been left in the Supplement as additional reading. We already have a line of text in the first paragraph of results on the number of included studies that were from early results templates submitted to us and have added the proportion, see the following passage (page 8, lines 330-333):

*“513 seroprevalence data sources containing studies aligned with the SEROPREV protocol were identified, 480 published (94%) and 33 aggregated results from collaborators (6%).*”

37. S.2.4: Please clarify the search dates throughout (here October 29, 2021 is mentioned as the end date).

**Response:** Thank you for noting this error in our text. We have updated the search dates with our updated search into May 2022 here and elsewhere throughout the manuscript.

Comments from the reviewers:

Reviewer #1: Important contribution to our knowledge of the pandemic.

**Response:** Thank you very much for your positive comment on our manuscript.

Reviewer #2: See attachment COPIED BELOW

This paper reports a systematic review with meta–analysis of studies of sero- prevalence of SARS–CoV–2 infection.

I was asked for a statistical report and I interpret that to include all aspects of the design and conduct of the study.

Points of detail

Page 4 Does this mean that the same two people did all three tasks (which seems to me undesirable in terms of reducing bias) or were they shared out amongst pairs of researchers? Many papers I read give the initials of the people involved.

**Response:** Thank you for your comment. We have also amended this sentence (page 5, lines 174-175) to read:” “*Studies were screened, data extracted, and critically appraised in duplicate, with these tasks shared within a team of 15 study authors (listed in Research Contributions section under data curation)”*

Page 5 Why use a multi–stage approach of meta–analysing first and then fitting a model to the summary estimates rather than fitting a one– stage model?

**Response:** We have rephrased paragraph 7-8 in the Methods (page 7, lines 277-293) to better explain the rationale for our approach, excerpted below:

*“We first produced monthly country-level estimates by meta-analyzing seroprevalence in each country, grouping studies in a 12-week rolling window considering the infrequent availability of seroprevalence studies in most countries (rma.glmm from R package metafor). We then produced monthly regional estimates by taking weighted averages of country estimates by population, ensuring that country contributions to these estimates are proportional to country population. We stratified these estimates by the expected key sources of heterogeneity among study results: region, income class, and time.”*

*“We produced monthly global estimates where estimates were available for a majority of regions, calculating global estimates as a population-weighted average of regional estimates to ensure regional representation. We produced 95% confidence intervals for the mean seroprevalence estimate, reflecting uncertainty in the summary effect size, and 95% prediction intervals to give a range for the predicted parameter value in a new study. All numerical results presented are from this stage. To visualize the trend in regional and global estimates over time, we fit a smooth curve to these estimates using nonparametric regression (gam from R package mgcv).”*

Page 5 The authors have a lot of data so I wonder why smoothing over a three month window was thought necessary.

**Response:** While we identified many studies, these are distributed across many countries and time points, meaning that very few countries have seroprevalence data for each month. Moreover, some regions have sparser data overall (e.g. Eastern Mediterranean, Western Pacific). Using a window allows us to produce regular estimates of seroprevalence over time by WHO region, without large gaps of several months. We have elaborated on the rationale for this in the manuscript in the Methods (page 7, lines 277-280): “[...] *grouping studies in a 12-week rolling window considering the infrequent availability of seroprevalence studies in most countries.”*

Page 6 Again I wonder about the reason for using a two–stage approach to the ratios here.

**Response:** We have described in a previous response our reasons for using a two-stage approach to produce regional estimates of seroprevalence. Under-ascertainment ratios were based on the same rationale (page 8, lines 302-305):

*“We then produced regional and global estimates of seroprevalence using the two-stage process described above, and computed the ratio to the corresponding cumulative incidence of confirmed SARS-CoV-2 cases in the region or globally.”*

Page 6 What scientific question caused the authors to discard variables? Selecting a subset of variables in a way driven by the data leads to a model which is unlikely to replicate (Babyak, 2004).

**Response:** Thank you for this question. We initially adopted the lowest AIC approach to select the best model among all competing models (Burnham and Anderson, 2002; Burnham and Anderson, 2004). We initially adopted this approach due to concerns about overfitting. However, we recognize the reviewer’s point that data-driven variable selection presents a greater risk of overfitting, and have modified our approach as outlined in the Methods (page 7, lines 268-270): *“Independent predictors were defined a priori as WHO region, income group, geographic scope, sample frame, pandemic timing, age, cumulative confirmed cases, and average public health and social measure (PHSM) stringency index. [...] We included all a priori predictors in the final model, and to evaluate the importance of each relevant predictor, we compared the Akaike Information Criterion (AIC) of the final model to all models dropping a single predictor at a time.”*

Page 7 Does machine readable mean machine translatable? Otherwise it is not clear to me.

**Response:** Yes, we have added text in the Methods explaining, “*We have study team members proficient in English, French, Portuguese, Spanish, and Cyrillic languages - articles in all other languages were translated using Google Translate where possible.”* (page 5, lines 175-177)

Page 7 Excluding more than 1000 studies for not fitting the SEROPREV protocol means much data is being omitted. The article really needs to give us more justification for this. There may be perfectly good reasons but we should know why so much is excluded.

**Response:** Thank you for your comment - we agree that a more detailed rationale would be helpful. We have added a few lines describing the rationale for including studies in order to align with the SEROPREV protocol (to control quality criteria such as assay performance and sampling methods; to minimize potential bias introduced by inter-study heterogeneity) in the methods section, and an explanation on the main reasons why studies were excluded for not being SEROPREV-aligned in the first paragraph of the results (page 8, lines 334-340):

*“Over 1,500 full-text articles were excluded due to not containing studies compatible with the SEROPREV protocol; the main reasons for articles’ exclusion at this stage was being an incorrect sample frame for this analyses’ scope (i.e., we focussed on seroprevalence in the general population and therefore excluded 1,073 articles of exclusively health care workers, close contacts of confirmed cases, or other specific closed populations) or not meeting our pre-defined assay quality performance threshold (374 articles).”*

Page 8 The maps are very hard to read. This particularly an issue for the Caribbean. I would be tempted to provide separate maps for suitably smaller regions.

**Response:** We have increased the resolution of the maps.

Page 9 ‘[. . . ] the seroprevalence estimated typically would not influence an attempt to publish.’ How do the authors know this?

**Response:** Thank you. We have added this consideration to our methods section, first paragraph (page 4, lines 159-161): “*We attempted to mitigate possible publication bias by including both published articles and unpublished literature such as grey literature, preprints, institutional reports, and media reports (full strategy in Supplementary File S.3.1)*”

We also removed the sentence from our results as we cannot confirm this hypothesis with the evidence we have produced in the scope of this analysis.

Page 9 Are there really 803 studies or 803 samples?

**Response:** Thank you for noting this. We were asked by another reviewer to clarify the difference between ‘articles’ and ‘studies,’ and we have added clarification on the definition of ‘studies’ in the fourth paragraph of the Methods (page 6, lines 248-250):

*“Where an article or source material contains multiple, methodologically distinct serosurveys we split the article into multiple ‘studies’ - for the purpose of this review ‘study’ means a distinct estimate.”*

Page 9 I have some doubts as to whether all of the populations sampled are really population samples. Blood donors are usually atypical whether altruistic or paid. Pregnant women differ in age from even the gener- ality of women. People living in slums are different in many ways.

**Response:** Thank you for the comment. We have clarified the rationale for this approach in the second paragraph of the Methods (page 5, lines 182-192):

*“Restricting inclusion to direct population samples such as household surveys would have led to very little data in some regions and times, as these studies are expensive and difficult to conduct. Thus, household and community samples were included, as well as studies where a robust sampling frame was described that approximates to a wider population, such as individuals attending medical services (blood donors, pregnant mothers, primary care attendees) or residual sera taken from patients for a variety of other investigations. Finally, we also included people residing in slum dwellings, and some patient populations in humanitarian settings where the patient population in question was extensive enough to be considered a proxy sample frame (evaluated on a case-by-case basis). Both random or non-random (i.e., convenience, sequential, quota) sampling methods were included. Convenience samples must have a clear and defined sampling frame, i.e., studies recruiting volunteers were not included.”*

Potential differences between populations and their implications were explored in the meta-regression analysis in the Results (Figure 2) and the fifth paragraph of the Discussion. Results suggest that blood donors, residual sera, and pregnant women can be reasonable proxies for the general population, as our model found no statistical difference between their seroprevalence estimates and those in households and communities.

Page 11 and 12 Supplement S 4.2 appears to be about risk of bias assess- ment and not about the smooth fit. I cannot find those details anywhere although with 300+ pages this is not to surprising.

**Response:** Thank you for noting this error. We have corrected the reference to cite Supplement S3.2.

Page 13 Given the methods used elsewhere the switch here to Krukal–Wallis seems surprising. The distributions in Figure S3 look fairly symmet- rical so ANOVA for age groups and a mean difference with confidence interval for sex would seem acceptable.

**Response:** Thank you for this suggestion. We agree and have updated our methods to ANOVA and mean differences with confidence intervals for age and sex groups (page 8, lines 307-309).

Page 13 Multivariate is used here but multivariable on page 14 (Hidalgo and Goodman, 2013). I suspect multivariable is meant but I suppose a multivariate model is possible. Multivariate means multiple variables on the left hand side.

**Response:** Yes, we have corrected it to multivariable; thank you for catching the error.

Page 14 Is τ2 the estimated variance of the distribution of effects? If so I think a confidence interval would be much preferable to giving a standard error for it given its lack of symmetry.

**Response:** Thank you for the suggestion. While the software outputs the standard error of τ^2^ by default, we agree that a confidence interval would be more useful given the asymmetry. We have computed the confidence interval using profile likelihood (confint() in R) and added this to the caption of Figure 4.

Supplement Table of Contents Some of these seem to have lost their anchor and other page references are > 10000 for some reason known only to Microsoft. While this is being fixed perhaps the page numbers can be converted to live links so the reader can jump to the required place?

**Response:** Thank you for this suggestion. We have fixed page references. The headings in the Table of Contents are live links that the reader can click to jump to the required place.

Supplement S2.1 I think much of this is too important to relegate to the supplement. Specifically the details of how sources not in English were dealt with and the inclusion and exclusion criteria are important topics.

**Response:** Thank you for your comment - at your suggestion and other reviewer’s suggestion, we have moved almost all the text from supplementary methods section 2.1 into the main text (on search strategy, inclusion and exclusion criteria, and other source evaluation methods).

Supplement S3.2 This reveals which R packages were used. I think they should be cited in the main paper alongside the R system itself. It is the only credit authors get. (Disclaimer: I am an R package author although not of these packages.)

**Response:** Thank you for this suggestion. We have a cite to the R system in the main paper on page 8, and have also now moved citations to specific R packages from the supplement into the main text.

Supplement Table S 11 Variables left out seem on the face of it to be important ones: study population and age. I think this needs more justification.

**Response:** Thank you for noting these. Age was in fact included in the model but omitted from Table S11 in error. The full model had the lowest AIC value relative to all models dropping a single predictor, so by our original method for model selection, we agree that study population should not have been left out and we have corrected this in our revision. We have also modified our approach to model selection (see response to a previous comment), using the AIC criterion not to discard variables but rather to evaluate the importance of each relevant predictor.

Points of more substance Heterogeneity and intervals

The authors present confidence intervals for their summary estimates. This tells us how precisely the summary estimate has been estimated but in a context of gloal prevalence estimates it would seem desirable to also give us prediction intervals (Riley et al., 2011).

**Response:** Thank you for the suggestion. We agree prediction intervals would be useful in this context to give a range for the predicted parameter value in a new study. We have incorporated both confidence and prediction intervals into the global estimates in Supplement Table S10 and the main text. We have also incorporated the suggested reference.

Meta–regression and summary estimates

If the meta–regression shows that one or more moderators was important then presenting an overall summary estimate is not representative of what is happening. It is averaging apples and pears. This occurs throughout starting in the abstract. Either present the coefficient of the regression in the case of a continuous moderator or values for each level of a categorical moderator.

**Response:** Thank you for the suggestion. The point about moderators is important, and is why we stratified the overall summary estimates by WHO region, income class, and time – which were the most important moderators identified in the meta-regression. Within each stratum, study results are less heterogeneous, and thus it does seem each summary estimate is representative of its region-income-month group.

We have restructured our manuscript to make this rationale clearer – starting the Results section with the meta–regression to identify moderators that explain heterogeneity among study results, and following this with the summary estimates stratified by the key moderators.

Summary

As my comments indicate I feel the authors have told us what they did but not why. I also have some points about the meta–analysis to consider.

Michael Dewey

References

M A Babyak. What you see may not be what you get: a brief, nontechni- cal introduction to overfitting in regression–type models. Psychosomatic Medicine, 66:411–421, 2004.

B Hidalgo and M Goodman. Multivariate or multivariable regression. Amer- ican Journal of Public Health, 103:39–40, 2013.

R D Riley, J P T Higgins, and J J Deeks. Interpretation of random effects meta–analyses. British Medical Journal, 342:964–967, 2011.

Reviewer #3: The authors present an important manuscript which presents global and national estimates of COVID-19 seroprevalence through a systematic review of existing literature and meta-analysis of seroprevalence data. My major comments are primarily focused on the inclusion criteria used for the meta-analysis.

**Response:** Thank you very much for your review of this manuscript. We have addressed your comments in the following responses.

1. Studies in the meta-analysis include those with non-random sampling methods (convenience, sequential, quota) and/or conducted among study populations that are not necessarily representative of subnational or national populations (i.e. blood donors, pregnant or parturient women, persons living in slums). Unfortunately, they make up a substantial proportion of all studies included in the meta-analysis (Table 1). While it's difficult to assess how much of the data from these studies influence sub-national and national seroprevalence estimates, there is a clear concern for bias. For example: convenience sample - Brooks-Sa 2020. JID. Estimation Without Representation: Early Severe Acute Respiratory Syndrome Coronavirus 2 Seroprevalence Studies and the Path Forward. Please provide adequate details so readers can understand how studies where the study population does not reflect the population of interest and studies with non-random sampling methods contribute to representative estimates.

**Response:** Thank you for your comment. First, on sampling methods, our inclusion criteria encompassed studies that conducted random or high-quality non-random sampling. As we have clarified in the methods (page 5, lines 190-192):

*“Both random or non-random (i.e., convenience, sequential, quota) sampling methods were included. Convenience samples must have a clear and defined sampling frame, i.e., studies recruiting volunteers were not included.”*

While convenience samples of the general population were included in the review, we did assess their representativeness as part of the risk of bias evaluation and excluded high risk of bias studies from the meta-analysis, meaning they did not influence our global and regional seroprevalence estimates. For example, although the Santa Clara study [1] cited in Brooks-Sa 2020 was included in our review, it was rated high risk of bias and thus excluded from our meta-analysis estimates. We have added text on the methodological quality of the studies and implications to the discussion section (page 17, lines 554-564):

*“The laboratory and epidemiological standardization enabled by the SEROPREV protocol, as well as the analysis of only studies assessed to have low or moderate risk of bias using a validated risk of bias tool [(20]), enabled high-quality and comparable data. Despite this effort, there are still methodological differences between the meta-analyzed studies that may reduce their comparability. For example, 14% of studies in our analysis dataset (66/459, 18 of which were household-based) were convenience samples, which are less representative than population-based probability samples. To limit this bias, we required UNITY-aligned convenience samples to have a clearly defined sample frame (i.e., sampling of volunteers excluded). Our risk of bias evaluation also included subjective review of the demographic breakdown in the study, coverage of subgroup estimates, and author comments on representativeness of the sample, such that the most non-representative studies were rated high risk of bias and excluded from analysis.”*

Second, on study populations, further description of this in text as below (page 5, lines 182-192):

*“Restricting inclusion to direct population samples such as household surveys would have led to very little data in some regions and times, as these studies are expensive and difficult to conduct. Thus, household and community samples were included, as well as studies where a robust sampling frame was described that approximates to a wider population, such as individuals attending medical services (blood donors, pregnant mothers, primary care attendees) or residual sera taken from patients for a variety of other investigations. Finally, we also included people residing in slum dwellings, and some patient populations in humanitarian settings where the patient population in question was extensive enough to be considered a proxy sample frame (evaluated on a case-by-case basis)”*

Potential differences between populations and their implications were explored in the meta-regression analysis in the Results (Figure 2) and the fifth paragraph of the Discussion (page 17, lines 542-545). Results suggest that blood donors, residual sera, and pregnant women can be reasonable proxies for the general population, as our model found no statistical difference between their seroprevalence estimates and those in households and communities.

[1] Eran Bendavid, Bianca Mulaney, Neeraj Sood, Soleil Shah, Rebecca Bromley-Dulfano, Cara Lai, Zoe Weissberg, Rodrigo Saavedra-Walker, Jim Tedrow, Andrew Bogan, Thomas Kupiec, Daniel Eichner, Ribhav Gupta, John P A Ioannidis, Jay Bhattacharya, COVID-19 antibody seroprevalence in Santa Clara County, California, *International Journal of Epidemiology*, Volume 50, Issue 2, April 2021, Pages 410–419, <https://doi.org/10.1093/ije/dyab010>

2. The authors state, "Studies had to use serological assays with at least 90% sensitivity and 97% specificity as reported by the manufacturer or study authors (Supplementary file S2.1), unless conducted in vulnerable countries as defined in the Global Humanitarian Response Plan" and "Data from…33 of 63 vulnerable HRP countries were included." Figure 2 suggests estimates from South America, Africa, and Middle east are primarily consider vulnerable. Without additional information, it's very difficult to assess how this large exception to the inclusion criterion might influence the estimates reported at the national and ultimately at the global level. Were any additional steps taken to account for differences in test accuracy among vulnerable countries?

**Response:** Thank you for your comment. The list of countries and definition we used is cited in the text and below.

We have added text justifying this exception criteria as follows: “*We employed an exception criteria for HRP countries where rapid diagnostic tests were the only operationally feasible option due to ongoing humanitarian emergencies, despite performance for some of these not being independently assessed..”*

Ultimately, 66 studies included in our meta-analysis are from HRP countries (Table 1). We have added a line of text in the results (page 9, lines 360-361) clarifying how many of these studies exactly were included due to exception criteria: *“20 of the 66 (30%) meta-analyzed studies in HRP countries had test performance values below 90% sensitivity or 97% specificity and were included due to exception criteria.”*

[17.	COVID-19 Global Humanitarian Response Plan [Internet]. United Nations Office for the Coordination of Humanitarian Affairs; 2020 Mar [cited 2021 Nov 4]. Available from: https://www.humanitarianresponse.info/en/programme-cycle/space/document/covid-19-global-humanitarian-response-plan](https://www.zotero.org/google-docs/?broken=tNV5Nr)

3. Do the authors have any thoughts on why sub-national studies have higher estimates than national studies (Table S11)?

**Response:** Thank you for your comment. We have added the following thoughts to our discussion (page 17, lines 540-543): “*our model also found that sub-national studies have higher estimates than national studies; one hypothesis for this is that sub-national studies are often concentrated in cities or areas with dense populations, which may contribute to increased transmission of the virus. Further research is needed to validate this hypothesis.”*

4. On page 4, the authors state "We also contacted WHO UNITY study collaborators that had not yet made results available to the general public prior to our inclusion dates, to upload their aggregated, standardized results to the Zenodo research data repository". It's unclear whether the analysis includes data external to the systematic literature review.

**Response:** Early results submitted to us via the UNITY initiative were captured as part of our secondary search and article capture strategy, rather than our primary search. We have moved our further clarification and explanation of the role and inclusion methods for UNITY studies from the supplement into the main text (page 4, lines 161-166) to provide more context for the reader. We have also added the number of sources captured via the UNITY initiative to the Figure 1 (PRISMA).

*“For our secondary search and article capture strategy, [...]In order to access timely evidence and mitigate challenges of publication delay, we also contacted WHO Unity study collaborators that had not yet made results available to the general public prior to our inclusion dates, to upload their aggregate results to the open access Zenodo research data repository.. We accepted these templates up to May 20th 2022 in line with our primary search strategy, and screened them according to the same criteria as other sources captured in our primary search. ”*

5. The seroprevalence to cumulative incidence ratio is a nice ecological approach for exploring case ascertainment. Please clarify how the 9-day criterion used "to account for time from infection to seroconversion" was derived and whether this adjustment was necessary given how much heterogeneity likely exists in sampling durations and mid-points.

**Response:** We have added references [1],[2] in the supplement to support the 9-day lag time used to account for time from infection to seroconversion. We agree with the reviewer that there is considerable heterogeneity in sampling durations; considering that the median sampling duration of studies in dataset 2 is 27 days, we use this criterion to help ensure alignment between seroprevalence study sampling and case dates.

[1] Wiersinga WJ, Rhodes A, Cheng AC, Peacock SJ, Prescott HC. Pathophysiology, Transmission, Diagnosis, and Treatment of Coronavirus Disease 2019 (COVID-19): A Review. JAMA. 2020 Aug 25;324(8):782–93. pmid:32648899

View ArticlePubMed/NCBIGoogle Scholar

[2] Wilson N, Kvalsvig A, Barnard L, Baker M. Case-Fatality Risk Estimates for COVID-19 Calculated by Using a Lag Time for Fatality. Emerg Infect Dis. 2020;26(6):1339–441. pmid:32168463

6. There some conflicting information provided regarding time of studies. The methods state studies were published between Jan 2020 and Oct 2021. The abstract and discussion state Jan 2020 to Dec 2021. Please correct or clarify.

**Response:** Thank you for noting this error. We have corrected these errors in search dates to our updated search - now Jan 01 2020 to May 20 2022.

Minor comments:

1. Clarify "local" vs. "sub-national"

**Response:** Thank you. “Local” in this case means at the city or county level, whereas “sub-national” refers to province or state level studies. We have clarified this in the first instance of mention of local/sub-national classifications on page 6 (lines 247-249).

2. Clarify "articles" vs. "studies" in the main text. This is only mentioned in Figure 1 description.

**Response:** We added clarification in the fourth paragraph of the Methods (page 6, lines 249-251):

*“Where an article or source material contained multiple, methodologically distinct serosurveys we split the article into multiple ‘studies’ - for the purpose of this review ‘study’ means a distinct estimate.”*

3. "To focus on factors associated with seroprevalence from infection, we excluded studies where over 5% of the national population was vaccinated two weeks before the sampling midpoint date." Please state how many studies remained after exclusion.

**Response:** We have added a sentence in the Results (page 12, lines 384-385) stating how many studies remained after exclusion (N=329).

4. S2.1 indicates articles in all other languages were translated using Google Translate where possible. While Google translate is fine for day-to-day translations, using it for a scientific article is uncommon, especially given technical language. How many articles were not in main languages and translated via Google?

**Response:** We have added a line in the Results (page 8, lines 333-334) indicating that 12/513 (2.3%) articles were not in main languages and translated via Google Translate.

5. Explicitly state where VOC data in Figure 3 comes from in the methods

**Response:** Incorporated (page 7, lines 294-295).

6. Not requested for this manuscript but it would be interesting to see prevalence ratios within smaller age groups among children 0-9 years. It may provide more insight to understanding lower prevalence in young children.

**Response:** Thank you for your comment - we agree, and will consider developing a separate analysis on children 0-9 years old to address this research gap for a later manuscript.

7. On page 14, the authors state "For example, our global estimate of seroprevalence attributable to infection (3.2%) is similar to the Institute of Health Metrics and Evaluation cumulative infection incidence estimate of 37.4% on 31 July 2021." Is this a typo?

**Response:** Thank you for noting this typo. We have amended the sentence (page 12, lines 411-412)as follows:

*“[...] global seroprevalence attributable to infection was 35.9% [CI 29.5-42.7%, prediction interval 22.8-51.4%].”*

8. Although the main purpose of figure 2 is clear, most of the text is not legible due to low resolution.

**Response:** We have increased the resolution of the text.

9. In the supplementary table of contents, please remove the superscript on S1 and correct "Error! Bookmark not defined"

**Response:** Thank you for noting the error. The bookmarks in the table of contents are working in the revised version.

10. Fix the incomplete sentence in the first paragraph of page 13.

**Response:** Incorporated.

11. "Appendix 6" is referenced on page 14. Please correct.

**Response:** Corrected.

12. Please include line numbers in future submissions. These are quite helpful for referencing specific items.

**Response:** We have added line numbers to our re-submission.

Reviewer #4: The authors conducted a systematic review and meta-analysis of SARS-CoV-2 seroprevalence studies after two years in the pandemic to estimate the extent of population infection and remaining susceptibility. They meta-analyzed seroprevalence by country and month, pooling to estimate regional and global seroprevalence over time; estimated the difference between seroprevalence versus infection identified through case reporting. These estimates were also conducted between demographic subgroups and national factors associated with seroprevalence.

This study is a huge undertaking and the authors have been very thorough in designing the study and executing the tasks. The manuscript is well written with clearly stated objectives, well described study design and methods, and the results are clearly displayed. The conclusions are consistent with the findings of the study. Although the authors have accounted for possible limitations of the study, there are some additional ones that the authors may consider:

**Response:** Thank you very much for your thoughtful review of our manuscript. We have addressed your additional comments below.

1.	Methods and Discussion: The majority of the seroprevalence studies are community-based. Did the authors investigate the sampling mechanism used in these community-based studies? If the sampling was carried out during sports, music or other mass gathering events, the results may not be as representative as studies in which a random sampling of a community population was carried out. The implications of sampling mechanisms in community-based studies should be discussed

**Response:** Thank you for your comment. Our inclusion criteria encompassed studies that conducted random or high-quality non-random sampling. As we have clarified in the methods (page 5, lines 190-192):

“*Both random or non-random (i.e., convenience, sequential, quota) sampling methods were included. Convenience samples must have a clear and defined sampling frame, i.e.,studies recruiting volunteers were not included.*” We added further description of this into the main text (was originally in the supplement), as well as a breakdown of which sampling methods were used in the household and community studies. We also added sentences (page 17, lines 557-565) on relevant implications to the discussion: “*Despite this effort, there are still methodological differences between the meta-analyzed studies that may reduce their comparability. For example, 15% of studies in our analysis dataset (68/461, 20 of which were household-based) were convenience samples, which are less representative than population-based probability samples. To limit this bias, we required UNITY-aligned convenience samples to have a clearly defined sample frame (i.e., sampling of volunteers excluded). Our risk of bias evaluation also included subjective review of the demographic breakdown in the study, coverage of subgroup estimates, and author comments on representativeness of the sample, such that the most non-representative studies were rated high risk of bias and excluded from analysis.*”

2.	Discussion, P.15 3rd paragraph: The high seroprevalence versus incidence rates in LMICs, especially in Africa: The authors should not take the high rates of seroprevalence for COVID-19 from studies at face value. A number of peer-reviewed publications have shown that false positive results in serological assays may be due to malaria. (e.g. Steinhardt LC, et al. Cross-reactivity of two SARSCoV-2 serological assays in a setting where malaria is endemic. J Clin Microbiol 2021; 59:e00514- 21. <https://doi.org/10.1128/JCM.00514-21>). The authors should discuss the implications of their findings in light of these publications.

**Response:** Thank you for your comment - we agree that this is an interesting discussion point that may contribute to high seroprevalence estimates in Africa. We have added the following passage to our discussion (page 16, lines 503-505) with supporting evidence including the reference above:

*“Another possibility for regional variation is the potential cross-reactivity of antibodies against P. falciparum or other common cold coronaviruses which has been remarked upon in the literature, which may impact seroprevalence estimates in areas of Africa or elsewhere with a high incidence of malaria.*”

3.	Discussion, P.16 2st paragraph: The authors stated, "In cases where antibodies can be measured quantitatively, such as for SARS-CoV-2, they can also provide correlates with protection against infection."	Since a global consensus on the exact targets and antibody concentrations for correlates of protection has not been reached, this statement should be modified.

**Response:** Thank you for your comment. We have modified this statement (page 18, lines 589-591) as follows: “I*n cases where antibodies can be measured quantitatively, it may also be possible to use them to assess the level of protection in a population, although there is currently no consensus on antibody-based correlates of protection for SARS-CoV-2.”*

Reviewed by Professor Rosanna W Peeling

2022-06-14 Additional comments by Editor Krisztina Konzella

**1. Please amend your detailed Financial Disclosure statement on the submission form.  This is published with the article. It must therefore be completed in full sentences and contain the exact wording you wish to be published.**

- Please state what role the funders took in the study in the Financial Disclosure section. If the funders had no role in your study, please state: “The funders had no role in study design, data collection and analysis, decision to publish, or preparation of the manuscript.”

**Response:** We have added this into the responses to financial disclosure section of the online submission form as suggested, reading as follows: “*WHO had a role in the study design, data collection, data analysis, data interpretation, and writing of the report. No other funders had any such role.”*

- Please ensure that the funders and grant numbers match between the Financial Disclosure field and the Funding Information tab in your submission form. Note that the funders must be provided in the same order in both places as well.

**Response:** We have reviewed and made sure that the funders and grant numbers match and are in the same order in the Funding Information tab.

-Currently you have this in the Financial Disclosure section: "... the authors alone are responsible for the views expressed in this publication and they do not necessarily represent the decisions, policy, or views of WHO." Could you please put this in the Acknowledgements in the manuscript? Also, please clarify this statement (about not representing the decisions, policy, views of WHO) given that several authors have WHO affiliations, and authors have mentioned in the FD the roles of WHO in the study design, etc?

**Response:** We have agreed to alter this sentence in the Financial Disclosure section and elsewhere as we agree with your perspective here that based on our authorship team and role of WHO in the study, that the conclusions in this paper are represented by WHO. We added the following sentence to the Acknowledgements section, to clarify WHO does not implicate responsibility for any political designations regarding our map creation specifically:

“*The designations employed and the presentation of the material in this publication do not imply the expression of any opinion whatsoever on the part of WHO concerning the legal status of any country, territory, city or area or of its authorities, or concerning the delimitation of its frontiers or boundaries. Dotted and dashed lines on maps represent approximate border lines for which there may not yet be full agreement”*

**2. Please declare all competing interests beginning with the statement "I have read the journal's policy and the authors of this manuscript have the following competing interests:"**

-Please amended your Competing Interest statement to include a competing interest declared by your co-author, Dr. Tingting Yan using only their initials:

-"Separate to this work, I worked for Health Canada as a part-time Senior Policy Analyst with the COVID-19 Testing and Screening Expert Panel, from Nov 2020-Jun 2021."

**Response:** Added the following into the competing interests section: “*TY reports working for Health Canada as a part=time Senior Policy Analyst with the COVID-19 Testing and Screening Expert Panel, from Nov 2020-Jun 2021 (Outside of the submitted work).”*

-Please remove this sentence from the Competing Interest section and place it in the Financial Disclosure section on the online submission form: "WHO had a role in study design, data collection, data analysis, data interpretation, and writing of the report. No other funders had any such role."

**Response:** Removed and put in the online section as requested.

**3. We have noticed that you have uploaded Supporting Information files, but you have not included a list of legends. Please add a full list of legends for your Supporting Information files after the references list.**

**Response:** Thank you for the clarification, we have now added this in. We referred to other PLoS Med publications to do so. Please see lines 999-1016.

**4. Figure S1: please (a) provide a direct link to the base layer of the map used and ensure this is also included in the figure legend; (b) provide a link to the terms of use / license information for the base layer. We cannot publish proprietary or copyrighted maps (e.g. Google Maps, Mapquest) and the terms of use for your map base layer must be compatible with our CC-BY 4.0 license.**

If your map was obtained from a copyrighted source please amend the figure so that the base map used is from an openly available source. Alternatively, please provide explicit written permission from the copyright holder granting you the right to publish the material under our CC-BY 4.0 license.

Please note that the following CC BY licenses are compatible with PLOS license: CC BY 4.0, CC BY 2.0 and CC BY 3.0, meanwhile such licenses as CC BY-ND 3.0 and others are not compatible due to additional restrictions.

If you are unsure whether you can use a map or not, please do reach out and we will be able to help you. The following websites are good examples of where you can source open access or public domain maps:

* U.S. Geological Survey (USGS) - All maps are in the public domain. (<http://www.usgs.gov>)

* PlaniGlobe - All maps are published under a Creative Commons license so please cite “PlaniGlobe, <http://www.planiglobe.com>, CC BY 2.0” in the image credit after the caption. (<http://www.planiglobe.com/?lang=enl>)

* Natural Earth - All maps are public domain. (<http://www.naturalearthdata.com/about/terms-of-use/>)

**Response:** Thank you for the helpful clarification on the map copyright policy. We have updated and replaced Figure S1.

**5. We do not publish any copyright or trademark symbols that usually accompany proprietary names, eg (R), (C), or TM (e.g. next to drug or reagent names). Therefore please remove all instances of trademark/copyright symbols throughout the text, including 'TM' in the Reference section.**

**Response**: Removed throughout.
