# Supplementary material for: Global SARS-CoV-2 seroprevalence from January 2020 to April 2022: A systematic review and meta-analysis of standardized population-based studies
Source: PLoS Med. 2022 Nov 10;19(11):e1004107. doi: 10.1371/journal.pmed.1004107 (PMC9648705; doi:10.1371/journal.pmed.1004107)
Supplement: Attachment — Submitted filename: 2022-09-01 Response to Reviewers.docx [file pmed.1004107.s005.docx]

**2022-09-01 Response to Reviewers**

From the Academic Editor:

1. Thank you for your edit to the text of the Discussion at lines 587-589, to clarify that there is no consensus on antibody-based correlates of protection for SARS-CoV-2; however, several other places in the text refer to seroprevalence in helping estimate who is still susceptible. Please edit these accordingly:

**Response:**  Thank you for your comment. We have modified these instances to discuss exposure rather than susceptibility, in-text as follows:

-Line 48 "to estimate the extent of population infection and remaining susceptibility."

Response: Changed to “to estimate the extent of population infection and seropositivity to the virus”

-Line 79 "however around 40 % of the global population remains susceptible to SARS-CoV-2 infection"

**Response:** Changed to “however over one third of the global population are seronegative to the SARS-CoV-2 virus”

-Line 128 "Synthesizing these studies is crucial to understand the shifting global dynamics and true extent of SARS-CoV-2 infection, humoral immunity, and population susceptibility"

**Response:** Changed to “Synthesizing these studies is crucial to understand the shifting global dynamics and true extent of SARS-CoV-2 infection and humoral immunity”

-Lines 602-604 "Seroprevalence data can be used to compare seroprevalence between different groups (e.g. age, sex geography, etc.) to identify susceptible populations and thus inform decisions regarding the implementation of counter measures such as vaccination programs and PHSM [58]."

**Response:** Changed to “Seroprevalence data can be used to compare seroprevalence between different groups (e.g. age, sex geography, etc.) to identify vulnerable populations and thus inform decisions regarding the implementation of counter measures such as vaccination programs and PHSM [58].”

-Line 620 "Nevertheless, there is regional variation and 40 % of the global population remains susceptible to SARS-CoV-2"

**Response:** Changed to “Nevertheless, there is regional variation and 40 % of the global population are seronegative to SARS-CoV-2”

2. Title: Please change the timeframe of the title to reflect the sampling of specimens (e.g. April of 2022) rather than the publication of the articles.

**Response:** We have incorporated your suggestion into the title of the article.

3. Results: Please move the meta-regression from being the first data presented (at line 383) to being between the subgroup analysis and sensitivity analysis (e.g. before line 470).

**Response:** We have moved the meta-regression results as suggested.

4. Results: Line 407: In this section, rather than reporting increases as 6.6 fold or 2.2x, etc, please compare differences with a prevalence ratio and 95% CI if possible.

**Response:** Thank you for the comment. The primary outcome of this analysis is the seroprevalence point estimate and 95% CI from each separate meta-analysis for a given region-month group, not the ratio between estimates. Thus, we have replaced reporting increases (eg, 6.6 fold or 2.2x) with simply reporting the point estimate and 95% CI in the earlier and later month (eg, Overall seroprevalence in February 2021 was 42.7% [37.6-48.0%] in EMR (compared to 33.6% [32.8-34.4%] in June 2020). We feel this approach makes it easier to interpret the trajectory of seroprevalence over time, while calculating differences between groups of interest with a prevalence ratio and 95% CI is more appropriate for the meta-regression model.

5. Results: Line 428: This section (Ratios of seroprevalence to cumulative incidence) may be difficult to interpret, and it may be easier to understand the completeness of the case surveillance data if percentages (with the denominator representing case burden derived from seroprevalence, and numerator representing the number of reported cases) are used instead.

**Response:** Thank you for the comment. While we understand that ratios of seroprevalence to cumulative incidence may be difficult to interpret, we prefer to leave them in to facilitate comparing our results to other studies with the same outcome in the Discussion section. To aid interpretation, we have added the corresponding percentage of reported cases to each ratio in the text – e.g., *“Globally, the median ratio was 51.3 infections derived from seroprevalence to 1 reported case (51.3:1) in 2020 Quarter Three, suggesting that around 1.9% of cases were reported, and 10.5:1 in 2021 Quarter Three, suggesting that around 9.5% of cases were reported.”*

6. Living systematic review: We would be willing to consider submissions of future versions of the review mapping continued seroprevalence changes. However, publication decisions for any such updates would be dependent upon editorial and peer review.

**Response:** Thank you for your comment and interest in submissions of future iterations of our living review. We will take this strongly into consideration for future analyses.

7. Update to included seroprevalence data: You are welcome to update with more recent seroprevalence results (e.g. anything more recent since May 2022) if you would like to do so.

**Response:** Thank you for your suggestion. At this time, we would like to prioritize timely publication of the article as-is, but we are open to further submissions of updated data as mentioned above.

Other editorial comments:

8. Title: We suggest: “Global SARS-CoV-2 seroprevalence from January 2020 to April 2022: A systematic review and meta-analysis of standardized population-based studies” Please be sure to capitalize the first word of the subtitle.

**Response:**  Thank you for your title suggestion, we agree and have altered it accordingly.

9. Financial disclosure: Thank you for providing the initials of each author who was supported by each award. Please provide the specific grant numbers and URLs to sponsors’ websites where applicable.

**Response:** We have added all applicable grant numbers and URLs (if no number or URL was provided, there were none associated with that award).

10. Data availability: For the data repository, please provide the link to the most up to date version (<https://zenodo.org/record/6915823#.YwOfW3YXZaQ>).

“Other relevant data are available in a data repository (doi:10.5281/zenodo.5773152) and/or available from the Zenodo community upon reasonable request.” Please describe the data not available in the repository and please provide details for access/contact information for accessing the data.

**Response:** Thank you for your comment. We have now made all the data openly available, so the previous text is no longer relevant. The full data availability statement is below.

Please also mention that the Python code for the automated estimate prioritizaiton is available on GitHub and provide the link.

**Response:** We have made all the data available in public, open access repositories and have revised the data availability statement with links up to date as follows:

*“Machine-readable data files for dataset 0, sub-dataset 1, and sub-dataset 2 are available on the following Zenodo link:* [*https://doi.org/10.5281/zenodo.6915823*](https://doi.org/10.5281/zenodo.6915823)*. Raw early results submitted via the Unity Study Collaborators initiative are available on the following Zenodo link:* [*https://zenodo.org/communities/unity-sero-2021?page=1&size=20*](https://zenodo.org/communities/unity-sero-2021?page=1&size=20)*. DOIs and citations for the studies in this Zenodo community is included in the Supplementary File (S5). The Python code used for our automated estimate prioritization is available on the following GitHub link: https://github.com/serotracker/iit-backend/blob/8059e9b905395de997f28a1a2dff5def795276ad/app/utils/estimate_prioritization/estimate_prioritization.py.”*

11. Abstract: Line 69: Please clarify if this should be “in European high-income countries…” or other.

**Response:** Thank you for your comment. We have clarified this line to outline the estimates in European high-income countries as follows: “…in others (e.g., 9.6% [8.3-11.0%] in June 2020 to 95.9% [92.6-97.8%] in December 2021, European high-income countries [HIC] in December 2021”

12. Abstract: Please define abbreviations at first use in the text (LMIC, EUR HIC, AMR HIC).

**Response:** We have modified the text in the abstract so that the use of abbreviations are consistent and all defined properly in their first instance.

13. Abstract: Methods and Findings: In the last sentence of the Abstract Methods and Findings section, please describe the main limitation(s) of the study's methodology. Please move the sentence beginning “The main limitations of our methodology…” from line 61-63 to the end of the section.

**Response:** We have moved the sentence to the end of the section as suggested.

14. Introduction: Line 112-113: Please update the disease burden ( the numbers reported here are out of date).

**Response:** Thank you for the suggestion. We have updated the numbers to 7 September 2022.

15. Methods: Line 157-158: Please note where a complete list of the search terms can be found (e.g. in Supporting Information file S2.4).

**Response:** We have noted in parentheses that the complete list of search terms is in Supplement S2.4.

16. Methods: Line 247: It might be helpful to provide some definitions for geographical scope here.

**Response:** Thank you for the suggestion, we have already provided definitions for geographical scope in line 247:

*“We classified seroprevalence studies by geographical scope (local [i.e. cities, counties], sub-national [i.e. provinces or states], or national), sample frame, sampling method, and type of serological assay (Supplement S2.1 Table S1, Table 5).”*

17. Results: Line 334: Please clarify this to “Google Translate” in this sentence.

**Response:** Addressed.

18. Results: Line 384-396: Please report p values as p<0.001 where applicable.

**Response:** Addressed.

19. Results: Line 461: Please report p values as p<0.001 where applicable.

**Response:** Addressed.

20. Discussion: We suggest that subheadings (e.g. “summary” and “results in context”) could be removed.

**Response:** We have removed the subheadings as suggested.

21. Discussion: “We synthesized data from over 900 seroprevalence studies worldwide (43% from LMICs) published up to December 2021” Please clarify this sentence as there were a few studies from 2022.

**Response:** Thank you for noting this error. We have corrected the date to May 2022.

22. Discussion: Please italicize P. falciparum.

**Response:** Addressed.

23. Page 19: Please remove the contributors, declarations of interests, and data availability sections from the main text of the manuscript. Please ensure all information is completely and accurately entered into the relevant sections of the manuscript submission system.

**Response:** We have deleted the sections as stated. All information is reported in the manuscript submission system. There was no spot in the author portal for the contributors section - we have omitted as per your comment, but please let us know if there is a more suitable spot for it.

24. References: Please check the formatting of each reference. Please use the "Vancouver" style for reference formatting, and see our website for other reference guidelines <https://journals.plos.org/plosmedicine/s/submission-guidelines#loc-references>

**Response:** Thank you, we will ensure a thorough check of all our references.

25. References: Please double check the information for reference 2.

**Response:** This reference has been revised according to the guidelines linked above.

26. References: Please update reference 6.

**Response:** This reference has been updated as noted.

27. References: For reference 9 and reference 12 and reference 43, please change the journal abbreviation to PLoS One.

**Response:** We have changed the abbreviation in these references as noted.

28. Figure 2: Please use p<0.001 where applicable.

**Response**: Addressed.

29. Figure 3: Please make the headings indicating the WHO regions more visible (e.g. larger text). We suggest referring to “panels” in the legend to make it clear the top, middle, and bottom panels are consistent across all WHO regions presented. In the legend, please fully define GISAID.

**Response:** We have increased the font size of the headings. To make it clear what the “panels” refer to, we have added a box around each WHO region and added this text to the beginning of the legend:

*“The figure contains nine boxes showing the global analysis and eight WHO regional analyses. Each box contains the following panels.”*

We have also added the full definition of GISAID to the legend.

30. Figure 4: For the top panel, please indicate “years” on the left axis. Please use I2. Please use p<0.001 where applicable. Please define PR.

**Response**: We have modified the figure to indicate “years” on the left axis and use p<0.001. We have defined PR and I2 in the figure legend.

31. PRISMA checklist: Please revise the checklist, removing references to line numbers. Please refer to locations within the text using section and paragraph numbers only (e.g. Methods, paragraph 1).

**Response:** We have modified the checklist as you have suggested.

32. Supporting information file S2.2: Rather than inserting a link into the text, please provide a reference to the protocol.

**Response:** Addressed with reference [14].

33. Supporting information file S2.4: Please update the search dates.

**Response:** Addressed.

34. Supporting information: Figure S1: Please confirm that the appropriate usage rights apply to the use of this map. Please see our guidelines for map images: <https://journals.plos.org/plosmedicine/s/figures#loc-maps>

Response: We confirm that the data belongs in the public domain and appropriate usage rights apply to the use of this map. We have added the source for the base layer of the map to the Figure S1 legend:

*“Base layer of the map used are from ArcGIS, ESR: https://who.maps.arcgis.com/home/item.html?id=a9bedbe28edf4c4eb835b93ae3b88ce0”*

35. Supporting information Table S11: Please briefly describe the different models in the legend. Please also define AIC and BIC in the legend.

Response: We have added a description of the different models in the legend and column headings of Table S11. We have also defined AIC and BIC in the legend.

36. Supporting Information S5: References: Please use the "Vancouver" style for reference formatting, and see our website for other reference guidelines <https://journals.plos.org/plosmedicine/s/submission-guidelines#loc-references>

**Response:** We have reviewed our references in the supporting information file according to your reference guidelines.

Comments from Reviewers:

Reviewer #2: The authors have addressed all my extensive comments.

Michael Dewey

**Response:** Thank you for your revisions on our paper, we are glad you are satisfied with the changes.

Reviewer #3: The authors have addressed all major comments brought up in my earlier review. I recommend a thorough grammatical check prior to publication.

**Response:** Thank you for your comment and revisions. Our author team will do a thorough grammar check.

Reviewer #4: The authors have provided comprehensive responses to the editorial and reviewers' comments and suggestions. This work will be useful in providing the rationale and framework for future seroprevalence studies during infectious disease outbreaks nationally, regionally and globally. I have no further comments.

**Response**: Thank you very much for your positive comments on the usefulness of our paper. We appreciate your revisions and feedback.
